# Supplementary material for: A synaptomic analysis reveals dopamine hub synapses in the mouse striatum
Source: Nat Commun. 2022 Jun 3;13:3102. doi: 10.1038/s41467-022-30776-9 (PMC9166739; doi:10.1038/s41467-022-30776-9)
Supplement: Supplementary file 1 — Supplementary Information [file 41467_2022_30776_MOESM1_ESM.pdf]

## A synaptomic analysis reveals dopamine hub synapses in the mouse striatum.

### Table of Contents

**SUPPLEMENTARY FIGURE 2: IMMUNOFLUORESCENCE OF DA-FASS SYNAPTOSOMES. ........3**

**SUPPLEMENTARY FIGURE 3: META-ANALYSIS OF ENRICHED PROTEINS WITH SINGLE CELL RNA DATABASES. ........4**

**SUPPLEMENTARY FIGURE 4: CHARACTERIZATION OF THALAMO-STRIATAL HUB SYNAPSES. ........5**

**SUPPLEMENTARY FIGURE 5: POSITIVE AND NEGATIVE CONTROLS TO DOPAMINE HUB SYNAPSE IDENTIFICATION. ........6**

**SUPPLEMENTARY FIGURE 6: CHARACTERIZATION OF VGLUT1/TH HUB SYNAPSES. ........8**

**SUPPLEMENTARY TABLE 1: ANTIBODIES LIST AND DILUTION ........9**

**SUPPLEMENTARY TABLE 2: STATISTICAL ANALYSIS ........12**

**SUPPLEMENTARY TABLE 3: PROTEOMICS DATASET ........20**

**SUPPLEMENTARY TABLE 4: PROTEOMICS COMPARISON WITH SHARMA ET AL. ........83**

**SUPPLEMENTARY TABLE 5: PROTEOMICS COMPARISON WITH KEGG NEUROTRANSMISSION PATHWAYS ........333**

**SUPPLEMENTARY DATA: UNCROPPED IMAGES FROM CAPILLARY IMMUNOBLOT EXPERIMENTS ........337**

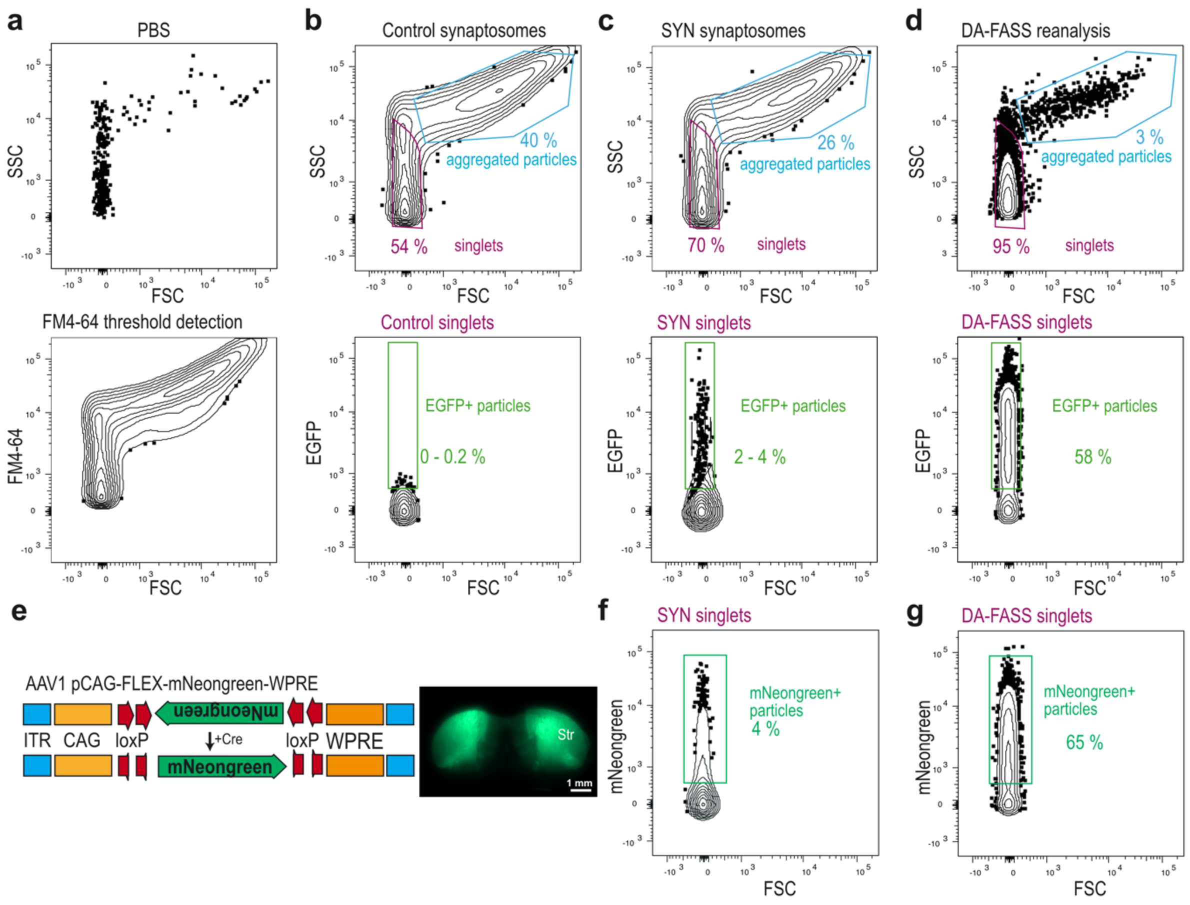


**Supplementary Figure 1: DA-FASS gating strategy. a-d** Representative FASS gate settings and particles detection for EGFP+ synaptosomes sorting. **a** Analysis of PBS allows defining the background noise of the thresholding using FM4-64 lipophilic styryl dye used. The noise is less than 500 events per minute. Particle detection using FM4-64 thresholding (bottom). **b** Side scatter (SSC) and forward scatter (FSC) analysis of synaptosomes allows defining aggregated particles (40 %, light blue gate) and singlets (54 %, magenta gate). As published previously, singlets gate was defined experimentally through trials and error [21]. Singlets are further analysed for EGFP fluorescence. Control synaptosomes display low autofluorescence. **c** SYN synaptosomes samples showed 26 % of aggregated particles and 70 % of singlets on this example. 2-4 % of the singlets were significantly fluorescent in the EGFP channel. **d** Sorted particles gated as “singlets” and “EGFP+” were re-analysed showing a drop in the proportion

of aggregated particles (3 %) and a steep rise in singlets (95 %). Up to 58 % of singlets were EGFP+. **e** Cre-dependent AAV expressing mNeongreen and mNeongreen striatal expression. Scale bar = 1mm. **f** Before sorting 4 % of the singlets were significantly fluorescent in the mNeongreen channel. **g** Same as d, after sorting up to 65 % of singlets were mNeongreen+ in the DA-FASS sample.

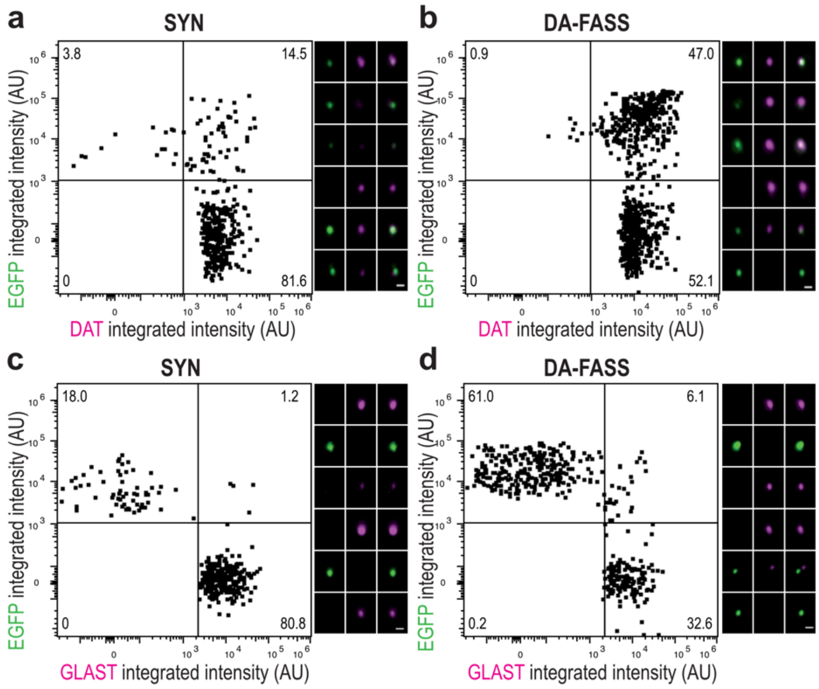


**Supplementary Figure 2: Immunofluorescence of DA-FASS synaptosomes. a-b** Dot plots of SYN and DA-FASS synaptosomes stained for the dopamine transporter DAT (x-axis) EGFP (y-axis) and galleries of representative epifluorescence images of individual synaptosomes. The population of particles positive for both EGFP and DAT (upper right quadrant) increases from 15 % in the SYN to 47 % in the DA-FASS sample. **c-d** Dot plots of intensity signal of singlets and DA-FASS synaptosomes stained for EGFP and the astrocyte membrane marker Slc1a3/GLAST and galleries of representative images. Note the very low representation of double positive particles. Scale bar = 1 µm.

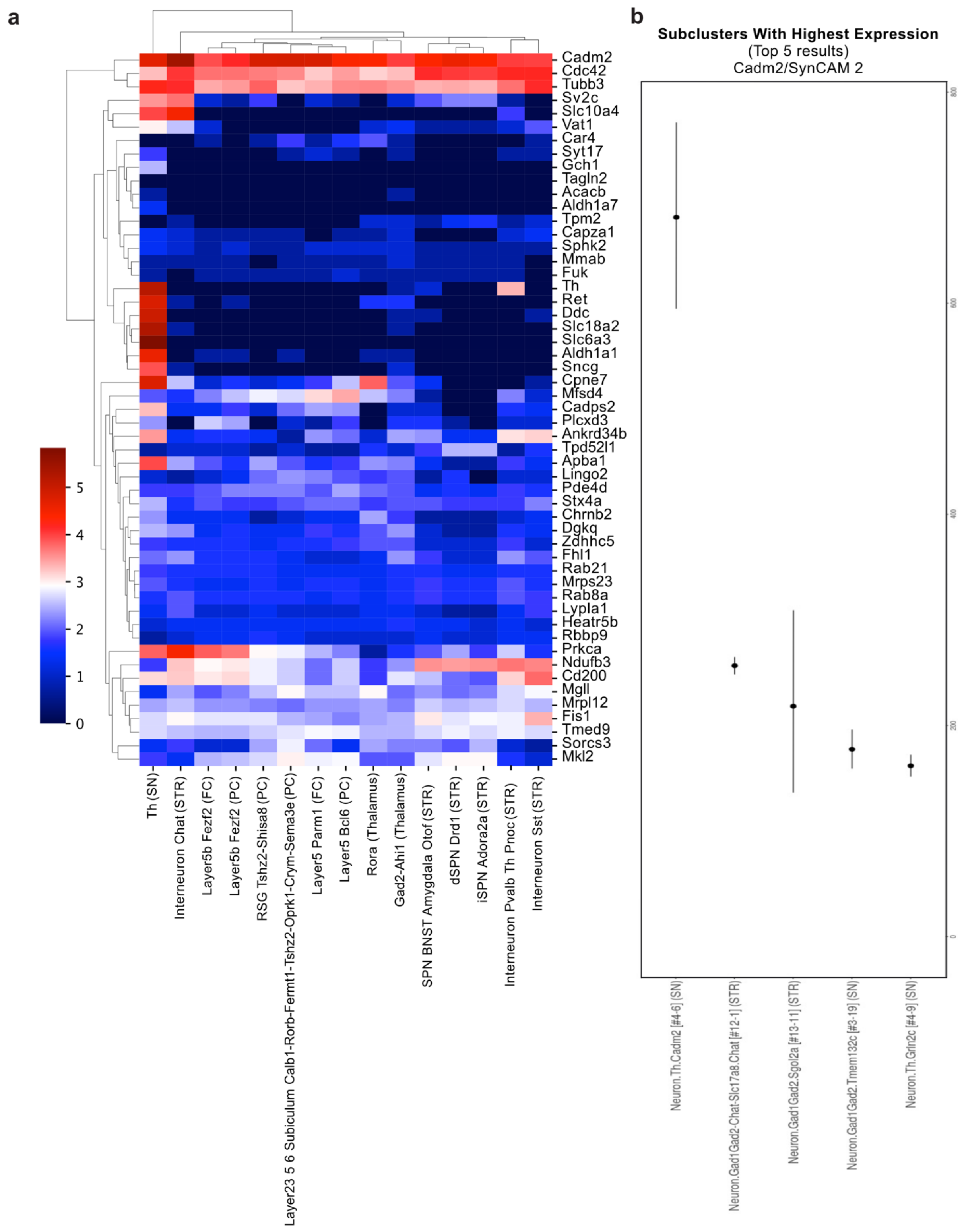


**Supplementary Figure 3: Meta-analysis of enriched proteins with single cell RNA databases. a** Extended Heatmap showing cell type specific mRNA abundance in neuronal cells from or projecting to the striatum (STR; Substancia Nigra, SN; Frontal Cortex, FC; Posterior Cortex, PC; Thalamus) of 53 of the enriched DA-FASS proteins present in the DropViz single

cell RNA sequencing database (Stx4a = Stx4, Mfsd4 = Mfsd4a, Fuk = Fcsk, Car4 = Ca4, Mkl2 = Mrtfb) (DropViz; [35]). **b** Drop Viz representation of Cadm2/ SynCAM 2 brain cellular subclusters with highest expression. Data represented per 100 000 transcripts in sub cluster. The error bars represent binomially distributed sampling noise given the number of cells in each subcluster; it does not represent heterogeneity in expression among cells within the subcluster. Dropviz data is obtained from at least N=4 independent replicates. Note that the highest Cadm2/SynCAM 2 mRNA expression throughout the brain is found in a sub cluster of substantia nigra Th neurons. Statistical significance was  not analysed in this figure.

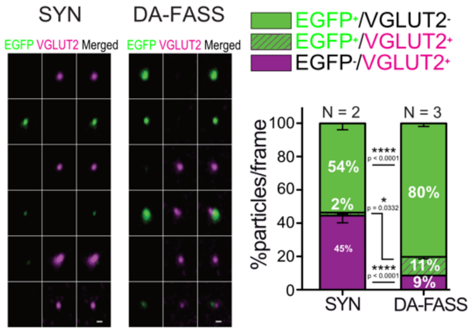


**Supplementary Figure 4: Characterization of thalamo-striatal hub synapses.** Epifluorescence images of a representative sample of synaptosome populations (singlets and DA FASS) labelled with anti-EGFP and anti-VGLUT2, labelling mainly thalamo-striatal terminals. Analysis of particle proportions per frame. The $EGFP^+/VGLUT2^+$ population increases from 2 % to 11 %. All data are mean ± SEM and pulled from 2 to 3 independent sorts. Statistical significance was analysed using Two-way ANOVA, EGFP/VGLUT2: Interaction $F_{2,54} = 80.90$ ****$p < 0.0001$, Condition $F_{1,54} = 0.0001$ $p = 0.991$, Immunolabelling $F_{2,54} = 293.7$ ****$p < 0.0001$ with Šídák's multiple comparisons test. Scale bar = 1 µm.

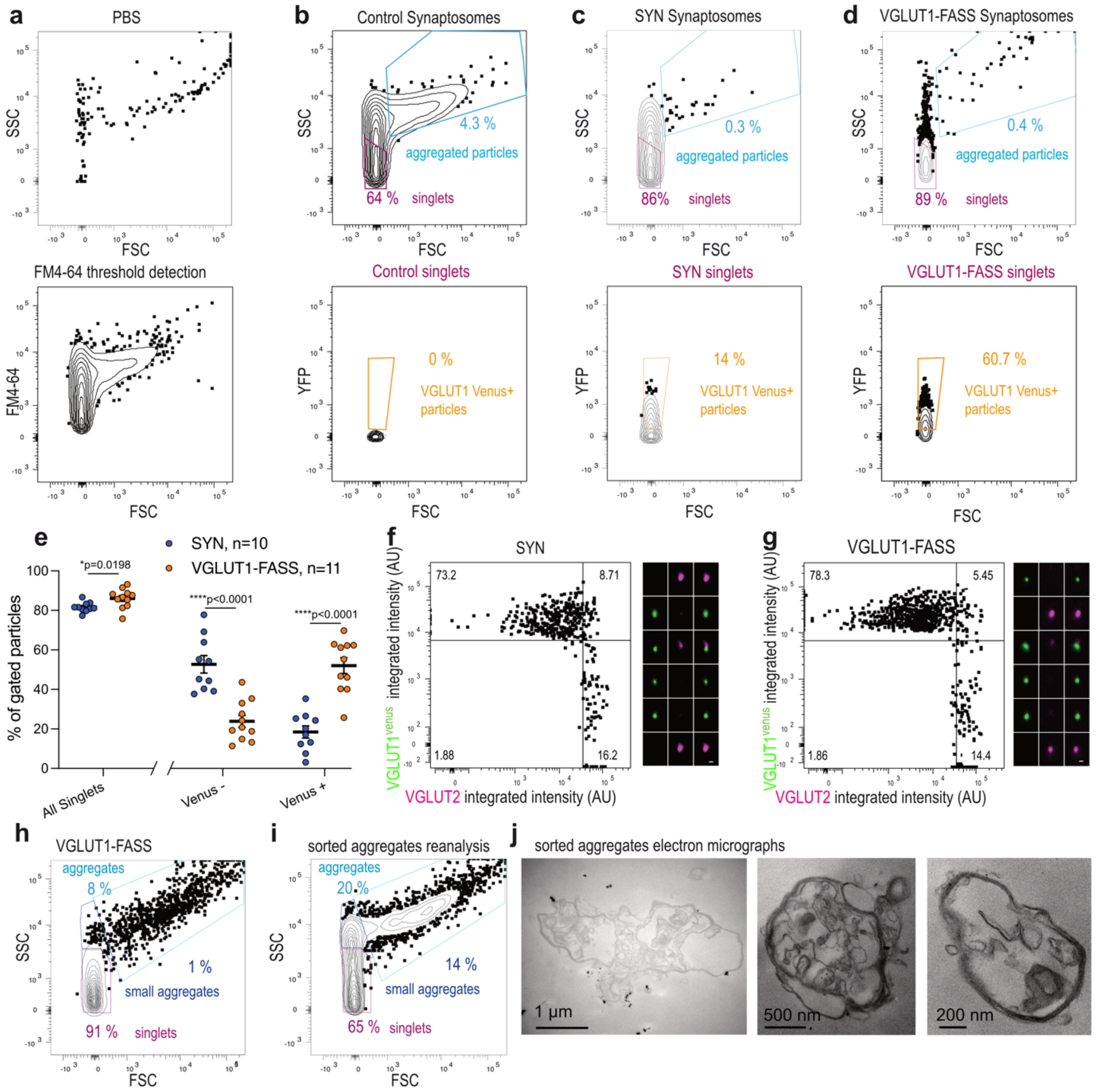

**Supplementary Figure 5: Positive and negative controls to dopamine hub synapse identification. a** Analysis of a PBS sample was used to define background noise in FM4-64 lipophilic styryl dye used for thresholding (top). The noise was less than 500 events per minute. Synaptosome sample detection using FM4-64 thresholding (bottom). **b** WT synaptosomes display aggregated particles (4.3 %, light blue) and singlets (64 %, magenta). Singlets gate was defined experimentally through trials and error as published previously (Luquet et al., 2017). Singlets were further analysed for Venus fluorescence to determine the autofluorescence level. **c** SYN synaptosomes samples showed 0.3 % of aggregated particles and 86 % of singlets. 14

% of synaptosomes were detected in the Venus$^+$ gate. **d** VGLUT1-FASS "singlets" were re-analysed and displayed 0.4 % of aggregated particles and 89 % singlets. Up to 60.7 % of sorted singlets were detected in the Venus+ gate. **e** Average striatal VGLUT1$^{venus}$ FASS results. SYN (blue) and VGLUT1-FASS (orange) samples for the different gates: all singlets, Venus$^{-}$, Venus$^+$. Data are mean ± SEM and was obtained through at least 10 independent sorts. Statistical significance was analysed using Two-tailed unpaired t-test for all singlets SYN – VGLUT1-FASS *p = 0.02 and Two-way ANOVA for VENUS$^{-/+}$ singlets: Interaction $F_{1,38}$ = 72.11 ****p < 0.0001, Gating $F_{1,38}$ = 0.668 p = 0.419, Condition $F_{1,38}$ = 0.412 p = 0.525 with Šídák's multiple comparisons test. **f-g** Dot plots SYN and VGLUT1-FASS synaptosomes stained for VGLUT1$^{Venus}$ and VGLUT2 with galleries of representative epifluorescence images. Note the low representation of synaptosomes associating VGLUT1 (cortico-striatal inputs) and VGLUT2 (Thalamo-striatal inputs). Indeed VGLUT1/-2 double positives do not enrich through FASS. Scale bar = 1 µm. **h-i** Representative FASS gating for aggregates sorting. **h** Sucrose/Ficoll VGLUT1$^{venus}$ synaptosome samples showed 8 % of aggregated particles (light blue). **i** Particles gated as "aggregates" and large tissue fragments were sorted and reanalysed. Small and large aggregates represented 34 % of particles after sorting. **j** Electron micrographs of sorted aggregated particles coming from one single experiment. Aggregates appear much larger than hub synapses. Their cellular content is difficult to identify though myelin membranes may be recognized on some of them. Scale bar, 1µm, 500nm, 200nm.

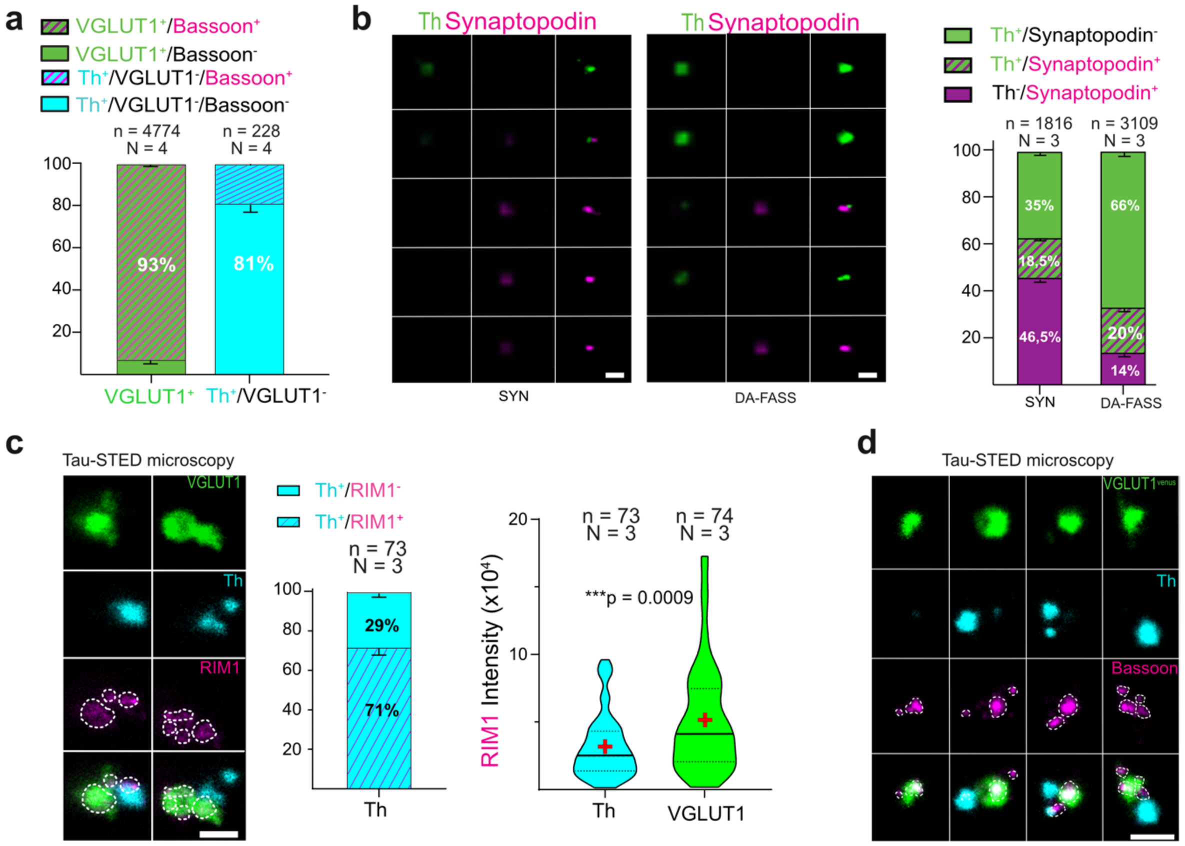


**Supplementary Figure 6: Characterization of VGLUT1/Th hub synapses. a** Proportion of Bassoon in VGLUT1$^{Venus+}$ or Th$^{+}$/VGLUT1$^{Venus-}$ synaptosomes. **b** Epifluorescence images of a representative sample of synaptosome populations (SYN and DA-FASS) labelled with anti-Th (green) and anti-Synaptopodin1 (magenta) and analysis of Th and Synaptopodin particle proportions per frame. Data are expressed as Mean ± SEM and pulled from 3 independent experiments. Statistical significance was analysed using Two-way ANOVA, Th/Synaptopodin: Interaction $F_{2,255}$ = 251.5 ****$p < 0.0001$, Condition $F_{1,255}$ = 7.774e-006 $p = 0.998$, Immunolabelling $F_{2,255}$ = 297.4 ****$p < 0.0001$. **c** Representative STED images of VGLUT1$^{Venus}$/Th/RIM1 synaptosomes, quantitative analysis of RIM1 proportion in Th+ synaptosome and of RIM1 intensity in Th+ or in VGLUT1$^{Venus}$+ synaptosome in Th-VGLUT1$^{Venus}$ hubs. Data is expressed as Mean ± SEM and pulled from 3 independent experiments. Statistical significance was analysed using Mann Whitney test for intensities,

RIM1: ***p = 0.0009. **d** Additional representative STED images of VGLUT1$^{Venus}$/Th/Bassoon synaptosomes coming pulled from 3 independent experiments. Scale bar = 1 µm

**Supplementary Table 1: Antibodies list And Dilution**

| Primary Antibodies | Source | Identifier | WES (25 ng/µL of protein) | Immuno Fluo |
|---|---|---|---|---|
| Rat monoclonal anti-SynCAM 2 | Thomas Biederer, Yale School of Medicine | N/A, Clone 3E1, No lot number | N/A | 1/200 |
| Goat polyclonal anti-Dopamine D1 receptor | Frontier Institute | Cat# D1R-Go-Af1000, RRID: AB_2571594, No lot number info not possible to retrieve from collaborator | N/A | 1/1000 |
| Guinea pig polyclonal anti-VGLUT2 | Millipore | Cat# AB2251, RRID:AB_1587626, Lot 2879548 | N/A | 1/1000 |
| Guinea pig polyclonal anti-VGLUT1 | Millipore | Cat# AB5905, RRID:AB_2301751, Lot 3616495 | N/A | 1/3000 |
| Guinea pig polyclonal anti-MGL Mgll | Frontier Institute | Cat# MGL-GP, RRID:AB_2716807, Lot Af200 | N/A | 1/200 |
| Guinea pig polyclonal anti-Homer-1c | Synaptic Systems | Cat# 160 004, RRID:AB_10549720, Lot 2-16 | N/A | 1/500 |
| Mouse monoclonal anti-GFP | Roche | Cat# 11814460001, RRID:AB_390913, Clone 7.1 and 13.1, Lot 27575600 | 1/100 | 1/1000 |
| Mouse monoclonal anti-Munc-18 | BD Biosciences | Cat# 610336, RRID:AB_397726, Clone 31, No lot number info not possible to retrieve from collaborator | 1/500 | N/A |
| Mouse monoclonal anti-Synaptophysin 1 | Synaptic Systems | Cat# 101 011C3, RRID:AB_887822, Clone 7.2, Lot 1-61 | 1/100 | N/A |
| Mouse monoclonal anti-Tyrosine hydroxylase | Millipore | Cat# MAB318, RRID:AB_2201528, Clone LNC1, Lot 3506431 | 1/100 | 1/3000 |
| Mouse monoclonal anti-PSD-95 | Abcam | Cat# ab2723, RRID:AB_303248, Clone 6G6-1C9, Lot GR3589-1 | N/A | 1/400 |
| Rabbit polyclonal anti-Tyrosine hydroxylase | Synaptic Systems | Cat# 213 102, RRID:AB_2619896, Lot 213102/2 | N/A | 1/500 |
| Rabbit polyclonal anti-Dopamine D2 receptor | Millipore | Cat# AB5084P, RRID:AB_2094980, No lot number info not possible to retrieve from collaborator | N/A | 1/200 |

| | | | | |
|---|---|---|---|---|
| Rabbit polyclonal anti-Synapsin 1/2 | Synaptic Systems | Cat# 106 002, RRID:AB_887804, Lot 106002/16 | N/A | 1/2000 |
| Rabbit polyclonal anti-VGLUT2 | E Herzog | Cat# VGLUT2, RRID:AB_2315563, Lot N/A | N/A | 1/1000 |
| Rabbit polyclonal anti-Synaptopodin | Synaptic Systems | Cat# 163 002, RRID:AB_887825, Lot 163002/7 | N/A | 1/800 |
| Rabbit polyclonal anti-VAChT | Synaptic Systems | Cat# 139 103, RRID:AB_887864, Lot 5-67 | N/A | 1/1000 |
| Rabbit polyclonal anti-VIAAT/VGATs | Synaptic Systems | Cat# 131 002, RRID:AB_887871, Lot 31002/240 | N/A | 1/4000 |
| Rabbit polyclonal anti-DAT | Millipore | Cat# AB2231, RRID: AB 1586991, Lot 3417479 | 1/100 | 1/2000 |
| Rabbit polyclonal anti-EAAT1/GLAST | Niels Christian Danbolt, University of Oslo | Cat# Ab#314, RRID:AB_2314561, Lot 1998-07-29 | 1/250 | 1/10000 |
| Rabbit polyclonal anti-GluA1 | Millipore | Cat# AB1504, RRID:AB_2113602, Lot 2207172 | 1/50 | N/A |
| Rabbit polyclonal anti-GFP | Abcam | Cat# ab290, RRID:AB_303395, Lot GR3251545-1 | N/A | 1/3000 |
| Rabbit polyclonal anti-Cpne7 | OriGene | Cat# TA334534, RRID: N/A, Lot QC28458/40659 | N/A | 1/500 |
| Rabbit polyclonal anti-Mint 1 Apba1 | Synaptic Systems | Cat# 144 103, RRID:AB_10635158, Lot 4 | N/A | 1/300 |
| Rabbit polyclonal anti-Cadps2 | Synaptic Systems | Cat# 262 103, RRID:AB_2619980, Lot 2 | N/A | 1/400 |
| Rabbit polyclonal anti-Syntaxin 4 | Synaptic Systems | Cat# 110 042, RRID:AB_887853, Lot 21 | N/A | 1/1000 |
| Rabbit polyclonal anti-Bassoon | Synaptic Systems | Cat# 141 003, RRID:AB_887697, Lot 1-25 | N/A | 1/750 |
| Rabbit polyclonal anti-RIM1 | Synaptic Systems | Cat# 140 013, RRID:AB_2238250, Lot 2-13 | N/A | 1/500 |
| Chicken polyclonal anti-Tyrosine hydroxylase | Millipore | Cat# AB9702, RRID:AB_570923, Lot 3616562 | N/A | 1/1000 |
| Chicken polyclonal anti-GFP | Abcam | Cat# ab13970, RRID:AB_300798, Lot GR3361051-2 | N/A | 1/3000 |
| Secondary Antibody | Source | Identifier, | WES (25 ng/µL of protein) | Immuno Fluo |
| Goat polyclonal Anti Mouse Alexa 488 | Thermo Fisher | Cat# A-11001, RRID: AB_2534069 , Lot 2379467 | N/A | 1/500 |

| | | | | |
|---|---|---|---|---|
| Goat polyclonal Anti Rabbit Alexa 568 | Thermo Fisher | Cat# A-11011, RRID:AB_143157, Lot 1778025 | N/A | 1/500 |
| Goat polyclonal Anti-Chicken Alexa 647 | Thermo Fisher | Cat# A-21449, RRID:AB_2535866, Lot 2186435 | N/A | 1/500 |
| Goat polyclonal Anti-Chicken Alexa 594 | Thermo Fisher | Cat# A-11042, RRID:AB_2534099, Lot 2181000 | N/A | 1/500 |
| Goat polyclonal Anti Rabbit Atto-647 | Sigma-Aldrich | Cat# 40839, RRID:AB_1137669, Lot BCCF3161 | N/A | 1/500 |
| Goat polyclonal Anti-Chicken Alexa 488 | Thermo Fisher | Cat# A-11039, RRID:AB_2534096, Lot 2304258 | N/A | 1/500 |
| Goat polyclonal Anti-Guinea Pig Alexa 647 | Thermo Fisher | Cat# A-21450, RRID:AB_2735091, Lot 2231672 | N/A | 1/500 |
| Goat polyclonal Anti-Mouse Alexa 647 | Thermo Fisher | Cat# A-21235, RRID:AB_2535804, Lot 2306581 | N/A | 1/500 |
| Goat polyclonal Anti-Mouse Alexa 568 | Thermo Fisher | Cat# A-11004, RRID:AB_2534072, Lot 1419715 | N/A | 1/500 |
| Goat polyclonal Anti Rabbit Alexa-647 | Thermo Fisher | Cat# A-21244, RRID:AB_2535812, Lot 1910774 | N/A | 1/500 |
| Goat polyclonal Anti Rabbit Alexa 488 | Thermo Fisher | Cat# A-11008, RRID:AB_143165, Lot 1705869 | N/A | 1/500 |

**Supplementary Table 2: Statistical Analysis**

| Figure | Variable | Condition | (Mean ± SEM) N, n | Source of Variation F (DFn, DFd) p value | Post-Hoc P value summary | Test |
|---|---|---|---|---|---|---|
| Fig. 1d | Th | H | 1 ± 0.26; N = 3 | Interaction F8,36 = 0.2733 p = 0.9706 | | Two-way ANOVA |
| | | P1 | 0.36 ± 0.07; N = 3 | Fraction F8,36 = 7.062 ****p<0.0001 | | |
| | | S2 | 0.77 ± 0.1; N = 3 | Protein F1,36 = 2.387 p = 0.1311 | | |
| | | P2 | 1.2 ± 0.27; N = 3 | | | |
| | | Syn | 1.5 ± 0.31; N = 3 | | | |
| | | LS1 | 2.2 ± 0.19; N = 3 | | | |
| | | SPM | 0.26 ± 0.02; N = 3 | | | |
| | | LS2 | 1.58 ± 0.32; N = 3 | | | |
| | | LP2 | 0.82 ± 0.4; N = 3 | | | |
| | | | | | | |
| | GFP | H | 1 ± 0.53; N = 3 | | | |
| | | P1 | 0.23 ± 0.09; N = 3 | | | |
| | | S2 | 0.49 ± 0.23; N = 3 | | | |
| | | P2 | 1.12 ± 0.52; N = 3 | | | |
| | | Syn | 1.48 ± 0.71; N = 3 | | | |
| | | LS1 | 1.8 ± 0.5; N = 3 | | | |
| | | SPM | 0.07 ± 0.02; N = 3 | | | |
| | | LS2 | 1.29 ± 0.22; N = 3 | | | |
| | | LP2 | 0.03 ± 0.01; N = 3 | | | |
| Fig. 1f | All Singlets | SYN | 73.79 ± 4.608; N = 9 | | *p = 0.0104 | Mann-Whitney test |
| | | DA-FASS | 83.91 ± 1.252; N = 8 | | | |
| | | | | | | |
| | DA-FASS | EGFP - Singlets | 30.94 ± 2.75; N = 8 | Interaction F1, 30 = 218.3 ****P<0.0001 | SYN - DA-FASS | Two-way ANOVA |
| | | EGFP + Singlets | 48.94 ± 2.30; N = 8 | Gating F1, 30 = 66.42 ****P<0.0001 | EGFP - Singlets ****p<0.0001 | |
| | SYN | EGFP - Singlets | 66.14 ± 3.97; N = 9 | Condition F1, 30 = 3.302 P = 0.0792 | EGFP + Singlets ****p<0.0001 | |
| | | EGFP + Singlets | 3.86 ± 0.53; N = 9 | | | |
| | | | | | | |
| Fig. 2c | EGFP-/Th+ | SYN | 29,915 ± 4.65 n = 13 | Interaction F2, 96 = 65.04 ****p<0.0001 | SYN - DA-FASS | Two-way ANOVA |
| | | DA-FASS | 10.027 ± 0.825 n = 21 | Condition F1, 96 = 0.03371 p = 0.8547 | EGFP-/Th+ ****p<0.0001 | |
| | | | | Immunolabelling F2, 96 = 510.3 ****p<0.0001 | EGFP+/Th+ ****p<0.0001 | |
| | EGFP+/ Th+ | SYN | 57.400 ± 2.81 n = 13 | | EGFP +/Th- p = 0.2534 | |

| | | | | | | |
|---|---|---|---|---|---|---|
| | | DA-FASS | 83.095 ± 1.131 n = 21 | | | |
| | | | | | | |
| | EGFP +/Th- | SYN | 11.772 ± 2.039 n = 13 | | | |
| | | DA-FASS | 6.882 ± 1.177 n = 21 | | | |
| Fig. 2g | EGFP- /D1R+ | SYN | 86.81 ± 1.388 n = 12 | Interaction F2, 96 = 208,0 ****p<0,0001 | Singlets - FASS | Two-way ANOVA |
| | | DA-FASS | 43.241 ± 2.035 n = 22 | Condition F1, 96 = 0,007420 p = 0,9315 | EGFP-/D1R+ ****p<0.0001 | |
| | | | | Immunolabelling F2, 96 = 437,9 ****p<0,0001 | EGFP+/D1R+ ****p<0.0001 | |
| | EGFP+/ D1R+ | SYN | 3.21 ± 0.715 n = 12 | | EGFP+/D1R- ****p<0.0001 | |
| | | DA-FASS | 28.585 ± 1.97 n = 22 | | | |
| | | | | | | |
| | EGFP+/ D1R- | SYN | 9.496 ± 1.281 n = 12 | | | |
| | | DA-FASS | 28.081 ± 1.416 n = 22 | | | |
| Fig. 2k | EGFP- /D2R+ | SYN | 72.19 ± 3.03 n = 32 | Interaction F2, 180 = 149.4 ****p<0.0001 | SYN - DA-FASS | Two-way ANOVA |
| | | DA-FASS | 32.328 ± 2.42 n = 30 | Condition F1, 180 = 0.6714 p = 0.4136 | D2R+/EGFP- ****p<0.0001 | |
| | | | | Immunolabelling F2, 180 = 163.5 ****p<0.0001 | EGFP+/D2R+ ****p<0.0001 | |
| | EGFP+/ D2R+ | SYN | 15.20 ± 2.27 n = 32 | | EGFP+/D2R- p = 0.1382 | |
| | | DA-FASS | 53.24 ± 2.27 n = 30 | | | |
| | | | | | | |
| | EGFP+/ D2R- | SYN | 8.18 ± 1.80 n = 32 | | | |
| | | DA-FASS | 14.55 ± 1.38 n = 30 | | | |
| Fig. 4b | Th- /Cpne7+ | SYN | 42.61 ± 2.4 n = 31 | Interaction F2, 180 = 131.9 ****p<0.0001 | SYN - DA-FASS | Two-way ANOVA |
| | | DA-FASS | 16.21 ± 1.28 n = 31 | Condition F1, 180 = 0.0004 p = 0.9841 | Th-/Cpne7+ ****p<0.0001 | |
| | | | | Immunolabelling F2, 180 = 570.4 ****p<0.0001 | Th+/Cpne7+ p = 0.9951 | |
| | Th+/Cpn e7+ | SYN | 8 ± 0.88 n = 31 | | Th+/Cpne7- ****p<0.0001 | |
| | | DA-FASS | 8.49 ± 0.44 n = 31 | | | |
| | | | | | | |
| | Th+/Cpn e7- | SYN | 49.3 ± 2.35 n = 31 | | | |
| | | DA-FASS | 75.29 ± 1.32 n = 31 | | | |
| Fig. 4c | Th- /Mint-1+ | SYN | 66.14 ± 3.09 n = 30 | Interaction F2, 183 = 163.7 ****p<0.0001 | SYN - DA-FASS | Two-way ANOVA |
| | | DA-FASS | 29.92 ± 1.27 n = 33 | Condition F1, 183 = 0.0009 p = 0.9754 | Th-/Mint-1+ ****p<0.0001 | |
| | | | | Immunolabelling F2, 183 = 316.5 ****p<0.0001 | Th+/Mint-1+ p = 0.2048 | |
| | Th+/Min t-1+ | SYN | 3.56 ± 0.5 n = 30 | | Th+/Mint-1- ****p<0.0001 | |

| | | | | | | |
|---|---|---|---|---|---|---|
| | | DA-FASS | 8.35 ± 0.65 n = 33 | | | |
| | | | | | | |
| | Th+/Mint-1- | SYN | 30.05 ± 2.99 n = 30 | | | |
| | | DA-FASS | 61.34 ± 1.33 n = 33 | | | |
| Fig. 4d | Th-/Cadps2+ | SYN | 57.71 ± 3.33 n = 30 | Interaction F2, 187 = 110.5 ****p<0.0001 | SYN - DA-FASS | Two-way ANOVA |
| | | DA-FASS | 22.63 ± 1.1 n = 34 | Condition F1, 187 = 0.004 p = 0.9511 | Th-/Cadps2+ ****p<0.0001 | |
| | | | | Immunolabelling F2, 187 = 88.12 ****p<0.0001 | Th+/Cadps2+ *p=0.0434 | |
| | Th+/Cadps2+ | SYN | 12.99 ± 1.65 n = 30 | | Th+/Cadps2- ****p<0.0001 | |
| | | DA-FASS | 20.54 ± 1.49 n = 34 | | | |
| | | | | | | |
| | Th+/Cadps2- | SYN | 28.97 ± 3.41 n = 31 | | | |
| | | DA-FASS | 56.83 ± 1.11 n = 34 | | | |
| Fig. 4e | DAT-/SynCAM2+ | SYN | 53.18 ± 2.38 n = 12 | Interaction F2, 60 = 84.92 ****p<0.0001 | SYN - DA-FASS | Two-way ANOVA |
| | | DA-FASS | 18.87 ± 3.05 n = 10 | Condition F1, 60 = 4.371e-005 p = 0.9947 | DAT-/SynCAM2+ ****p<0.0001 | |
| | | | | Immunolabelling F2, 60 = 73.75 ****p<0.0001 | DAT+/SynCam2+ ****p<0.0001 | |
| | DAT+/SynCam2+ | SYN | 28.69 ± 1.56 n = 12 | | DAT+/SynCam2- p = 0.1265 | |
| | | DA-FASS | 71.76 ± 5.67 n = 10 | | | |
| | | | | | | |
| | DAT+/SynCam2- | SYN | 18.18 ± 1.93 n = 12 | | | |
| | | DA-FASS | 9.37 ± 2.81 n = 10 | | | |
| Fig. 4f | Th-/Stx4+ | SYN | 62.45 ± 3.11 n = 31 | Interaction F2, 177 = 69.25 ****p<0.0001 | SYN - DA-FASS | Two-way ANOVA |
| | | DA-FASS | 29.18 ± 1.74 n = 30 | Condition F1, 177 = 0.004 p = 0.9497 | Th-/Stx4+ ****p<0.0001 | |
| | | | | Immunolabelling F2, 177 = 49.15 ****p<0.0001 | Th+/Stx4+ ****p<0.0001 | |
| | Th+/Stx4+ | SYN | 7.16 ± 1.22 n = 31 | | Th+/Stx4- p = 0.1185 | |
| | | DA-FASS | 33.31 ± 3.08 n = 30 | | | |
| | | | | | | |
| | Th+/Stx4- | SYN | 30 ± 2.66 n = 31 | | | |
| | | DA-FASS | 37.51 ± 3.06 n = 30 | | | |
| Fig. 4g | Th-/Mgll+ | SYN | 31.18 ± 2.17 n = 33 | Interaction F2, 192 = 98.57 ****p<0.0001 | SYN - DA-FASS | Two-way ANOVA |
| | | DA-FASS | 8.83 ± 0.57 n = 33 | Condition F1, 192 = 4.217e-005 p = 0.9948 | Th-/Mgll+ ****p<0.0001 | |
| | | | | Immunolabelling F2, 192 = 1242 ****p<0.0001 | Th+/Mgll+ **p = 0.0015 | |

| | | | | | | |
|---|---|---|---|---|---|---|
| | Th+/Mgll+ | SYN | 3.22 ± 0.54 n = 33 | | Th+/Mgll- ****p<0.0001 | |
| | | DA-FASS | 10.28 ± 0.95 n = 33 | | | |
| | | | | | | |
| | Th+/Mgll- | SYN | 65.48 ± 2.08 n = 33 | | | |
| | | DA-FASS | 80.79 ± 1.17 n = 33 | | | |
| Fig. 5b | Synapsin+/EGFP- | SYN | 83.2 ± 1.06 n = 9 | Interaction F2, 51 = 237,8 ****p<0.0001 | SYN - DA-FASS | Two-way ANOVA |
| | | DA-FASS | 33.78 ± 2.38 n = 10 | Condition F1, 51 = 0,01020 p = 0.9199 | EGFP-/Synapsin+ ****p<0.0001 | |
| | | | | Immunolabelling F2, 51 = 237,5 ****p<0.0001 | EGFP+/Synapsin+ ****p<0.0001 | |
| | EGFP+/Synapsin+ | SYN | 6.62 ± 1.15 n = 9 | | EGFP+/Synapsin- ***p = 0.0006 | |
| | | DA-FASS | 44.88 ± 3.31 n = 10 | | | |
| | | | | | | |
| | EGFP+/Synapsin- | SYN | 9.64 ± 1.43 n = 9 | | | |
| | | DA-FASS | 21.31 ± 1.6 n = 10 | | | |
| Fig. 5d | EGFP-/VGLUT1+ | SYN | 76.23 ± 1.62 n = 12 | Interaction F2, 63 = 91,49 ****p<0.0001 | SYN - DA-FASS | Two-way ANOVA |
| | | DA-FASS | 30.93 ± 4.53 n = 11 | Condition F1, 63 = 0,002 p = 0.9695 | EGFP-/VGLUT1+ ****p<0.0001 | |
| | | | | Immunolabelling F2, 63 = 92,06 ****p<0.0001 | EGFP+/VGLUT1+ **p = 0.0022 | |
| | EGFP+/VGLUT1+ | SYN | 5.92 ± 1.39 n = 12 | | EGFP+/VGLUT1- ****p<0.0001 | |
| | | DA-FASS | 20.78 ± 2.68 n = 11 | | | |
| | | | | | | |
| | EGFP+/VGLUT1- | SYN | 17.8 ± 1.68 n = 12 | | | |
| | | DA-FASS | 48.52 ± 4.61 n = 11 | | | |
| Fig. 5f | EGFP-/VIAAT+ | SYN | 68.19 ± 3.24 n = 9 | Interaction F2, 78 = 54,90 ****p<0.0001 | SYN - DA-FASS | Two-way ANOVA |
| | | DA-FASS | 25.93 ± 1.9 n = 19 | Condition F1, 78 = 0,04 p = 0.8438 | EGFP-/VIAAT+ ****p<0.0001 | |
| | | | | Immunolabelling F2, 78 = 55,34 ****p<0.0001 | EGFP+/VIAAT+ *p = 0.0158 | |
| | EGFP+/VIAAT+ | SYN | 4.11 ± 0.92 n = 9 | | EGFP+/VIAAT- ****p<0.0001 | |
| | | DA-FASS | 18.76 ± 3.22 n = 19 | | | |
| | | | | | | |
| | EGFP+/VIAAT- | SYN | 25.96 ± 2.72 n = 9 | | | |
| | | DA-FASS | 55.32 ± 4.28 n = 19 | | | |

| | | | | | | |
|---|---|---|---|---|---|---|
| Fig. 5h | Th- /VAChT + | SYN | 62.5 ± 2.08 n = 30 | Interaction F2, 144 = 180,3 ****p<0.0001 | SYN - DA-FASS | Two-way ANOVA |
| | | DA-FASS | 28.01 ± 1.34 n = 20 | Condition F1, 144 = 0,01571 p = 0.9004 | Th-/VAChT+ ****p<0.0001 | |
| | | | | Immunolabelling F2, 144 = 412,2 ****p<0.0001 | Th+/VAChT+ **p = 0.0011 | |
| | Th+/VAChT+ | SYN | 1.68 ± 0.37 n = 30 | | Th+/VAChT- ****p<0.0001 | |
| | | DA-FASS | 10.19 ± 0.65 n = 20 | | | |
| | | | | | | |
| | Th+/VAChT- | SYN | 35.23 ± 2.18 n = 30 | | | |
| | | DA-FASS | 61.71 ± 1.19 n = 20 | | | |
| | | | | | | |
| Fig. 6a | Th | DA-FASS | 0.1742 ± 0.003 n = 1175 | ****p<0.0001 | Th - Labelling | Kruskal-Wallis |
| | Syncam2 | DA-FASS | 0.2852 ± 0.006 n = 949 | | Th vs. Syncam2 ****p<0.0001 | |
| | D2R | DA-FASS | 0.2982 ± 0.015 n = 181 | | Th vs. D2R ****p<0.0001 | |
| | VAChT | DA-FASS | 0.3182 ± 0.017 n = 151 | | Th vs. VAChT ****p<0.0001 | |
| | VGLUT 1 | DA-FASS | 0.3813 ± 0.017 n = 193 | | Th vs. VGLUT1 ****p<0.0001 | |
| | VIAAT | DA-FASS | 0.3982 ± 0.015 n = 362 | | Th vs. VIAAT ****p<0.0001 | |
| | VGLUT 2 | DA-FASS | 0.4077 ± 0.019 n = 150 | | Th vs. VGLUT2 ****p<0.0001 | |
| | Stx4 | DA-FASS | 0.4965 ± 0.009 n = 880 | | Th vs. Stx4 ****p<0.0001 | |
| | D1R | DA-FASS | 0.5126 ± 0.022 n = 246 | | Th vs. D1R ****p<0.0001 | |
| | | | | | | |
| | | | | | | |
| | | | | | | |
| Fig. 7b | VGLUT 1venus- /Th+ | SYN | 55.06 ± 3.19 n = 19 | Interaction F2,111 = 47,14 ****p<0.0001 | SYN - VGLUT1-FASS | Two-way ANOVA |
| | | VGLUT1-FASS | 22.03 ± 1.55 n = 20 | Condition F1, 111 = 0,002573 p = 0.9596 | VGLUT1venus- /Th+ ****p<0.0001 | |
| | | | | Immunolabelling F2, 111 = 15,54 ****p<0.0001 | VGLUT1venus+ /Th+ ***p = 0.0006 | |
| | VGLUT 1venus+ /Th+ | SYN | 15.46 ± 2.6 n = 19 | | VGLUT1venus+ /Th- ***p = 0.0002 | |
| | | VGLUT1-FASS | 31.5 ± 4.02 n = 20 | | | |
| | | | | | | |
| | VGLUT 1venus+ /Th- | SYN | 28.14 ± 2.6 n = 19 | | | |
| | | VGLUT1-FASS | 45.5 ± 3.16 n = 20 | | | |
| Fig. 7c | Th | SYN | 437200 ± 79456.71 n = 3 | | p = 0.5839 | Unpaired t-test |

| | | | | | | |
|---|---|---|---|---|---|---|
| | | VGLUT1-FASS | 357648 ± 107551.79 n = 3 | | | |
| | | | | | | |
| | VGLUT 1venus | SYN | 391944.33 ± 60156.53 n = 3 | | *p = 0.0431 | |
| | | VGLUT1-FASS | 670425.33 ± 73824.66 n = 3 | | | |
| Fig. 7d | VGLUT1venus+ | | | | | |
| | | Th- | 143489 ± 2037 n = 3609 | ****p<0.0001 | | Mann Whitney |
| | | Th+ | 182796 ± 4513 n = 1206 | ****p<0.0001 | | Kolmogorov -Smirnov |
| | | | | | | |
| | Bassoon+ | | | | | |
| | | Th- | 143489 ± 2037 n = 3609 | ****p<0.0001 | | Mann Whitney |
| | | Th+ | 182796 ± 4513 n = 1206 | ****p<0.0001 | | Kolmogorov -Smirnov |
| Fig. 7e | | | | | | |
| | Th+/VG LUT1+ | Th_Bassoo n+ | 39,67 ± 4,055 n = 84 | | | descriptive |
| | | Th_Bassoo n- | 60,33 ± 4,055 n = 82 | | | descriptive |
| | | | | | | |
| | Bassoon + | Th_Bassoo n+ | 65498.8 ± 6546 n = 84 | | | |
| | | VGLUT1v enus_Bass oon+ | 255181.0 ± 24922 n = 82 | ****p<0.0001 | | Mann Whitney |
| Fig. 7f | | | | | | |
| | Homer+ | | | | | |
| | | Th- | 55356 ± 1052 n = 1877 | ****p<0.0001 | | Mann Whitney |
| | | Th+ | 64756 ± 1385 n = 1536 | ****p<0.0001 | | Kolmogorov -Smirnov |
| Fig. 7g | | | | | | |
| | PSD95+ | | | | | |
| | | Th- | 21758 ± 471.1 n = 2232 | ****p<0.0001 | | Mann Whitney |
| | | Th+ | 20149 ± 580.9 n = 1533 | ***p = 0.0004 | | Kolmogorov -Smirnov |
| | | | | | | |
| Fig. 7h | Synaptopodin1+ | | | | | |
| | | Th- | 213786 ± 6158 n = 1528 | **p = 0.0015 | | Mann Whitney |
| | | Th+ | 382663 ± 36567 n = 725 | *p = 0.0272 | | Kolmogorov -Smirnov |
| Fig. 7i | VGLUT 1venus+/ Synaptop odin1- | Th- | 68.71 ± 1.17 n = 81 | Interaction F1, 320 = 95,48 ****p<0.0001 | Th- - Th+ | Two-way ANOVA |
| | | Th+ | 54.63 ± 1.7 n = 81 | Condition F1, 320 = 0,1688 p = 0.6815 | VGLUT1+ ****p<0.0001 | |
| | | | | Immunolabelling F1, 320 = 326,0 ****p<0.0001 | VGLUT1+ Synaptopodin1+ ****p<0.0001 | |
| | VGLUT 1venus+/ | Th- | 30.22 ± 0.92 n = 81 | | | |

| | | | | | | |
|---|---|---|---|---|---|---|
| | Synaptopodin1+ | | | | | |
| | | Th+ | 43.17 ± 1.6 n = 81 | | | |
| | | | | | | |
| Supplementary Fig.4 | EGFP-/VGLUT2+ | SYN | 44.55 ± 4.57 n = 6 | Interaction F2, 54 = 80,90 ****p<0.0001 | Singlets - FASS | Two-way ANOVA |
| | | DA-FASS | 8.57 ± 1.53 n = 14 | Condition F1, 54 = 0,0001 p = 0.9912 | VGLUT1+ ****p<0.0001 | |
| | | | | Immunolabelling F2, 54 = 293,7 ****p<0.0001 | ++ *p = 0.0332 | |
| | EGFP+/VGLUT2+ | SYN | 1.79 ± 1.14 n = 6 | | EGFP+ ****p<0.0001 | |
| | | DA-FASS | 11.22 ± 1.38 n = 14 | | | |
| | | | | | | |
| | EGFP+/VGLUT2- | SYN | 53.65 ± 4.29 n = 6 | | | |
| | | DA-FASS | 80.13 ± 2.27 n = 14 | | | |
| Supplementary Fig.5e | All Singlets | SYN | 81.68 ± 0.7955 N = 10 | | *p = 0.0198 | Unpaired t test |
| | | VGLUT1-FASS | 86.05 ± 1.468 N = 11 | | | |
| | | | | | | |
| | SYN | VENUS - Singlets | 52.71 ± 4.38 N = 10 | Interaction F1, 38 = 72.11 ****p<0.0001 | SYN - VGLUT1-FASS | Two-way ANOVA |
| | | VENUS + Singlets | 18.48 ± 3.08 N = 10 | Gating F1, 38 = 0.6683 p = 0.4187 | VENUS - Singlets ****p<0.0001 | |
| | VGLUT1-FASS | VENUS - Singlets | 23.85 ± 3.053 N = 11 | Condition F1, 38 = 0.4118 p = 0.5249 | VENUS + Singlets ****p<0.0001 | |
| | | VENUS + Singlets | 52,06 ± 4,02 N = 11 | | | |
| Supplementary Fig.6a | | | | | | |
| | Bassoon | VGLUT1+ | 93,37 ± 0,40 n = 4456 | | | |
| | | VGLUT1-/Th+ | 19,57 ± 3,73 n = 45 | | | descriptive |
| | | | | | | |
| | | | | | | |
| Supplementary Fig.6b | Th+/Synaptopodin- | SYN | 36.51 ± 1.49 n = 49 | Interaction F2, 255 = 251,5 ****p<0.0001 | Singlets - FASS | Two-way ANOVA |
| | | DA-FASS | 66.65 ± 0.95 n = 38 | Condition F1, 255 = 7,774e-006 p = 0.9978 | Th+/Synaptopodin- ****p<0.0001 | |
| | | | | Immunolabelling F2, 255 = 297,4 ****p<0.0001 | Th+/Synaptopodin+ p = 0.9576 | |
| | Th+/Synaptopodin+ | SYN | 18.59 ± 1.51 n = 49 | | Th-/Synaptopodin+ ****p<0.0001 | |
| | | DA-FASS | 19.47 ± 0.84 n = 38 | | | |
| | | | | | | |

| | | | | | | |
|---|---|---|---|---|---|---|
| | Th-/Synaptopodin+ | SYN | 44.91 ± 1.72 n = 49 | | | |
| | | DA-FASS | 13.9 ± 0.58 n = 38 | | | |
| | | | | | | |
| Supplementary Fig.6c | Th | RIM1+ | 69,67 ± 4,3 n = 73 | | | |
| | | RIM1- | 30,33 ± 4,3 n = 73 | | | descriptive |
| | | | | | | |
| | RIM1+ | Th | 31886 ± 2739 n = 73 | | | |
| | | VGLUT1venus | 255181.0 ± 24922 n = 74 | ***p = 0.0009 | | Mann Whitney |
| | | | | | | |

**Supplementary Table 3: Proteomics Dataset**

| Accession | Coverage [%] | # Unique Peptides | Score Sequest HT: Sequest HT | Entrez Gene ID | Gene Symbol | # Razor Peptides | Abundance Ratio: (DA-FASS) / (SYN) | Abundance Ratio Adj. P-Value: (DA-FASS) / (SYN) |
|---|---|---|---|---|---|---|---|---|
| P16546 | 65 | 195 | 5133,76 | | Sptan1 | 0 | 1,22 | 6,95E-01 |
| Q62261 | 60 | 150 | 2762,32 | 20742 | Sptbn1 | 7 | 1,07 | 9,67E-01 |
| Q9JHU4 | 42 | 196 | 1648,46 | 13424 | Dync1h1 | 0 | 1,25 | 6,06E-01 |
| Q68FD5 | 51 | 91 | 4778,24 | 67300 | Cltc | 0 | 1,14 | 8,70E-01 |
| K2C1_HUMAN | 70 | 44 | 11983,21 | | | 12 | 1,26 | 5,95E-01 |
| Q9QXS1 | 43 | 197 | 1059 | 18810 | Plec | 3 | 1,15 | 8,37E-01 |
| Q6PIC6 | 56 | 39 | 4985,81 | 232975 | Atp1a3 | 3 | 1,10 | 9,34E-01 |
| K22E_HUMAN | 81 | 51 | 8081,59 | | | 14 | 1,34 | 3,61E-01 |
| Q61879 | 53 | 101 | 1111,52 | 77579 | Myh10 | 19 | 1,06 | 9,74E-01 |
| Q8VDN2 | 51 | 37 | 3378,23 | 11928 | Atp1a1 | 0 | 0,98 | 9,81E-01 |
| Q6PIE5 | 54 | 39 | 3610,82 | 98660 | Atp1a2 | 27 | 1,01 | 1,00E+00 |
| G5E829 | 44 | 30 | 2356,23 | 67972 | Atp2b1 | 5 | 0,93 | 9,36E-01 |
| Q9QYX7 | 32 | 123 | 662,32 | 26875 | Pclo | 4 | 0,98 | 9,79E-01 |
| Q9R0K7 | 46 | 36 | 2109,59 | 11941 | Atp2b2 | 24 | 1,02 | 9,90E-01 |
| O88737 | 39 | 101 | 978,92 | 12217 | Bsn | 1 | 0,90 | 8,85E-01 |
| P39053 | 61 | 49 | 2052,86 | 13429 | Dnm1 | 16 | 1,00 | 9,97E-01 |
| Q8C8R3 | 35 | 96 | 1041,55 | 109676 | Ank2 | 13 | 1,31 | 4,46E-01 |
| O88935 | 58 | 27 | 1933,82 | 20964 | Syn1 | 6 | 1,03 | 9,85E-01 |
| K1C10_HUMAN | 60 | 26 | 8256,42 | | | 20 | 1,27 | 5,46E-01 |
| E9Q557 | 30 | 112 | 1229,56 | 109620 | Dsp | 0 | 1,03 | 9,86E-01 |
| P63017 | 64 | 29 | 2583,46 | 15481 | Hspa8 | 14 | 1,09 | 9,42E-01 |
| P56480 | 48 | 21 | 3232,08 | 11947 | Atp5f1b | 0 | 0,73 | 2,60E-01 |
| O08599 | 64 | 45 | 1984,97 | 20910 | Stxbp1 | 1 | 0,93 | 9,56E-01 |
| Q7TMM9 | 62 | 2 | 4324,27 | 22151 | Tubb2a | 8 | 1,11 | 9,21E-01 |
| Q99104 | 41 | 85 | 1194,19 | 17918 | Myo5a | 0 | 1,16 | 8,10E-01 |
| O08553 | 60 | 26 | 2077,38 | 12934 | Dpysl2 | 5 | 0,98 | 9,77E-01 |
| Q9CWF2 | 62 | 2 | 4244,39 | 73710 | Tubb2b | 0 | 1,09 | 9,43E-01 |
| K1C9_HUMAN | 76 | 38 | 5136,35 | | | 0 | 0,81 | 6,07E-01 |
| Q8BPN8 | 31 | 80 | 584,48 | 235380 | Dmxl2 | 2 | 1,18 | 7,79E-01 |
| P60710 | 74 | 2 | 3031,11 | 11461 | Actb | 36 | 0,98 | 9,85E-01 |
| Q9D6F9 | 62 | 8 | 3899,35 | 22153 | Tubb4a | 0 | 1,14 | 8,70E-01 |
| Q64332 | 42 | 18 | 1553,11 | 20965 | Syn2 | 1 | 1,03 | 9,85E-01 |
| P99024 | 61 | 4 | 4269,14 | 22154 | Tubb5 | 2 | 1,19 | 7,50E-01 |
| P50516 | 55 | 34 | 1557,11 | 11964 | Atp6v1a | 0 | 1,16 | 8,10E-01 |
| Q8CAQ8 | 58 | 45 | 1065,87 | 76614 | Immt | 0 | 0,97 | 9,76E-01 |
| Q9DBG3 | 47 | 23 | 1220,75 | 71770 | Ap2b1 | 29 | 1,09 | 9,44E-01 |
| Q9ERD7 | 54 | 10 | 3304,9 | 22152 | Tubb3 | 1 | 1,62 | 2,37E-02 |
| P14873 | 36 | 68 | 554,4 | 17755 | Map1b | 1 | 1,05 | 9,76E-01 |

| | | | | | | | | |
|---|---|---|---|---|---|---|---|---|
| P16858 | 56 | 21 | 1474,42 | 14433 | Gapdh | 0 | 0,94 | 9,60E-01 |
| P46460 | 54 | 52 | 1764,79 | 18195 | Nsf | 0 | 1,19 | 7,50E-01 |
| Q99KI0 | 51 | 39 | 1535,43 | 11429 | Aco2 | 0 | 0,69 | 1,22E-01 |
| P13595 | 43 | 37 | 1553,74 | 17967 | Ncam1 | 0 | 1,07 | 9,69E-01 |
| P17426 | 49 | 35 | 1372,25 | 11771 | Ap2a1 | 13 | 1,14 | 8,53E-01 |
| P17182 | 62 | 28 | 1288,7 | 13806; 433182 | Eno1 | 2 | 0,95 | 9,74E-01 |
| Q9Z1G4 | 35 | 30 | 1264,68 | 11975 | Atp6v0a1 | 0 | 1,02 | 9,96E-01 |
| Q80TJ1 | 45 | 40 | 785,32 | 27062 | Cadps | 8 | 1,06 | 9,74E-01 |
| Q7TSJ2 | 67 | 53 | 1015,41 | 17760 | Map6 | 0 | 1,24 | 6,37E-01 |
| P17710 | 43 | 43 | 1363,22 | 15275 | Hk1 | 6 | 1,05 | 9,76E-01 |
| Q4ACU6 | 48 | 65 | 855,92 | 58234 | Shank3 | 2 | 1,20 | 7,46E-01 |
| Q62108 | 40 | 32 | 1028,69 | 13385 | Dlg4 | 2 | 0,97 | 9,76E-01 |
| Q91VD9 | 53 | 37 | 1182,94 | 227197 | Ndufs1 | 0 | 0,80 | 5,69E-01 |
| P68369 | 59 | 9 | 3054,9 | 22142 | Tuba1a | 23 | 0,96 | 9,74E-01 |
| P12960 | 43 | 39 | 964,72 | 12805 | Cntn1 | 0 | 1,13 | 8,86E-01 |
| Q7TPR4 | 54 | 24 | 711,66 | 109711 | Actn1 | 4 | 1,15 | 8,41E-01 |
| P17427 | 45 | 29 | 1191,78 | 11772 | Ap2a2 | 0 | 1,10 | 9,34E-01 |
| P52480 | 63 | 32 | 1241,64 | 18746 | Pkm | 0 | 0,91 | 9,19E-01 |
| P05064 | 70 | 27 | 1254,57 | 11674 | Aldoa | 3 | 1,32 | 4,17E-01 |
| P68368 | 53 | 8 | 2672,45 | 22145 | Tuba4a | 0 | 0,72 | 1,98E-01 |
| P60879 | 76 | 21 | 1318,03 | 20614 | Snap25 | 2 | 1,19 | 7,51E-01 |
| P19096 | 27 | 61 | 561,73 | 14104 | Fasn | 0 | 1,15 | 8,42E-01 |
| P58281 | 50 | 47 | 848,68 | 74143 | Opa1 | 0 | 0,69 | 1,40E-01 |
| Q9WV92 | 56 | 42 | 688,09 | 13823 | Epb41l3 | 4 | 1,02 | 9,94E-01 |
| P07901 | 45 | 25 | 1185,58 | 15519 | Hsp90aa1 | 0 | 1,09 | 9,42E-01 |
| P11499 | 47 | 25 | 1124,89 | 15516 | Hsp90ab1 | 19 | 1,04 | 9,77E-01 |
| P38647 | 51 | 34 | 756,38 | 15526 | Hspa9 | 0 | 0,84 | 7,39E-01 |
| Q61316 | 59 | 39 | 702,35 | | Hspa4 | 6 | 1,08 | 9,56E-01 |
| P61264 | 56 | 20 | 1178,54 | 56216 | Stx1b | 0 | 0,98 | 9,79E-01 |
| P05214 | 46 | 6 | 2715,95 | 22144; 22147 | Tuba3a; Tuba3b | 0 | 1,08 | 9,56E-01 |
| Q03265 | 56 | 33 | 1879,62 | 11946 | Atp5f1a | 0 | 0,93 | 9,41E-01 |
| Q02053 | 34 | 33 | 729,61 | 22201 | Uba1 | 0 | 1,15 | 8,37E-01 |
| Q6Q477 | 27 | 18 | 1133,86 | 381290 | Atp2b4 | 0 | 0,95 | 9,64E-01 |
| P06837 | 85 | 21 | 814,43 | 14432 | Gap43 | 0 | 1,22 | 6,82E-01 |
| P46096 | 52 | 19 | 1283,36 | 20979 | Syt1 | 13 | 1,14 | 8,66E-01 |
| P63038 | 53 | 35 | 1107,96 | 15510 | Hspd1 | 0 | 1,04 | 9,76E-01 |
| Q4KMM3 | 56 | 42 | 526,02 | 170719 | Oxr1 | 0 | 0,83 | 6,74E-01 |
| Q02257 | 48 | 28 | 1188,43 | 16480 | Jup | 7 | 1,02 | 9,94E-01 |
| Q04447 | 55 | 19 | 1249,15 | 12709 | Ckb | 0 | 0,85 | 7,49E-01 |
| Q920I9 | 39 | 52 | 692,76 | 104082 | Wdr7 | 0 | 1,10 | 9,23E-01 |
| Q922U2 | 36 | 14 | 3490,83 | 110308 | Krt5 | 9 | 0,97 | 9,76E-01 |
| P62259 | 78 | 22 | 1216,67 | 22627 | Ywhae | 3 | 0,86 | 7,86E-01 |
| O55143 | 39 | 41 | 973,84 | 11938 | Atp2a2 | 0 | 0,98 | 9,80E-01 |
| Q61781 | 43 | 3 | 2944,49 | 16664 | Krt14 | 10 | 1,15 | 8,42E-01 |
| Q61644 | 73 | 36 | 774,14 | 23969 | Pacsin1 | 0 | 0,93 | 9,42E-01 |
| Q8CHC4 | 39 | 51 | 754,53 | | Synj1 | 0 | 1,17 | 8,09E-01 |

| | | | | | | | | |
|---|---|---|---|---|---|---|---|---|
| O35643 | 41 | 19 | 865,02 | 11764 | Ap1b1 | 0 | 1,02 | 9,94E-01 |
| Q71LX4 | 33 | 54 | 267,65 | 70549 | Tln2 | 8 | 1,11 | 9,19E-01 |
| Q76MZ3 | 48 | 24 | 725,24 | 51792 | Ppp2r1a | 0 | 1,03 | 9,83E-01 |
| P24549 | 48 | 19 | 678,88 | 11668 | Aldh1a1 | 12 | 3,45 | 6,00E-14 |
| P28652 | 49 | 15 | 1057,84 | 12323 | Camk2b | 9 | 1,02 | 9,91E-01 |
| P57780 | 53 | 27 | 555,74 | 60595 | Actn4 | 18 | 0,91 | 9,12E-01 |
| Q91XM9 | 46 | 31 | 610,04 | 23859 | Dlg2 | 7 | 0,95 | 9,70E-01 |
| Q8VDD5 | 28 | 44 | 325,18 | 17886 | Myh9 | 1 | 0,86 | 7,77E-01 |
| P62814 | 56 | 27 | 1004,09 | 11966 | Atp6v1b2 | 0 | 1,18 | 7,79E-01 |
| P50396 | 66 | 20 | 679,87 | 14567 | Gdi1 | 9 | 0,95 | 9,74E-01 |
| Q62420 | 48 | 13 | 811,31 | 20404 | Sh3gl2 | 7 | 1,04 | 9,76E-01 |
| Q64521 | 47 | 36 | 664,19 | 14571 | Gpd2 | 0 | 0,90 | 8,91E-01 |
| P26443 | 54 | 30 | 697,53 | 14661 | Glud1 | 0 | 0,78 | 4,92E-01 |
| ALBU_BOVIN | 63 | 33 | 913,64 | 280717 | ALB | 7 | 0,56 | 2,12E-03 |
| Q9QYR6 | 24 | 52 | 320,53 | 17754 | Map1a | 0 | 1,24 | 6,48E-01 |
| P23819 | 37 | 29 | 633,93 | | Gria2 | 5 | 1,04 | 9,76E-01 |
| P47857 | 44 | 24 | 677,38 | 18642 | Pfkm | 3 | 1,06 | 9,76E-01 |
| O35526 | 48 | 21 | 859,23 | 20907 | Stx1a | 1 | 1,03 | 9,83E-01 |
| P18872 | 52 | 16 | 1511,41 | 14681 | Gnao1 | 2 | 0,97 | 9,76E-01 |
| Q8QZT1 | 54 | 23 | 805,99 | 110446 | Acat1 | 0 | 0,83 | 6,98E-01 |
| Q6IFX2 | 36 | 6 | 1947,7 | 68239 | Krt42 | 1 | 0,99 | 9,87E-01 |
| Q9CZW5 | 48 | 29 | 673,1 | 28185 | Tomm70 | 0 | 0,65 | 5,06E-02 |
| P50446 | 38 | 3 | 1814,27 | 16687 | Krt6a | 21 | 0,92 | 9,23E-01 |
| P17183 | 55 | 19 | 683,06 | 13807 | Eno2 | 0 | 0,75 | 3,18E-01 |
| D3Z7P3 | 45 | 26 | 532,29 | 14660 | Gls | 0 | 0,91 | 9,02E-01 |
| P60469 | 39 | 43 | 425,79 | 76787 | Ppfia3 | 3 | 1,08 | 9,56E-01 |
| Q01853 | 51 | 35 | 531,46 | 269523 | Vcp | 0 | 1,13 | 8,86E-01 |
| Q9QWL7 | 51 | 10 | 2258,36 | 16667 | Krt17 | 27 | 1,18 | 7,74E-01 |
| P28663 | 68 | 18 | 632,17 | 17957 | Napb | 5 | 1,04 | 9,78E-01 |
| Q6ZQ38 | 34 | 41 | 611,6 | 71902 | Cand1 | 2 | 1,03 | 9,82E-01 |
| Q60597 | 40 | 39 | 526,18 | 18293 | Ogdh | 0 | 0,80 | 5,86E-01 |
| Q91V14 | 24 | 25 | 819,22 | 57138 | Slc12a5 | 5 | 0,98 | 9,85E-01 |
| Q9QWI6 | 41 | 45 | 418,52 | 56013 | Srcin1 | 0 | 1,12 | 8,94E-01 |
| Q9JI91 | 49 | 27 | 525,03 | 11472 | Actn2 | 0 | 1,20 | 7,33E-01 |
| Q91V92 | 44 | 43 | 539,17 | 104112 | Acly | 0 | 1,04 | 9,78E-01 |
| Q9DBJ1 | 57 | 11 | 748,51 | 18648 | Pgam1 | 7 | 1,11 | 9,17E-01 |
| O55131 | 47 | 24 | 747,42 | 235072 | Septin7 | 1 | 1,10 | 9,34E-01 |
| P20029 | 50 | 32 | 740,25 | 14828 | Hspa5 | 0 | 0,97 | 9,77E-01 |
| P56564 | 22 | 11 | 514,62 | 20512 | Slc1a3 | 2 | 0,87 | 8,12E-01 |
| P06745 | 45 | 27 | 896,42 | 14751 | Gpi | 0 | 1,10 | 9,34E-01 |
| Q9Z2H5 | 44 | 30 | 469,07 | 13821 | Epb41l1 | 0 | 1,29 | 5,04E-01 |
| Q810U3 | 31 | 37 | 637,85 | 269116 | Nfasc | 0 | 1,24 | 6,42E-01 |
| Q5SQX6 | 34 | 23 | 607,36 | 76884 | Cyfip2 | 22 | 1,00 | 9,99E-01 |
| Q9DB77 | 47 | 20 | 881,71 | 67003 | Uqcrc2 | 0 | 0,74 | 3,11E-01 |
| P63101 | 79 | 18 | 1055 | 22631 | Ywhaz | 6 | 0,94 | 9,64E-01 |
| Q8K2B3 | 48 | 28 | 587,19 | 66945 | Sdha | 0 | 0,79 | 4,99E-01 |

| | | | | | | | | |
|---|---|---|---|---|---|---|---|---|
| Q91XV3 | 85 | 16 | 546,47 | 70350 | Basp1 | 0 | 0,84 | 7,09E-01 |
| P05063 | 60 | 20 | 600,25 | 11676 | Aldoc | 0 | 0,88 | 8,41E-01 |
| Q922S4 | 38 | 33 | 653,47 | 207728 | Pde2a | 0 | 0,86 | 7,86E-01 |
| Q7TQF7 | 46 | 27 | 696,32 | 218038 | Amph | 2 | 1,18 | 7,79E-01 |
| P09411 | 74 | 21 | 636,16 | 18655 | Pgk1 | 7 | 1,11 | 9,16E-01 |
| Q3UHJ0 | 46 | 28 | 377,48 | 269774 | Aak1 | 0 | 1,09 | 9,42E-01 |
| Q9JME5 | 39 | 40 | 410,01 | 11775 | Ap3b2 | 0 | 1,09 | 9,39E-01 |
| Q9WUA3 | 37 | 28 | 452,23 | 56421 | Pfkp | 5 | 1,17 | 8,07E-01 |
| Q9R1T4 | 41 | 9 | 719,13 | 56526 | Septin6 | 0 | 1,09 | 9,39E-01 |
| ALDOA_RABIT | 47 | 19 | 828,18 | | | 0 | 1,23 | 6,66E-01 |
| Q8BKX1 | 65 | 30 | 628,91 | 108100 | Baiap2 | 0 | 1,13 | 8,85E-01 |
| Q8CI94 | 45 | 30 | 531,98 | 110078 | Pygb | 8 | 0,92 | 9,34E-01 |
| Q02248 | 43 | 21 | 522,33 | 12387 | Ctnnb1 | 0 | 1,17 | 7,91E-01 |
| P12382 | 35 | 21 | 423,29 | 18641 | Pfkl | 0 | 1,01 | 1,00E+00 |
| Q9Z1B3 | 34 | 39 | 415,85 | 18795 | Plcb1 | 0 | 1,00 | 1,00E+00 |
| Q61301 | 44 | 29 | 362,41 | 12386 | Ctnna2 | 4 | 0,91 | 9,02E-01 |
| P08249 | 69 | 21 | 1072,3 | 17448 | Mdh2 | 0 | 0,79 | 5,33E-01 |
| D3YVF0 | 50 | 23 | 731,38 | 238276 | Akap5 | 0 | 0,78 | 4,66E-01 |
| Q9ES97 | 41 | 29 | 542,49 | 20168 | Rtn3 | 1 | 1,03 | 9,83E-01 |
| P14094 | 38 | 18 | 911,79 | 11931 | Atp1b1 | 0 | 0,98 | 9,83E-01 |
| Q8BH59 | 39 | 26 | 1132,69 | 78830 | Slc25a12 | 6 | 0,78 | 4,69E-01 |
| F6SEU4 | 33 | 35 | 438,55 | 240057 | Syngap1 | 1 | 1,09 | 9,51E-01 |
| P68134 | 50 | 5 | 2049,61 | 11459 | Acta1 | 0 | 0,57 | 3,07E-03 |
| Q9QXZ0 | 10 | 65 | 105,97 | | Macf1 | 1 | 1,21 | 7,50E-01 |
| P31324 | 57 | 17 | 512,32 | 19088 | Prkar2b | 4 | 0,92 | 9,30E-01 |
| P47708 | 46 | 27 | 422,2 | 19894 | Rph3a | 0 | 1,23 | 6,58E-01 |
| Q8BFR5 | 54 | 21 | 570,41 | 233870 | Tufm | 0 | 0,92 | 9,31E-01 |
| Q9Z2Y3 | 76 | 27 | 703,02 | 26556 | Homer1 | 1 | 0,94 | 9,59E-01 |
| P68033 | 45 | 4 | 2037,32 | 11464 | Actc1 | 0 | 0,45 | 3,99E-06 |
| P17156 | 40 | 10 | 995,63 | 15512 | Hspa2 | 2 | 1,12 | 9,15E-01 |
| P16330 | 53 | 30 | 832,02 | 12799 | Cnp | 0 | 0,89 | 8,54E-01 |
| E9Q3L2 | 23 | 50 | 337,62 | 224020 | Pi4ka | 0 | 0,88 | 8,41E-01 |
| P70704 | 33 | 37 | 491,24 | 11980 | Atp8a1 | 0 | 0,94 | 9,56E-01 |
| P43006 | 34 | 19 | 1106,81 | 20511 | Slc1a2 | 0 | 0,91 | 9,16E-01 |
| Q60864 | 57 | 39 | 396,04 | 20867 | Stip1 | 0 | 0,93 | 9,48E-01 |
| P15508 | 29 | 47 | 208,99 | | Sptb | 0 | 1,39 | 2,63E-01 |
| Q8K0T0 | 44 | 20 | 628,57 | 104001 | Rtn1 | 0 | 1,09 | 9,47E-01 |
| P46660 | 58 | 29 | 621,57 | 226180 | Ina | 0 | 1,22 | 6,94E-01 |
| P40142 | 39 | 29 | 554,04 | 21881 | Tkt | 0 | 0,99 | 9,95E-01 |
| P28660 | 34 | 36 | 479,02 | 50884 | Nckap1 | 1 | 0,94 | 9,63E-01 |
| P48722 | 42 | 30 | 415,57 | 18415 | Hspa4l | 0 | 1,17 | 7,98E-01 |
| Q99P72 | 37 | 34 | 482,17 | 68585 | Rtn4 | 0 | 1,16 | 8,09E-01 |
| P11798 | 44 | 19 | 1004,59 | 12322 | Camk2a | 9 | 1,10 | 9,23E-01 |
| Q80TE7 | 36 | 40 | 407,17 | 242274 | Lrrc7 | 3 | 0,86 | 7,89E-01 |
| Q8CA95 | 38 | 32 | 567,47 | 23984 | Pde10a | 0 | 0,88 | 8,53E-01 |
| Q62188 | 46 | 17 | 635,22 | 22240 | Dpysl3 | 1 | 1,15 | 8,35E-01 |

| | | | | | | | | |
|---|---|---|---|---|---|---|---|---|
| P62737 | 42 | 4 | 1789,42 | 11475 | Acta2 | 0 | 0,45 | 3,99E-06 |
| Q9Z0P4 | 58 | 20 | 507,58 | 18483 | Palm | 2 | 0,87 | 8,25E-01 |
| Q9Z1G3 | 65 | 31 | 608,28 | 66335 | Atp6v1c1 | 0 | 1,16 | 8,26E-01 |
| Q8C1B7 | 48 | 13 | 719,3 | 52398 | Septin11 | 18 | 1,10 | 9,34E-01 |
| Q9JIS5 | 25 | 18 | 793,47 | 64051 | Sv2a | 0 | 1,01 | 1,00E+00 |
| Q9Z2Q6 | 43 | 18 | 546,08 | 18951 | Septin5 | 1 | 1,13 | 8,82E-01 |
| P70398 | 21 | 50 | 176,02 | 22284 | Usp9x | 0 | 1,06 | 9,74E-01 |
| Q61699 | 47 | 30 | 370,07 | 15505 | Hsph1 | 0 | 1,11 | 9,16E-01 |
| P84091 | 58 | 25 | 641,11 | 11773 | Ap2m1 | 0 | 1,25 | 6,23E-01 |
| O08788 | 33 | 35 | 249,26 | 13191 | Dctn1 | 0 | 1,32 | 4,56E-01 |
| Q8K596 | 29 | 21 | 626,13 | 110891 | Slc8a2 | 1 | 0,96 | 9,74E-01 |
| P40124 | 57 | 21 | 446,56 | 12331 | Cap1 | 1 | 0,88 | 8,51E-01 |
| Q6P9K9 | 23 | 25 | 356,9 | 18191 | Nrxn3 | 6 | 1,18 | 7,89E-01 |
| O08917 | 57 | 21 | 383,56 | 14251 | Flot1 | 0 | 1,00 | 1,00E+00 |
| P11881 | 19 | 53 | 204,63 | 16438 | Itpr1 | 0 | 1,07 | 9,70E-01 |
| P05201 | 64 | 25 | 763,62 | 14718 | Got1 | 0 | 1,04 | 9,77E-01 |
| P63268 | 39 | 3 | 1786,67 | 11468 | Actg2 | 0 | 0,45 | 3,99E-06 |
| Q05920 | 32 | 32 | 394,15 | | Pc | 0 | 0,73 | 2,61E-01 |
| Q60932 | 59 | 15 | 756,81 | 22333 | Vdac1 | 2 | 0,84 | 7,33E-01 |
| P08553 | 36 | 30 | 525,54 | 18040 | Nefm | 4 | 1,14 | 8,66E-01 |
| Q9Z2K1 | 24 | 8 | 2452,76 | 16666 | Krt16 | 0 | 0,86 | 7,90E-01 |
| Q9CZ13 | 44 | 18 | 646,85 | 22273 | Uqcrc1 | 1 | 0,83 | 6,74E-01 |
| O08532 | 26 | 27 | 334,25 | 12293 | Cacna2d1 | 1 | 0,86 | 7,89E-01 |
| P05202 | 47 | 23 | 805,54 | 14719 | Got2 | 0 | 0,99 | 9,85E-01 |
| P56399 | 34 | 25 | 488,45 | 22225 | Usp5 | 0 | 1,09 | 9,42E-01 |
| Q61548 | 24 | 20 | 733,3 | 20616 | Snap91 | 3 | 1,10 | 9,34E-01 |
| Q8VD37 | 47 | 28 | 330,25 | 73094 | Sgip1 | 0 | 1,18 | 7,77E-01 |
| Q91V41 | 78 | 16 | 571,04 | 68365 | Rab14 | 2 | 1,11 | 9,16E-01 |
| P08113 | 38 | 30 | 359,39 | 22027 | Hsp90b1 | 0 | 0,83 | 6,94E-01 |
| P35564 | 39 | 24 | 405,44 | 12330 | Canx | 0 | 0,81 | 6,23E-01 |
| Q11011 | 35 | 31 | 482,15 | 19155 | Npepps | 0 | 1,08 | 9,60E-01 |
| B1AWN6 | 19 | 21 | 216,93 | 110876 | Scn2a | 20 | 1,09 | 9,36E-01 |
| TRYP_PIG | 50 | 9 | 10937,77 | | | 0 | 1,07 | 9,71E-01 |
| P35486 | 54 | 29 | 531,05 | 18597 | Pdha1 | 1 | 0,86 | 7,90E-01 |
| P50518 | 51 | 19 | 660,91 | 11973 | Atp6v1e1 | 0 | 1,17 | 8,07E-01 |
| O35927 | 31 | 29 | 358,55 | 18163 | Ctnnd2 | 5 | 1,04 | 9,76E-01 |
| Q99KJ8 | 53 | 17 | 352,5 | 69654 | Dctn2 | 0 | 1,09 | 9,44E-01 |
| KRHB6_HUMAN | 53 | 7 | 295,74 | 3892 | KRT86 | 8 | 2,20 | 9,01E-06 |
| DHE3_BOVIN | 36 | 22 | 528,11 | 281785 | GLUD1 | 0 | 0,81 | 5,97E-01 |
| Q8BVE3 | 48 | 22 | 396,68 | 108664 | Atp6v1h | 0 | 1,09 | 9,39E-01 |
| Q80TZ3 | 29 | 26 | 424,74 | 72685 | Dnajc6 | 3 | 1,32 | 4,06E-01 |
| Q8VEM8 | 39 | 16 | 1019,25 | 18674 | Slc25a3 | 0 | 0,85 | 7,41E-01 |
| Q8BMF4 | 29 | 17 | 538,19 | 235339 | Dlat | 0 | 0,73 | 2,39E-01 |
| O88533 | 31 | 14 | 347,65 | 13195 | Ddc | 0 | 4,01 | 1,60E-16 |
| Q61598 | 59 | 15 | 354,8 | 14569 | Gdi2 | 0 | 0,89 | 8,54E-01 |
| Q9D051 | 42 | 14 | 503,14 | 68263 | Pdhb | 0 | 0,87 | 8,09E-01 |

| | | | | | | | | |
|---|---|---|---|---|---|---|---|---|
| Q64487 | 20 | 20 | 324,15 | 19266 | Ptprd | 10 | 1,03 | 9,85E-01 |
| Q91WD5 | 37 | 16 | 480,05 | 226646 | Ndufs2 | 0 | 0,81 | 6,06E-01 |
| P16125 | 43 | 16 | 669,58 | 16832 | Ldhb | 0 | 1,03 | 9,83E-01 |
| Q8K1M6 | 46 | 30 | 354,23 | 74006 | Dnm1l | 0 | 1,23 | 6,61E-01 |
| Q8BW75 | 44 | 18 | 400,14 | 109731 | Maob | 3 | 0,93 | 9,40E-01 |
| P20357 | 25 | 37 | 178,12 | 17756 | Map2 | 0 | 1,08 | 9,70E-01 |
| Q8BFZ3 | 39 | 4 | 875,69 | 238880 | Actbl2 | 0 | 0,71 | 5,00E-01 |
| Q8BZ98 | 30 | 14 | 561,75 | 103967 | Dnm3 | 6 | 1,10 | 9,36E-01 |
| P14824 | 51 | 33 | 286,14 | 11749 | Anxa6 | 0 | 1,05 | 9,76E-01 |
| Q9CQV8 | 59 | 8 | 696,38 | 54401 | Ywhab | 0 | 0,90 | 8,86E-01 |
| P97427 | 35 | 12 | 627,85 | 12933 | Crmp1 | 2 | 1,16 | 8,25E-01 |
| P48962 | 57 | 13 | 987,34 | 11739 | Slc25a4 | 11 | 0,80 | 5,72E-01 |
| Q8K0U4 | 43 | 30 | 391,99 | 73442 | Hspa12a | 0 | 0,92 | 9,33E-01 |
| P97797 | 35 | 13 | 371,19 | 19261 | Sirpa | 0 | 0,90 | 8,93E-01 |
| P17751 | 66 | 17 | 757,85 | 21991 | Tpi1 | 0 | 1,10 | 9,34E-01 |
| Q9Z218 | 32 | 26 | 417,5 | 13483 | Dpp6 | 3 | 1,08 | 9,60E-01 |
| P63318 | 44 | 24 | 372,36 | 18752 | Prkcg | 4 | 0,81 | 6,35E-01 |
| O70161 | 36 | 19 | 208,88 | 18717 | Pip5k1c | 0 | 1,09 | 9,60E-01 |
| E9PUL5 | 53 | 11 | 345,54 | 69017 | Prrt2 | 0 | 0,87 | 8,10E-01 |
| Q6PB66 | 25 | 34 | 183,07 | 72416 | Lrpprc | 0 | 0,79 | 5,35E-01 |
| B0V2N1 | 20 | 21 | 289,01 | 19280 | Ptprs | 0 | 1,11 | 9,34E-01 |
| Q3UHL1 | 49 | 21 | 347,99 | 235604 | Camkv | 0 | 1,18 | 7,86E-01 |
| P07356 | 47 | 20 | 520,58 | 12306 | Anxa2 | 0 | 0,94 | 9,60E-01 |
| P15105 | 48 | 19 | 515,74 | 14645 | Glul | 0 | 0,87 | 8,07E-01 |
| P47757 | 54 | 17 | 344,96 | 12345 | Capzb | 0 | 1,02 | 9,91E-01 |
| P27773 | 43 | 23 | 452,98 | 14827 | Pdia3 | 0 | 0,90 | 8,95E-01 |
| Q9Z2I0 | 34 | 25 | 356,68 | 56384 | Letm1 | 0 | 0,75 | 3,18E-01 |
| O08539 | 39 | 20 | 507,16 | 30948 | Bin1 | 0 | 1,03 | 9,85E-01 |
| O70589 | 31 | 28 | 294,42 | 12361 | Cask | 0 | 1,12 | 8,91E-01 |
| Q9QXS6 | 35 | 23 | 309,32 | 56320 | Dbn1 | 0 | 1,02 | 9,96E-01 |
| Q7TMB8 | 22 | 8 | 397,1 | 20430 | Cyfip1 | 0 | 0,77 | 3,85E-01 |
| Q9DB05 | 61 | 12 | 352,88 | 108124 | Napa | 0 | 1,03 | 9,93E-01 |
| P06151 | 44 | 17 | 531,85 | 16828 | Ldha | 1 | 0,92 | 9,34E-01 |
| Q9EQH3 | 32 | 25 | 367,3 | 65114 | Vps35 | 0 | 1,09 | 9,50E-01 |
| Q8CHH9 | 38 | 17 | 424,64 | 20362 | Septin8 | 0 | 0,98 | 9,81E-01 |
| KRHB5_HUMAN | 51 | 6 | 250,66 | 3891 | KRT85 | 27 | 2,27 | 3,20E-06 |
| Q9DCX2 | 54 | 12 | 461,55 | 71679 | Atp5pd | 0 | 0,79 | 5,01E-01 |
| P63044 | 69 | 6 | 464 | 22318 | Vamp2 | 2 | 1,42 | 1,91E-01 |
| Q9R0P9 | 63 | 13 | 538,74 | 22223 | Uchl1 | 0 | 1,28 | 5,43E-01 |
| Q60634 | 54 | 23 | 269,56 | 14252 | Flot2 | 0 | 0,99 | 9,89E-01 |
| Q9WU78 | 39 | 29 | 215,65 | 18571 | Pdcd6ip | 0 | 1,08 | 9,56E-01 |
| P08551 | 37 | 24 | 367,01 | 18039 | Nefl | 0 | 1,06 | 9,74E-01 |
| P68254 | 47 | 11 | 607,63 | 22630 | Ywhaq | 0 | 0,88 | 8,26E-01 |
| Q6PH08 | 35 | 28 | 266,74 | 238988 | Erc2 | 5 | 1,35 | 3,45E-01 |
| KRHB3_HUMAN | 42 | 2 | 277,23 | | | 0 | 0,51 | 1,51E-02 |
| P70175 | 31 | 23 | 221,57 | 53310 | Dlg3 | 0 | 1,06 | 9,76E-01 |

| | | | | | | | | |
|---|---|---|---|---|---|---|---|---|
| P68404 | 36 | 15 | 363,09 | 18751 | Prkcb | 4 | 1,08 | 9,56E-01 |
| P10637 | 32 | 22 | 437,39 | 17762 | Mapt | 0 | 0,98 | 9,85E-01 |
| Q9CS84 | 22 | 24 | 257,11 | 18189 | Nrxn1 | 3 | 1,00 | 9,99E-01 |
| P51881 | 53 | 11 | 846,87 | 11740 | Slc25a5 | 0 | 0,72 | 2,06E-01 |
| Q91V61 | 60 | 16 | 520,12 | 94280 | Sfxn3 | 2 | 0,78 | 4,72E-01 |
| Q99N28 | 42 | 12 | 385,96 | 94332 | Cadm3 | 0 | 0,97 | 9,76E-01 |
| P62874 | 33 | 7 | 756,38 | 14688 | Gnb1 | 6 | 0,77 | 4,48E-01 |
| P61922 | 34 | 16 | 486,71 | 268860 | Abat | 0 | 0,70 | 1,64E-01 |
| Q61490 | 31 | 20 | 345,97 | 11658 | Alcam | 0 | 1,06 | 9,74E-01 |
| Q91ZX7 | 9 | 36 | 112,42 | 16971 | Lrp1 | 2 | 0,89 | 8,57E-01 |
| Q8R0Y6 | 35 | 27 | 210,87 | 107747 | Aldh1l1 | 0 | 0,85 | 7,48E-01 |
| O08749 | 39 | 18 | 618,89 | 13382 | Dld | 0 | 0,88 | 8,33E-01 |
| P60904 | 46 | 10 | 396,22 | 13002 | Dnajc5 | 0 | 1,27 | 5,48E-01 |
| P61982 | 58 | 8 | 668,79 | 22628 | Ywhag | 5 | 1,04 | 9,76E-01 |
| P63328 | 31 | 11 | 671,42 | 19055 | Ppp3ca | 6 | 0,95 | 9,70E-01 |
| Q811D0 | 26 | 14 | 310,56 | 13383 | Dlg1 | 0 | 1,18 | 7,77E-01 |
| Q91ZA3 | 36 | 25 | 239,51 | 110821 | Pcca | 0 | 0,64 | 4,82E-02 |
| P58252 | 31 | 26 | 274,73 | 13629 | Eef2 | 0 | 1,01 | 1,00E+00 |
| Q6ZQ18 | 26 | 19 | 233,58 | 668212 | Efr3b | 1 | 0,92 | 9,24E-01 |
| O35136 | 31 | 23 | 257,97 | 17968 | Ncam2 | 0 | 1,08 | 9,59E-01 |
| O88342 | 37 | 19 | 343,51 | 22388 | Wdr1 | 0 | 1,01 | 1,00E+00 |
| P42932 | 45 | 22 | 393,78 | 12469 | Cct8 | 0 | 1,04 | 9,77E-01 |
| ALBU_HUMAN | 41 | 20 | 505,78 | 213 | ALB | 0 | 0,80 | 5,96E-01 |
| Q8CGY8 | 28 | 28 | 229,93 | 108155 | Ogt | 0 | 1,08 | 9,56E-01 |
| O35098 | 35 | 13 | 216,66 | | Dpysl4 | 0 | 1,31 | 4,42E-01 |
| Q91WC3 | 41 | 27 | 239,22 | 216739 | Acsl6 | 1 | 0,88 | 8,56E-01 |
| Q9D0M3 | 34 | 10 | 468,71 | 66445 | Cyc1 | 0 | 0,86 | 7,90E-01 |
| P08752 | 49 | 8 | 622,09 | 14678 | Gnai2 | 0 | 0,90 | 8,91E-01 |
| P35700 | 68 | 11 | 432,11 | 18477 | Prdx1 | 9 | 1,33 | 3,97E-01 |
| Q80TL4 | 51 | 19 | 285,75 | 230085 | Phf24 | 0 | 1,09 | 9,38E-01 |
| P80318 | 45 | 24 | 212,87 | 12462 | Cct3 | 0 | 1,19 | 7,60E-01 |
| P35802 | 20 | 9 | 467,92 | 234267 | Gpm6a | 0 | 0,89 | 8,85E-01 |
| Q9D0K2 | 31 | 15 | 287,22 | 67041 | Oxct1 | 0 | 0,86 | 7,78E-01 |
| AGG56535.1 | 42 | 12 | 368,16 | | | 0 | 5,12 | 1,60E-16 |
| Q99PU5 | 33 | 19 | 232,58 | 94180 | Acsbg1 | 0 | 0,94 | 9,56E-01 |
| P97807 | 40 | 15 | 351,81 | 14194 | Fh | 0 | 0,82 | 6,57E-01 |
| Q99PT1 | 54 | 9 | 306,55 | 192662 | Arhgdia | 0 | 0,92 | 9,22E-01 |
| Q9Z1S5 | 44 | 16 | 417,01 | 24050 | Septin3 | 0 | 0,92 | 9,34E-01 |
| P24529 | 41 | 19 | 256,87 | 21823 | Th | 0 | 4,52 | 1,60E-16 |
| Q8R1Q8 | 48 | 23 | 250,14 | 235661 | Dync1li1 | 1 | 1,06 | 9,74E-01 |
| Q8BGZ7 | 29 | 5 | 1120,73 | 109052 | Krt75 | 1 | 1,08 | 9,69E-01 |
| Q9EPN1 | 15 | 34 | 104,37 | 26422 | Nbea | 0 | 1,04 | 9,82E-01 |
| P10852 | 30 | 16 | 600,45 | 17254 | Slc3a2 | 0 | 1,05 | 9,76E-01 |
| P63011 | 51 | 6 | 909,09 | 19339 | Rab3a | 1 | 1,09 | 9,48E-01 |
| P23818 | 31 | 23 | 305,7 | 14799 | Gria1 | 2 | 1,11 | 9,22E-01 |
| P70441 | 53 | 17 | 218,16 | 26941 | Slc9a3r1 | 0 | 1,05 | 9,76E-01 |

| | | | | | | | | |
|---|---|---|---|---|---|---|---|---|
| B2RSH2 | 44 | 9 | 629,06 | 14677 | Gnai1 | 6 | 0,81 | 6,36E-01 |
| P70168 | 27 | 19 | 220,2 | 16211 | Kpnb1 | 0 | 1,06 | 9,74E-01 |
| Q8K212 | 37 | 30 | 225,15 | 107975 | Pacs1 | 0 | 1,18 | 7,97E-01 |
| Q03137 | 24 | 17 | 212,74 | 13838 | Epha4 | 2 | 1,14 | 8,85E-01 |
| P84309 | 20 | 21 | 216,43 | 224129 | Adcy5 | 1 | 0,73 | 2,47E-01 |
| KRHB4_HUMAN | 48 | 23 | 417,99 | | | 1 | 1,11 | 9,19E-01 |
| Q8JZQ2 | 29 | 25 | 230,23 | 69597 | Afg3l2 | 0 | 0,76 | 4,02E-01 |
| O70318 | 32 | 25 | 176,97 | 13822 | Epb41l2 | 0 | 0,87 | 8,09E-01 |
| P63085 | 47 | 11 | 389,32 | 26413 | Mapk1 | 8 | 1,16 | 8,10E-01 |
| Q8K021 | 41 | 10 | 345,45 | 107767 | Scamp1 | 0 | 1,22 | 6,94E-01 |
| Q9Z2I9 | 48 | 23 | 317,15 | 20916 | Sucla2 | 0 | 0,89 | 8,53E-01 |
| Q4KUS2 | 19 | 35 | 140,61 | 382018 | Unc13a | 0 | 1,10 | 9,24E-01 |
| Q8BJI1 | 24 | 15 | 290,3 | 229706 | Slc6a17 | 0 | 0,94 | 9,60E-01 |
| P09103 | 35 | 18 | 202,09 | 18453 | P4hb | 0 | 0,93 | 9,36E-01 |
| Q8BLQ9 | 41 | 16 | 281,82 | 239857 | Cadm2 | 0 | 1,28 | 5,42E-01 |
| Q9Z2W9 | 28 | 18 | 259,48 | 53623 | Gria3 | 2 | 0,99 | 9,87E-01 |
| P97300 | 34 | 16 | 462,22 | 20320 | Nptn | 0 | 1,09 | 9,48E-01 |
| TAU_HUMAN | 20 | 16 | 417,45 | | | 0 | 0,98 | 9,78E-01 |
| P02535 | 22 | 3 | 3020,19 | 16661 | Krt10 | 0 | 3,77 | 4,06E-05 |
| Q923T9 | 28 | 6 | 467,57 | 12325 | Camk2g | 0 | 1,00 | 9,91E-01 |
| P80314 | 38 | 19 | 245,98 | 12461 | Cct2 | 0 | 1,21 | 7,25E-01 |
| P68181 | 40 | 7 | 350,82 | 18749 | Prkacb | 13 | 1,03 | 9,87E-01 |
| P53810 | 65 | 17 | 174,71 | 18738 | Pitpna | 3 | 1,11 | 9,19E-01 |
| O54983 | 34 | 11 | 483,93 | 12971 | Crym | 0 | 0,87 | 8,05E-01 |
| P68510 | 58 | 12 | 629,08 | 22629 | Ywhah | 3 | 0,90 | 8,89E-01 |
| Q6NXK7 | 29 | 21 | 349,87 | 269109 | Dpp10 | 0 | 0,89 | 8,57E-01 |
| P14152 | 43 | 14 | 615,45 | 17449 | Mdh1 | 0 | 1,05 | 9,76E-01 |
| P80315 | 45 | 21 | 213,98 | 12464 | Cct4 | 1 | 1,01 | 1,00E+00 |
| P47754 | 67 | 11 | 276,73 | 12343 | Capza2 | 2 | 1,04 | 9,76E-01 |
| Q91YT0 | 36 | 18 | 380,74 | 17995 | Ndufv1 | 0 | 0,80 | 5,47E-01 |
| Q8BYR5 | 21 | 17 | 167,27 | 320405 | Cadps2 | 0 | 1,62 | 2,37E-02 |
| Q8BG39 | 24 | 14 | 418,51 | 64176 | Sv2b | 2 | 0,96 | 9,76E-01 |
| O55106 | 32 | 16 | 197,83 | 268980 | Strn | 2 | 0,78 | 4,82E-01 |
| P17742 | 63 | 11 | 336 | 268373 | Ppia | 0 | 0,87 | 8,26E-01 |
| P48453 | 31 | 9 | 409,29 | 19056 | Ppp3cb | 0 | 0,95 | 9,70E-01 |
| CATA_HUMAN | 33 | 16 | 293,24 | | | 4 | 0,93 | 9,39E-01 |
| P62761 | 70 | 14 | 208,53 | 26950 | Vsnl1 | 3 | 1,20 | 7,33E-01 |
| Q80U63 | 28 | 20 | 193,54 | 170731 | Mfn2 | 0 | 0,88 | 8,43E-01 |
| P62880 | 32 | 3 | 685,85 | 14693 | Gnb2 | 3 | 0,76 | 3,75E-01 |
| Q9D6R2 | 43 | 17 | 566,42 | 67834 | Idh3a | 0 | 0,85 | 7,46E-01 |
| P63005 | 46 | 18 | 257,37 | 18472 | Pafah1b1 | 1 | 1,02 | 9,98E-01 |
| Q9CWZ7 | 62 | 19 | 332,19 | 108123 | Napg | 0 | 1,07 | 9,71E-01 |
| PPIA_HUMAN | 57 | 10 | 312,06 | | | 0 | 0,88 | 8,50E-01 |
| O35945 | 28 | 4 | 243,14 | 26358 | Aldh1a7 | 0 | 4,30 | 6,26E-06 |

| | | | | | | | | |
|---|---|---|---|---|---|---|---|---|
| Q8BX70 | 11 | 35 | 46,46 | 320528 | Vps13c | 0 | 0,96 | 9,74E-01 |
| P53994 | 69 | 8 | 299,03 | 59021 | Rab2a | 8 | 1,10 | 9,25E-01 |
| Q921M7 | 42 | 9 | 211,56 | 223601 | Cyrib | 4 | 1,07 | 9,72E-01 |
| O55042 | 57 | 6 | 426,58 | 20617 | Snca | 4 | 0,91 | 9,09E-01 |
| P05132 | 32 | 6 | 340,62 | 18747 | Prkaca | 0 | 0,97 | 9,76E-01 |
| A2AN08 | 8 | 40 | 50,97 | 69116 | Ubr4 | 0 | 1,22 | 6,92E-01 |
| P42859 | 12 | 33 | 79,6 | | Htt | 0 | 1,19 | 7,60E-01 |
| P11627 | 19 | 21 | 186,89 | | L1cam | 0 | 0,94 | 9,64E-01 |
| Q9R111 | 44 | 22 | 309,64 | 14544 | Gda | 0 | 0,81 | 6,07E-01 |
| P19783 | 60 | 15 | 432,3 | 12857 | Cox4i1 | 0 | 0,81 | 6,28E-01 |
| Q9QYC0 | 28 | 17 | 227,31 | 11518 | Add1 | 1 | 1,17 | 8,05E-01 |
| P47738 | 34 | 15 | 242,75 | 11669 | Aldh2 | 0 | 0,75 | 3,15E-01 |
| E9Q401 | 8 | 31 | 51,6 | 20191 | Ryr2 | 5 | 1,01 | 1,00E+00 |
| Q8VHW2 | 42 | 10 | 180,6 | | Cacng8 | 0 | 0,99 | 9,91E-01 |
| O08709 | 64 | 15 | 265,89 | 11758 | Prdx6 | 0 | 0,82 | 6,58E-01 |
| Q3UV17 | 19 | 7 | 871,98 | 77055 | Krt76 | 2 | 1,29 | 4,83E-01 |
| P61161 | 31 | 12 | 312,46 | 66713 | Actr2 | 0 | 0,96 | 9,74E-01 |
| Q60902 | 28 | 21 | 124,31 | 13859 | Eps15l1 | 0 | 0,90 | 8,81E-01 |
| Q9CZS1 | 36 | 16 | 209,41 | 72535 | Aldh1b1 | 1 | 0,66 | 7,46E-02 |
| O70443 | 52 | 16 | 283,76 | 14687 | Gnaz | 0 | 0,97 | 9,76E-01 |
| Q8BWF0 | 35 | 17 | 302,99 | 214579 | Aldh5a1 | 0 | 0,97 | 9,76E-01 |
| Q3UNH4 | 30 | 23 | 171,81 | 26913 | Gprin1 | 0 | 0,88 | 8,26E-01 |
| Q6PHZ2 | 31 | 8 | 401,24 | 108058 | Camk2d | 0 | 1,29 | 5,02E-01 |
| Q9CQQ7 | 38 | 16 | 353,34 | 11950 | Atp5pb | 0 | 0,82 | 6,58E-01 |
| Q80UG2 | 18 | 20 | 100,5 | 243743 | Plxna4 | 11 | 0,93 | 9,56E-01 |
| Q9WV34 | 45 | 21 | 285,18 | 50997 | Mpp2 | 2 | 0,99 | 9,91E-01 |
| K1H1_HUMAN | 56 | 4 | 580,53 | | | 1 | 1,00 | 9,91E-01 |
| P28738 | 28 | 14 | 146,44 | 16574 | Kif5c | 8 | 1,24 | 6,21E-01 |
| P21279 | 49 | 12 | 288,8 | 14682 | Gnaq | 7 | 0,89 | 8,53E-01 |
| K1HB_HUMAN | 54 | 5 | 549,56 | 3884 | KRT33B | 17 | 1,87 | 9,82E-04 |
| P26645 | 55 | 10 | 191,05 | 17118 | Marcks | 0 | 0,93 | 9,42E-01 |
| Q9CYT6 | 41 | 18 | 309,94 | 67252 | Cap2 | 0 | 0,88 | 8,32E-01 |
| Q6ZPE2 | 20 | 31 | 99,06 | 77980 | Sbf1 | 0 | 0,99 | 9,94E-01 |
| G5E8K5 | 13 | 14 | 150,72 | 11735 | Ank3 | 0 | 0,80 | 6,23E-01 |
| P31648 | 18 | 8 | 243,08 | 232333 | Slc6a1 | 0 | 1,04 | 9,78E-01 |
| Q3TXX4 | 20 | 11 | 435,36 | 72961 | Slc17a7 | 3 | 0,97 | 9,76E-01 |
| Q9D6M3 | 42 | 9 | 420,88 | 68267 | Slc25a22 | 3 | 0,85 | 7,61E-01 |
| P11983 | 39 | 20 | 283,34 | 21454 | Tcp1 | 0 | 1,12 | 8,93E-01 |
| Q9Z0X1 | 29 | 15 | 199,27 | 26926 | Aifm1 | 0 | 0,76 | 3,03E-01 |
| Q9CZC8 | 38 | 14 | 200,36 | 69938 | Scrn1 | 0 | 1,08 | 9,55E-01 |
| Q9QXL2 | 14 | 18 | 189,94 | 16564 | Kif21a | 1 | 1,22 | 7,20E-01 |
| K1HA_HUMAN | 58 | 2 | 224,8 | | | 6 | 1,19 | 7,71E-01 |
| Q60931 | 40 | 10 | 402,3 | 22335 | Vdac3 | 0 | 0,86 | 7,77E-01 |
| P10126 | 30 | 8 | 435,39 | 13627 | Eef1a1 | 9 | 1,05 | 9,76E-01 |
| Q9Z0E0 | 28 | 19 | 318,13 | 26562 | Ncdn | 0 | 0,96 | 9,76E-01 |

| | | | | | | | | |
|---|---|---|---|---|---|---|---|---|
| Q9CR62 | 55 | 16 | 377,57 | 67863 | Slc25a11 | 0 | 0,94 | 9,56E-01 |
| Q80TR1 | 19 | 19 | 221,84 | 330814 | Adgrl1 | 2 | 1,06 | 9,74E-01 |
| P62823 | 48 | 7 | 605,55 | 67295 | Rab3c | 2 | 1,27 | 5,50E-01 |
| Q64727 | 30 | 29 | 130,67 | 22330 | Vcl | 0 | 1,05 | 9,76E-01 |
| Q01065 | 36 | 16 | 203,09 | 18574 | Pde1b | 2 | 0,81 | 6,23E-01 |
| P80316 | 40 | 20 | 180,52 | 12465 | Cct5 | 0 | 1,08 | 9,56E-01 |
| Q91V12 | 30 | 11 | 218,11 | 70025 | Acot7 | 0 | 1,17 | 8,25E-01 |
| P62821 | 68 | 8 | 456,14 | 19324 | Rab1A | 4 | 1,19 | 7,61E-01 |
| Q60598 | 42 | 21 | 186,64 | 13043 | Cttn | 0 | 1,08 | 9,56E-01 |
| Q8BMS1 | 27 | 19 | 222,88 | 97212 | Hadha | 0 | 1,03 | 9,85E-01 |
| P67778 | 56 | 14 | 278,51 | 18673 | Phb | 0 | 0,85 | 7,61E-01 |
| Q62465 | 35 | 11 | 258,46 | 26949 | Vat1 | 0 | 1,65 | 1,58E-02 |
| Q8BUV3 | 35 | 19 | 153,71 | 268566 | Gphn | 0 | 1,16 | 8,45E-01 |
| P10649 | 65 | 16 | 294,11 | 14862 | Gstm1 | 1 | 0,96 | 9,76E-01 |
| O54991 | 18 | 21 | 203,03 | 53321 | Cntnap1 | 0 | 0,96 | 9,74E-01 |
| Q8CC35 | 30 | 17 | 132,01 | | Synpo | 0 | 1,02 | 1,00E+00 |
| Q9EQ20 | 33 | 16 | 177,43 | 104776 | Aldh6a1 | 0 | 0,79 | 5,25E-01 |
| Q8JZW4 | 35 | 19 | 229 | 240058 | Cpne5 | 1 | 1,00 | 9,96E-01 |
| Q3UM45 | 54 | 20 | 204,31 | 66385 | Ppp1r7 | 0 | 0,88 | 8,34E-01 |
| Q9QXY6 | 30 | 13 | 265,7 | 57440 | Ehd3 | 8 | 1,04 | 9,78E-01 |
| P46097 | 30 | 4 | 462,78 | 20980 | Syt2 | 0 | 1,07 | 9,74E-01 |
| Q3UVX5 | 21 | 24 | 247,98 | 108071 | Grm5 | 0 | 0,87 | 8,07E-01 |
| Q61696 | 33 | 9 | 326,29 | 193740 | Hspa1a | 6 | 1,05 | 9,76E-01 |
| P17879 | 33 | 9 | 326,29 | 15511 | Hspa1b | 0 | 1,05 | 9,76E-01 |
| Q80U28 | 19 | 26 | 129,67 | 228355 | Madd | 0 | 1,25 | 6,29E-01 |
| P32037 | 17 | 10 | 247,63 | 20527 | Slc2a3 | 0 | 0,94 | 9,64E-01 |
| Q6URW6 | 15 | 21 | 131,56 | 71960 | Myh14 | 0 | 0,84 | 6,72E-01 |
| Q80UG5 | 34 | 21 | 256,38 | 53860 | Septin9 | 0 | 1,00 | 1,00E+00 |
| P14211 | 37 | 12 | 195,36 | 12317 | Calr | 0 | 0,77 | 4,33E-01 |
| Q8CGK3 | 25 | 22 | 160,37 | 74142 | Lonp1 | 0 | 0,80 | 5,41E-01 |
| Q01097 | 18 | 25 | 169,14 | 14812 | Grin2b | 0 | 1,04 | 9,78E-01 |
| Q60930 | 39 | 10 | 507,97 | 22334 | Vdac2 | 0 | 0,73 | 2,50E-01 |
| P15116 | 24 | 12 | 170,5 | 12558 | Cdh2 | 2 | 0,98 | 9,83E-01 |
| Q99LC5 | 43 | 11 | 134,38 | 110842 | Etfa | 0 | 0,85 | 7,50E-01 |
| O89053 | 37 | 20 | 278,49 | 12721 | Coro1a | 0 | 0,93 | 9,39E-01 |
| O35633 | 25 | 13 | 268,43 | 22348 | Slc32a1 | 0 | 0,86 | 7,82E-01 |
| P28740 | 28 | 21 | 198,79 | 16563 | Kif2a | 0 | 1,35 | 4,04E-01 |
| Q80SW1 | 28 | 6 | 247,89 | 229709 | Ahcyl1 | 13 | 0,95 | 9,74E-01 |
| P23242 | 32 | 9 | 209,89 | 14609 | Gja1 | 0 | 0,98 | 9,83E-01 |
| Q9CZU6 | 33 | 18 | 429,28 | 12974 | Cs | 0 | 0,81 | 6,17E-01 |
| Q9CQA3 | 44 | 15 | 285,89 | 67680 | Sdhb | 0 | 0,84 | 7,38E-01 |
| P14231 | 37 | 10 | 292,24 | 11932 | Atp1b2 | 0 | 0,96 | 9,74E-01 |
| P61027 | 55 | 11 | 507,8 | 19325 | Rab10 | 1 | 1,15 | 8,37E-01 |
| Q9CVB6 | 40 | 14 | 186,04 | 76709 | Arpc2 | 0 | 0,96 | 9,76E-01 |
| Q8BVI4 | 47 | 7 | 152,15 | 110391 | Qdpr | 0 | 0,89 | 7,99E-01 |
| O35129 | 55 | 15 | 406,82 | 12034 | Phb2 | 0 | 0,94 | 9,56E-01 |
| Q61553 | 40 | 19 | 261,85 | 14086 | Fscn1 | 0 | 0,92 | 9,21E-01 |

| | | | | | | | | |
|---|---|---|---|---|---|---|---|---|
| O88447 | 35 | 15 | 174,78 | | Klc1 | 4 | 1,22 | 7,09E-01 |
| Q9DCT2 | 41 | 13 | 167,61 | 68349 | Ndufs3 | 0 | 0,80 | 5,64E-01 |
| Q9JJK2 | 37 | 14 | 180,84 | 71835 | Lancl2 | 0 | 1,03 | 9,85E-01 |
| Q8BIZ1 | 11 | 16 | 268,49 | 77531 | Anks1b | 0 | 1,02 | 9,97E-01 |
| Q9EQF6 | 31 | 18 | 169,51 | 65254 | Dpysl5 | 0 | 1,20 | 7,38E-01 |
| Q8BKZ9 | 37 | 14 | 238,82 | 27402 | Pdhx | 0 | 1,04 | 9,77E-01 |
| Q810U4 | 20 | 19 | 170,46 | 319504 | Nrcam | 0 | 1,10 | 9,34E-01 |
| Q9QYG0 | 40 | 9 | 218,56 | 29811 | Ndrg2 | 0 | 1,04 | 9,76E-01 |
| Q8BWS5 | 30 | 17 | 156,06 | 243385 | Gprin3 | 0 | 0,91 | 9,07E-01 |
| P19157 | 56 | 9 | 141,85 | 14870 | Gstp1 | 2 | 0,85 | 7,52E-01 |
| Q9JKC6 | 70 | 10 | 152,78 | 57754 | Cend1 | 0 | 0,94 | 9,56E-01 |
| E9Q6P5 | 27 | 20 | 113,66 | 104718 | Ttc7b | 0 | 0,99 | 9,85E-01 |
| Q68FF6 | 29 | 18 | 163,67 | 216963 | Git1 | 0 | 1,17 | 8,07E-01 |
| Q60625 | 22 | 16 | 196,07 | 15898 | Icam5 | 0 | 1,23 | 7,09E-01 |
| Q9ESW4 | 44 | 15 | 203,5 | 69923 | Agk | 0 | 0,99 | 9,85E-01 |
| Q9CWK8 | 37 | 14 | 130,04 | 67804 | Snx2 | 2 | 1,07 | 9,73E-01 |
| Q91VN4 | 43 | 11 | 166,31 | 66098 | Chchd6 | 0 | 0,82 | 6,69E-01 |
| Q8BRT1 | 21 | 19 | 116,42 | 76499 | Clasp2 | 3 | 1,13 | 8,70E-01 |
| Q61327 | 26 | 17 | 403,45 | 13162 | Slc6a3 | 1 | 2,66 | 8,05E-09 |
| Q9JJY3 | 29 | 14 | 216,78 | 58994 | Smpd3 | 0 | 0,89 | 8,68E-01 |
| Q8BGH2 | 40 | 18 | 191,08 | 68653 | Samm50 | 0 | 0,76 | 3,87E-01 |
| P16054 | 25 | 19 | 112,73 | 18754 | Prkce | 0 | 1,02 | 1,00E+00 |
| P12367 | 32 | 7 | 221,7 | | Prkar2a | 0 | 1,23 | 6,80E-01 |
| P04104 | 10 | 3 | 2720,03 | 16678 | Krt1 | 1 | 1,24 | 7,36E-01 |
| Q8CGK7 | 42 | 10 | 417,97 | 14680 | Gnal | 3 | 0,87 | 8,01E-01 |
| Q9D6J6 | 50 | 10 | 266,04 | 72900 | Ndufv2 | 0 | 0,66 | 5,63E-02 |
| K1H4_HUMAN | 45 | 6 | 169,92 | | | 0 | 4,45 | 1,60E-16 |
| Q91VR2 | 39 | 14 | 398,95 | 11949 | Atp5f1c | 0 | 0,70 | 1,44E-01 |
| Q6WVG3 | 41 | 11 | 165,22 | 239217 | Kctd12 | 2 | 0,76 | 4,04E-01 |
| Q8VDM4 | 24 | 19 | 144,85 | 21762 | Psmd2 | 0 | 1,12 | 9,12E-01 |
| Q99LC3 | 31 | 15 | 266,02 | 67273 | Ndufa10 | 0 | 0,78 | 4,50E-01 |
| Q9WTT4 | 42 | 6 | 128,5 | 66237 | Atp6v1g2 | 0 | 1,20 | 7,39E-01 |
| Q9R1Q8 | 67 | 14 | 262,99 | 56370 | Tagln3 | 0 | 1,35 | 3,45E-01 |
| Q8VED5 | 18 | 3 | 1425,55 | 223917 | Krt79 | 1 | 1,33 | 6,70E-01 |
| P31938 | 24 | 7 | 238,8 | 26395 | Map2k1 | 6 | 1,01 | 1,00E+00 |
| P18760 | 64 | 10 | 281,12 | 12631 | Cfl1 | 3 | 0,94 | 9,64E-01 |
| P30275 | 32 | 15 | 219,4 | 12716 | Ckmt1 | 0 | 0,99 | 9,87E-01 |
| Q64133 | 33 | 15 | 244,19 | 17161 | Maoa | 0 | 1,18 | 7,74E-01 |
| P35438 | 23 | 24 | 271,81 | 14810 | Grin1 | 0 | 0,98 | 9,81E-01 |
| ADH1_YEAST | 42 | 14 | 163,31 | | | 0 | 1,01 | 1,00E+00 |
| Q6IFZ6 | 15 | 4 | 2408,92 | 406220 | Krt77 | 0 | 1,01 | 1,00E+00 |
| P01831 | 33 | 6 | 618,17 | 21838 | Thy1 | 0 | 0,97 | 9,76E-01 |
| P51863 | 41 | 17 | 255,93 | 11972 | Atp6v0d1 | 0 | 1,19 | 7,62E-01 |
| Q62418 | 38 | 14 | 120,77 | 13169 | Dbnl | 0 | 0,99 | 9,81E-01 |
| Q99KY4 | 17 | 19 | 111,09 | 231580 | Gak | 0 | 1,18 | 8,01E-01 |
| Q3V3R1 | 25 | 21 | 152,87 | 270685 | Mthfd1l | 0 | 1,04 | 9,78E-01 |

| | | | | | | | | |
|---|---|---|---|---|---|---|---|---|
| Q8BGQ7 | 22 | 18 | 131,1 | 234734 | Aars1 | 0 | 1,00 | 1,00E+00 |
| P70404 | 36 | 13 | 208,25 | 15929 | Idh3g | 0 | 0,96 | 9,76E-01 |
| Q9D8Y0 | 49 | 15 | 175,48 | | Efhd2 | 0 | 0,89 | 8,58E-01 |
| Q9EQZ6 | 24 | 24 | 124,07 | 56508 | Rapgef4 | 0 | 1,00 | 1,00E+00 |
| P57746 | 44 | 14 | 190,94 | 73834 | Atp6v1d | 0 | 1,17 | 8,07E-01 |
| Q9Z1L5 | 18 | 19 | 166,03 | 12294 | Cacna2d3 | 0 | 0,86 | 7,99E-01 |
| Q68FL4 | 24 | 5 | 232,8 | 74340 | Ahcyl2 | 0 | 1,10 | 9,50E-01 |
| PRDX1_HUMAN | 57 | 7 | 394,73 | 100994710; 456537; 5052 | PRDX1 | 2 | 1,47 | 1,25E-01 |
| Q8C419 | 18 | 18 | 143,09 | 241263 | Gpr158 | 0 | 1,07 | 9,74E-01 |
| Q3UHK6 | 11 | 25 | 66,34 | 23966 | Tenm4 | 6 | 1,14 | 8,66E-01 |
| Q9D1A2 | 34 | 15 | 168,2 | 66054 | Cndp2 | 0 | 1,02 | 9,94E-01 |
| Q921G7 | 33 | 18 | 143,22 | 66841 | Etfdh | 0 | 0,94 | 9,55E-01 |
| Q9QYS2 | 21 | 16 | 226,78 | 108069 | Grm3 | 0 | 0,87 | 8,02E-01 |
| P21836 | 28 | 14 | 115,95 | 11423 | Ache | 0 | 1,26 | 6,37E-01 |
| P70296 | 63 | 8 | 258,68 | 23980 | Pebp1 | 0 | 1,16 | 8,19E-01 |
| Q8R3V5 | 31 | 14 | 228,06 | 227700 | Sh3glb2 | 1 | 1,13 | 8,89E-01 |
| Q8CAA7 | 26 | 17 | 182,64 | 70974 | Pgm2l1 | 0 | 1,10 | 9,45E-01 |
| Q9CZ44 | 53 | 15 | 177,96 | 386649 | Nsfl1c | 0 | 0,95 | 9,59E-01 |
| Q9EPJ9 | 39 | 12 | 111,95 | 228998 | Arfgap1 | 0 | 1,15 | 8,42E-01 |
| Q9JM52 | 19 | 13 | 101,79 | 50932 | Mink1 | 8 | 0,94 | 9,62E-01 |
| Q14BI2 | 20 | 17 | 167,78 | 108068 | Grm2 | 3 | 0,96 | 9,76E-01 |
| Q61792 | 45 | 13 | 213,86 | 16796 | Lasp1 | 1 | 1,02 | 9,96E-01 |
| Q8BLE7 | 19 | 11 | 209,28 | 140919 | Slc17a6 | 0 | 1,08 | 9,59E-01 |
| Q9WV55 | 41 | 9 | 225,82 | 30960 | Vapa | 1 | 1,04 | 9,76E-01 |
| P48320 | 24 | 10 | 134,1 | 14417 | Gad2 | 1 | 0,96 | 9,74E-01 |
| Q80XN0 | 35 | 11 | 180,8 | 71911 | Bdh1 | 0 | 0,72 | 2,11E-01 |
| P80317 | 28 | 15 | 186,71 | 12466 | Cct6a | 0 | 1,17 | 8,06E-01 |
| O88448 | 35 | 14 | 193,81 | | Klc2 | 0 | 1,21 | 7,02E-01 |
| P21278 | 42 | 10 | 205,75 | 14672 | Gna11 | 0 | 0,91 | 9,16E-01 |
| Q9DBF1 | 32 | 16 | 159,84 | 110695 | Aldh7a1 | 0 | 0,81 | 6,06E-01 |
| Q8R429 | 15 | 14 | 255,53 | 11937 | Atp2a1 | 0 | 0,99 | 9,91E-01 |
| P31650 | 19 | 12 | 194,74 | 243616 | Slc6a11 | 1 | 0,92 | 9,34E-01 |
| Q9Z268 | 23 | 16 | 145,25 | 19415 | Rasal1 | 0 | 1,07 | 9,66E-01 |
| Q99JY9 | 36 | 12 | 215,92 | 74117 | Actr3 | 2 | 0,94 | 9,56E-01 |
| Q3UVL4 | 24 | 16 | 111,97 | 68505 | Vps51 | 0 | 1,02 | 1,00E+00 |
| O88844 | 36 | 13 | 164,96 | 15926 | Idh1 | 0 | 0,99 | 9,93E-01 |
| Q3UMR5 | 27 | 11 | 143,37 | 215999 | Mcu | 0 | 0,70 | 1,69E-01 |
| P70206 | 14 | 17 | 82,47 | 18844 | Plxna1 | 1 | 1,01 | 1,00E+00 |
| Q9ER00 | 45 | 10 | 136,44 | 100226 | Stx12 | 0 | 1,08 | 9,66E-01 |
| Q8VDQ8 | 32 | 12 | 170,47 | 64383 | Sirt2 | 0 | 0,89 | 8,82E-01 |
| Q6P9K8 | 20 | 18 | 119,3 | 268932 | Caskin1 | 0 | 1,21 | 7,09E-01 |
| Q6NZL0 | 22 | 19 | 179,59 | 67412 | Soga3 | 0 | 0,92 | 9,24E-01 |
| Q9CPW0 | 15 | 16 | 145,96 | 66797 | Cntnap2 | 0 | 1,04 | 9,78E-01 |
| P35492 | 22 | 15 | 106,96 | 15109 | Hal | 0 | 1,05 | 9,76E-01 |
| P20444 | 21 | 6 | 157,52 | 18750 | Prkca | 0 | 1,62 | 2,69E-02 |

| | | | | | | | | |
|---|---|---|---|---|---|---|---|---|
| Q80Z24 | 39 | 12 | 202,95 | 320840 | Negr1 | 0 | 0,82 | 6,42E-01 |
| Q148V7 | 20 | 21 | 106,21 | 227446 | Relch | 0 | 1,01 | 9,99E-01 |
| P51150 | 65 | 13 | 216,29 | 19349 | Rab7a | 0 | 1,05 | 9,76E-01 |
| P26040 | 24 | 9 | 157,59 | 22350 | Ezr | 9 | 0,82 | 6,46E-01 |
| Q80TB8 | 40 | 15 | 206,09 | 270097 | Vat1l | 0 | 1,26 | 6,01E-01 |
| Q3UHB1 | 30 | 17 | 176,28 | 103466 | Nt5dc3 | 0 | 0,99 | 9,85E-01 |
| P46471 | 47 | 20 | 133,31 | 19181 | Psmc2 | 0 | 1,14 | 8,53E-01 |
| Q99NE5 | 17 | 21 | 75,1 | 116837 | Rims1 | 0 | 1,26 | 6,05E-01 |
| Q61753 | 28 | 13 | 212,92 | 236539 | Phgdh | 0 | 0,80 | 5,95E-01 |
| Q9JLB0 | 32 | 15 | 193,6 | 56524 | Mpp6 | 0 | 1,02 | 9,99E-01 |
| A2ALS5 | 28 | 15 | 102,14 | 110351 | Rap1gap | 1 | 1,02 | 1,00E+00 |
| Q8C8N2 | 27 | 15 | 101,25 | 320271 | Scai | 0 | 1,19 | 7,50E-01 |
| Q63844 | 43 | 8 | 172,17 | 26417 | Mapk3 | 0 | 1,23 | 6,66E-01 |
| O88643 | 31 | 7 | 110,06 | | Pak1 | 10 | 0,90 | 8,63E-01 |
| Q6R891 | 24 | 16 | 93 | 217124 | Ppp1r9b | 0 | 1,03 | 9,91E-01 |
| Q80TQ2 | 20 | 18 | 169,08 | 74256 | Cyld | 0 | 0,98 | 9,83E-01 |
| Q6IME9 | 11 | 3 | 606,85 | 105866 | Krt72 | 0 | 1,24 | 6,33E-01 |
| Q9Z0R4 | 16 | 20 | 36,21 | 16443 | Itsn1 | 4 | 1,00 | 9,97E-01 |
| Q9JLM8 | 23 | 14 | 106,41 | 13175 | Dclk1 | 0 | 1,06 | 9,76E-01 |
| O88712 | 34 | 14 | 280,91 | 13016 | Ctbp1 | 0 | 1,23 | 6,66E-01 |
| Q68FH0 | 23 | 18 | 120,63 | 227937 | Pkp4 | 0 | 1,08 | 9,56E-01 |
| P35279 | 47 | 4 | 352,8 | 19346 | Rab6a | 0 | 1,33 | 4,42E-01 |
| P70336 | 17 | 21 | 70,07 | 19878 | Rock2 | 0 | 1,06 | 9,76E-01 |
| P62881 | 31 | 10 | 163,34 | 14697 | Gnb5 | 0 | 1,18 | 7,94E-01 |
| Q99LX0 | 53 | 10 | 190,93 | 57320 | Park7 | 0 | 1,06 | 9,76E-01 |
| Q5FWK3 | 34 | 14 | 146,32 | 228359 | Arhgap1 | 0 | 1,00 | 1,00E+00 |
| Q7TMF3 | 71 | 11 | 163,38 | 66414 | Ndufa12 | 0 | 1,17 | 8,01E-01 |
| Q9CRB9 | 31 | 11 | 234,14 | 66075 | Chchd3 | 0 | 0,78 | 4,61E-01 |
| Q61036 | 35 | 5 | 90,33 | 18481 | Pak3 | 3 | 1,11 | 9,25E-01 |
| Q8BJ42 | 22 | 16 | 80,32 | 244310 | Dlgap2 | 1 | 0,96 | 9,76E-01 |
| A2APX8 | 9 | 9 | 51,97 | 20265 | Scn1a | 0 | 1,04 | 9,78E-01 |
| Q8JZS0 | 45 | 4 | 200,88 | 108030 | Lin7a | 6 | 1,06 | 9,76E-01 |
| Q8K183 | 32 | 9 | 184,85 | 216134 | Pdxk | 0 | 1,18 | 8,02E-01 |
| Q9EP69 | 33 | 19 | 137,91 | 83493 | Sacm1l | 0 | 1,17 | 8,09E-01 |
| Q791V5 | 39 | 10 | 250,35 | 56428 | Mtch2 | 0 | 0,75 | 3,57E-01 |
| Q99K51 | 30 | 12 | 104,63 | 102866 | Pls3 | 5 | 1,01 | 1,00E+00 |
| Q9CR68 | 25 | 10 | 196,99 | 66694 | Uqcrfs1 | 0 | 0,91 | 9,19E-01 |
| P84075 | 64 | 6 | 170,68 | 15444 | Hpca | 7 | 0,96 | 9,76E-01 |
| P63330 | 46 | 3 | 178 | 19052 | Ppp2ca | 11 | 0,90 | 8,91E-01 |
| Q7TME0 | 27 | 16 | 112,32 | 229791 | Plppr4 | 0 | 1,20 | 7,89E-01 |
| Q99PJ0 | 31 | 11 | 242,74 | | Ntm | 0 | 0,97 | 9,76E-01 |
| Q9DCJ5 | 48 | 8 | 170,74 | 68375 | Ndufa8 | 0 | 0,98 | 9,81E-01 |
| P54071 | 43 | 17 | 119,1 | 269951 | Idh2 | 1 | 0,83 | 6,74E-01 |
| Q9DB20 | 72 | 15 | 260,95 | 28080 | Atp5po | 0 | 0,75 | 3,46E-01 |
| Q8VE33 | 26 | 9 | 167,29 | | Gdap1l1 | 0 | 0,98 | 9,85E-01 |
| Q9JLV5 | 23 | 15 | 103,99 | 26554 | Cul3 | 0 | 1,08 | 9,56E-01 |
| Q61165 | 17 | 11 | 100,62 | 20544 | Slc9a1 | 0 | 0,86 | 7,86E-01 |

| | | | | | | | | |
|---|---|---|---|---|---|---|---|---|
| Q9CZT8 | 37 | 4 | 493,25 | 69908 | Rab3b | 0 | 0,94 | 9,55E-01 |
| Q9WUB3 | 23 | 10 | 164,99 | 19309 | Pygm | 0 | 0,90 | 9,02E-01 |
| Q7TT50 | 13 | 18 | 78,47 | 217866 | Cdc42bpb | 4 | 1,09 | 9,59E-01 |
| Q8JZN5 | 29 | 17 | 115,98 | 229211 | Acad9 | 0 | 0,85 | 7,37E-01 |
| Q8BP47 | 26 | 14 | 83,03 | 70223 | NARS1 | 0 | 1,42 | 1,94E-01 |
| Q9DCN2 | 32 | 10 | 158,5 | 109754 | Cyb5r3 | 0 | 1,05 | 9,77E-01 |
| O55022 | 49 | 10 | 151,04 | 53328 | Pgrmc1 | 1 | 1,12 | 9,12E-01 |
| O88544 | 33 | 11 | 129,7 | 26891 | Cops4 | 0 | 0,93 | 9,33E-01 |
| Q9CPV4 | 49 | 13 | 121,01 | 67201 | Glod4 | 0 | 0,97 | 9,76E-01 |
| Q9JKK7 | 38 | 12 | 111,63 | 50876 | Tmod2 | 1 | 0,90 | 9,02E-01 |
| Q80VP1 | 22 | 9 | 52 | 13854 | Epn1 | 0 | 1,13 | 8,85E-01 |
| O35874 | 17 | 7 | 107,98 | 55963 | Slc1a4 | 0 | 0,90 | 8,53E-01 |
| Q8R3Z5 | 35 | 10 | 104,52 | 12295 | Cacnb1 | 5 | 1,06 | 9,76E-01 |
| P83510 | 18 | 11 | 104,76 | 665113 | Tnik | 0 | 0,85 | 6,91E-01 |
| Q0KL02 | 7 | 15 | 38,4 | 223435 | Trio | 5 | 1,13 | 9,02E-01 |
| Q60829 | 69 | 7 | 106,26 | 19049 | Ppp1r1b | 0 | 0,90 | 8,53E-01 |
| Q3U1J4 | 18 | 19 | 96,65 | 13194 | Ddb1 | 0 | 0,96 | 9,76E-01 |
| P99029 | 46 | 10 | 240,97 | 54683 | Prdx5 | 0 | 0,78 | 4,77E-01 |
| P62715 | 43 | 2 | 168,46 | 19053 | Ppp2cb | 0 | 1,02 | 1,00E+00 |
| P19246 | 15 | 14 | 169,65 | 380684 | Nefh | 0 | 1,28 | 5,92E-01 |
| P33173 | 14 | 22 | 72,81 | | Kif1a | 0 | 0,92 | 8,91E-01 |
| Q9R0N7 | 31 | 12 | 134,14 | 54525 | Syt7 | 0 | 1,30 | 4,61E-01 |
| P80313 | 32 | 16 | 142,36 | 12468 | Cct7 | 0 | 1,11 | 9,18E-01 |
| P08730 | 18 | 3 | 1329,28 | 16663 | Krt13 | 2 | 1,02 | 1,00E+00 |
| P15532 | 55 | 5 | 268,43 | 18102 | Nme1 | 6 | 1,06 | 9,72E-01 |
| Q61481 | 28 | 10 | 106,58 | 18573 | Pde1a | 0 | 0,99 | 9,78E-01 |
| K1H6_HUMAN | 37 | 10 | 417,08 | 8689 | KRT36 | 5 | 4,53 | 1,60E-16 |
| Q9QY76 | 38 | 7 | 213,15 | | Vapb | 0 | 1,24 | 6,44E-01 |
| P35585 | 41 | 14 | 119,02 | 11767 | Ap1m1 | 0 | 0,92 | 9,16E-01 |
| P48758 | 54 | 11 | 171,4 | 12408 | Cbr1 | 1 | 1,02 | 1,00E+00 |
| Q3TDK6 | 53 | 14 | 148,04 | 66049 | Rogdi | 0 | 1,14 | 8,54E-01 |
| Q6PER3 | 36 | 11 | 195,62 | 100732 | Mapre3 | 2 | 1,15 | 8,37E-01 |
| Q80X80 | 27 | 12 | 112,14 | 71764 | C2cd2l | 0 | 1,29 | 5,33E-01 |
| Q9R1V6 | 13 | 11 | 146,5 | 11496 | Adam22 | 0 | 0,94 | 9,64E-01 |
| Q8BH66 | 25 | 13 | 182,77 | 73991 | Atl1 | 1 | 1,10 | 9,48E-01 |
| P97350 | 19 | 12 | 131,49 | 18772 | Pkp1 | 0 | 1,27 | 5,69E-01 |
| O54828 | 23 | 16 | 116,04 | 19739 | Rgs9 | 0 | 0,93 | 9,32E-01 |
| Q9CXJ4 | 26 | 15 | 101,28 | 74610 | Abcb8 | 0 | 0,89 | 8,60E-01 |
| Q9JLN9 | 11 | 24 | 28,66 | 56717 | Mtor | 0 | 1,32 | 4,52E-01 |
| P61294 | 45 | 4 | 333,53 | 270192 | Rab6b | 8 | 1,23 | 6,58E-01 |
| P62631 | 24 | 5 | 334,72 | 13628 | Eef1a2 | 0 | 0,94 | 9,56E-01 |
| Q8BSS9 | 16 | 16 | 83,01 | 327814 | Ppfia2 | 0 | 1,24 | 6,72E-01 |
| O35609 | 30 | 8 | 124,82 | 24045 | Scamp3 | 0 | 1,15 | 8,50E-01 |
| Q61137 | 17 | 15 | 69,31 | 11899 | Astn1 | 0 | 1,21 | 7,46E-01 |
| Q9WVK8 | 38 | 17 | 69,8 | 13116 | Cyp46a1 | 0 | 0,95 | 9,64E-01 |
| Q8BNW9 | 29 | 13 | 142,21 | 74901 | Kbtbd11 | 0 | 1,14 | 8,69E-01 |

| | | | | | | | | |
|---|---|---|---|---|---|---|---|---|
| Q8R366 | 21 | 9 | 200,78 | 140559 | Igsf8 | 0 | 1,27 | 5,52E-01 |
| Q8BIJ6 | 19 | 16 | 100,3 | 381314 | Iars2 | 0 | 0,67 | 7,30E-02 |
| P62137 | 30 | 5 | 158,06 | 19045 | Ppp1ca | 5 | 1,04 | 9,77E-01 |
| Q9CQN1 | 20 | 11 | 152,83 | 68015 | Trap1 | 0 | 1,29 | 5,24E-01 |
| Q91YQ5 | 29 | 16 | 126,85 | 103963 | Rpn1 | 0 | 0,89 | 8,57E-01 |
| Q9DBC7 | 33 | 9 | 114,93 | 19084 | Prkar1a | 3 | 0,99 | 9,81E-01 |
| Q8JZP2 | 33 | 11 | 130,95 | 27204 | Syn3 | 0 | 0,95 | 9,50E-01 |
| Q9JII6 | 50 | 14 | 154,19 | 58810 | Akr1a1 | 1 | 0,97 | 9,76E-01 |
| Q922D8 | 19 | 15 | 129,41 | 108156 | Mthfd1 | 0 | 1,04 | 9,89E-01 |
| P55012 | 11 | 10 | 97,45 | 20496 | Slc12a2 | 0 | 1,19 | 7,74E-01 |
| A2RT62 | 29 | 11 | 99,51 | 214931 | Fbxl16 | 0 | 0,98 | 9,78E-01 |
| Q8C729 | 29 | 14 | 149,75 | 213056 | Fam126b | 0 | 1,01 | 1,00E+00 |
| P61205 | 41 | 6 | 207,28 | 11842 | Arf3 | 3 | 1,22 | 6,92E-01 |
| P84078 | 41 | 6 | 207,28 | 11840 | Arf1 | 0 | 1,22 | 6,92E-01 |
| Q8BHE3 | 23 | 6 | 114,6 | 16467 | Atcay | 0 | 0,93 | 9,27E-01 |
| Q8K400 | 18 | 14 | 128,4 | 78808 | Stxbp5 | 1 | 0,94 | 9,56E-01 |
| Q8BTM8 | 8 | 15 | 28,45 | 192176 | Flna | 0 | 1,00 | 1,00E+00 |
| Q9ESN6 | 21 | 12 | 74,21 | 80890 | Trim2 | 0 | 1,06 | 9,76E-01 |
| Q99JP7 | 22 | 13 | 103,74 | 207182 | Ggt7 | 0 | 0,97 | 9,76E-01 |
| P70207 | 10 | 8 | 63,41 | 18845 | Plxna2 | 0 | 0,73 | 6,42E-01 |
| Q9JLJ2 | 31 | 14 | 117,03 | 56752 | Aldh9a1 | 0 | 0,88 | 7,90E-01 |
| P32921 | 36 | 14 | 66,64 | 22375 | Wars1 | 0 | 0,97 | 9,74E-01 |
| Q9Z2W0 | 33 | 13 | 44,79 | 13437 | Dnpep | 0 | 1,08 | 9,64E-01 |
| Q9WUM4 | 27 | 13 | 157,63 | 23790 | Coro1c | 0 | 1,21 | 7,41E-01 |
| Q9QZX7 | 31 | 8 | 158,02 | 27364 | Srr | 0 | 1,01 | 1,00E+00 |
| Q9DCS9 | 45 | 8 | 196,17 | 68342 | Ndufb10 | 0 | 0,91 | 9,16E-01 |
| P35803 | 23 | 8 | 216,49 | 14758 | Gpm6b | 0 | 0,84 | 7,38E-01 |
| Q5DQR4 | 15 | 11 | 116,31 | 207227 | Stxbp5l | 0 | 0,94 | 9,48E-01 |
| P24527 | 29 | 14 | 85,88 | 16993 | Lta4h | 0 | 1,06 | 9,76E-01 |
| Q91WQ3 | 34 | 18 | 113,3 | 107271 | Yars1 | 0 | 0,93 | 9,56E-01 |
| Q2M3X8 | 22 | 14 | 108,82 | 218194 | Phactr1 | 0 | 1,06 | 9,76E-01 |
| Q00493 | 29 | 12 | 122,94 | 12876 | Cpe | 0 | 0,91 | 8,91E-01 |
| Q8BG32 | 38 | 14 | 89,95 | 69077 | Psmd11 | 0 | 1,00 | 9,93E-01 |
| Q8R5C5 | 38 | 5 | 179,29 | 226977 | Actr1b | 6 | 1,08 | 9,56E-01 |
| P18572 | 19 | 7 | 246,78 | 12215 | Bsg | 0 | 0,75 | 3,18E-01 |
| P63001 | 41 | 4 | 206,04 | 19353 | Rac1 | 3 | 1,33 | 3,83E-01 |
| Q8R5M8 | 21 | 9 | 102,29 | 54725 | Cadm1 | 0 | 1,06 | 9,74E-01 |
| P23506 | 46 | 10 | 62,36 | | Pcmt1 | 0 | 1,00 | 9,96E-01 |
| Q8BH95 | 35 | 9 | 116,15 | 93747 | Echs1 | 0 | 0,83 | 6,58E-01 |
| Q9WV80 | 29 | 13 | 87,22 | 56440 | Snx1 | 0 | 1,05 | 9,76E-01 |
| Q99JR1 | 45 | 10 | 219,56 | 14057 | Sfxn1 | 0 | 1,12 | 9,13E-01 |
| Q9QYB8 | 21 | 12 | 109,3 | 11519 | Add2 | 0 | 1,05 | 9,76E-01 |
| Q9JLZ3 | 32 | 11 | 117,92 | 11992 | Auh | 0 | 0,94 | 9,56E-01 |
| Q9D172 | 39 | 10 | 164,42 | 28295 | Gatd3a | 0 | 0,82 | 6,42E-01 |
| Q5H8C4 | 7 | 22 | 19,72 | 271564 | Vps13a | 0 | 1,17 | 8,12E-01 |
| Q64516 | 27 | 14 | 104,31 | 14933 | Gk | 1 | 0,89 | 8,43E-01 |
| Q8BXR1 | 13 | 8 | 106,94 | 241919 | Slc7a14 | 0 | 0,88 | 8,01E-01 |

| | | | | | | | | |
|---|---|---|---|---|---|---|---|---|
| P60487 | 35 | 8 | 105,31 | 57028 | Pdxp | 0 | 1,23 | 6,74E-01 |
| Q6WQJ1 | 16 | 17 | 79,04 | 269060 | Dagla | 0 | 0,91 | 8,88E-01 |
| Q9DC69 | 39 | 13 | 104,02 | 66108 | Ndufa9 | 0 | 0,95 | 9,66E-01 |
| Q6PHS9 | 13 | 15 | 127,31 | 56808 | Cacna2d2 | 0 | 1,10 | 9,26E-01 |
| Q91WS0 | 54 | 6 | 231,22 | 52637 | Cisd1 | 0 | 0,71 | 1,81E-01 |
| P63094 | 28 | 7 | 400,06 | 14683 | Gnas | 0 | 0,92 | 9,09E-01 |
| Q6R0H7 | 10 | 7 | 400,06 | 14683 | Gnas | 0 | 0,92 | 9,09E-01 |
| P61021 | 48 | 5 | 262,28 | 19344 | Rab5b | 4 | 1,21 | 7,09E-01 |
| Q8BYM5 | 16 | 10 | 125,48 | 245537 | Nlgn3 | 2 | 0,91 | 8,95E-01 |
| Q8R0S2 | 20 | 14 | 123,18 | 232227 | Iqsec1 | 5 | 1,19 | 7,69E-01 |
| Q6P1F6 | 29 | 9 | 202,07 | 71978 | Ppp2r2a | 4 | 1,01 | 1,00E+00 |
| P42208 | 41 | 10 | 73,54 | 18000 | Septin2 | 0 | 1,19 | 7,74E-01 |
| P00405 | 28 | 5 | 256,01 | 17709 | Mtco2 | 0 | 0,84 | 7,12E-01 |
| Q9R0N5 | 16 | 3 | 259,45 | | Syt5 | 0 | 0,96 | 9,76E-01 |
| CAS1_BOVIN | 34 | 7 | 73,53 | 282208 | CSN1S1 | 0 | 0,55 | 4,39E-02 |
| P62141 | 29 | 4 | 128,05 | 19046 | Ppp1cb | 1 | 1,12 | 9,19E-01 |
| Q9D394 | 35 | 14 | 91,86 | 52822 | Rufy3 | 0 | 1,06 | 9,74E-01 |
| Q9D0F9 | 27 | 14 | 99,68 | 72157 | Pgm1 | 0 | 1,36 | 3,20E-01 |
| Q64514 | 18 | 19 | 46,58 | 22019 | Tpp2 | 0 | 0,92 | 9,02E-01 |
| Q925N0 | 33 | 8 | 135,41 | 94282 | Sfxn5 | 0 | 0,93 | 9,32E-01 |
| O55234 | 38 | 11 | 172,68 | 19173 | Psmb5 | 0 | 1,21 | 7,13E-01 |
| O88910 | 28 | 12 | 69,4 | | Mpp3 | 0 | 1,05 | 9,76E-01 |
| Q64105 | 48 | 10 | 90,56 | | Spr | 0 | 1,08 | 9,74E-01 |
| Q9R0Q6 | 34 | 12 | 148,25 | 56443 | Arpc1a | 0 | 1,03 | 9,87E-01 |
| K1M2_SHEEP | 33 | 3 | 135,46 | | | 0 | 1,31 | 8,34E-01 |
| O88998 | 23 | 9 | 60,91 | 56177 | Olfm1 | 0 | 0,97 | 9,76E-01 |
| Q8VHH5 | 14 | 9 | 128,58 | | Agap3 | 2 | 1,01 | 9,99E-01 |
| Q8R0A7 | 31 | 12 | 120,7 | 234797 | Kiaa0513 | 0 | 0,96 | 9,70E-01 |
| Q9CQX2 | 45 | 6 | 98,92 | 66427 | Cyb5b | 0 | 0,61 | 1,29E-02 |
| Q9CWJ9 | 31 | 15 | 78,89 | 108147 | Atic | 0 | 1,35 | 3,18E-01 |
| Q6PFD5 | 20 | 15 | 103,84 | 242667 | Dlgap3 | 0 | 1,14 | 8,53E-01 |
| P48678 | 23 | 17 | 38,91 | 16905 | Lmna | 0 | 1,00 | 9,94E-01 |
| Q56A07 | 37 | 8 | 130,33 | 72821 | Scn2b | 0 | 0,83 | 6,75E-01 |
| Q812A2 | 17 | 17 | 85,3 | 259302 | Srgap3 | 0 | 1,11 | 9,19E-01 |
| Q01768 | 47 | 2 | 216,79 | 18103 | Nme2 | 0 | 1,13 | 8,95E-01 |
| Q3UYC0 | 34 | 13 | 102,54 | 319468 | Ppm1h | 0 | 0,99 | 9,87E-01 |
| Q9Z140 | 28 | 13 | 106,2 | 12891 | Cpne6 | 0 | 1,14 | 8,93E-01 |
| Q6PAJ1 | 16 | 15 | 74,29 | 110279 | Bcr | 2 | 1,15 | 8,59E-01 |
| O88741 | 21 | 9 | 179,85 | 14545 | Gdap1 | 0 | 0,96 | 9,74E-01 |
| P61164 | 35 | 4 | 167,06 | 54130 | Actr1a | 0 | 1,11 | 9,24E-01 |
| Q5SSL4 | 18 | 11 | 87,63 | 109934 | Abr | 0 | 0,92 | 9,14E-01 |
| P63321 | 34 | 5 | 174,75 | 56044 | Rala | 6 | 1,21 | 7,39E-01 |
| P63087 | 25 | 2 | 125,96 | 19047 | Ppp1cc | 0 | 1,00 | 9,99E-01 |
| Q8R016 | 24 | 11 | 92,24 | 104184 | Blmh | 0 | 0,67 | 4,66E-02 |
| P70414 | 12 | 11 | 99,99 | 20541 | Slc8a1 | 0 | 0,98 | 9,81E-01 |
| Q61166 | 44 | 9 | 101,79 | 13589 | Mapre1 | 0 | 1,21 | 6,96E-01 |

| | | | | | | | | |
|---|---|---|---|---|---|---|---|---|
| O88485 | 25 | 10 | 107,38 | 13426 | Dync1i1 | 2 | 1,20 | 7,33E-01 |
| P11679 | 12 | 2 | 1144,08 | 16691 | Krt8 | 1 | 0,69 | 1,41E-01 |
| Q9D1T0 | 20 | 11 | 92,97 | 235402 | Lingo1 | 0 | 1,05 | 9,78E-01 |
| CATD_HUMAN | 26 | 9 | 150,15 | 1509 | CTSD; HEL-S-130P | 2 | 1,11 | 9,28E-01 |
| O35857 | 31 | 13 | 52,99 | 21856 | Timm44 | 0 | 1,08 | 9,74E-01 |
| Q8R5H6 | 20 | 9 | 134,25 | 83767 | Wasf1 | 0 | 1,39 | 2,14E-01 |
| Q5SWU9 | 10 | 20 | 26,22 | 107476 | Acaca | 0 | 1,30 | 5,06E-01 |
| Q7TSF1 | 6 | 8 | 153,4 | 225256 | Dsg1b | 0 | 1,04 | 9,78E-01 |
| O88343 | 13 | 11 | 87,14 | 54403 | Slc4a4 | 1 | 0,72 | 1,34E-01 |
| Q62419 | 33 | 7 | 174,55 | 20405 | Sh3gl1 | 0 | 1,22 | 7,45E-01 |
| Q8CAY6 | 37 | 12 | 96,79 | 110460 | Acat2 | 0 | 1,06 | 9,76E-01 |
| Q62277 | 16 | 6 | 499,45 | 20977 | Syp | 0 | 1,01 | 1,00E+00 |
| Q07076 | 33 | 14 | 153,22 | 11750 | Anxa7 | 0 | 1,07 | 9,70E-01 |
| Q9D2G2 | 25 | 11 | 246,15 | 78920 | Dlst | 0 | 0,89 | 8,58E-01 |
| Q60737 | 25 | 7 | 124,46 | 12995 | Csnk2a1 | 1 | 1,01 | 1,00E+00 |
| Q80TS3 | 9 | 12 | 101,51 | 319387 | Adgrl3 | 0 | 0,89 | 8,49E-01 |
| Q9CPQ3 | 67 | 6 | 93,53 | 223696 | Tomm22 | 0 | 0,84 | 7,06E-01 |
| P41216 | 25 | 17 | 119,57 | 14081 | Acsl1 | 0 | 0,76 | 2,99E-01 |
| Q9DCW4 | 49 | 12 | 199,88 | 110826 | Etfb | 0 | 0,95 | 9,69E-01 |
| Q8BLK3 | 33 | 11 | 254,07 | | Lsamp | 0 | 1,10 | 9,32E-01 |
| Q6NS52 | 23 | 14 | 112,54 | 217480 | Dgkb | 0 | 0,97 | 9,76E-01 |
| Q8BSL7 | 30 | 5 | 171,03 | 11841 | Arf2 | 0 | 1,22 | 6,94E-01 |
| Q03059 | 21 | 12 | 79,66 | | Chat | 0 | 0,97 | 9,74E-01 |
| Q9CWS0 | 39 | 8 | 139,01 | 69219 | Ddah1 | 1 | 0,90 | 9,00E-01 |
| Q8C163 | 23 | 8 | 80,72 | 208194 | Exog | 0 | 0,57 | 3,46E-03 |
| P29341 | 21 | 13 | 132,18 | 18458 | Pabpc1 | 0 | 1,13 | 8,97E-01 |
| Q61171 | 63 | 10 | 257,15 | 21672 | Prdx2 | 0 | 1,32 | 4,18E-01 |
| A2AQ25 | 12 | 19 | 37,94 | 208618 | Skt | 0 | 1,08 | 9,64E-01 |
| Q9Z2W8 | 13 | 5 | 108,87 | 14802 | Gria4 | 0 | 1,25 | 8,50E-01 |
| Q8BMG7 | 14 | 18 | 58,84 | 98732 | Rab3gap2 | 0 | 1,01 | 1,00E+00 |
| Q8R570 | 35 | 11 | 125,84 | 67826 | Snap47 | 0 | 0,94 | 9,42E-01 |
| Q9Z2U0 | 39 | 10 | 122,6 | 26444 | Psma7 | 0 | 1,13 | 8,89E-01 |
| Q9CPY7 | 35 | 14 | 93,67 | 66988 | Lap3 | 0 | 0,71 | 1,33E-01 |
| Q80Z38 | 15 | 15 | 84,82 | 210274 | Shank2 | 0 | 1,09 | 9,56E-01 |
| P51660 | 22 | 13 | 109,08 | 15488 | Hsd17b4 | 0 | 1,00 | 1,00E+00 |
| P49615 | 35 | 8 | 131,95 | 12568 | Cdk5 | 0 | 1,24 | 6,74E-01 |
| P50247 | 25 | 9 | 172,49 | 11615; 269378 | Ahcy | 0 | 1,04 | 9,79E-01 |
| Q6PE13 | 19 | 13 | 78,85 | 210673 | Prrt3 | 0 | 1,02 | 1,00E+00 |
| Q8BHN3 | 15 | 13 | 90,27 | 14376 | Ganab | 0 | 0,69 | 1,21E-01 |
| Q91ZJ5 | 29 | 15 | 112,87 | 216558 | Ugp2 | 0 | 1,05 | 9,76E-01 |
| P03995 | 36 | 14 | 126,14 | 14580 | Gfap | 0 | 0,97 | 9,74E-01 |
| Q91ZP9 | 27 | 11 | 72,02 | 117148 | Necab2 | 0 | 1,09 | 9,59E-01 |
| B9EJA2 | 13 | 18 | 63,76 | 30785 | Cttnbp2 | 0 | 1,12 | 9,11E-01 |
| Q9D1G1 | 55 | 4 | 369,43 | 76308 | Rab1b | 0 | 1,11 | 9,17E-01 |
| P61202 | 30 | 12 | 85,18 | 12848 | Cops2 | 0 | 0,88 | 8,04E-01 |

| | | | | | | | | |
|---|---|---|---|---|---|---|---|---|
| Q9DC51 | 30 | 5 | 419,39 | 14679 | Gnai3 | 0 | 0,81 | 5,72E-01 |
| Q7TQI3 | 40 | 9 | 164,45 | 107260 | Otub1 | 0 | 0,86 | 7,61E-01 |
| E9Q7X7 | 10 | 10 | 91,04 | | Nrxn2 | 0 | 1,05 | 9,76E-01 |
| Q9QZF2 | 23 | 9 | 98,16 | 14733 | Gpc1 | 0 | 0,87 | 7,89E-01 |
| P26043 | 19 | 4 | 88,29 | 19684 | Rdx | 0 | 1,02 | 1,00E+00 |
| Q61495 | 5 | 7 | 150,15 | 13510 | Dsg1a | 0 | 0,99 | 9,87E-01 |
| Q60676 | 30 | 13 | 54,84 | 19060 | Ppp5c | 0 | 1,26 | 6,23E-01 |
| Q5SRX1 | 26 | 9 | 65,6 | 216810 | Tom1l2 | 0 | 1,15 | 8,85E-01 |
| Q925I1 | 27 | 15 | 125,17 | 108888 | Atad3 | 0 | 0,89 | 8,70E-01 |
| Q99K85 | 30 | 10 | 152,01 | 107272 | Psat1 | 0 | 0,81 | 5,42E-01 |
| P48036 | 39 | 13 | 94,25 | 11747 | Anxa5 | 0 | 0,95 | 9,74E-01 |
| O08529 | 20 | 12 | 80,53 | 12334 | Capn2 | 0 | 1,09 | 9,56E-01 |
| P61226 | 40 | 5 | 139,86 | 74012 | Rap2b | 3 | 0,90 | 9,02E-01 |
| P62812 | 25 | 9 | 135,42 | 14394 | Gabra1 | 4 | 1,12 | 9,19E-01 |
| Q8BWT1 | 39 | 12 | 104,81 | 52538 | Acaa2 | 0 | 0,60 | 6,52E-03 |
| Q8BLF1 | 21 | 10 | 118,85 | 320024 | Nceh1 | 0 | 0,85 | 7,38E-01 |
| Q9R0X4 | 28 | 13 | 105,49 | 56360 | Acot9 | 0 | 1,00 | 9,85E-01 |
| Q9WTP7 | 46 | 11 | 62,41 | 56248 | Ak3 | 0 | 0,79 | 5,24E-01 |
| P14685 | 32 | 14 | 31,61 | 22123 | Psmd3 | 0 | 1,22 | 6,98E-01 |
| P49025 | 9 | 19 | 41,86 | 12704 | Cit | 0 | 1,25 | 6,42E-01 |
| Q8CIN4 | 27 | 4 | 59,28 | 224105 | Pak2 | 0 | 1,01 | 1,00E+00 |
| Q9QYB5 | 18 | 11 | 94,64 | 27360 | Add3 | 0 | 1,05 | 9,76E-01 |
| O55126 | 27 | 10 | 136,83 | | Nipsnap2 | 0 | 0,90 | 8,93E-01 |
| Q8BL65 | 26 | 11 | 61,27 | 231148 | Ablim2 | 0 | 1,40 | 2,41E-01 |
| Q8CGF6 | 13 | 11 | 105,01 | 99512 | Wdr47 | 0 | 1,14 | 8,85E-01 |
| P27601 | 38 | 12 | 350,13 | 14674 | Gna13 | 0 | 1,03 | 9,94E-01 |
| Q6ZWR6 | 3 | 23 | 12,47 | 64009 | Syne1 | 0 | 0,98 | 9,76E-01 |
| Q3ULJ0 | 30 | 11 | 102,43 | 333433 | Gpd1l | 0 | 0,80 | 5,96E-01 |
| P37040 | 25 | 16 | 130,06 | 18984 | Por | 0 | 0,99 | 9,91E-01 |
| Q8BG51 | 22 | 10 | 87,31 | 59040 | Rhot1 | 2 | 1,00 | 9,91E-01 |
| P46638 | 52 | 12 | 253,47 | 19326 | Rab11b | 0 | 1,08 | 9,59E-01 |
| Q9DB73 | 39 | 10 | 83,97 | 72017 | Cyb5r1 | 0 | 1,26 | 6,21E-01 |
| Q8BR92 | 33 | 11 | 103,52 | 677884 | Palm2 | 0 | 1,13 | 9,15E-01 |
| Q6IRU5 | 38 | 11 | 240,08 | 74325 | Cltb | 0 | 1,07 | 9,71E-01 |
| Q99LD4 | 27 | 10 | 90,01 | | Gps1 | 0 | 1,46 | 1,12E-01 |
| Q68EF6 | 21 | 10 | 63,9 | 380785 | Begain | 0 | 0,96 | 9,71E-01 |
| P30999 | 17 | 14 | 56,44 | 12388 | Ctnnd1 | 0 | 0,97 | 9,74E-01 |
| Q9JMA1 | 23 | 11 | 80,96 | 59025 | Usp14 | 0 | 1,19 | 7,50E-01 |
| P61028 | 42 | 3 | 346,64 | 235442 | Rab8b | 2 | 1,30 | 4,98E-01 |
| O08756 | 28 | 5 | 73,49 | | Hsd17b10 | 0 | 0,69 | 1,30E-01 |
| Q9QZ06 | 53 | 11 | 96,23 | 54473 | Tollip | 0 | 0,91 | 8,85E-01 |
| Q78ZA7 | 23 | 6 | 78,21 | 17955 | Nap1l4 | 1 | 0,89 | 8,56E-01 |
| P60335 | 29 | 5 | 119,42 | 23983 | Pcbp1 | 4 | 1,05 | 9,76E-01 |
| Q61290 | 8 | 13 | 44,16 | | Cacna1e | 1 | 1,16 | 8,59E-01 |
| O35382 | 16 | 13 | 55,33 | 20336 | Exoc4 | 0 | 1,08 | 9,65E-01 |
| P05480 | 28 | 10 | 85,36 | | Src | 1 | 1,01 | 1,00E+00 |
| O54829 | 32 | 9 | 142,47 | 24012 | Rgs7 | 4 | 1,20 | 7,39E-01 |

| | | | | | | | | |
|---|---|---|---|---|---|---|---|---|
| Q61330 | 13 | 12 | 105,88 | 21367 | Cntn2 | 0 | 1,12 | 9,23E-01 |
| O35239 | 25 | 14 | 76,2 | 56294 | Ptpn9 | 0 | 1,02 | 1,00E+00 |
| Q08331 | 39 | 10 | 117,88 | 12308 | Calb2 | 1 | 1,15 | 8,53E-01 |
| Q61233 | 23 | 9 | 35,85 | 18826 | Lcp1 | 0 | 0,40 | 6,88E-05 |
| Q99MR8 | 21 | 11 | 52,68 | 72039 | Mccc1 | 0 | 0,80 | 5,37E-01 |
| Q61102 | 20 | 13 | 75,68 | 11306 | Abcb7 | 0 | 0,95 | 9,64E-01 |
| P51174 | 21 | 8 | 100,77 | 11363 | Acadl | 0 | 0,89 | 8,43E-01 |
| P28656 | 15 | 5 | 78,07 | 53605 | Nap1l1 | 0 | 0,94 | 9,56E-01 |
| O55023 | 31 | 10 | 156,87 | | Impa1 | 0 | 1,06 | 9,76E-01 |
| P03921 | 9 | 4 | 101,01 | 17721 | Mtnd5 | 0 | 0,92 | 9,19E-01 |
| O88685 | 27 | 9 | 71,37 | 19182 | Psmc3 | 0 | 1,08 | 9,64E-01 |
| Q9WVK4 | 19 | 5 | 127,66 | 13660 | Ehd1 | 0 | 0,85 | 7,77E-01 |
| P35278 | 39 | 4 | 243,87 | 19345 | Rab5c | 0 | 1,23 | 6,58E-01 |
| Q9CQJ8 | 50 | 8 | 73,42 | 66218 | Ndufb9 | 0 | 0,89 | 8,40E-01 |
| Q9DBG6 | 16 | 7 | 69,79 | 20014 | Rpn2 | 0 | 0,91 | 9,01E-01 |
| P62492 | 49 | 11 | 243,23 | 53869 | Rab11a | 0 | 1,14 | 8,70E-01 |
| P46425 | 33 | 4 | 80,44 | 14869 | Gstp2 | 0 | 0,72 | 1,44E-01 |
| P33175 | 12 | 2 | 72,78 | 16572 | Kif5a | 0 | 1,03 | 1,00E+00 |
| Q9CZ42 | 31 | 8 | 91,51 | 69225 | Naxd | 0 | 1,04 | 9,85E-01 |
| Q99JI6 | 44 | 3 | 94,09 | 215449 | Rap1b | 7 | 0,99 | 9,87E-01 |
| P08228 | 47 | 6 | 194,3 | 20655 | Sod1 | 1 | 1,08 | 9,56E-01 |
| Q9CRD0 | 28 | 7 | 81,05 | 68095 | Ociad1 | 0 | 0,71 | 1,64E-01 |
| Q3UUG6 | 22 | 13 | 73,07 | 224617 | Tbc1d24 | 0 | 1,09 | 9,62E-01 |
| P58404 | 14 | 7 | 73,99 | 97387 | Strn4 | 0 | 1,01 | 1,00E+00 |
| Q9CQW1 | 57 | 10 | 98,95 | 56418 | Ykt6 | 0 | 1,32 | 4,19E-01 |
| Q9D880 | 31 | 8 | 65,76 | 66525 | Timm50 | 0 | 0,98 | 9,85E-01 |
| P00493 | 50 | 9 | 89,11 | 15452 | Hprt1 | 0 | 1,14 | 8,70E-01 |
| P50544 | 21 | 11 | 70,01 | 11370 | Acadvl | 0 | 0,78 | 3,97E-01 |
| Q91ZR1 | 53 | 7 | 228,38 | 19342 | Rab4b | 2 | 1,22 | 7,24E-01 |
| P16460 | 26 | 14 | 169,25 | 11898 | Ass1 | 0 | 1,25 | 6,28E-01 |
| Q8R001 | 29 | 7 | 92,46 | 212307 | Mapre2 | 0 | 0,97 | 9,76E-01 |
| Q9QUR6 | 17 | 11 | 52,78 | 19072 | Prep | 0 | 1,03 | 9,94E-01 |
| O88952 | 52 | 5 | 136,83 | 22343 | Lin7c | 0 | 1,43 | 1,88E-01 |
| P39447 | 12 | 18 | 19,9 | 21872 | Tjp1 | 0 | 0,98 | 9,76E-01 |
| P45376 | 33 | 11 | 108,79 | 11677 | Akr1b1 | 1 | 1,15 | 8,62E-01 |
| O54774 | 15 | 15 | 23,59 | 11776 | Ap3d1 | 0 | 1,15 | 8,82E-01 |
| Q8BNN1 | 23 | 8 | 85,85 | 78779 | Spata2l | 0 | 0,86 | 7,33E-01 |
| Q8C754 | 17 | 11 | 75,32 | 224705 | Vps52 | 0 | 1,10 | 9,34E-01 |
| Q62417 | 17 | 17 | 45,75 | 20411 | Sorbs1 | 0 | 0,89 | 8,57E-01 |
| Q9JM76 | 37 | 6 | 67,4 | 56378 | Arpc3 | 0 | 1,05 | 9,76E-01 |
| Q00612 | 30 | 15 | 72,75 | 14381 | G6pdx | 0 | 0,93 | 9,24E-01 |
| P54285 | 30 | 7 | 83,48 | 12297 | Cacnb3 | 0 | 1,14 | 9,16E-01 |
| A2A8L5 | 8 | 6 | 118,19 | 19268 | Ptprf | 0 | 0,80 | 5,63E-01 |
| P00920 | 44 | 9 | 73,99 | 12349 | Ca2 | 0 | 1,09 | 9,64E-01 |
| Q91YR1 | 27 | 8 | 69,7 | 19230 | Twf1 | 1 | 0,98 | 9,76E-01 |
| P63024 | 32 | 2 | 204,07 | 22319 | Vamp3 | 0 | 0,95 | 9,73E-01 |
| Q69ZK9 | 18 | 6 | 116,65 | 216856 | Nlgn2 | 1 | 1,09 | 9,64E-01 |

| | | | | | | | | |
|---|---|---|---|---|---|---|---|---|
| O35551 | 18 | 13 | 33,68 | 54189 | Rabep1 | 0 | 0,90 | 8,82E-01 |
| P48318 | 15 | 8 | 91,96 | 14415 | Gad1 | 0 | 0,97 | 9,74E-01 |
| Q8VD04 | 20 | 14 | 45,37 | 54645 | Gripap1 | 0 | 1,05 | 9,76E-01 |
| O70250 | 23 | 2 | 174,03 | 56012 | Pgam2 | 0 | 1,03 | 1,00E+00 |
| P97390 | 28 | 15 | 67,47 | 22365 | Vps45 | 0 | 1,11 | 9,34E-01 |
| Q9D7G0 | 30 | 4 | 106,77 | 19139 | Prps1 | 5 | 1,23 | 7,00E-01 |
| Q922R8 | 28 | 9 | 74,76 | 71853 | Pdia6 | 0 | 0,78 | 4,72E-01 |
| P62484 | 25 | 6 | 84,62 | 329165 | Abi2 | 0 | 0,87 | 8,01E-01 |
| Q9QZM0 | 16 | 5 | 75,24 | 54609 | Ubqln2 | 2 | 0,93 | 9,22E-01 |
| Q61081 | 23 | 8 | 68,34 | 12539 | Cdc37 | 0 | 0,96 | 9,67E-01 |
| Q9Z2H2 | 26 | 8 | 114,97 | 50779 | Rgs6 | 0 | 1,25 | 6,17E-01 |
| O54865 | 21 | 12 | 66,66 | 54195 | Gucy1b1 | 0 | 1,00 | 9,85E-01 |
| Q9CR95 | 37 | 8 | 123,65 | 67602 | Necap1 | 0 | 0,92 | 9,25E-01 |
| Q6GQS1 | 28 | 10 | 79,91 | 66972 | Slc25a23 | 3 | 0,83 | 6,66E-01 |
| O35678 | 32 | 8 | 89,1 | 23945 | Mgll | 0 | 1,93 | 4,54E-04 |
| Q8CHG7 | 14 | 16 | 21,73 | | Rapgef2 | 0 | 1,10 | 9,37E-01 |
| P62748 | 45 | 3 | 133,91 | 53602 | Hpcal1 | 0 | 1,10 | 9,48E-01 |
| Q9ERS2 | 51 | 8 | 128,97 | 67184 | Ndufa13 | 0 | 0,83 | 6,98E-01 |
| Q80U40 | 15 | 11 | 52,1 | 231760 | Rimbp2 | 0 | 1,01 | 1,00E+00 |
| Q9R0Y5 | 45 | 8 | 110,86 | 11636 | Ak1 | 0 | 1,03 | 9,79E-01 |
| Q91YJ2 | 21 | 8 | 77,42 | 69150 | Snx4 | 0 | 1,16 | 8,49E-01 |
| Q3UJU9 | 24 | 8 | 64,8 | 67809 | Rmdn3 | 0 | 0,72 | 1,88E-01 |
| Q8CBY8 | 27 | 9 | 75,4 | 67665 | Dctn4 | 0 | 1,39 | 2,85E-01 |
| Q9JIA1 | 22 | 11 | 89,13 | 56839 | Lgi1 | 0 | 1,47 | 1,55E-01 |
| Q9DCD0 | 22 | 10 | 75,6 | 110208 | Pgd | 0 | 1,05 | 9,78E-01 |
| P47802 | 31 | 10 | 84,45 | | Mtx1 | 0 | 0,65 | 5,16E-02 |
| O70435 | 35 | 9 | 92,03 | 19167 | Psma3 | 0 | 1,02 | 9,94E-01 |
| Q8C0T5 | 13 | 17 | 21,79 | 217692 | Sipa1l1 | 0 | 1,03 | 9,87E-01 |
| Q9CX34 | 31 | 9 | 100,96 | 67955 | Sugt1 | 0 | 0,99 | 9,81E-01 |
| Q3TXS7 | 15 | 11 | 79,72 | 70247 | Psmd1 | 0 | 1,03 | 9,94E-01 |
| Q9EQP2 | 18 | 6 | 75,04 | 98878 | Ehd4 | 0 | 1,34 | 4,76E-01 |
| P62897 | 50 | 10 | 120,63 | 13063 | Cycs | 0 | 0,93 | 9,56E-01 |
| Q8BHC1 | 46 | 7 | 184,55 | 67790 | Rab39b | 0 | 1,08 | 9,71E-01 |
| Q8C1A5 | 19 | 11 | 62,79 | 50492 | Thop1 | 0 | 0,95 | 9,56E-01 |
| P26039 | 7 | 9 | 41,84 | 21894 | Tln1 | 0 | 0,74 | 6,42E-01 |
| P55258 | 39 | 2 | 330,66 | 17274 | Rab8a | 0 | 3,22 | 1,26E-03 |
| Q99L13 | 34 | 9 | 93,14 | 58875 | Hibadh | 0 | 1,06 | 9,76E-01 |
| Q6PCP5 | 35 | 8 | 69,31 | 75734 | Mff | 0 | 0,97 | 9,74E-01 |
| P21107 | 31 | 12 | 104,27 | 59069 | Tpm3 | 0 | 1,21 | 7,10E-01 |
| Q62443 | 19 | 9 | 63,02 | 18164 | Nptx1 | 0 | 0,93 | 9,19E-01 |
| Q9R1P4 | 41 | 10 | 127,66 | 26440 | Psma1 | 0 | 1,15 | 8,53E-01 |
| Q91X97 | 33 | 3 | 121,38 | 52589 | Ncald | 0 | 0,89 | 8,47E-01 |
| Q9JKY5 | 14 | 12 | 49,35 | 29816 | Hip1r | 1 | 1,17 | 8,25E-01 |
| Q8R071 | 26 | 10 | 72,12 | 228550 | Itpka | 0 | 1,09 | 9,59E-01 |
| Q9WUR9 | 46 | 10 | 61,77 | 11639 | Ak4 | 0 | 0,99 | 9,91E-01 |
| Q8K0S0 | 28 | 8 | 115,07 | 105653 | Phyhip | 0 | 0,95 | 9,59E-01 |
| Q8VEK0 | 30 | 9 | 93,32 | 69981 | Tmem30a | 0 | 1,05 | 9,76E-01 |

| | | | | | | | | |
|---|---|---|---|---|---|---|---|---|
| P12658 | 39 | 8 | 65,3 | 12307 | Calb1 | 0 | 1,31 | 4,64E-01 |
| Q8K3H0 | 18 | 9 | 49,62 | 72993 | Appl1 | 0 | 1,06 | 9,76E-01 |
| Q3UV70 | 25 | 11 | 21,62 | 381511 | Pdp1 | 0 | 0,80 | 6,17E-01 |
| P0DI97 | 23 | 8 | 67,17 | 18189 | Nrxn1 | 0 | 1,13 | 8,91E-01 |
| Q99JI4 | 27 | 11 | 88,28 | 66413 | Psmd6 | 0 | 1,36 | 3,38E-01 |
| P70297 | 21 | 9 | 57,18 | 20844 | Stam | 0 | 1,07 | 9,74E-01 |
| Q9WUM5 | 35 | 10 | 105,58 | 56451 | Suclg1 | 0 | 1,06 | 9,74E-01 |
| O88487 | 17 | 8 | 53,91 | 13427 | Dync1i2 | 0 | 0,86 | 7,78E-01 |
| Q8BYI9 | 11 | 13 | 52,2 | 21960 | Tnr | 0 | 1,16 | 8,57E-01 |
| Q8K386 | 38 | 5 | 296,07 | | Rab15 | 0 | 0,97 | 9,74E-01 |
| P10630 | 28 | 2 | 112,82 | 13682 | Eif4a2 | 0 | 1,41 | 6,86E-01 |
| Q9D593 | 17 | 6 | 97,79 | 74915 | Atp6v1e2 | 0 | 1,26 | 5,86E-01 |
| Q6PHN9 | 41 | 6 | 319,63 | 77407 | Rab35 | 1 | 1,06 | 9,74E-01 |
| Q9Z1Z2 | 38 | 10 | 51,64 | 20901 | Strap | 0 | 0,92 | 9,09E-01 |
| Q9JHR7 | 13 | 12 | 34,19 | | Ide | 0 | 1,18 | 7,97E-01 |
| P0C192 | 15 | 7 | 67,72 | 272381 | Lrrc4b | 1 | 1,04 | 9,76E-01 |
| Q9CR16 | 33 | 11 | 61,05 | 67738 | Ppid | 0 | 1,13 | 8,91E-01 |
| Q9QUI0 | 33 | 9 | 98,97 | 11848 | Rhoa | 0 | 1,03 | 9,85E-01 |
| P62334 | 27 | 10 | 88,8 | 67089 | Psmc6 | 0 | 0,82 | 6,13E-01 |
| P35235 | 22 | 13 | 60,92 | 19247 | Ptpn11 | 0 | 1,00 | 9,94E-01 |
| O35954 | 13 | 14 | 55,53 | 18739 | Pitpnm1 | 0 | 1,13 | 9,11E-01 |
| Q9Z1N9 | 6 | 12 | 72,34 | 22249 | Unc13b | 0 | 1,26 | 5,82E-01 |
| Q99L47 | 20 | 8 | 76,64 | 70356 | St13 | 0 | 1,11 | 9,19E-01 |
| Q9WTR1 | 17 | 10 | 55,63 | 22368 | Trpv2 | 0 | 1,13 | 9,21E-01 |
| O08585 | 26 | 11 | 166,91 | | Clta | 0 | 1,01 | 1,00E+00 |
| Q01405 | 17 | 8 | 63,95 | 20334 | Sec23a | 1 | 1,25 | 6,75E-01 |
| P26041 | 23 | 7 | 70,5 | 17698 | Msn | 1 | 0,83 | 6,42E-01 |
| Q8BGT8 | 38 | 10 | 76,74 | 70911 | Phyhipl | 4 | 0,98 | 9,85E-01 |
| P07744 | 12 | 2 | 422,95 | 16682 | Krt4 | 0 | 0,69 | 5,69E-01 |
| P52503 | 45 | 7 | 48,17 | 407785 | Ndufs6 | 0 | 0,81 | 5,69E-01 |
| Q9WV60 | 36 | 8 | 82,94 | 56637 | Gsk3b | 3 | 1,01 | 1,00E+00 |
| Q8CJ19 | 9 | 17 | 10,98 | 194401 | Mical3 | 0 | 1,07 | 9,74E-01 |
| Q91ZZ3 | 50 | 3 | 211,73 | 104069 | Sncb | 0 | 0,80 | 5,25E-01 |
| Q8CBW3 | 25 | 6 | 79,61 | 11308 | Abi1 | 4 | 1,11 | 9,25E-01 |
| P48774 | 42 | 9 | 120,88 | 14866 | Gstm5 | 0 | 0,86 | 7,61E-01 |
| Q9D6J5 | 46 | 6 | 74 | 67264 | Ndufb8 | 0 | 0,72 | 2,01E-01 |
| Q8C985 | 18 | 8 | 98,27 | 18191 | Nrxn3 | 0 | 1,33 | 4,06E-01 |
| O08547 | 39 | 7 | 103,62 | 20333 | Sec22b | 0 | 0,94 | 9,56E-01 |
| Q3UHD6 | 21 | 10 | 90,45 | 76742 | Snx27 | 0 | 1,04 | 9,85E-01 |
| Q6P1B1 | 18 | 10 | 72,19 | 170750 | Xpnpep1 | 0 | 0,84 | 7,09E-01 |
| P29387 | 21 | 2 | 314,38 | 14696 | Gnb4 | 0 | 1,02 | 1,00E+00 |
| O35685 | 34 | 10 | 66,96 | 18221 | Nudc | 0 | 0,79 | 5,23E-01 |
| P08003 | 27 | 13 | 20,39 | 12304 | Pdia4 | 0 | 0,64 | 3,07E-02 |
| O88951 | 42 | 4 | 98,84 | 22342 | Lin7b | 0 | 1,01 | 1,00E+00 |
| Q9JKR6 | 12 | 10 | 56,82 | 12282 | Hyou1 | 0 | 0,91 | 8,85E-01 |
| P63141 | 19 | 3 | 99,21 | 16490 | Kcna2 | 1 | 0,92 | 9,56E-01 |
| Q8QZS1 | 27 | 9 | 73,65 | 227095 | Hibch | 0 | 0,87 | 7,91E-01 |

| | | | | | | | | |
|---|---|---|---|---|---|---|---|---|
| A2CG49 | 5 | 11 | 34,61 | 545156 | Kalrn | 0 | 0,77 | 4,72E-01 |
| P97441 | 18 | 7 | 100,1 | 22784 | Slc30a3 | 0 | 1,04 | 9,78E-01 |
| O55125 | 28 | 8 | 120,36 | 18082 | Nipsnap1 | 0 | 0,81 | 6,28E-01 |
| Q69ZS6 | 11 | 8 | 119,37 | 75209 | Sv2c | 0 | 2,24 | 1,17E-05 |
| P0DP27 | 46 | 7 | 122,34 | 12313; 12314; 12315 | Calm2 | 0 | 0,82 | 6,66E-01 |
| P13707 | 27 | 11 | 70,2 | 14555 | Gpd1 | 0 | 0,96 | 9,71E-01 |
| P56371 | 47 | 6 | 199,13 | 19341 | Rab4a | 0 | 1,48 | 1,82E-01 |
| P09671 | 33 | 9 | 162,84 | 20656 | Sod2 | 0 | 0,82 | 6,53E-01 |
| Q8K221 | 29 | 9 | 70,39 | 76932 | Arfip2 | 0 | 1,02 | 1,00E+00 |
| P80560 | 10 | 8 | 66,75 | 19276 | Ptprn2 | 1 | 1,08 | 9,64E-01 |
| P60843 | 27 | 2 | 81,24 | 13681 | Eif4a1 | 6 | 0,94 | 9,45E-01 |
| Q9D8N0 | 19 | 8 | 87,75 | 67160 | Eef1g | 0 | 1,04 | 9,83E-01 |
| P62984 | 46 | 7 | 281,85 | 22186 | Uba52 | 0 | 0,92 | 9,25E-01 |
| RS27A_HUMAN | 38 | 7 | 281,85 | 100032038; 100052644; 100286787; 100394663; 100401239; 100411672; 100476697; 100514637; 100985757; 101020976; 101029100; 101059860; 101092929; 101136689; 101316708; 101361983; 101385092; 101571052; 101674222; 101710786; 101822924; | HEL112; LOC100411672; LOC101361983; LOC102746862; LOC102816131; LOC111148109; LOC116566029; rps27a; Rps27a; RPS27A; rps27a.S | 0 | 0,92 | 9,25E-01 |

| | | | | | | | | |
|---|---|---|---|---|---|---|---|---|
| | | | | 101864869; 101962652; 102142514; 102187139; 102420450; 102527818; 102746286; 102746862; 102816131; 102926163; 102976798; 103010032; 103088763; 103121632; 103220242; 103671271; 104856443; 104989863; 105294656; 105465049; 105521178; 105522856; 105536598; 105551330; 105576398; 105734369; 105814769; 105986879; 106976779; 106992282; | | | | |

| | | | | | | | | |
|---|---|---|---|---|---|---|---|---|
| | | | | 108313818; 109252147; 109566084; 109681758; 286839; 444494; 454140; 474599; 548926; 6233; 718909 | | | | |
| P62983 | 38 | 7 | 281,85 | 78294 | Rps27a | 0 | 0,92 | 9,25E-01 |
| P0CG49 | 77 | 7 | 281,85 | 22187 | Ubb | 0 | 0,92 | 9,25E-01 |
| P0CG50 | 73 | 7 | 281,85 | 22190 | Ubc | 0 | 0,92 | 9,25E-01 |
| Q99JB2 | 28 | 7 | 56,53 | 66592 | Stoml2 | 0 | 0,90 | 8,85E-01 |
| Q8C0E2 | 30 | 8 | 73,39 | 69091 | Vps26b | 1 | 1,21 | 7,50E-01 |
| Q3UHD9 | 14 | 12 | 46,72 | 216439 | Agap2 | 0 | 1,04 | 9,78E-01 |
| Q8VCT3 | 24 | 12 | 53,96 | 215615 | Rnpep | 0 | 0,86 | 7,50E-01 |
| Q9D3A9 | 11 | 4 | 98,68 | 57776 | Ttyh1 | 0 | 0,92 | 9,22E-01 |
| Q6PB44 | 10 | 13 | 26,3 | 104831 | Ptpn23 | 0 | 0,90 | 8,85E-01 |
| O35658 | 15 | 5 | 67,85 | | C1qbp | 0 | 0,52 | 3,45E-04 |
| Q7TQD2 | 33 | 7 | 159,12 | 72948 | Tppp | 0 | 0,91 | 9,13E-01 |
| Q9ES28 | 19 | 13 | 45,38 | 54126 | Arhgef7 | 0 | 1,19 | 7,87E-01 |
| Q99KK7 | 18 | 10 | 39,82 | 75221 | Dpp3 | 0 | 1,21 | 7,28E-01 |
| Q9R0P3 | 24 | 6 | 52,6 | 13885 | Esd | 0 | 0,96 | 9,74E-01 |
| Q9JHI5 | 21 | 10 | 79,35 | 56357 | Ivd | 0 | 1,05 | 9,76E-01 |
| Q9ERI6 | 28 | 8 | 67,27 | 105014 | Rdh14 | 0 | 0,94 | 9,56E-01 |
| Q61503 | 16 | 8 | 82,28 | 23959 | Nt5e | 0 | 0,90 | 8,54E-01 |
| A2ARS0 | 30 | 9 | 84,95 | 383787 | Ankrd63 | 0 | 1,17 | 7,77E-01 |
| A2AGT5 | 8 | 14 | 30,44 | 75786 | Ckap5 | 0 | 1,15 | 9,09E-01 |
| Q9CQD1 | 34 | 4 | 206,84 | 271457 | Rab5a | 0 | 1,16 | 8,33E-01 |
| Q9D855 | 49 | 6 | 71,53 | | Uqcrb | 0 | 0,93 | 9,28E-01 |
| Q9ERK4 | 13 | 13 | 52,21 | 110750 | Cse1l | 0 | 1,08 | 9,61E-01 |
| Q7TMY8 | 4 | 14 | 11,95 | 59026 | Huwe1 | 0 | 1,39 | 4,83E-01 |
| GSTP1_HUMAN | 40 | 5 | 35,07 | | | 0 | 1,41 | 7,44E-01 |
| Q8R127 | 21 | 10 | 91,67 | 109232 | Sccpdh | 0 | 1,00 | 9,98E-01 |
| Q8R464 | 27 | 7 | 73,96 | 260299 | Cadm4 | 0 | 1,01 | 9,96E-01 |
| Q9CQC7 | 54 | 7 | 126,68 | 100042503; 68194 | Ndufb4 | 0 | 1,15 | 8,43E-01 |
| Q9D7X3 | 23 | 3 | 88,39 | 72349 | Dusp3 | 0 | 0,79 | 4,49E-01 |
| Q9WV18 | 13 | 12 | 49,73 | 54393 | Gabbr1 | 0 | 1,03 | 9,99E-01 |
| Q9Z1J3 | 23 | 11 | 61,51 | 18041 | Nfs1 | 0 | 1,19 | 7,46E-01 |
| Q80U56 | 19 | 11 | 65,39 | 78937 | Avl9 | 0 | 1,09 | 9,56E-01 |
| Q8VEH3 | 38 | 4 | 131,79 | 68724 | Arl8a | 0 | 1,25 | 6,47E-01 |
| Q9Z0S1 | 35 | 10 | 107,19 | 23827 | Bpnt1 | 0 | 1,10 | 9,24E-01 |

| | | | | | | | | |
|---|---|---|---|---|---|---|---|---|
| O08528 | 4 | 3 | 257,54 | 15277 | Hk2 | 0 | 0,95 | 9,74E-01 |
| Q9QUM9 | 31 | 7 | 101,96 | 26443 | Psma6 | 0 | 1,09 | 9,50E-01 |
| P39688 | 26 | 7 | 118,86 | 14360 | Fyn | 7 | 0,94 | 9,64E-01 |
| Q9WTS4 | 6 | 13 | 23,16 | 23963 | Tenm1 | 0 | 1,04 | 9,85E-01 |
| Q91VI7 | 29 | 10 | 73,88 | 107702 | Rnh1 | 0 | 1,15 | 8,57E-01 |
| Q8BGX2 | 26 | 6 | 63,9 | 69773 | Timm29 | 0 | 0,79 | 4,82E-01 |
| P62962 | 72 | 9 | 60,63 | 18643 | Pfn1 | 0 | 0,87 | 7,77E-01 |
| P60202 | 26 | 7 | 196,48 | 18823 | Plp1 | 0 | 0,86 | 7,90E-01 |
| Q8BKC5 | 13 | 12 | 45,46 | 70572 | Ipo5 | 0 | 0,96 | 9,72E-01 |
| Q641P0 | 28 | 8 | 68,86 | 242894 | Actr3b | 0 | 0,93 | 9,32E-01 |
| Q99LI8 | 16 | 10 | 68,58 | 15239 | Hgs | 0 | 1,14 | 8,91E-01 |
| Q9DBS2 | 32 | 7 | 85,37 | 67808 | Tprg1l | 0 | 1,04 | 9,81E-01 |
| Q63912 | 14 | 7 | 177,29 | 18377 | Omg | 0 | 0,82 | 6,61E-01 |
| Q9ERG2 | 16 | 9 | 40,99 | 94186 | Strn3 | 0 | 0,99 | 9,94E-01 |
| Q99MI1 | 11 | 5 | 104,27 | 111173 | Erc1 | 0 | 1,04 | 9,79E-01 |
| P62835 | 44 | 2 | 91,35 | 109905 | Rap1a | 0 | 0,76 | 7,46E-01 |
| P35762 | 21 | 3 | 55,28 | 12520 | Cd81 | 0 | 1,07 | 9,74E-01 |
| Q3UGY8 | 7 | 12 | 27,08 | 215821 | Arfgef3 | 0 | 1,03 | 9,94E-01 |
| Q9ESN9 | 9 | 11 | 55,06 | 30957 | Mapk8ip3 | 0 | 1,19 | 7,89E-01 |
| Q60631 | 41 | 10 | 87,78 | 14784 | Grb2 | 0 | 0,98 | 9,76E-01 |
| RASH_HUMAN | 39 | 2 | 119,8 | 100406017; 100773737; 101002992; 101047663; 101838968; 101959985; 102118744; 102905357; 103246363; 103250130; 104873574; 105494134; 105503192; 105549333; 105577198; 105998617; 108288791; | c-Ha-ras; Hras; HRAS | 5 | 1,12 | 9,08E-01 |

| | | | | | | | | |
|---|---|---|---|---|---|---|---|---|
| | | | | 108543525; 15461; 293621; 3265; 466302; 698830 | | | | |
| Q8BH04 | 20 | 10 | 52,72 | 74551 | Pck2 | 0 | 1,17 | 8,25E-01 |
| P39087 | 13 | 8 | 64,12 | | Grik2 | 0 | 0,96 | 9,74E-01 |
| Q80VP0 | 13 | 11 | 51,74 | 70381 | Tecpr1 | 0 | 1,00 | 9,85E-01 |
| Q9CQH3 | 33 | 8 | 86,09 | 66046 | Ndufb5 | 0 | 0,83 | 6,25E-01 |
| Q04690 | 7 | 18 | 12,18 | 18015 | Nf1 | 0 | 1,16 | 8,43E-01 |
| Q920N7 | 23 | 9 | 64,07 | 171180 | Syt12 | 0 | 1,14 | 8,85E-01 |
| P58389 | 31 | 10 | 114,95 | 110854 | Ptpa | 0 | 1,12 | 9,02E-01 |
| Q93092 | 34 | 11 | 106,91 | 21351 | Taldo1 | 0 | 0,86 | 7,97E-01 |
| Q6P8X1 | 27 | 12 | 57,98 | 72183 | Snx6 | 0 | 1,02 | 9,94E-01 |
| Q62442 | 55 | 3 | 68,34 | 22317 | Vamp1 | 0 | 1,06 | 9,76E-01 |
| Q6PGF7 | 14 | 11 | 41,81 | 102058 | Exoc8 | 0 | 1,19 | 7,91E-01 |
| P63080 | 19 | 5 | 71,12 | 14402 | Gabrb3 | 3 | 1,02 | 1,00E+00 |
| Q5U458 | 21 | 11 | 85,18 | 230935 | Dnajc11 | 0 | 0,82 | 6,06E-01 |
| P49443 | 24 | 6 | 68,71 | 19042 | Ppm1a | 3 | 1,14 | 8,81E-01 |
| Q8CI71 | 12 | 9 | 67,18 | 73288 | Vps50 | 0 | 0,94 | 9,41E-01 |
| Q8R2R9 | 31 | 8 | 47,82 | 64933 | Ap3m2 | 2 | 1,28 | 5,69E-01 |
| Q2NL51 | 23 | 5 | 81,49 | 606496 | Gsk3a | 0 | 0,83 | 6,94E-01 |
| P20108 | 32 | 8 | 139,99 | 11757 | Prdx3 | 0 | 1,23 | 6,72E-01 |
| P62482 | 22 | 9 | 73,83 | 16498 | Kcnab2 | 2 | 1,34 | 3,31E-01 |
| Q8C3Q5 | 19 | 8 | 32,41 | 232813 | Shisa7 | 0 | 0,93 | 9,34E-01 |
| O70439 | 31 | 7 | 128,24 | | Stx7 | 0 | 0,88 | 8,07E-01 |
| Q99J85 | 18 | 8 | 96,67 | | Nptxr | 0 | 1,08 | 9,56E-01 |
| Q6VNB8 | 5 | 16 | 17,88 | 72145 | Wdfy3 | 0 | 1,39 | 2,43E-01 |
| Q9JLF6 | 13 | 11 | 113,33 | 21816 | Tgm1 | 0 | 1,53 | 7,32E-02 |
| Q7M6Y3 | 17 | 7 | 127,32 | 233489 | Picalm | 0 | 0,98 | 9,76E-01 |
| Q8C996 | 24 | 6 | 44,85 | 72160 | Tmem163 | 0 | 0,95 | 9,64E-01 |
| Q9DC07 | 35 | 9 | 94,32 | 74103 | Nebl | 0 | 1,06 | 9,76E-01 |
| Q80T41 | 12 | 9 | 87,88 | 242425 | Gabbr2 | 0 | 1,06 | 9,76E-01 |
| Q91V09 | 20 | 9 | 57,65 | 73447 | Wdr13 | 0 | 1,10 | 9,34E-01 |
| Q9D8W5 | 20 | 9 | 68,72 | 66997 | Psmd12 | 0 | 1,28 | 5,85E-01 |
| Q9WTS5 | 5 | 12 | 18,11 | | Tenm2 | 0 | 1,13 | 9,42E-01 |
| Q9EPL8 | 10 | 9 | 38,88 | 233726 | Ipo7 | 0 | 0,85 | 7,09E-01 |
| Q9D5V5 | 13 | 11 | 55,36 | 75717 | Cul5 | 0 | 0,89 | 8,25E-01 |
| P16388 | 23 | 5 | 85,3 | 16485 | Kcna1 | 5 | 0,99 | 9,87E-01 |
| O35465 | 18 | 6 | 47,53 | 14232 | Fkbp8 | 0 | 0,74 | 3,59E-01 |
| O70172 | 22 | 5 | 67,77 | 18718 | Pip4k2a | 3 | 1,01 | 1,00E+00 |
| Q68FM6 | 12 | 8 | 55,6 | 207393 | Elfn2 | 0 | 1,04 | 9,91E-01 |
| Q9DC53 | 15 | 8 | 101,66 | 66871 | Cpne8 | 0 | 0,91 | 9,03E-01 |
| P35282 | 38 | 7 | 111,87 | 216344 | Rab21 | 0 | 1,65 | 2,89E-02 |
| Q505F5 | 19 | 8 | 43,76 | 72946 | Lrrc47 | 0 | 1,21 | 7,33E-01 |
| P54775 | 28 | 11 | 61,84 | 23996 | Psmc4 | 0 | 1,08 | 9,64E-01 |

| | | | | | | | | |
|---|---|---|---|---|---|---|---|---|
| Q80ZF8 | 10 | 10 | 32,17 | 210933 | Adgrb3 | 1 | 0,96 | 9,71E-01 |
| P45591 | 55 | 6 | 94,51 | 12632 | Cfl2 | 0 | 0,99 | 9,85E-01 |
| O54988 | 12 | 13 | 12,49 | 20874 | Slk | 0 | 1,08 | 9,69E-01 |
| O35621 | 39 | 9 | 72,94 | 29858 | Pmm1 | 0 | 1,41 | 2,56E-01 |
| Q61768 | 11 | 3 | 70,23 | 16573 | Kif5b | 0 | 2,09 | 1,01E-01 |
| Q8C437 | 20 | 9 | 46,82 | 58869 | Pex5l | 0 | 1,07 | 9,74E-01 |
| Q5DTY9 | 22 | 9 | 82,11 | 383348 | Kctd16 | 0 | 1,15 | 8,25E-01 |
| Q64518 | 8 | 7 | 115,13 | 53313 | Atp2a3 | 0 | 0,62 | 9,98E-03 |
| Q62421 | 31 | 10 | 79,97 | 20408 | Sh3gl3 | 0 | 0,85 | 7,49E-01 |
| Q6P5U7 | 7 | 11 | 44,58 | 319807 | Nwd2 | 0 | 1,43 | 1,91E-01 |
| P08414 | 17 | 7 | 71,4 | | Camk4 | 0 | 0,95 | 9,56E-01 |
| Q5SVL6 | 19 | 9 | 64,74 | 380711 | Rap1gap2 | 0 | 1,02 | 1,00E+00 |
| Q8CDN6 | 34 | 7 | 57,74 | 53382 | Txnl1 | 0 | 1,07 | 9,74E-01 |
| K1H8_HUMAN | 18 | 4 | 386,58 | | | 0 | 1,56 | 6,23E-02 |
| Q91VZ6 | 15 | 5 | 79 | 98366 | Smap1 | 0 | 1,08 | 9,67E-01 |
| Q9ESJ4 | 19 | 12 | 44,48 | 80987 | Nckipsd | 0 | 1,08 | 9,56E-01 |
| Q9D0I9 | 20 | 12 | 30,93 | 104458 | Rars1 | 0 | 0,88 | 8,42E-01 |
| Q8BPQ7 | 11 | 9 | 72,11 | 52850 | Sgsm1 | 0 | 1,24 | 6,70E-01 |
| P42669 | 26 | 6 | 107,39 | 19290 | Pura | 0 | 1,17 | 7,90E-01 |
| Q921H8 | 25 | 7 | 35,56 | 113868 | Acaa1a | 0 | 0,99 | 9,85E-01 |
| Q2PFD7 | 10 | 9 | 39,85 | 234353 | Psd3 | 0 | 1,12 | 9,07E-01 |
| Q8CGU1 | 15 | 9 | 42,44 | 67488 | Calcoco1 | 0 | 1,08 | 9,74E-01 |
| Q8BFT9 | 14 | 6 | 84,86 | 68666 | Svop | 0 | 0,92 | 9,12E-01 |
| P62746 | 26 | 7 | 66,61 | 11852 | Rhob | 0 | 0,90 | 9,02E-01 |
| Q9D4H8 | 13 | 10 | 64,16 | 71745 | Cul2 | 0 | 0,99 | 9,85E-01 |
| Q9CXW3 | 44 | 8 | 52,09 | 12301 | Cacybp | 0 | 1,13 | 9,02E-01 |
| P13020 | 14 | 5 | 57,21 | 227753 | Gsn | 6 | 1,16 | 8,42E-01 |
| Q61187 | 28 | 9 | 79,99 | 22088 | Tsg101 | 0 | 0,90 | 8,42E-01 |
| O88545 | 31 | 8 | 69,48 | 26893 | Cops6 | 0 | 1,26 | 5,66E-01 |
| P31254 | 7 | 8 | 130,34 | | Uba1y | 0 | 1,38 | 2,59E-01 |
| Q61206 | 26 | 6 | 46,73 | 18475 | Pafah1b2 | 0 | 1,10 | 9,36E-01 |
| O09111 | 49 | 6 | 74,55 | 104130 | Ndufb11 | 0 | 0,79 | 5,27E-01 |
| Q8CHP8 | 26 | 7 | 90,09 | 67078 | Pgp | 0 | 1,07 | 9,76E-01 |
| O35435 | 28 | 9 | 32,36 | 56749 | Dhodh | 0 | 0,65 | 2,06E-01 |
| Q8CFI0 | 14 | 10 | 50,67 | | Nedd4l | 1 | 0,84 | 6,53E-01 |
| K1H5_HUMAN | 16 | 3 | 371,18 | | | 0 | 0,72 | 1,81E-01 |
| Q9QWY8 | 11 | 10 | 23,73 | 13196 | Asap1 | 1 | 1,08 | 9,62E-01 |
| P62196 | 27 | 9 | 35,24 | 19184 | Psmc5 | 1 | 0,98 | 9,76E-01 |
| Q9JKK1 | 31 | 8 | 38,91 | 58244 | Stx6 | 0 | 0,90 | 8,59E-01 |
| Q8R191 | 28 | 6 | 229,3 | 20974 | Syngr3 | 0 | 1,33 | 4,00E-01 |
| Q61990 | 21 | 3 | 55,95 | 18521 | Pcbp2 | 0 | 1,20 | 8,42E-01 |
| Q05512 | 16 | 7 | 38,69 | 13728 | Mark2 | 2 | 1,16 | 8,47E-01 |
| Q8BHZ0 | 21 | 4 | 75,32 | 76820 | Cyria | 0 | 1,13 | 9,34E-01 |
| Q9CZP5 | 21 | 7 | 68,36 | 66821 | Bcs1l | 0 | 0,78 | 4,95E-01 |
| Q3UHC7 | 10 | 9 | 50,61 | 69601 | Dab2ip | 0 | 0,96 | 9,74E-01 |
| Q9QVP9 | 11 | 9 | 39,49 | 19229 | Ptk2b | 1 | 0,99 | 9,78E-01 |

| | | | | | | | | |
|---|---|---|---|---|---|---|---|---|
| O70325 | 39 | 8 | 49,42 | 625249 | Gpx4 | 0 | 0,94 | 9,39E-01 |
| Q62393 | 28 | 6 | 62,76 | 21985 | Tpd52 | 0 | 1,18 | 7,77E-01 |
| Q99L43 | 12 | 4 | 90,38 | 110911 | Cds2 | 0 | 0,93 | 9,24E-01 |
| P29758 | 23 | 9 | 47,76 | 18242 | Oat | 0 | 0,66 | 4,67E-02 |
| Q924N4 | 8 | 5 | 168,08 | 107723 | Slc12a6 | 0 | 0,78 | 4,48E-01 |
| P46664 | 24 | 8 | 56,81 | 11566 | Adss2 | 0 | 0,94 | 9,56E-01 |
| Q8CCB4 | 17 | 12 | 21,85 | 68299 | Vps53 | 0 | 0,74 | 2,77E-01 |
| Q9R1V7 | 10 | 7 | 66,34 | 23792 | Adam23 | 0 | 1,05 | 9,76E-01 |
| P47753 | 35 | 5 | 81,72 | | Capza1 | 0 | 2,19 | 1,02E-03 |
| Q3B7Z2 | 16 | 8 | 37,66 | 76303 | Osbp | 0 | 1,47 | 2,60E-01 |
| LYSC_CHICK | 44 | 6 | 38,99 | 396218 | LYZ | 0 | 1,10 | 9,74E-01 |
| O88696 | 35 | 7 | 56,38 | 53895 | Clpp | 0 | 0,97 | 9,76E-01 |
| Q9EP89 | 20 | 11 | 56,5 | 80907 | Lactb | 0 | 0,76 | 4,00E-01 |
| Q80UJ7 | 13 | 11 | 34,3 | 226407 | Rab3gap1 | 0 | 0,84 | 7,13E-01 |
| Q7TQ95 | 34 | 9 | 42,49 | 69605 | Lnpk | 0 | 1,17 | 8,37E-01 |
| Q8BIW1 | 20 | 7 | 44,37 | 229589 | Prune1 | 0 | 1,30 | 5,87E-01 |
| Q8CBE3 | 17 | 9 | 47,36 | 207615 | Wdr37 | 0 | 1,52 | 7,23E-02 |
| Q9WVE8 | 18 | 9 | 71,58 | 23970 | Pacsin2 | 0 | 0,89 | 8,50E-01 |
| Q9Z1Q2 | 19 | 8 | 55,94 | 193742 | Abhd16a | 0 | 1,02 | 1,00E+00 |
| Q91VM9 | 22 | 7 | 57,02 | 74776 | Ppa2 | 0 | 0,97 | 9,76E-01 |
| Q99KB8 | 24 | 6 | 50,7 | 14651 | Hagh | 0 | 1,40 | 2,39E-01 |
| Q61390 | 11 | 5 | 71,44 | 12467 | Cct6b | 0 | 1,45 | 1,66E-01 |
| Q8CDG3 | 12 | 11 | 39,68 | 70675 | Vcpip1 | 0 | 1,22 | 7,41E-01 |
| Q9D0M5 | 40 | 4 | 75,69 | 68097 | Dynll2 | 3 | 1,03 | 9,94E-01 |
| P26638 | 19 | 8 | 33,03 | 20226 | Sars1 | 0 | 0,88 | 8,89E-01 |
| Q60854 | 20 | 8 | 66,16 | 20719 | Serpinb6 | 0 | 1,32 | 4,29E-01 |
| Q8K2K6 | 16 | 6 | 62,73 | 15463 | Agfg1 | 0 | 1,29 | 5,52E-01 |
| Q9WVJ2 | 32 | 11 | 47,26 | 23997 | Psmd13 | 0 | 1,21 | 6,94E-01 |
| P49442 | 22 | 7 | 44,85 | 16329 | Inpp1 | 0 | 0,91 | 9,05E-01 |
| Q99MN9 | 17 | 7 | 52,77 | 66904 | Pccb | 0 | 0,76 | 5,67E-01 |
| Q9R1R2 | 13 | 8 | 53,27 | 55992 | Trim3 | 0 | 0,95 | 9,70E-01 |
| P24270 | 21 | 6 | 41,39 | 12359 | Cat | 0 | 0,97 | 9,81E-01 |
| Q9D1K2 | 43 | 6 | 34,81 | 66144 | Atp6v1f | 0 | 1,40 | 2,37E-01 |
| Q5DU25 | 9 | 8 | 40,89 | 245666 | Iqsec2 | 0 | 1,30 | 7,80E-01 |
| Q9DBP5 | 42 | 8 | 75,93 | 66588 | Cmpk1 | 0 | 0,94 | 9,49E-01 |
| O88384 | 38 | 8 | 56,07 | | Vti1b | 0 | 0,84 | 6,74E-01 |
| Q8VBZ3 | 21 | 9 | 30,02 | 56457 | Clptm1 | 0 | 1,04 | 9,87E-01 |
| Q9EPK7 | 10 | 10 | 42,37 | 65246 | Xpo7 | 0 | 1,06 | 9,74E-01 |
| P52196 | 26 | 6 | 52,76 | 22117 | Tst | 0 | 0,80 | 4,98E-01 |
| Q9QYA2 | 17 | 5 | 30,63 | 53333 | Tomm40 | 0 | 0,88 | 8,10E-01 |
| Q9CQW2 | 35 | 5 | 121,3 | 67166 | Arl8b | 4 | 0,98 | 9,76E-01 |
| Q9CR51 | 24 | 3 | 50,48 | 66290 | Atp6v1g1 | 0 | 1,45 | 1,88E-01 |
| P70208 | 4 | 2 | 48,79 | 18846 | Plxna3 | 0 | 1,38 | 7,09E-01 |
| Q8C0M9 | 27 | 8 | 74,02 | 66514 | Asrgl1 | 0 | 1,16 | 8,33E-01 |
| Q99JY8 | 22 | 8 | 97,1 | 67916 | Plpp3 | 0 | 0,84 | 7,39E-01 |
| Q1RLL3 | 13 | 7 | 66,58 | 211232 | Cpne9 | 0 | 0,89 | 8,10E-01 |

| | | | | | | | | |
|---|---|---|---|---|---|---|---|---|
| Q9D4C9 | 26 | 6 | 74,49 | 74438 | Clvs1 | 2 | 1,17 | 8,25E-01 |
| Q9Z0V2 | 15 | 7 | 35,74 | 16508 | Kcnd2 | 0 | 0,86 | 7,77E-01 |
| Q8K2C9 | 17 | 7 | 74,54 | 57874 | Hacd3 | 0 | 0,95 | 9,56E-01 |
| Q91VE0 | 17 | 9 | 45,44 | 26569 | Slc27a4 | 1 | 0,82 | 6,42E-01 |
| Q9CZD3 | 15 | 10 | 35,12 | 353172 | Gars1 | 0 | 1,04 | 9,77E-01 |
| Q9CTY5 | 17 | 8 | 37,79 | 78506 | Micu3 | 0 | 0,81 | 5,84E-01 |
| Q50H33 | 17 | 5 | 61,49 | 243043 | Kctd8 | 0 | 0,83 | 8,43E-01 |
| P62071 | 32 | 4 | 54,06 | 66922 | Rras2 | 2 | 1,13 | 8,91E-01 |
| Q9JK48 | 26 | 8 | 61,26 | 54673 | Sh3glb1 | 0 | 1,16 | 8,45E-01 |
| Q9QYF9 | 23 | 6 | 75,22 | 29812 | Ndrg3 | 0 | 0,86 | 7,23E-01 |
| Q9WTX6 | 17 | 13 | 28,88 | 26965 | Cul1 | 0 | 0,96 | 9,70E-01 |
| Q9D898 | 45 | 6 | 80,53 | 74192 | Arpc5l | 0 | 0,92 | 9,22E-01 |
| Q8BK64 | 31 | 10 | 36,3 | 217737 | Ahsa1 | 0 | 1,23 | 6,63E-01 |
| Q3TDD9 | 15 | 8 | 15,93 | 73825 | Ppp1r21 | 0 | 1,08 | 9,74E-01 |
| Q3THS6 | 17 | 7 | 41,1 | 232087 | Mat2a | 0 | 1,21 | 8,01E-01 |
| Q3ULD5 | 15 | 7 | 57,42 | 78038 | Mccc2 | 0 | 0,71 | 2,72E-01 |
| Q61885 | 33 | 8 | 81,72 | 17441 | Mog | 0 | 0,84 | 7,18E-01 |
| Q8BGU5 | 28 | 8 | 41,72 | 67974 | Ccny | 0 | 1,04 | 9,79E-01 |
| P19536 | 36 | 6 | 86,69 | | Cox5b | 0 | 1,07 | 9,74E-01 |
| P35293 | 36 | 7 | 103,47 | 19330 | Rab18 | 0 | 1,10 | 9,40E-01 |
| P61089 | 46 | 8 | 70,17 | 93765 | Ube2n | 0 | 0,95 | 9,74E-01 |
| Q61151 | 21 | 6 | 59,45 | 26932 | Ppp2r5e | 2 | 1,09 | 9,56E-01 |
| Q8CC88 | 8 | 13 | 5,74 | 219189 | Vwa8 | 0 | 0,88 | 8,86E-01 |
| Q9DCL9 | 21 | 10 | 55,8 | 67054 | Paics | 0 | 0,99 | 9,77E-01 |
| P59016 | 20 | 11 | 22,64 | 233405 | Vps33b | 0 | 0,93 | 9,34E-01 |
| Q9D517 | 23 | 10 | 69,52 | 28169 | Agpat3 | 0 | 1,01 | 1,00E+00 |
| Q9Z127 | 10 | 6 | 87,49 | 20539 | Slc7a5 | 0 | 1,00 | 9,91E-01 |
| Q9QZQ1 | 8 | 12 | 13,69 | 17356 | Afdn | 0 | 0,98 | 9,85E-01 |
| P47740 | 18 | 7 | 27,68 | 11671 | Aldh3a2 | 0 | 0,97 | 9,76E-01 |
| P45952 | 19 | 8 | 81,11 | 11364 | Acadm | 0 | 0,82 | 6,07E-01 |
| Q9QXX4 | 12 | 3 | 138,55 | 50799 | Slc25a13 | 0 | 0,63 | 4,61E-01 |
| Q80UU9 | 38 | 5 | 42 | 70804 | Pgrmc2 | 0 | 1,10 | 9,48E-01 |
| Q80ZJ1 | 40 | 4 | 83,15 | 76108 | Rap2a | 0 | 1,06 | 9,74E-01 |
| Q8C166 | 15 | 8 | 63,27 | 266692 | Cpne1 | 1 | 1,00 | 9,87E-01 |
| P58771 | 23 | 8 | 65,2 | 22003 | Tpm1 | 0 | 1,33 | 4,00E-01 |
| Q3V1U8 | 19 | 4 | 53,68 | 270162 | Elmod1 | 0 | 0,84 | 8,25E-01 |
| Q8BLR2 | 16 | 7 | 56,07 | 74020 | Cpne4 | 0 | 1,28 | 6,58E-01 |
| Q7TNP2 | 10 | 5 | 62,1 | 73699 | Ppp2r1b | 0 | 0,79 | 4,06E-01 |
| Q9Z1W9 | 12 | 5 | 25,39 | 53416 | Stk39 | 1 | 1,31 | 4,72E-01 |
| P35546 | 12 | 12 | 13,62 | 19713 | Ret | 0 | 2,35 | 1,04E-04 |
| P03911 | 11 | 5 | 95,15 | 17719 | Mtnd4 | 0 | 0,81 | 6,15E-01 |
| O09061 | 28 | 6 | 49,9 | 19170 | Psmb1 | 0 | 1,13 | 9,05E-01 |
| P12849 | 17 | 4 | 51,22 | 19085 | Prkar1b | 0 | 0,99 | 9,91E-01 |
| Q9QYJ0 | 30 | 11 | 56,46 | 56445 | Dnaja2 | 0 | 1,20 | 7,86E-01 |
| Q3UTJ2 | 13 | 11 | 26,63 | 234214 | Sorbs2 | 0 | 1,04 | 9,79E-01 |
| P08226 | 32 | 9 | 51,32 | 11816 | Apoe | 0 | 0,92 | 9,18E-01 |
| Q9Z1R2 | 9 | 7 | 41,71 | 224727 | Bag6 | 0 | 1,21 | 7,89E-01 |

| | | | | | | | | |
|---|---|---|---|---|---|---|---|---|
| Q9CPQ8 | 33 | 2 | 20,99 | 27425 | Atp5mg | 0 | 0,87 | 8,10E-01 |
| O35864 | 24 | 7 | 76,02 | 26754 | Cops5 | 0 | 1,12 | 9,16E-01 |
| Q8BTG7 | 26 | 7 | 52,31 | 234593 | Ndrg4 | 0 | 0,91 | 8,97E-01 |
| Q9CZR3 | 16 | 5 | 72,76 | 641376 | Tomm40l | 0 | 0,65 | 4,79E-02 |
| Q9EST1 | 11 | 7 | 70 | 57911 | Gsdma | 0 | 0,96 | 9,71E-01 |
| P21271 | 3 | 7 | 137,74 | | Myo5b | 0 | 1,23 | 7,02E-01 |
| Q9CQS4 | 24 | 7 | 64,22 | 67453 | Slc25a46 | 0 | 0,81 | 5,77E-01 |
| Q9R1P3 | 34 | 6 | 55,69 | 26445 | Psmb2 | 0 | 1,43 | 2,18E-01 |
| O35639 | 35 | 9 | 27,17 | 11745 | Anxa3 | 1 | 0,39 | 1,45E-07 |
| Q8K3J1 | 37 | 8 | 72,25 | 225887 | Ndufs8 | 0 | 0,78 | 4,83E-01 |
| Q9JMH9 | 6 | 10 | 13,68 | 360013 | Myo18a | 0 | 0,90 | 8,91E-01 |
| Q9EPR5 | 10 | 12 | 31,55 | 81840 | Sorcs2 | 0 | 0,86 | 7,61E-01 |
| Q71M36 | 17 | 7 | 76,7 | 29873 | Cspg5 | 0 | 1,09 | 9,44E-01 |
| Q80U23 | 25 | 10 | 26,62 | 241727 | Snph | 0 | 0,99 | 9,76E-01 |
| P12023 | 17 | 9 | 25,73 | 11820 | App | 1 | 1,01 | 9,97E-01 |
| TRFL_HUMAN | 15 | 10 | 18,58 | 4057 | HEL110; LTF | 0 | 3,30 | 2,00E-13 |
| B1AZP2 | 12 | 8 | 35,66 | 228836 | Dlgap4 | 0 | 1,06 | 9,76E-01 |
| Q7TNC9 | 23 | 10 | 29,37 | 212111 | Inpp5a | 0 | 1,14 | 8,70E-01 |
| Q80YA9 | 11 | 8 | 34,43 | 245684 | Cnksr2 | 0 | 1,29 | 6,61E-01 |
| Q9DC70 | 18 | 5 | 80,33 | 75406 | Ndufs7 | 0 | 1,10 | 9,39E-01 |
| P40336 | 23 | 5 | 53,74 | 30930 | Vps26a | 0 | 1,15 | 8,78E-01 |
| Q80TL0 | 9 | 5 | 42,21 | 320472 | Ppm1e | 0 | 0,96 | 9,74E-01 |
| P56695 | 12 | 8 | 29,52 | 22393 | Wfs1 | 0 | 0,98 | 9,78E-01 |
| Q0VE82 | 19 | 9 | 49,19 | 102278 | Cpne7 | 0 | 1,72 | 1,07E-02 |
| O89112 | 15 | 6 | 89,56 | 14768 | Lancl1 | 0 | 0,90 | 8,93E-01 |
| Q99P58 | 43 | 8 | 55,43 | 80718 | Rab27b | 0 | 1,11 | 9,34E-01 |
| Q922B2 | 20 | 9 | 34,46 | 226414 | Dars1 | 0 | 0,82 | 6,33E-01 |
| Q920M7 | 26 | 10 | 34,98 | 110058 | Syt17 | 0 | 2,06 | 6,37E-04 |
| Q62425 | 48 | 6 | 101,79 | 17992 | Ndufa4 | 0 | 0,73 | 2,39E-01 |
| Q811P8 | 6 | 10 | 26,58 | 330914 | Arhgap32 | 1 | 1,13 | 9,09E-01 |
| Q8BHF7 | 16 | 7 | 40,36 | 74451 | Pgs1 | 0 | 0,96 | 9,64E-01 |
| Q6P5F7 | 15 | 7 | 54,68 | 78339 | Ttyh3 | 0 | 0,92 | 9,27E-01 |
| A2A5R2 | 8 | 8 | 19,29 | 99371 | Arfgef2 | 5 | 1,09 | 9,65E-01 |
| Q64010 | 50 | 11 | 72,61 | 12928 | Crk | 0 | 0,93 | 9,23E-01 |
| Q8BHL5 | 13 | 5 | 20,08 | 140579 | Elmo2 | 0 | 1,15 | 8,91E-01 |
| P28271 | 10 | 7 | 37,97 | 11428 | Aco1 | 0 | 0,96 | 9,74E-01 |
| Q3UH60 | 8 | 11 | 30,86 | 239667 | Dip2b | 0 | 1,12 | 9,16E-01 |
| Q99J47 | 23 | 8 | 22,17 | 216820 | Dhrs7b | 0 | 0,85 | 8,95E-01 |
| P27612 | 12 | 8 | 23,29 | 18786 | Plaa | 0 | 0,96 | 9,76E-01 |
| Q99JY0 | 20 | 9 | 48,72 | 231086 | Hadhb | 0 | 0,75 | 2,56E-01 |
| Q9R0P5 | 32 | 4 | 112,78 | 56431 | Dstn | 0 | 1,01 | 9,99E-01 |
| Q8CIG8 | 18 | 11 | 32,77 | 27374 | Prmt5 | 0 | 1,07 | 9,76E-01 |
| P63137 | 19 | 4 | 43,81 | 14401 | Gabrb2 | 1 | 0,98 | 9,85E-01 |
| O35682 | 19 | 4 | 90,09 | 50918 | Myadm | 0 | 1,12 | 9,03E-01 |
| Q62283 | 18 | 4 | 85,59 | 21912 | Tspan7 | 0 | 1,01 | 1,00E+00 |
| Q9DCT8 | 34 | 3 | 34,87 | 68337 | Crip2 | 0 | 1,25 | 6,86E-01 |

| | | | | | | | | |
|---|---|---|---|---|---|---|---|---|
| Q9D0L7 | 31 | 7 | 53,03 | 67211 | Armc10 | 0 | 0,93 | 9,32E-01 |
| Q5SSM3 | 9 | 7 | 36,92 | 216831 | Arhgap44 | 0 | 1,39 | 4,42E-01 |
| Q9Z2C4 | 14 | 9 | 48,19 | 53332 | Mtmr1 | 0 | 0,93 | 9,21E-01 |
| P54822 | 20 | 7 | 45,41 | 11564 | Adsl | 0 | 1,03 | 9,96E-01 |
| Q80XI4 | 15 | 3 | 52,66 | 108083 | Pip4k2b | 0 | 1,06 | 9,76E-01 |
| Q61831 | 17 | 2 | 56,95 | 26414 | Mapk10 | 6 | 1,07 | 9,67E-01 |
| P55288 | 14 | 8 | 29,47 | 12552 | Cdh11 | 0 | 1,02 | 1,00E+00 |
| Q9QXW9 | 11 | 6 | 69,25 | 50934 | Slc7a8 | 0 | 0,94 | 9,42E-01 |
| GELS_HUMAN | 12 | 3 | 40,95 | 2934 | GSN | 0 | 0,37 | 4,63E-03 |
| Q8BNU0 | 25 | 8 | 46,14 | 76813 | Armc6 | 0 | 1,40 | 3,35E-01 |
| Q99MX7 | 18 | 6 | 32,98 | 94047 | Tmem121b | 0 | 0,88 | 8,23E-01 |
| Q922Q1 | 23 | 9 | 76,97 | 67247 | Mtarc2 | 0 | 0,81 | 6,36E-01 |
| O70252 | 23 | 7 | 32,43 | 15369 | Hmox2 | 0 | 1,02 | 1,00E+00 |
| Q9CPU4 | 33 | 4 | 61,46 | 66447 | Mgst3 | 0 | 0,75 | 3,03E-01 |
| Q7TPB0 | 12 | 7 | 38,47 | 216152 | Plppr3 | 0 | 0,83 | 8,11E-01 |
| P22723 | 13 | 7 | 39,89 | 14406 | Gabrg2 | 0 | 1,42 | 2,28E-01 |
| Q8BPU7 | 13 | 7 | 15,84 | 140580 | Elmo1 | 1 | 0,97 | 9,76E-01 |
| Q9CQ54 | 35 | 6 | 78,27 | 68197 | Ndufc2 | 0 | 0,76 | 3,68E-01 |
| Q63810 | 25 | 5 | 107,25 | 19058 | Ppp3r1 | 0 | 1,43 | 1,46E-01 |
| Q9D2N4 | 11 | 7 | 45,78 | 13527 | Dtna | 0 | 1,06 | 9,76E-01 |
| P50171 | 31 | 6 | 42,92 | 14979 | Hsd17b8 | 0 | 0,65 | 2,28E-01 |
| O70503 | 30 | 8 | 34,76 | 56348 | Hsd17b12 | 0 | 1,09 | 9,56E-01 |
| O54833 | 20 | 6 | 31,26 | 13000 | Csnk2a2 | 0 | 1,22 | 7,73E-01 |
| Q9R1P0 | 28 | 8 | 82,46 | 26441 | Psma4 | 0 | 1,17 | 8,01E-01 |
| Q9WV69 | 26 | 9 | 49,24 | 13829 | Dmtn | 0 | 1,09 | 9,59E-01 |
| O35295 | 23 | 6 | 53,38 | 19291 | Purb | 0 | 0,96 | 9,74E-01 |
| Q32MW3 | 18 | 9 | 61,39 | 64833 | Acot10 | 0 | 1,05 | 9,76E-01 |
| Q9JIF0 | 23 | 6 | 47,23 | 15469 | Prmt1 | 3 | 0,93 | 9,34E-01 |
| P29533 | 14 | 8 | 24,46 | | Vcam1 | 0 | 1,18 | 8,09E-01 |
| Q9CYH2 | 35 | 8 | 59,02 | 70564 | Prxl2a | 0 | 0,93 | 9,32E-01 |
| Q6KAR6 | 11 | 7 | 41,79 | 211446 | Exoc3 | 0 | 0,89 | 8,42E-01 |
| P20917 | 14 | 6 | 34,08 | 17136 | Mag | 0 | 0,96 | 9,71E-01 |
| P50571 | 20 | 3 | 23,65 | 14400 | Gabrb1 | 0 | 0,94 | 9,48E-01 |
| Q8K354 | 37 | 7 | 68,82 | 109857 | Cbr3 | 0 | 0,93 | 9,36E-01 |
| Q6VNS1 | 8 | 3 | 62,35 | 18213 | Ntrk3 | 0 | 0,97 | 9,76E-01 |
| Q8VDP6 | 22 | 4 | 52,61 | 52858 | Cdipt | 0 | 1,13 | 9,02E-01 |
| P63168 | 40 | 2 | 64,36 | 56455 | Dynll1 | 0 | 1,59 | 4,46E-01 |
| Q99J99 | 27 | 7 | 43 | 246221 | Mpst | 0 | 0,82 | 6,29E-01 |
| Q8BY89 | 11 | 7 | 74,02 | 68682 | Slc44a2 | 0 | 0,78 | 4,04E-01 |
| Q3UIU2 | 36 | 5 | 49,68 | 230075 | Ndufb6 | 0 | 0,76 | 3,81E-01 |
| Q80WG5 | 13 | 10 | 34,29 | 241296 | Lrrc8a | 1 | 0,82 | 6,52E-01 |
| Q9QUP5 | 24 | 9 | 66,93 | 12950 | Hapln1 | 0 | 1,12 | 9,23E-01 |
| P15209 | 11 | 4 | 23,19 | 18212 | Ntrk2 | 4 | 1,03 | 9,91E-01 |
| Q8BTX9 | 15 | 5 | 72,75 | 72552 | Hsdl1 | 0 | 1,12 | 9,16E-01 |
| Q8BJU0 | 27 | 7 | 56,03 | 52551 | Sgta | 0 | 0,90 | 8,85E-01 |
| B0F2B4 | 8 | 3 | 62,22 | | Nlgn4l | 0 | 1,04 | 9,97E-01 |

| | | | | | | | | |
|---|---|---|---|---|---|---|---|---|
| Q6P5F9 | 8 | 7 | 19,5 | 103573 | Xpo1 | 0 | 1,01 | 1,00E+00 |
| Q8VCW8 | 13 | 7 | 41,55 | 264895 | Acsf2 | 0 | 0,67 | 2,91E-01 |
| Q8VEH5 | 10 | 5 | 32,97 | 77781 | Epm2aip1 | 0 | 1,10 | 9,65E-01 |
| Q9D5T0 | 25 | 7 | 22,11 | 67979 | Atad1 | 0 | 1,04 | 9,85E-01 |
| Q8R0S4 | 17 | 3 | 47,36 | 12298 | Cacnb4 | 0 | 0,86 | 9,16E-01 |
| Q6PGE7 | 8 | 6 | 74,88 | 240332 | Slc6a7 | 0 | 1,02 | 9,91E-01 |
| Q9Z2U1 | 24 | 5 | 87,5 | 26442 | Psma5 | 0 | 1,05 | 9,76E-01 |
| P24369 | 35 | 7 | 33,61 | 19035 | Ppib | 0 | 1,13 | 9,19E-01 |
| P63037 | 19 | 6 | 36,67 | 15502 | Dnaja1 | 0 | 1,23 | 6,51E-01 |
| O70456 | 19 | 2 | 270,49 | 55948 | Sfn | 0 | 1,14 | 9,71E-01 |
| Q9WTQ5 | 8 | 9 | 6,68 | 83397 | Akap12 | 0 | 1,20 | 8,91E-01 |
| Q06890 | 19 | 9 | 41,79 | 12759 | Clu | 0 | 0,96 | 9,74E-01 |
| P62743 | 36 | 5 | 74,95 | 232910 | Ap2s1 | 0 | 1,16 | 8,33E-01 |
| P55302 | 26 | 9 | 30,63 | 16976 | Lrpap1 | 0 | 0,79 | 5,68E-01 |
| Q0VBF8 | 21 | 2 | 102,41 | 381310 | Stum | 0 | 0,92 | 8,93E-01 |
| Q9CR26 | 20 | 5 | 39,25 | 66201 | Vta1 | 0 | 1,07 | 9,74E-01 |
| Q920P3 | 12 | 9 | 37,15 | 56710 | Brinp1 | 0 | 0,98 | 9,78E-01 |
| Q9D6F4 | 22 | 9 | 26,76 | 14397 | Gabra4 | 0 | 0,90 | 8,89E-01 |
| Q64331 | 8 | 8 | 29,75 | | Myo6 | 0 | 1,16 | 8,54E-01 |
| Q6GQT9 | 9 | 9 | 20,11 | 211548 | Nomo1 | 0 | 0,66 | 1,46E-01 |
| Q9DBT5 | 11 | 9 | 40,12 | 109674 | Ampd2 | 0 | 1,11 | 9,12E-01 |
| Q99LR1 | 15 | 6 | 46,61 | 76192 | Abhd12 | 0 | 1,12 | 9,34E-01 |
| Q8R550 | 17 | 10 | 15,5 | 58194 | Sh3kbp1 | 0 | 1,08 | 9,61E-01 |
| P26048 | 14 | 3 | 54,61 | 14395 | Gabra2 | 0 | 0,81 | 7,18E-01 |
| P54728 | 24 | 10 | 47,76 | 19359 | Rad23b | 0 | 0,91 | 9,09E-01 |
| Q9CZY3 | 41 | 6 | 96,35 | 66589 | Ube2v1 | 0 | 1,01 | 1,00E+00 |
| P28571 | 9 | 5 | 54,23 | 14664 | Slc6a9 | 0 | 0,77 | 4,24E-01 |
| Q9CRB6 | 35 | 7 | 62,58 | 67971 | Tppp3 | 0 | 1,17 | 8,44E-01 |
| A2ASZ8 | 16 | 5 | 56,96 | 227731 | Slc25a25 | 0 | 0,79 | 5,02E-01 |
| Q3UEZ8 | 5 | 3 | 71,88 | 231290 | Slc10a4 | 0 | 2,47 | 4,67E-08 |
| Q9JJV2 | 36 | 5 | 69,76 | 18645 | Pfn2 | 0 | 1,35 | 3,68E-01 |
| Q9CR61 | 45 | 5 | 39,11 | 66916 | Ndufb7 | 0 | 1,07 | 9,74E-01 |
| Q91ZU6 | 2 | 11 | 1,66 | 13518 | Dst | 0 | 0,84 | 8,85E-01 |
| Q5PR73 | 41 | 6 | 85,42 | 68203 | Diras2 | 0 | 1,05 | 9,78E-01 |
| P11404 | 61 | 8 | 84,86 | 14077 | Fabp3 | 0 | 1,18 | 7,74E-01 |
| Q9D1G5 | 38 | 9 | 28,95 | | Lrrc57 | 0 | 0,98 | 9,76E-01 |
| Q6PDL0 | 17 | 7 | 40,48 | 234663 | Dync1li2 | 0 | 1,03 | 9,97E-01 |
| Q922Q4 | 32 | 7 | 37,62 | 69051 | Pycr2 | 0 | 0,94 | 9,56E-01 |
| Q8C078 | 20 | 10 | 26,95 | 207565 | Camkk2 | 0 | 1,00 | 9,87E-01 |
| Q06138 | 24 | 8 | 30,81 | 12283 | Cab39 | 0 | 1,21 | 7,39E-01 |
| Q6NS60 | 12 | 10 | 50,74 | 330369 | Fbxo41 | 0 | 1,14 | 8,91E-01 |
| P47934 | 13 | 7 | 35,35 | 12908 | Crat | 0 | 0,95 | 9,64E-01 |
| P60766 | 31 | 4 | 78,36 | 12540 | Cdc42 | 0 | 1,64 | 2,22E-02 |
| O88543 | 14 | 5 | 61,71 | 26572 | Cops3 | 0 | 1,05 | 9,78E-01 |
| P47199 | 31 | 7 | 39,72 | 12972 | Cryz | 0 | 0,71 | 1,72E-01 |
| A2A690 | 5 | 11 | 3,95 | 77097 | Tanc2 | 0 | 1,08 | 9,65E-01 |
| Q99KH8 | 21 | 4 | 39,57 | 223255 | Stk24 | 4 | 1,32 | 4,82E-01 |

| | | | | | | | | |
|---|---|---|---|---|---|---|---|---|
| Q8VHJ5 | 11 | 5 | 32,59 | 226778 | Mark1 | 0 | 0,83 | 8,11E-01 |
| P48193 | 13 | 5 | 11,84 | 269587 | Epb41 | 0 | 0,87 | 9,32E-01 |
| O54901 | 16 | 4 | 100,33 | | Cd200 | 0 | 1,56 | 4,81E-02 |
| Q61735 | 18 | 5 | 110,14 | 16423 | Cd47 | 0 | 0,89 | 8,58E-01 |
| Q7TSQ8 | 10 | 9 | 31,53 | 319518 | Pdpr | 0 | 0,75 | 3,18E-01 |
| Q8BH44 | 17 | 9 | 28,45 | 235431 | Coro2b | 0 | 1,21 | 7,61E-01 |
| Q9D892 | 23 | 5 | 60,07 | 16434 | Itpa | 0 | 1,08 | 9,67E-01 |
| Q9D819 | 23 | 8 | 69,4 | 67895 | Ppa1 | 0 | 1,08 | 9,67E-01 |
| P84084 | 34 | 4 | 106,38 | 11844 | Arf5 | 0 | 1,13 | 9,02E-01 |
| Q9R257 | 26 | 4 | 27,44 | 15199 | Hebp1 | 0 | 0,89 | 9,34E-01 |
| Q920E5 | 24 | 7 | 42,9 | 110196 | Fdps | 0 | 1,40 | 2,39E-01 |
| P55066 | 7 | 10 | 93,03 | 13004 | Ncan | 0 | 0,99 | 9,81E-01 |
| Q9CQZ5 | 54 | 6 | 75,36 | 67130 | Ndufa6 | 0 | 0,98 | 9,76E-01 |
| Q9JI18 | 2 | 9 | 14,88 | | Lrp1b | 0 | 1,11 | 9,76E-01 |
| Q80UW2 | 24 | 5 | 68,94 | 230904 | Fbxo2 | 0 | 0,81 | 7,33E-01 |
| P60764 | 33 | 2 | 99,93 | 170758 | Rac3 | 0 | 1,62 | 1,94E-01 |
| Q9JIW9 | 27 | 4 | 47,7 | 64143 | Ralb | 0 | 1,22 | 9,23E-01 |
| Q8CHU3 | 16 | 7 | 24,35 | 13855 | Epn2 | 0 | 0,78 | 6,82E-01 |
| Q9DBE8 | 15 | 5 | 23,69 | 56737 | Alg2 | 0 | 0,79 | 6,58E-01 |
| Q3UH66 | 6 | 8 | 26,3 | 75607 | Wnk2 | 2 | 1,01 | 9,97E-01 |
| Q791T5 | 21 | 7 | 58,57 | 56462 | Mtch1 | 0 | 0,73 | 2,19E-01 |
| Q9Z0J4 | 7 | 8 | 31,58 | 18125 | Nos1 | 0 | 0,81 | 6,07E-01 |
| F8VPU2 | 8 | 7 | 42,05 | 223254 | Farp1 | 0 | 0,74 | 3,02E-01 |
| Q99KE1 | 15 | 5 | 54,62 | 107029 | Me2 | 0 | 0,92 | 9,56E-01 |
| Q8K097 | 7 | 2 | 58,23 | 72393 | Faim2 | 0 | 1,00 | 9,96E-01 |
| O35963 | 16 | 2 | 187,52 | 19338 | Rab33b | 0 | 1,16 | 8,91E-01 |
| Q61035 | 18 | 2 | 58,57 | 15115 | Hars1 | 7 | 1,45 | 1,61E-01 |
| Q9WTR5 | 11 | 5 | 48,28 | 12554 | Cdh13 | 0 | 0,88 | 8,07E-01 |
| Q80VL1 | 19 | 8 | 22,77 | 72634 | Tdrkh | 0 | 0,71 | 3,52E-01 |
| Q9D8U8 | 19 | 8 | 45,11 | 69178 | Snx5 | 0 | 0,88 | 7,86E-01 |
| D3YWQ0 | 8 | 6 | 26,31 | 320127 | Dgki | 0 | 1,07 | 9,76E-01 |
| Q8BH55 | 11 | 7 | 24,02 | 208967 | Thnsl1 | 0 | 0,78 | 5,84E-01 |
| P55088 | 20 | 5 | 46,45 | 11829 | Aqp4 | 0 | 0,70 | 1,28E-01 |
| P70182 | 5 | 3 | 48,71 | 18720 | Pip5k1a | 0 | 1,06 | 9,76E-01 |
| P47746 | 10 | 6 | 52,94 | 12801 | Cnr1 | 0 | 0,79 | 5,42E-01 |
| P63046 | 20 | 5 | 30,14 | 29859 | Sult4a1 | 0 | 1,19 | 8,33E-01 |
| Q8BJY1 | 14 | 7 | 47,08 | 66998 | Psmd5 | 0 | 0,97 | 9,74E-01 |
| P62827 | 32 | 7 | 106,44 | 19384 | Ran | 0 | 1,13 | 8,85E-01 |
| P04370 | 18 | 5 | 67,5 | 17196 | Mbp | 0 | 0,68 | 8,48E-02 |
| P02088 | 45 | 4 | 84,12 | 100503605; 101488143; 15129 | Hbb-b1 | 0 | 1,27 | 5,85E-01 |
| P47809 | 18 | 6 | 63,52 | 26398 | Map2k4 | 0 | 1,29 | 5,46E-01 |
| Q9WTX5 | 39 | 6 | 39,21 | 21402 | Skp1 | 0 | 1,11 | 9,56E-01 |
| Q9JHK4 | 17 | 10 | 32,89 | 56187 | Rabggta | 0 | 0,73 | 1,94E-01 |
| Q9D7B6 | 23 | 9 | 18,72 | 66948 | Acad8 | 0 | 1,16 | 8,89E-01 |

| | | | | | | | | |
|---|---|---|---|---|---|---|---|---|
| P97315 | 30 | 5 | 47,69 | 13007 | Csrp1 | 0 | 0,76 | 3,87E-01 |
| P09528 | 42 | 8 | 51,88 | 14319 | Fth1 | 0 | 1,18 | 7,50E-01 |
| Q64433 | 65 | 7 | 61,91 | 15528 | Hspe1 | 0 | 1,15 | 8,43E-01 |
| Q69Z98 | 13 | 5 | 30,31 | 75770 | Brsk2 | 2 | 1,23 | 7,46E-01 |
| P12787 | 27 | 5 | 82,04 | 12858 | Cox5a | 0 | 0,82 | 6,74E-01 |
| Q3THK7 | 13 | 7 | 22,17 | 229363 | Gmps | 0 | 1,01 | 1,00E+00 |
| Q6I6G8 | 7 | 9 | 7,99 | 329152 | Hecw2 | 0 | 1,36 | 6,27E-01 |
| Q9ERB0 | 35 | 7 | 23,03 | 67474 | Snap29 | 0 | 1,17 | 8,25E-01 |
| P84086 | 31 | 2 | 34,39 | 12890 | Cplx2 | 4 | 0,95 | 9,70E-01 |
| Q3TY60 | 17 | 5 | 27,51 | 76156 | Fam131b | 0 | 0,95 | 9,74E-01 |
| Q0VBK2 | 12 | 6 | 104,39 | 74127 | Krt80 | 0 | 1,10 | 9,48E-01 |
| Q8K0T7 | 2 | 6 | 20,74 | 208898 | Unc13c | 0 | 1,27 | 6,42E-01 |
| Q8R574 | 25 | 6 | 28,65 | 212627 | Prpsap2 | 1 | 0,85 | 7,09E-01 |
| Q8C031 | 13 | 6 | 28,72 | 241568 | Lrrc4c | 0 | 1,11 | 9,41E-01 |
| Q99PL6 | 22 | 9 | 58,49 | 66530 | Ubxn6 | 0 | 1,10 | 9,34E-01 |
| Q8BGZ1 | 32 | 3 | 44,56 | 170638 | Hpcal4 | 0 | 1,10 | 9,64E-01 |
| Q01063 | 9 | 4 | 38,08 | 238871 | Pde4d | 2 | 1,63 | 4,81E-02 |
| Q3UHH2 | 11 | 6 | 45,8 | 73102 | Slc22a23 | 0 | 1,00 | 1,00E+00 |
| O35449 | 12 | 3 | 65,59 | 260297 | Prrt1 | 0 | 0,96 | 9,72E-01 |
| O88746 | 16 | 5 | 45,8 | 21968 | Tom1 | 0 | 1,12 | 9,34E-01 |
| P42125 | 26 | 6 | 16,33 | 13177 | Eci1 | 0 | 0,79 | 5,32E-01 |
| B1AXV0 | 22 | 8 | 43,14 | 230235 | Frrs1l | 0 | 0,91 | 8,85E-01 |
| Q5DTL9 | 9 | 8 | 15,53 | 94229 | Slc4a10 | 0 | 0,95 | 9,76E-01 |
| Q61627 | 10 | 7 | 31,17 | 14803 | Grid1 | 1 | 0,65 | 2,15E-01 |
| Q9Z0P5 | 15 | 3 | 35,23 | 23999 | Twf2 | 0 | 1,02 | 1,00E+00 |
| Q9WUM3 | 18 | 8 | 40,48 | 23789 | Coro1b | 0 | 1,03 | 9,94E-01 |
| Q9CQ60 | 31 | 7 | 20,59 | 66171 | Pgls | 0 | 1,01 | 1,00E+00 |
| P56391 | 80 | 8 | 62,26 | 110323 | Cox6b1 | 0 | 0,75 | 3,29E-01 |
| Q8BVQ5 | 17 | 6 | 46,67 | 72590 | Ppme1 | 0 | 1,18 | 8,49E-01 |
| Q9D0M1 | 25 | 5 | 32,47 | 67763 | Prpsap1 | 0 | 1,14 | 9,16E-01 |
| Q8CE50 | 20 | 8 | 22,12 | 209131 | Snx30 | 0 | 0,92 | 9,30E-01 |
| Q8R3Q2 | 9 | 5 | 19,93 | | Ppp6r2 | 0 | 1,30 | 8,51E-01 |
| P22892 | 10 | 10 | 59,72 | 11765 | Ap1g1 | 0 | 0,99 | 9,83E-01 |
| P57776 | 21 | 4 | 34,35 | 66656 | Eef1d | 0 | 1,06 | 9,78E-01 |
| P18242 | 19 | 5 | 34 | 13033 | Ctsd | 0 | 0,64 | 2,37E-02 |
| P97370 | 24 | 6 | 58,98 | 11933 | Atp1b3 | 0 | 1,07 | 9,71E-01 |
| Q8CJH3 | 5 | 8 | 52,46 | 235611 | Plxnb1 | 1 | 0,89 | 8,62E-01 |
| CAS2_BOVIN | 31 | 7 | 36,74 | 109560546; 282209 | CSN1S2; LOC109560546; LOC113894643 | 0 | 1,08 | 9,60E-01 |
| Q91YE6 | 7 | 5 | 36,2 | | Ipo9 | 0 | 0,95 | 9,76E-01 |
| P14869 | 25 | 6 | 29,93 | 11837 | Rplp0 | 0 | 0,97 | 9,76E-01 |
| Q91YP2 | 15 | 8 | 30,93 | 75805 | Nln | 0 | 0,90 | 8,86E-01 |
| Q9CPQ1 | 63 | 7 | 81,91 | 12864 | Cox6c | 0 | 1,13 | 8,86E-01 |
| THIO_HUMAN | 35 | 2 | 83,08 | | | 2 | 1,23 | 6,58E-01 |

| | | | | | | | | |
|---|---|---|---|---|---|---|---|---|
| Q9CZN4 | 15 | 5 | 44,49 | 72555 | Shisa9 | 0 | 1,12 | 9,25E-01 |
| P70408 | 10 | 6 | 33,21 | 320873 | Cdh10 | 1 | 0,91 | 9,50E-01 |
| Q9CWH6 | 16 | 3 | 79,8 | 73677 | Psma8 | 0 | 1,27 | 5,84E-01 |
| Q8BLJ3 | 17 | 4 | 20,17 | 239318 | Plcxd3 | 0 | 2,16 | 2,11E-02 |
| Q9Z1P6 | 64 | 7 | 38,26 | 66416 | Ndufa7 | 0 | 0,94 | 9,34E-01 |
| Q6PD03 | 15 | 5 | 25,13 | 226849 | Ppp2r5a | 0 | 1,02 | 1,00E+00 |
| Q7TN29 | 21 | 6 | 57,8 | 69780 | Smap2 | 1 | 0,97 | 9,74E-01 |
| O35681 | 12 | 6 | 49,49 | 20981 | Syt3 | 0 | 0,94 | 9,56E-01 |
| Q9CQ62 | 16 | 4 | 66,33 | 67460 | Decr1 | 0 | 0,95 | 9,56E-01 |
| Q8BFZ9 | 20 | 4 | 47,69 | 244373 | Erlin2 | 1 | 1,06 | 9,74E-01 |
| Q91Y86 | 20 | 3 | 28,35 | 26419 | Mapk8 | 1 | 1,02 | 1,00E+00 |
| Q9CXZ1 | 31 | 5 | 42,99 | 17993 | Ndufs4 | 0 | 0,82 | 6,27E-01 |
| Q9WUK2 | 24 | 4 | 49,92 | 22384 | Eif4h | 0 | 0,84 | 7,52E-01 |
| O88441 | 16 | 5 | 46,28 | 53375 | Mtx2 | 0 | 0,78 | 4,01E-01 |
| P26049 | 16 | 5 | 33,64 | 14396 | Gabra3 | 0 | 0,48 | 4,21E-03 |
| Q9Z329 | 3 | 10 | 3,71 | 16439 | Itpr2 | 0 | 0,99 | 9,80E-01 |
| Q62083 | 16 | 6 | 33,66 | | Pick1 | 0 | 1,07 | 9,74E-01 |
| Q920Q4 | 9 | 7 | 48,67 | | Vps16 | 0 | 1,01 | 1,00E+00 |
| Q8VBV7 | 30 | 4 | 45,46 | 108679 | Cops8 | 0 | 1,06 | 9,76E-01 |
| Q3TLI0 | 6 | 7 | 14,03 | | | 0 | 0,79 | 4,61E-01 |
| Q9CYZ2 | 29 | 5 | 40,8 | 66314 | Tpd52l2 | 0 | 1,36 | 3,52E-01 |
| Q6A065 | 5 | 5 | 10,79 | 545389 | Cep170 | 2 | 1,24 | 8,43E-01 |
| Q60575 | 4 | 7 | 39,78 | 16561 | Kif1b | 0 | 1,01 | 1,00E+00 |
| Q9EQ80 | 25 | 7 | 27,23 | 65102 | Nif3l1 | 0 | 1,09 | 9,64E-01 |
| Q9R1Q9 | 7 | 5 | 79,52 | 54411 | Atp6ap1 | 0 | 1,13 | 8,85E-01 |
| P20352 | 20 | 5 | 30,23 | 14066 | F3 | 0 | 1,05 | 9,83E-01 |
| P53395 | 19 | 9 | 32,7 | 13171 | Dbt | 0 | 0,78 | 4,63E-01 |
| Q9CQ92 | 24 | 4 | 33,41 | 66437 | Fis1 | 0 | 2,12 | 1,14E-04 |
| Q69Z26 | 7 | 6 | 32,38 | 269784 | Cntn4 | 0 | 0,93 | 9,42E-01 |
| Q91YI0 | 18 | 7 | 11,49 | 109900 | Asl | 0 | 0,83 | 6,90E-01 |
| Q3UHK1 | 11 | 7 | 11,97 | 239606 | Slc2a13 | 0 | 1,30 | 5,42E-01 |
| P70181 | 4 | 2 | 33,2 | 18719 | Pip5k1b | 0 | 0,94 | 9,39E-01 |
| Q8K012 | 14 | 8 | 18,59 | | Fnbp1l | 0 | 0,97 | 9,77E-01 |
| Q6NVG1 | 12 | 5 | 33,22 | 99010 | Lpcat4 | 0 | 0,93 | 9,74E-01 |
| P84096 | 26 | 2 | 41,67 | 56212 | Rhog | 0 | 0,75 | 7,38E-01 |
| P52760 | 42 | 5 | 40,07 | 15473 | Rida | 0 | 1,08 | 9,63E-01 |
| Q91YP0 | 16 | 7 | 35,7 | 217666 | L2hgdh | 0 | 0,83 | 8,65E-01 |
| Q9DCP2 | 11 | 5 | 59,69 | 76257 | Slc38a3 | 0 | 1,20 | 7,49E-01 |
| Q923S9 | 32 | 4 | 157,21 | 75985 | Rab30 | 0 | 0,86 | 8,85E-01 |
| Q99L04 | 22 | 7 | 59,99 | 52585 | Dhrs1 | 0 | 0,79 | 4,98E-01 |
| P11835 | 10 | 6 | 14,21 | 16414 | Itgb2 | 0 | 0,40 | 1,06E-02 |
| Q99P47 | 7 | 9 | 13,37 | 170571 | Cntnap4 | 0 | 1,29 | 7,99E-01 |
| Q9DCR2 | 34 | 6 | 20,86 | 11777 | Ap3s1 | 0 | 1,22 | 7,93E-01 |
| Q8R317 | 17 | 5 | 19,57 | 56085 | Ubqln1 | 0 | 1,15 | 9,64E-01 |
| Q8C7X2 | 8 | 6 | 31,36 | 230866 | Emc1 | 0 | 0,84 | 6,92E-01 |
| P14206 | 22 | 5 | 42,59 | 16785 | Rpsa | 0 | 1,17 | 8,86E-01 |
| P97445 | 3 | 6 | 34,32 | 12286 | Cacna1a | 0 | 1,02 | 1,00E+00 |

| | | | | | | | | |
|---|---|---|---|---|---|---|---|---|
| Q8BVI5 | 19 | 5 | 15,77 | 228960 | Stx16 | 0 | 0,95 | 9,76E-01 |
| Q99K10 | 6 | 2 | 78,69 | 192167 | Nlgn1 | 0 | 1,07 | 9,74E-01 |
| Q6NVE9 | 21 | 5 | 15,97 | 320717 | Pptc7 | 0 | 0,62 | 2,75E-01 |
| Q6PAK3 | 16 | 4 | 43,11 | 381813 | Prmt8 | 0 | 1,01 | 9,96E-01 |
| Q9CR00 | 30 | 6 | 31,67 | 67151 | Psmd9 | 0 | 1,26 | 5,86E-01 |
| Q6NVE8 | 10 | 7 | 16,11 | 72404 | Wdr44 | 0 | 1,36 | 4,13E-01 |
| P43406 | 10 | 9 | 8,51 | 16410 | Itgav | 0 | 1,04 | 9,85E-01 |
| Q8BW96 | 22 | 3 | 27,4 | 227541 | Camk1d | 4 | 1,22 | 8,40E-01 |
| Q8C522 | 12 | 5 | 34,22 | 71946 | Endod1 | 0 | 1,14 | 8,95E-01 |
| P34152 | 7 | 5 | 32,08 | 14083 | Ptk2 | 0 | 0,96 | 9,76E-01 |
| P57759 | 27 | 6 | 47,34 | 67397 | Erp29 | 0 | 0,88 | 8,25E-01 |
| P35436 | 6 | 8 | 9,41 | 14811 | Grin2a | 0 | 1,16 | 8,45E-01 |
| Q9D0L4 | 13 | 5 | 13,56 | 72113 | Adck1 | 0 | 0,77 | 6,46E-01 |
| Q640R3 | 16 | 5 | 36,9 | 72927 | Hepacam | 0 | 1,17 | 8,53E-01 |
| Q7TSF0 | 4 | 5 | 76,27 | 211924 | Dsg1c | 0 | 0,91 | 9,15E-01 |
| Q9WV31 | 15 | 5 | 43,07 | 11838 | Arc | 0 | 0,95 | 9,70E-01 |
| Q5M8N4 | 22 | 6 | 39,01 | 654795 | Sdr39u1 | 0 | 1,07 | 9,76E-01 |
| P03888 | 11 | 4 | 39,8 | 17716; 3338902 | Mtnd1 | 0 | 0,92 | 9,09E-01 |
| Q9Z0Y1 | 38 | 9 | 78,96 | 53598 | Dctn3 | 0 | 1,23 | 6,42E-01 |
| P28474 | 19 | 6 | 39,59 | 11532 | Adh5 | 0 | 1,12 | 9,17E-01 |
| P16332 | 10 | 5 | 17,19 | 17850 | Mmut | 0 | 1,10 | 9,32E-01 |
| P56135 | 38 | 3 | 53,62 | 57423 | Atp5mf | 0 | 0,77 | 4,31E-01 |
| Q8JZZ7 | 4 | 3 | 19,77 | | Adgrl2 | 0 | 1,04 | 1,00E+00 |
| Q9JI46 | 36 | 4 | 37,03 | 56409 | Nudt3 | 1 | 0,99 | 9,85E-01 |
| Q8CHT1 | 11 | 7 | 35,9 | 53972 | Ngef | 0 | 1,18 | 7,99E-01 |
| Q9DB75 | 18 | 3 | 50,31 | 66626 | Cdip1 | 0 | 0,84 | 6,61E-01 |
| P18052 | 8 | 5 | 27,64 | 19262 | Ptpra | 1 | 0,73 | 3,76E-01 |
| Q9JMH6 | 11 | 6 | 46,45 | 50493 | Txnrd1 | 0 | 1,26 | 6,58E-01 |
| Q62433 | 16 | 4 | 38,54 | 17988 | Ndrg1 | 0 | 1,08 | 9,76E-01 |
| Q9DCZ4 | 27 | 5 | 80,41 | 68316 | Apoo | 0 | 0,93 | 9,25E-01 |
| P54227 | 36 | 4 | 82,23 | 16765 | Stmn1 | 2 | 0,96 | 9,74E-01 |
| Q9JL56 | 21 | 7 | 54,3 | 56209 | Gde1 | 0 | 0,99 | 9,78E-01 |
| Q9QUR7 | 50 | 5 | 26,98 | 23988 | Pin1 | 0 | 1,15 | 8,81E-01 |
| B2RUJ5 | 11 | 7 | 25,8 | 319924 | Apba1 | 0 | 1,57 | 4,46E-02 |
| P59999 | 36 | 6 | 78,99 | 68089 | Arpc4 | 0 | 1,17 | 7,90E-01 |
| P11352 | 29 | 6 | 38,41 | 14775 | Gpx1 | 0 | 1,01 | 1,00E+00 |
| Q9CWE0 | 20 | 5 | 31,23 | 76824 | Mtfr1l | 0 | 0,96 | 9,76E-01 |
| A2AGL3 | 2 | 5 | 3,88 | 20192 | Ryr3 | 0 | 0,86 | 9,47E-01 |
| Q9D379 | 22 | 9 | 24,37 | 13849 | Ephx1 | 0 | 1,11 | 9,41E-01 |
| Q61543 | 5 | 6 | 25,19 | 20340 | Glg1 | 0 | 0,99 | 9,94E-01 |
| Q9DBR7 | 9 | 7 | 15,81 | 17931 | Ppp1r12a | 0 | 1,51 | 2,83E-01 |
| Q9CQM9 | 19 | 6 | 34,21 | 30926 | Glrx3 | 0 | 0,95 | 9,60E-01 |
| Q9Z0F7 | 45 | 5 | 18,81 | 20618 | Sncg | 0 | 1,89 | 2,51E-03 |
| Q68ED2 | 10 | 6 | 23,08 | 108073 | Grm7 | 1 | 0,89 | 9,24E-01 |
| Q9DB60 | 27 | 5 | 31,96 | 66469 | Prxl2b | 0 | 1,09 | 9,64E-01 |

| | | | | | | | | |
|---|---|---|---|---|---|---|---|---|
| P28028 | 9 | 5 | 39,42 | | | 2 | 0,85 | 7,12E-01 |
| Q8BRU6 | 6 | 3 | 26,26 | 214084 | Slc18a2 | 0 | 3,45 | 1,60E-16 |
| P35283 | 25 | 4 | 151,48 | 19328 | Rab12 | 0 | 1,92 | 1,80E-01 |
| Q9D906 | 14 | 9 | 9,82 | 74244 | Atg7 | 0 | 1,23 | 6,82E-01 |
| Q6IR34 | 16 | 9 | 6,33 | 67839 | Gpsm1 | 0 | 1,13 | 9,12E-01 |
| G3X9K3 | 5 | 4 | 15,98 | 211673 | Arfgef1 | 0 | 0,98 | 9,76E-01 |
| Q64467 | 5 | 2 | 168,55 | 14447 | Gapdhs | 0 | 0,77 | 4,28E-01 |
| P59017 | 18 | 5 | 8,37 | 94044 | Bcl2l13 | 0 | 0,82 | 6,42E-01 |
| Q32M21 | 7 | 5 | 27,53 | 76758 | Gsdma2 | 0 | 0,77 | 4,61E-01 |
| Q8K0G5 | 17 | 5 | 31,98 | 380752 | Eipr1 | 0 | 1,30 | 5,50E-01 |
| Q11136 | 15 | 8 | 26,4 | 18624 | Pepd | 0 | 1,42 | 2,01E-01 |
| Q9D7N9 | 17 | 7 | 28,11 | 71881 | Apmap | 0 | 0,97 | 9,76E-01 |
| Q6ZPJ3 | 8 | 9 | 9,48 | 217342 | Ube2o | 0 | 1,39 | 4,72E-01 |
| Q9JKD3 | 25 | 3 | 246,67 | 56807 | Scamp5 | 0 | 1,21 | 7,23E-01 |
| Q01341 | 6 | 6 | 26,56 | 11512 | Adcy6 | 0 | 0,93 | 9,39E-01 |
| Q9JHW2 | 24 | 6 | 35,26 | 52633 | Nit2 | 0 | 0,77 | 5,45E-01 |
| Q9D710 | 19 | 6 | 34,27 | 66958 | Tmx2 | 0 | 1,00 | 1,00E+00 |
| Q8VD65 | 6 | 7 | 8,94 | 75669 | Pik3r4 | 0 | 1,07 | 9,78E-01 |
| Q9R1V4 | 8 | 5 | 23,79 | 11488 | Adam11 | 0 | 1,01 | 9,97E-01 |
| Q9WV54 | 15 | 6 | 16,9 | 11886 | Asah1 | 0 | 0,42 | 2,58E-05 |
| Q5M8N0 | 30 | 5 | 80,61 | 380686 | Cnrip1 | 0 | 1,16 | 8,57E-01 |
| Q80W21 | 19 | 3 | 101,45 | 68312 | Gstm7 | 0 | 1,06 | 9,74E-01 |
| Q8C5H8 | 17 | 6 | 19,17 | 68646 | Nadk2 | 0 | 0,59 | 2,27E-01 |
| ANXA5_HUMAN | 15 | 6 | 43,92 | | | 0 | 0,92 | 9,18E-01 |
| O08795 | 10 | 6 | 39,04 | 19089 | Prkcsh | 0 | 0,83 | 6,52E-01 |
| Q8CHT0 | 10 | 5 | 23,84 | 212647 | Aldh4a1 | 0 | 0,74 | 3,31E-01 |
| A2AJA9 | 9 | 7 | 10,72 | 381353 | Ajm1 | 0 | 1,16 | 8,94E-01 |
| Q924L1 | 20 | 6 | 17,9 | 68614 | Letmd1 | 0 | 0,79 | 6,95E-01 |
| Q8BRF7 | 11 | 5 | 24,6 | 76983 | Scfd1 | 0 | 1,09 | 9,76E-01 |
| Q3TPX4 | 12 | 8 | 21,11 | 105504 | Exoc5 | 0 | 0,91 | 9,12E-01 |
| Q8BGQ1 | 15 | 5 | 16,29 | 104799 | Vipas39 | 0 | 0,95 | 9,76E-01 |
| Q06185 | 66 | 5 | 86,04 | 11958 | Atp5me | 0 | 1,38 | 2,43E-01 |
| Q9CPX6 | 19 | 5 | 46,61 | 67841 | Atg3 | 0 | 1,36 | 6,66E-01 |
| Q8K1Z0 | 15 | 4 | 38,47 | 67914 | Coq9 | 0 | 0,68 | 1,44E-01 |
| Q62159 | 18 | 5 | 67,21 | 11853 | Rhoc | 0 | 0,96 | 9,64E-01 |
| Q9JKB1 | 31 | 5 | 8,34 | 50933 | Uchl3 | 0 | 0,83 | 7,86E-01 |
| Q8VCH0 | 12 | 4 | 22,9 | 235674 | Acaa1b | 0 | 1,10 | 9,76E-01 |
| P27546 | 7 | 6 | 37,02 | 17758 | Map4 | 0 | 0,75 | 4,31E-01 |
| Q3U0M1 | 7 | 7 | 14,12 | 76510 | Trappc9 | 0 | 0,98 | 9,78E-01 |
| CYC_HUMAN | 27 | 5 | 50,24 | | | 0 | 0,97 | 9,76E-01 |
| P34022 | 37 | 6 | 56,92 | 19385 | Ranbp1 | 0 | 1,01 | 9,99E-01 |
| Q8BYA0 | 7 | 6 | 15,12 | 108903 | Tbcd | 0 | 0,88 | 9,25E-01 |
| P36993 | 16 | 3 | 18,21 | 19043 | Ppm1b | 0 | 1,36 | 7,86E-01 |
| Q8BXK8 | 8 | 3 | 44,06 | 347722 | Agap1 | 0 | 1,23 | 9,03E-01 |
| Q6X893 | 10 | 5 | 20,73 | 100434 | Slc44a1 | 0 | 0,87 | 9,12E-01 |
| P34914 | 11 | 5 | 32,84 | 13850 | Ephx2 | 0 | 0,99 | 9,97E-01 |

| | | | | | | | | |
|---|---|---|---|---|---|---|---|---|
| Q00558 | 14 | 3 | 33,56 | | F8a1 | 0 | 0,94 | 9,74E-01 |
| Q9D4H1 | 9 | 8 | 10,59 | 66482 | Exoc2 | 0 | 1,10 | 9,56E-01 |
| Q8CIQ7 | 4 | 7 | 5,93 | | Dock3 | 1 | 1,19 | 8,93E-01 |
| P59108 | 10 | 4 | 27,45 | 234577 | Cpne2 | 0 | 0,85 | 8,93E-01 |
| O35344 | 12 | 4 | 13,16 | 16648 | Kpna3 | 2 | 0,96 | 9,76E-01 |
| Q9CY64 | 23 | 6 | 25,92 | 109778 | Blvra | 0 | 0,87 | 9,11E-01 |
| GAG_SCVLA | 10 | 8 | 30,75 | 940477 | gag | 0 | 0,01 | 1,60E-16 |
| Q3U5Q7 | 14 | 6 | 29,54 | 22169 | Cmpk2 | 0 | 1,03 | 9,91E-01 |
| Q60613 | 10 | 3 | 42,27 | 11540 | Adora2a | 0 | 0,64 | 1,53E-01 |
| Q9D8B3 | 19 | 4 | 43,29 | 75608 | Chmp4b | 0 | 1,05 | 9,76E-01 |
| Q6P9R2 | 13 | 5 | 3,85 | 108737 | Oxsr1 | 0 | 1,03 | 1,00E+00 |
| Q9JJ69 | 23 | 6 | 28,54 | 80906 | Kcnip2 | 0 | 0,95 | 9,64E-01 |
| Q8C7M3 | 7 | 5 | 29,73 | 94090 | Trim9 | 0 | 0,92 | 9,56E-01 |
| D3YZI9 | 12 | 6 | 40,52 | 209966 | Pgbd5 | 0 | 1,23 | 7,02E-01 |
| P62806 | 39 | 4 | 45,36 | 100041230; 319155; 319156; 319157; 319158; 319159; 319160; 319161; 320332; 326619; 326620; 69386; 97122 | H4c1; H4c11; H4c12; H4c14; H4c2; H4c3; H4c4; H4c6; H4c8; H4c9; H4f16; Hist1h4m | 0 | 1,21 | 7,41E-01 |
| Q9Z110 | 9 | 6 | 33,07 | 56454 | Aldh18a1 | 0 | 1,00 | 9,91E-01 |
| P30416 | 14 | 7 | 34,96 | 14228 | Fkbp4 | 0 | 1,09 | 9,57E-01 |
| A2A699 | 10 | 6 | 33,92 | 217219 | Fam171a2 | 0 | 0,86 | 7,33E-01 |
| P63028 | 23 | 3 | 32,98 | 22070 | Tpt1 | 0 | 1,25 | 6,72E-01 |
| Q8VHK5 | 14 | 3 | 11,39 | 170790 | Mlc1 | 0 | 1,07 | 9,78E-01 |
| Q9CQI3 | 31 | 4 | 40,1 | 63985 | Gmfb | 0 | 1,09 | 9,64E-01 |
| Q7TNR6 | 15 | 5 | 37,41 | 230868 | Igsf21 | 0 | 1,01 | 9,99E-01 |
| Q08189 | 5 | 3 | 44,86 | 21818 | Tgm3 | 0 | 0,87 | 7,90E-01 |
| P04925 | 13 | 3 | 46,34 | 19122 | Prnp | 0 | 1,05 | 9,76E-01 |
| P61082 | 30 | 6 | 39,05 | 22192 | Ube2m | 0 | 1,14 | 8,85E-01 |
| Q8CFE4 | 9 | 7 | 6,21 | 213326 | Scyl2 | 0 | 0,98 | 9,87E-01 |
| Q9WU79 | 11 | 4 | 24,59 | 19125 | Prodh | 0 | 1,17 | 9,56E-01 |
| Q7TPM6 | 10 | 5 | 25,55 | 240121 | Fsd1 | 0 | 1,08 | 9,70E-01 |
| Q9JM96 | 13 | 4 | 16,54 | 56699 | Cdc42ep4 | 0 | 1,06 | 9,81E-01 |
| Q9Z1Z0 | 8 | 7 | 12,02 | 56041 | Uso1 | 0 | 1,17 | 8,21E-01 |
| Q8CHX7 | 12 | 4 | 25,76 | 74013 | Rftn2 | 0 | 0,76 | 5,96E-01 |
| P01942 | 33 | 3 | 34,52 | | Hba | 1 | 0,82 | 6,42E-01 |
| O35316 | 10 | 4 | 52,69 | 21366 | Slc6a6 | 0 | 1,11 | 9,69E-01 |
| Q8R307 | 5 | 4 | 20,55 | 228545 | Vps18 | 0 | 1,10 | 9,76E-01 |
| P28661 | 14 | 5 | 76,01 | 18952 | Septin4 | 0 | 0,84 | 7,74E-01 |
| Q9JMC3 | 12 | 4 | 25,63 | 58233 | Dnaja4 | 0 | 1,01 | 1,00E+00 |

| | | | | | | | | |
|---|---|---|---|---|---|---|---|---|
| P99026 | 20 | 4 | 58,26 | 19172 | Psmb4 | 0 | 1,18 | 7,77E-01 |
| Q9CZJ2 | 5 | 4 | 52,52 | 72630 | Hspa12b | 0 | 1,06 | 9,76E-01 |
| O35250 | 9 | 5 | 17,04 | 53413 | Exoc7 | 0 | 1,37 | 6,72E-01 |
| Q61616 | 12 | 4 | 14,81 | 13488 | Drd1 | 0 | 0,85 | 7,78E-01 |
| Q9D023 | 32 | 5 | 30,69 | 70456 | Mpc2 | 0 | 0,85 | 7,38E-01 |
| Q61335 | 22 | 5 | 35,35 | 27061 | Bcap31 | 0 | 1,09 | 9,56E-01 |
| P97411 | 12 | 5 | 12,98 | 15893 | Ica1 | 0 | 1,05 | 9,78E-01 |
| B9EJ86 | 14 | 8 | 8,17 | 237542 | Osbpl8 | 0 | 1,08 | 9,76E-01 |
| Q3URD3 | 9 | 7 | 9,37 | 83997 | Slmap | 0 | 0,81 | 7,20E-01 |
| P97765 | 17 | 5 | 46,42 | 22378 | Wbp2 | 0 | 1,35 | 3,54E-01 |
| P70302 | 8 | 5 | 22,58 | 20866 | Stim1 | 0 | 1,07 | 9,76E-01 |
| O35304 | 8 | 4 | 30,38 | | Slc18a3 | 0 | 1,13 | 9,56E-01 |
| P55264 | 17 | 6 | 17,96 | 11534 | Adk | 0 | 0,77 | 4,12E-01 |
| Q9CZ30 | 14 | 5 | 32,29 | 67059 | Ola1 | 0 | 0,96 | 9,74E-01 |
| Q9CPU0 | 35 | 8 | 67,16 | 109801 | Glo1 | 0 | 0,97 | 9,76E-01 |
| G3X9C2 | 9 | 3 | 53,82 | 233038 | Nccrp1 | 0 | 1,08 | 9,74E-01 |
| Q9WVQ1 | 6 | 5 | 11,49 | 50791 | Magi2 | 0 | 1,05 | 9,85E-01 |
| P60122 | 23 | 7 | 14,61 | 56505 | Ruvbl1 | 0 | 1,22 | 8,53E-01 |
| Q61016 | 63 | 5 | 43,59 | | Gng7 | 0 | 0,82 | 6,22E-01 |
| P24288 | 15 | 6 | 47,4 | 12035 | Bcat1 | 0 | 1,47 | 1,32E-01 |
| Q9D1E6 | 26 | 6 | 27,93 | 66411 | Tbcb | 0 | 1,14 | 8,80E-01 |
| Q8BHE8 | 20 | 5 | 29,64 | 68115 | Maip1 | 0 | 0,92 | 9,33E-01 |
| O70251 | 16 | 3 | 26,03 | 55949 | Eef1b | 0 | 0,81 | 7,50E-01 |
| Q0GA42 | 8 | 5 | 4 | 83674 | Cnnm1 | 2 | 1,38 | 4,87E-01 |
| HBB_HUMAN | 36 | 4 | 53,96 | | | 1 | 1,35 | 4,83E-01 |
| O55100 | 15 | 3 | 122,47 | 20972 | Syngr1 | 0 | 1,10 | 9,34E-01 |
| KRHB2_HUMAN | 12 | 2 | 13,27 | | | 0 | 0,78 | 8,50E-01 |
| P62774 | 33 | 3 | 23,99 | 14489 | Mtpn | 0 | 1,13 | 9,13E-01 |
| Q6A4J8 | 6 | 6 | 15,33 | 252870 | Usp7 | 0 | 0,93 | 9,64E-01 |
| O35350 | 9 | 6 | 7,37 | 12333 | Capn1 | 0 | 0,84 | 8,78E-01 |
| Q3TDN2 | 16 | 5 | 13,92 | 76577 | Faf2 | 0 | 0,90 | 9,00E-01 |
| Q64674 | 21 | 6 | 25,56 | 20810 | Srm | 0 | 0,84 | 7,33E-01 |
| P58321 | 27 | 4 | 8,34 | 93841 | Uchl4 | 0 | 0,94 | 9,76E-01 |
| Q9D387 | 11 | 3 | 60,25 | 76161 | Lamp5 | 0 | 1,03 | 9,96E-01 |
| Q9JKL5 | 28 | 5 | 11,56 | 57816 | Tesc | 0 | 0,85 | 8,95E-01 |
| Q8BL86 | 27 | 7 | 21,69 | 72852 | Mblac2 | 0 | 1,30 | 5,10E-01 |
| Q9DC61 | 12 | 6 | 17,02 | 66865 | Pmpca | 0 | 0,85 | 8,56E-01 |
| Q8CH72 | 10 | 5 | 27,61 | 69807 | Trim32 | 0 | 1,04 | 9,85E-01 |
| Q99M71 | 16 | 3 | 26,62 | 105298 | Epdr1 | 0 | 0,56 | 2,87E-02 |
| P60755 | 8 | 6 | 3,88 | 320772 | Mdga2 | 0 | 1,00 | 9,87E-01 |
| P52189 | 11 | 4 | 7,25 | | Kcnj4 | 0 | 1,20 | 7,41E-01 |
| Q91XY4 | 6 | 4 | 23,94 | 93712 | Pcdhga4 | 0 | 1,33 | 3,99E-01 |
| Q8R050 | 8 | 6 | 30,61 | 14852 | Gspt1 | 0 | 1,23 | 7,61E-01 |
| Q6PNC0 | 3 | 7 | 5,77 | 240283 | Dmxl1 | 0 | 1,08 | 9,76E-01 |
| Q9DCM2 | 19 | 3 | 17,12 | 76263 | Gstk1 | 0 | 0,52 | 1,21E-01 |
| Q8K4Q0 | 5 | 5 | 20,92 | | Rptor | 0 | 1,26 | 8,85E-01 |

| | | | | | | | | |
|---|---|---|---|---|---|---|---|---|
| Q9QZB1 | 23 | 4 | 34,51 | 58175 | Rgs20 | 1 | 0,95 | 9,61E-01 |
| Q9D7A8 | 13 | 5 | 37,88 | 74252 | Armc1 | 0 | 1,00 | 9,87E-01 |
| Q61037 | 6 | 8 | 0 | | Tsc2 | 0 | 0,85 | 9,02E-01 |
| Q9JJL8 | 16 | 5 | 6,43 | 71984 | Sars2 | 0 | 1,10 | 9,76E-01 |
| O70228 | 5 | 5 | 17,6 | 11981 | Atp9a | 0 | 1,05 | 9,87E-01 |
| Q8JZN7 | 8 | 2 | 33,46 | 214952 | Rhot2 | 0 | 0,59 | 4,27E-01 |
| Q99LB6 | 18 | 4 | 21,24 | 108645 | Mat2b | 0 | 1,00 | 9,96E-01 |
| P55096 | 13 | 6 | 5,68 | 19299 | Abcd3 | 0 | 1,13 | 9,28E-01 |
| Q68FD9 | 6 | 8 | 8,7 | 330286 | Kiaa1549 | 0 | 0,83 | 8,70E-01 |
| Q8BP92 | 8 | 4 | 30,02 | 26611 | Rcn2 | 0 | 0,47 | 4,61E-02 |
| Q3UHB8 | 6 | 4 | 11,55 | 380768 | Ccdc177 | 0 | 1,12 | 9,74E-01 |
| Q62348 | 20 | 4 | 18,12 | 22099 | Tsn | 0 | 1,25 | 8,69E-01 |
| Q9D2M8 | 25 | 4 | 77,33 | 70620 | Ube2v2 | 0 | 1,06 | 9,74E-01 |
| Q9ESN4 | 16 | 3 | 25,38 | 227580 | C1ql3 | 0 | 1,01 | 1,00E+00 |
| A6H5Z3 | 7 | 5 | 32,48 | 75914 | Exoc6b | 0 | 1,05 | 9,81E-01 |
| Q8BFP9 | 13 | 4 | 25,73 | 228026 | Pdk1 | 1 | 0,80 | 7,38E-01 |
| Q7M729 | 14 | 4 | 47,77 | 399548 | Scn4b | 0 | 0,98 | 9,78E-01 |
| Q8BG92 | 14 | 2 | 39,09 | 215890 | Clvs2 | 0 | 0,76 | 7,90E-01 |
| Q8CGA0 | 11 | 4 | 16,97 | 68606 | Ppm1f | 0 | 1,05 | 9,81E-01 |
| P53811 | 22 | 2 | 33,48 | 56305 | Pitpnb | 0 | 0,65 | 6,13E-01 |
| Q9CPV9 | 15 | 5 | 25,78 | 70839 | P2ry12 | 0 | 0,40 | 1,45E-06 |
| Q9QYI5 | 16 | 5 | 31,5 | 56812 | Dnajb2 | 0 | 1,30 | 6,95E-01 |
| Q3UHD1 | 5 | 7 | 5,1 | 107831 | Adgrb1 | 0 | 0,85 | 7,52E-01 |
| P49813 | 17 | 5 | 12,87 | 21916 | Tmod1 | 0 | 0,76 | 7,49E-01 |
| P97384 | 12 | 5 | 27,38 | 11744 | Anxa11 | 0 | 0,77 | 6,64E-01 |
| P61750 | 16 | 2 | 50,65 | 11843 | Arf4 | 0 | 0,92 | 9,64E-01 |
| Q8R1S0 | 13 | 6 | 20,13 | 217707 | Coq6 | 0 | 0,50 | 4,72E-03 |
| Q3UPL0 | 6 | 6 | 16,8 | 69162 | Sec31a | 0 | 1,10 | 9,64E-01 |
| CYC_HORSE | 26 | 5 | 26,96 | | CYCS | 0 | 0,83 | 6,29E-01 |
| Q9CZR8 | 14 | 2 | 33,31 | 66399 | Tsfm | 0 | 0,72 | 6,44E-01 |
| Q5GH67 | 7 | 3 | 51,28 | 497097 | Xkr4 | 0 | 1,60 | 1,50E-01 |
| A2AWA9 | 6 | 5 | 11,51 | 227800 | Rabgap1 | 0 | 1,42 | 6,09E-01 |
| P10107 | 20 | 6 | 29,08 | 16952 | Anxa1 | 0 | 1,20 | 7,49E-01 |
| Q8BH57 | 11 | 6 | 14,4 | 67561 | Wdr48 | 0 | 1,16 | 8,43E-01 |
| Q61820 | 22 | 5 | 68,56 | 19428 | Rasl2-9 | 0 | 1,05 | 9,78E-01 |
| O08530 | 9 | 3 | 36,98 | 13609 | S1pr1 | 0 | 0,97 | 9,81E-01 |
| Q64442 | 17 | 6 | 14,2 | 20322 | Sord | 0 | 1,06 | 9,76E-01 |
| P15626 | 16 | 2 | 80,47 | 14863 | Gstm2 | 0 | 1,17 | 8,21E-01 |
| Q91Z53 | 15 | 5 | 56,39 | 76238 | Grhpr | 0 | 1,13 | 9,02E-01 |
| O08579 | 22 | 4 | 33,6 | 13726 | Emd | 0 | 1,35 | 6,23E-01 |
| Q8CHK3 | 8 | 3 | 15,91 | 77582 | Mboat7 | 0 | 0,99 | 9,85E-01 |
| Q9CQR6 | 14 | 4 | 33,01 | 67857 | Ppp6c | 0 | 1,04 | 9,89E-01 |
| Q9D3D9 | 14 | 2 | 72,52 | 66043 | Atp5f1d | 0 | 0,85 | 7,52E-01 |
| Q60780 | 14 | 5 | 6,18 | | Gas7 | 0 | 1,13 | 9,19E-01 |
| Q6PF93 | 7 | 5 | 15,02 | 225326 | Pik3c3 | 0 | 1,03 | 9,96E-01 |
| Q5EBJ4 | 23 | 4 | 17,36 | 77767 | Ermn | 0 | 0,85 | 8,94E-01 |

| | | | | | | | | |
|---|---|---|---|---|---|---|---|---|
| Q05186 | 15 | 4 | 15,5 | 19672 | Rcn1 | 0 | 0,87 | 8,91E-01 |
| Q91Z61 | 22 | 4 | 24,48 | 208666 | Diras1 | 0 | 1,05 | 9,76E-01 |
| Q9WUL7 | 26 | 4 | 22,05 | 56350 | Arl3 | 0 | 1,27 | 7,42E-01 |
| Q9CX00 | 15 | 6 | 28,63 | 71955 | Ist1 | 0 | 0,92 | 9,07E-01 |
| B2RXS4 | 4 | 5 | 30,88 | 140570 | Plxnb2 | 0 | 0,81 | 8,40E-01 |
| P63143 | 13 | 3 | 38,48 | 16497 | Kcnab1 | 0 | 1,17 | 9,56E-01 |
| O88531 | 18 | 4 | 11,72 | 19063 | Ppt1 | 0 | 0,83 | 7,93E-01 |
| O35544 | 4 | 2 | 23,03 | 20513 | Slc1a6 | 0 | 0,91 | 9,00E-01 |
| Q91W89 | 8 | 7 | 14,69 | 73744 | Man2c1 | 0 | 0,92 | 9,68E-01 |
| Q5SV85 | 4 | 3 | 15,72 | 217030 | Synrg | 0 | 0,93 | 9,76E-01 |
| Q9ERR1 | 14 | 4 | 37,32 | 83431 | Ndel1 | 0 | 1,01 | 1,00E+00 |
| Q6PDS3 | 8 | 5 | 17,92 | 237868 | Sarm1 | 0 | 1,03 | 9,97E-01 |
| Q61176 | 9 | 3 | 53,1 | 11846 | Arg1 | 0 | 1,07 | 9,74E-01 |
| P23927 | 31 | 5 | 21,04 | 12955 | Cryab | 0 | 0,76 | 7,49E-01 |
| Q9JJV5 | 16 | 2 | 12,84 | 54376 | Cacng3 | 0 | 1,27 | 8,86E-01 |
| Q9QYF1 | 14 | 4 | 24,57 | 17252 | Rdh11 | 0 | 0,81 | 8,10E-01 |
| P97494 | 11 | 5 | 14,71 | 14629 | Gclc | 0 | 0,81 | 7,90E-01 |
| Q8K4X7 | 16 | 6 | 17,24 | 68262 | Agpat4 | 0 | 1,38 | 5,13E-01 |
| P70429 | 18 | 5 | 16,61 | 14026 | Evl | 0 | 0,43 | 1,05E-02 |
| Q9QXJ1 | 8 | 4 | 13,97 | 11785 | Apbb1 | 0 | 1,06 | 9,81E-01 |
| Q921J2 | 19 | 5 | 20,42 | 19744 | Rheb | 0 | 1,53 | 9,64E-02 |
| Q8CI32 | 12 | 4 | 18,86 | 70369 | Bag5 | 0 | 1,11 | 9,69E-01 |
| P20152 | 12 | 2 | 82,1 | 22352 | Vim | 0 | 0,95 | 9,78E-01 |
| P29391 | 42 | 5 | 9,1 | | Ftl1 | 0 | 0,98 | 9,85E-01 |
| Q9D8B4 | 21 | 3 | 27,5 | | Ndufa11 | 0 | 0,74 | 6,55E-01 |
| P62908 | 23 | 5 | 32,74 | 27050 | Rps3 | 0 | 0,79 | 7,33E-01 |
| Q3TES0 | 5 | 4 | 11,95 | 243621 | Iqsec3 | 0 | 1,11 | 9,76E-01 |
| Q8VE47 | 10 | 3 | 16,62 | 66663 | Uba5 | 0 | 1,08 | 9,76E-01 |
| P12815 | 23 | 4 | 39,71 | 18570 | Pdcd6 | 0 | 0,89 | 9,17E-01 |
| G3XA57 | 13 | 5 | 10,05 | 74998 | Rab11fip 2 | 0 | 1,41 | 6,59E-01 |
| Q61655 | 11 | 5 | 15,54 | 13680 | Ddx19a | 0 | 0,88 | 9,34E-01 |
| Q4VAE3 | 22 | 5 | 38,48 | 74868 | Tmem65 | 0 | 0,98 | 9,76E-01 |
| Q924M7 | 8 | 2 | 15,8 | 110119 | Mpi | 0 | 0,94 | 9,76E-01 |
| Q99LD8 | 26 | 5 | 18,75 | 51793 | Ddah2 | 0 | 0,83 | 7,81E-01 |
| Q9CXW2 | 15 | 5 | 11,68 | 64655 | Mrps22 | 0 | 0,76 | 7,42E-01 |
| P34884 | 24 | 3 | 66,22 | 17319 | Mif | 0 | 0,92 | 9,12E-01 |
| Q3THE2 | 24 | 4 | 27,65 | 67938 | Myl12b | 0 | 1,04 | 9,89E-01 |
| Q5RJI5 | 7 | 3 | 19,63 | 381979 | Brsk1 | 0 | 0,94 | 9,76E-01 |
| Q8BXZ1 | 11 | 5 | 22,78 | 67988 | Tmx3 | 0 | 0,79 | 4,92E-01 |
| Q9R1P1 | 18 | 3 | 19,91 | 26446 | Psmb3 | 0 | 1,06 | 9,76E-01 |
| Q9ER88 | 16 | 5 | 8,22 | 65111 | Dap3 | 0 | 0,60 | 2,63E-01 |
| O88456 | 19 | 3 | 19,04 | 12336 | Capns1 | 2 | 0,88 | 8,54E-01 |
| Q6ZQI3 | 14 | 4 | 29,73 | 109154 | Mlec | 0 | 1,19 | 8,70E-01 |
| Q9R0D8 | 23 | 5 | 8,35 | 75659 | Wdr54 | 0 | 1,05 | 9,96E-01 |
| Q3URS9 | 13 | 4 | 7,03 | 66658 | Ccdc51 | 0 | 0,90 | 9,56E-01 |
| Q8BI08 | 18 | 2 | 52,32 | 105853 | Mal2 | 0 | 1,08 | 9,59E-01 |

| | | | | | | | | |
|---|---|---|---|---|---|---|---|---|
| Q8BZ81 | 10 | 4 | 17,42 | 216028 | Lrrtm3 | 1 | 0,98 | 9,78E-01 |
| Q9Z0N1 | 9 | 3 | 13,62 | 26905 | Eif2s3x | 0 | 1,95 | 2,86E-01 |
| Q9CQ69 | 37 | 3 | 49,23 | 22272 | Uqcrq | 0 | 0,91 | 9,05E-01 |
| P53986 | 12 | 4 | 40,79 | 20501 | Slc16a1 | 0 | 1,12 | 9,21E-01 |
| Q6PAR5 | 5 | 7 | 1,89 | 66691 | Gapvd1 | 0 | 1,05 | 9,76E-01 |
| O35226 | 14 | 3 | 20,11 | 19185 | Psmd4 | 0 | 1,05 | 9,96E-01 |
| P32211 | 11 | 2 | 22,81 | 12672 | Chrm4 | 0 | 0,72 | 3,52E-01 |
| Q60771 | 11 | 2 | 8,91 | 18417 | Cldn11 | 0 | 1,03 | 9,94E-01 |
| Q8R2Y0 | 15 | 4 | 18,79 | 66082 | Abhd6 | 0 | 1,01 | 1,00E+00 |
| O55033 | 14 | 4 | 12,74 | 17974 | Nck2 | 1 | 0,99 | 9,87E-01 |
| Q80U49 | 5 | 4 | 1,84 | 217882 | Cep170b | 0 | 1,55 | 5,86E-01 |
| Q99LP6 | 22 | 4 | 17,31 | 17713 | Grpel1 | 0 | 0,69 | 5,00E-01 |
| O08919 | 9 | 3 | 42,54 | 18223 | Numbl | 1 | 0,79 | 5,56E-01 |
| Q9D6Y9 | 8 | 5 | 32,89 | 74185 | Gbe1 | 0 | 1,61 | 1,08E-01 |
| Q9DCS3 | 12 | 5 | 5,21 | 26922 | Mecr | 0 | 1,08 | 9,74E-01 |
| Q9D4J1 | 9 | 2 | 21,03 | 98363 | Efhd1 | 0 | 0,79 | 4,86E-01 |
| P58774 | 12 | 5 | 38,6 | 22004 | Tpm2 | 0 | 1,83 | 1,83E-03 |
| Q9R0Q7 | 19 | 4 | 31,53 | 56351 | Ptges3 | 0 | 0,89 | 8,78E-01 |
| Q149F3 | 7 | 5 | 21,01 | 14853 | Gspt2 | 0 | 0,94 | 9,74E-01 |
| Q3TYD6 | 5 | 6 | 5,16 | 231876 | Lmtk2 | 0 | 1,08 | 9,76E-01 |
| Q9JM14 | 22 | 3 | 24,55 | 50773 | Nt5c | 0 | 1,24 | 9,03E-01 |
| Q60673 | 4 | 2 | 16,11 | | Ptprn | 0 | 1,07 | 9,76E-01 |
| O54984 | 16 | 5 | 38,26 | 56495 | Get3 | 0 | 1,22 | 7,20E-01 |
| P50136 | 10 | 3 | 21,33 | | Bckdha | 0 | 0,54 | 8,64E-02 |
| P10605 | 15 | 4 | 14,28 | 13030 | Ctsb | 0 | 0,54 | 5,41E-02 |
| Q8BWG8 | 13 | 5 | 15,56 | 109689 | Arrb1 | 0 | 1,09 | 9,70E-01 |
| P70202 | 18 | 3 | 67,79 | 17035 | Lxn | 0 | 0,84 | 7,09E-01 |
| Q9QUR8 | 6 | 3 | 13,08 | 20361 | Sema7a | 0 | 1,24 | 9,02E-01 |
| Q3V0K9 | 6 | 2 | 35,55 | 102502 | Pls1 | 0 | 0,91 | 9,18E-01 |
| Q63836 | 14 | 6 | 6,89 | 20342 | Selenbp2 | 0 | 0,73 | 1,89E-01 |
| P17563 | 14 | 6 | 6,89 | 20341 | Selenbp1 | 0 | 0,73 | 1,89E-01 |
| Q8BNY6 | 23 | 4 | 27,6 | 14299 | Ncs1 | 0 | 0,85 | 6,94E-01 |
| Q9CXR1 | 15 | 5 | 23,68 | 66375 | Dhrs7 | 0 | 1,11 | 9,40E-01 |
| Q9CQF9 | 10 | 3 | 25,9 | 66881 | Pcyox1 | 0 | 1,19 | 9,25E-01 |
| Q9D0S9 | 34 | 4 | 34,72 | 68917 | Hint2 | 0 | 0,82 | 8,11E-01 |
| Q8BG67 | 6 | 3 | 36,85 | 76740 | Efr3a | 0 | 0,83 | 9,13E-01 |
| Q8C561 | 7 | 4 | 19,53 | 320506 | Lmbrd2 | 0 | 1,12 | 9,62E-01 |
| P22315 | 15 | 5 | 11,78 | | Fech | 0 | 0,66 | 1,88E-01 |
| P57716 | 7 | 5 | 19,62 | 59287 | Ncstn | 0 | 0,70 | 4,20E-01 |
| Q9Z2Z6 | 16 | 5 | 42,76 | 57279 | Slc25a20 | 0 | 0,95 | 9,56E-01 |
| P49722 | 17 | 3 | 39,65 | 19166 | Psma2 | 0 | 1,01 | 1,00E+00 |
| P53612 | 12 | 4 | 22,13 | 19352 | Rabggtb | 0 | 0,94 | 9,56E-01 |
| Q8K4L4 | 9 | 5 | 7,34 | 69693 | Pof1b | 0 | 2,39 | 1,19E-02 |
| P04627 | 9 | 3 | 30,97 | 11836 | Araf | 0 | 0,81 | 8,07E-01 |
| Q6P069 | 23 | 5 | 27,86 | 109552 | Sri | 0 | 1,26 | 6,12E-01 |
| Q9DBL7 | 12 | 5 | 27,03 | 71743 | Coasy | 0 | 0,94 | 9,74E-01 |
| P61087 | 24 | 4 | 5,99 | 53323 | Ube2k | 0 | 0,89 | 9,42E-01 |

| | | | | | | | | |
|---|---|---|---|---|---|---|---|---|
| P35288 | 19 | 4 | 23,11 | | Rab23 | 0 | 1,13 | 9,12E-01 |
| Q99KP3 | 12 | 3 | 15,66 | 68631 | Cryl1 | 0 | 0,93 | 9,76E-01 |
| Q8VED9 | 25 | 4 | 53,61 | 216551 | Lgalsl | 0 | 1,21 | 7,33E-01 |
| Q9WUD1 | 15 | 4 | 17,07 | 56424 | Stub1 | 0 | 0,81 | 8,45E-01 |
| Q9WU65 | 9 | 4 | 26,54 | 14626 | Gk2 | 0 | 0,86 | 7,74E-01 |
| Q9Z2A0 | 12 | 5 | 4,19 | 18607 | Pdpk1 | 0 | 1,13 | 9,56E-01 |
| Q9D1X0 | 18 | 3 | 4,14 | 78688 | Nol3 | 0 | 1,69 | 5,99E-01 |
| Q62167 | 8 | 5 | 11,47 | 13205 | Ddx3x | 0 | 1,06 | 9,87E-01 |
| P16381 | 8 | 5 | 11,47 | 110957 | D1Pas1 | 0 | 1,06 | 9,87E-01 |
| Q9CQC9 | 21 | 4 | 24,77 | 66397 | Sar1b | 0 | 1,14 | 9,02E-01 |
| Q8VBW6 | 8 | 4 | 29,84 | 234664 | Nae1 | 0 | 1,07 | 9,78E-01 |
| Q9Z2I8 | 12 | 4 | 15,6 | 20917 | Suclg2 | 0 | 0,74 | 6,94E-01 |
| Q9D0R2 | 9 | 5 | 13,26 | 110960 | Tars1 | 1 | 0,85 | 8,50E-01 |
| P70227 | 2 | 6 | 1,68 | 16440 | Itpr3 | 0 | 1,13 | 9,34E-01 |
| Q60996 | 10 | 3 | 16,57 | 26931 | Ppp2r5c | 0 | 0,95 | 9,76E-01 |
| Q8CAK1 | 10 | 3 | 21,89 | 216792 | Iba57 | 0 | 0,58 | 2,16E-01 |
| P58242 | 12 | 4 | 18,24 | 100340 | Smpdl3b | 0 | 0,84 | 8,72E-01 |
| Q9Z0H8 | 5 | 5 | 7,1 | 269713 | Clip2 | 0 | 1,23 | 9,12E-01 |
| Q99M87 | 8 | 3 | 18,89 | 83945 | Dnaja3 | 0 | 0,93 | 9,72E-01 |
| P11438 | 6 | 2 | 34,23 | 16783 | Lamp1 | 0 | 0,79 | 5,01E-01 |
| O55026 | 10 | 4 | 31,82 | 12496 | Entpd2 | 0 | 0,88 | 8,57E-01 |
| Q8VD26 | 12 | 3 | 13,83 | 70209 | Tmem143 | 0 | 0,95 | 9,76E-01 |
| Q9EQQ9 | 6 | 5 | 3,27 | 76055 | Oga | 0 | 1,70 | 1,18E-01 |
| Q8CBH5 | 10 | 5 | 5,69 | 98682 | Mfsd6 | 0 | 2,25 | 5,92E-02 |
| P26883 | 26 | 2 | 51,9 | 14225 | Fkbp1a | 0 | 0,98 | 9,76E-01 |
| Q3UUQ7 | 5 | 5 | 10,03 | 241062 | Pgap1 | 0 | 0,93 | 9,66E-01 |
| P20060 | 9 | 4 | 18,12 | 15212 | Hexb | 0 | 0,64 | 5,56E-02 |
| Q9QZ88 | 23 | 4 | 42,54 | 56433 | Vps29 | 0 | 1,20 | 7,89E-01 |
| Q8BM13 | 11 | 5 | 22,67 | 244723 | Olfm2 | 0 | 0,88 | 8,64E-01 |
| Q64737 | 7 | 6 | 13,79 | 14450 | Gart | 0 | 1,24 | 7,51E-01 |
| Q6P8J7 | 6 | 2 | 25,74 | 76722 | Ckmt2 | 0 | 0,68 | 8,19E-02 |
| Q3TIR3 | 11 | 4 | 9,13 | 101489 | Ric8a | 0 | 0,84 | 9,25E-01 |
| Q9CXT8 | 10 | 4 | 50,99 | 73078 | Pmpcb | 0 | 0,72 | 6,13E-01 |
| Q8R3P0 | 12 | 3 | 6,23 | 11484 | Aspa | 0 | 0,66 | 4,00E-01 |
| Q91Z67 | 4 | 4 | 27,23 | 14270 | Srgap2 | 0 | 1,01 | 1,00E+00 |
| Q91XU3 | 9 | 3 | 21,4 | 117150 | Pip4k2c | 0 | 1,15 | 9,70E-01 |
| Q9QZI8 | 5 | 2 | 28,45 | 56442 | Serinc1 | 0 | 0,65 | 3,83E-01 |
| Q9CQ75 | 30 | 3 | 17,51 | 17991 | Ndufa2 | 0 | 1,24 | 7,52E-01 |
| B2RQC6 | 4 | 7 | 1,78 | 69719 | Cad | 0 | 1,31 | 8,10E-01 |
| P70195 | 19 | 5 | 41,48 | 19177 | Psmb7 | 0 | 0,81 | 6,23E-01 |
| Q99MR3 | 7 | 5 | 7,52 | 83704 | Slc12a9 | 0 | 0,93 | 9,74E-01 |
| Q6ZQK5 | 7 | 4 | 7,43 | 78618 | Acap2 | 0 | 0,55 | 3,33E-01 |
| A2APV2 | 7 | 6 | 1,64 | 71409 | Fmnl2 | 0 | 0,99 | 9,96E-01 |
| Q60738 | 11 | 4 | 31,41 | 22782 | Slc30a1 | 0 | 0,98 | 9,87E-01 |
| Q99KQ4 | 11 | 4 | 5,82 | 59027 | Nampt | 0 | 1,33 | 8,10E-01 |
| Q8BGN8 | 10 | 3 | 13,9 | 72003 | Synpr | 0 | 0,79 | 5,77E-01 |
| P54923 | 20 | 5 | 4,02 | 11544 | Adprh | 0 | 1,28 | 8,53E-01 |

| | | | | | | | | |
|---|---|---|---|---|---|---|---|---|
| Q9QZ08 | 17 | 5 | 19,86 | 56174 | Nagk | 0 | 1,32 | 5,82E-01 |
| Q8VBT0 | 12 | 4 | 25,13 | 72736 | Tmx1 | 0 | 1,07 | 9,85E-01 |
| Q8R555 | 9 | 5 | 9,61 | 72832 | Crtac1 | 0 | 1,02 | 9,94E-01 |
| Q8BK08 | 23 | 4 | 28,45 | 216821 | Tmem11 | 0 | 0,98 | 9,76E-01 |
| Q3TC33 | 15 | 4 | 33,78 | 67433 | Ccdc127 | 0 | 0,85 | 7,42E-01 |
| Q8K0D5 | 7 | 5 | 16,3 | 28030 | Gfm1 | 0 | 0,63 | 1,50E-01 |
| Q9EQ06 | 14 | 5 | 16,54 | 114664 | Hsd17b11 | 0 | 0,86 | 8,82E-01 |
| P46935 | 7 | 4 | 14,58 | 17999 | Nedd4 | 0 | 1,43 | 7,10E-01 |
| Q9QZB7 | 13 | 5 | 18,88 | 56444 | Actr10 | 0 | 1,07 | 9,74E-01 |
| Q8BMS4 | 9 | 2 | 15,97 | 230027 | Coq3 | 0 | 0,50 | 1,24E-01 |
| Q8BHW2 | 13 | 5 | 15,31 | 230751 | Oscp1 | 0 | 0,90 | 9,32E-01 |
| P51855 | 12 | 5 | 19,99 | 14854 | Gss | 0 | 1,00 | 9,97E-01 |
| Q99J09 | 15 | 4 | 13,06 | 70465 | Wdr77 | 0 | 1,05 | 9,85E-01 |
| Q9QZD8 | 20 | 5 | 13,02 | 27376 | Slc25a10 | 0 | 0,92 | 9,53E-01 |
| O55013 | 23 | 5 | 26,77 | 27096 | Trappc3 | 0 | 1,48 | 2,33E-01 |
| Q9R0N0 | 14 | 5 | 13,91 | 14635 | Galk1 | 0 | 1,01 | 1,00E+00 |
| Q8VE70 | 24 | 6 | 21,05 | 56426 | Pdcd10 | 0 | 0,80 | 5,42E-01 |
| O55017 | 2 | 5 | 11,04 | 12287 | Cacna1b | 0 | 1,26 | 7,77E-01 |
| Q8R4N0 | 15 | 4 | 12,48 | 69634 | Clybl | 0 | 1,32 | 7,77E-01 |
| Q99J83 | 12 | 3 | 18,25 | 11793 | Atg5 | 0 | 1,08 | 9,76E-01 |
| Q80XK6 | 3 | 6 | 1,74 | 76559 | Atg2b | 0 | 1,01 | 9,94E-01 |
| Q9D6P8 | 25 | 4 | 42,87 | 70405 | Calml3 | 0 | 0,99 | 9,77E-01 |
| Q9CX56 | 14 | 6 | 18,09 | 57296 | Psmd8 | 0 | 1,03 | 9,85E-01 |
| Q9CZW4 | 8 | 4 | 10,91 | 74205 | Acsl3 | 0 | 1,27 | 8,44E-01 |
| Q8BMF3 | 8 | 5 | 24,72 | 109264 | Me3 | 1 | 0,97 | 9,76E-01 |
| Q8BZN6 | 3 | 6 | 1,67 | 210293 | Dock10 | 0 | 0,87 | 9,47E-01 |
| Q3TY86 | 8 | 5 | 14,89 | 72168 | Aifm3 | 0 | 1,06 | 9,78E-01 |
| Q8BFY9 | 6 | 2 | 7,5 | 238799 | Tnpo1 | 2 | 0,85 | 9,32E-01 |
| Q9R0M0 | 2 | 6 | 1,89 | 53883 | Celsr2 | 0 | 0,65 | 4,52E-01 |
| P42567 | 8 | 5 | 2 | 13858 | Eps15 | 0 | 1,26 | 8,50E-01 |
| Q8BHK2 | 29 | 4 | 27,09 | 235281 | Scn3b | 0 | 1,44 | 4,49E-01 |
| P63115 | 8 | 2 | 13,84 | 53896 | Slc7a10 | 0 | 0,71 | 6,42E-01 |
| Q9JKV1 | 11 | 4 | 24,19 | 56436 | Adrm1 | 0 | 1,16 | 8,88E-01 |
| LYSC_HUMAN | 18 | 3 | 34,57 | 100970943; 4069; 450190 | LYZ | 0 | 1,50 | 7,84E-02 |
| Q8BGD9 | 7 | 4 | 5,13 | 75705 | Eif4b | 0 | 1,19 | 9,16E-01 |
| Q99KK9 | 10 | 2 | 43,13 | 70791 | Hars2 | 0 | 0,01 | 1,60E-16 |
| Q922P8 | 7 | 5 | 12,88 | 98170 | Tmem132a | 0 | 0,96 | 9,76E-01 |
| Q8VD33 | 13 | 3 | 27,35 | 218544 | Sgtb | 0 | 1,20 | 9,16E-01 |
| Q60605 | 30 | 4 | 9,41 | 17904 | Myl6 | 0 | 0,97 | 9,76E-01 |
| Q8C0L0 | 13 | 3 | 30,43 | 52837 | Tmx4 | 0 | 0,96 | 9,76E-01 |
| P00397 | 4 | 2 | 33,66 | 17708 | Mtco1 | 0 | 1,43 | 2,03E-01 |
| Q3TC72 | 16 | 6 | 33,13 | 68126 | Fahd2 | 0 | 0,81 | 6,58E-01 |
| Q8R164 | 14 | 4 | 22 | 68021 | Bphl | 0 | 0,77 | 6,07E-01 |
| Q9CRD2 | 12 | 3 | 19,99 | 66736 | Emc2 | 0 | 0,80 | 8,21E-01 |

| | | | | | | | | |
|---|---|---|---|---|---|---|---|---|
| P06801 | 8 | 4 | 10,76 | 17436 | Me1 | 0 | 1,02 | 1,00E+00 |
| O88983 | 22 | 5 | 5,96 | 55943 | Stx8 | 0 | 0,86 | 7,77E-01 |
| Q99LY9 | 49 | 5 | 15,91 | 595136 | Ndufs5 | 0 | 0,72 | 1,88E-01 |
| Q80TY0 | 7 | 5 | 9,72 | 14269 | Fnbp1 | 0 | 1,10 | 9,70E-01 |
| Q66JS6 | 16 | 4 | 16,52 | 100042 807 | Eif3j2 | 0 | 0,96 | 9,76E-01 |
| Q3UGC7 | 16 | 4 | 16,52 | 78655 | Eif3j1 | 0 | 0,96 | 9,76E-01 |
| Q8WTY4 | 16 | 3 | 9,03 | 109006 | Ciapin1 | 0 | 1,37 | 7,77E-01 |
| Q91V64 | 12 | 3 | 17,45 | 66307 | Isoc1 | 0 | 1,56 | 5,58E-01 |
| Q9CZ04 | 14 | 3 | 9,11 | 26894 | Cops7a | 0 | 1,47 | 6,06E-01 |
| Q9EPW0 | 5 | 5 | 9,14 | 269180 | Inpp4a | 0 | 0,97 | 9,76E-01 |
| Q60692 | 13 | 3 | 72,96 | 19175 | Psmb6 | 0 | 1,28 | 5,13E-01 |
| Q9D358 | 24 | 3 | 41,47 | 11431 | Acp1 | 0 | 1,20 | 7,61E-01 |
| Q9D3D0 | 15 | 4 | 10,13 | 76080 | Ttpal | 0 | 1,26 | 8,60E-01 |
| Q8R5H1 | 4 | 3 | 24,06 | 14479 | Usp15 | 0 | 1,12 | 9,76E-01 |
| O88507 | 6 | 2 | 34,78 | 12804 | Cntfr | 0 | 0,86 | 8,10E-01 |
| Q8BFU3 | 9 | 4 | 16,2 | 235315 | Rnf214 | 0 | 0,87 | 9,25E-01 |
| P97371 | 19 | 5 | 11,49 | 19186 | Psme1 | 0 | 1,44 | 5,62E-01 |
| Q5HZI9 | 21 | 6 | 11,56 | 230125 | Slc25a51 | 0 | 1,18 | 9,11E-01 |
| Q9JKC8 | 10 | 2 | 35,86 | 55946 | Ap3m1 | 0 | 0,55 | 4,06E-01 |
| Q8K411 | 5 | 4 | 1,98 | 69617 | Pitrm1 | 0 | 0,99 | 9,91E-01 |
| Q9WTS6 | 2 | 4 | 3,34 | | Tenm3 | 0 | 1,05 | 9,97E-01 |
| Q7TPD3 | 5 | 6 | 4,97 | | Robo2 | 0 | 1,05 | 9,78E-01 |
| Q9CPP6 | 28 | 3 | 32,04 | 68202 | Ndufa5 | 0 | 0,89 | 8,91E-01 |
| CASK_BO VIN | 21 | 3 | 7,52 | 281728 | CSN3 | 0 | 0,76 | 6,23E-01 |
| O35405 | 6 | 3 | 65,4 | 18807 | Pld3 | 0 | 0,76 | 3,50E-01 |
| Q99K70 | 15 | 4 | 5,57 | 54170 | Rragc | 0 | 1,14 | 8,85E-01 |
| Q8K1N1 | 9 | 6 | 9,72 | 67452 | Pnpla8 | 0 | 0,66 | 3,84E-01 |
| Q8BT60 | 11 | 3 | 27,29 | 70568 | Cpne3 | 0 | 2,11 | 2,01E-01 |
| Q505D7 | 20 | 2 | 28,56 | 403187 | Opa3 | 1 | 0,85 | 7,79E-01 |
| Q03141 | 6 | 2 | 15,12 | 17169 | Mark3 | 0 | 1,04 | 9,91E-01 |
| Q69ZH9 | 6 | 5 | 9,91 | 58996 | Arhgap23 | 1 | 1,12 | 9,74E-01 |
| Q8VCX5 | 10 | 4 | 14,31 | 216001 | Micu1 | 0 | 0,51 | 4,45E-02 |
| P55821 | 21 | 2 | 52,3 | 20257 | Stmn2 | 0 | 1,50 | 5,79E-01 |
| Q9CR67 | 13 | 3 | 37,01 | 67878 | Tmem33 | 0 | 1,35 | 3,83E-01 |
| Q9QZ23 | 20 | 4 | 19,76 | 56748 | Nfu1 | 0 | 0,86 | 8,86E-01 |
| Q9D1H7 | 10 | 3 | 15,32 | 67604 | Get4 | 0 | 0,00 | 1,60E-16 |
| Q78IK2 | 47 | 3 | 11,38 | 66477 | Atp5md | 0 | 0,78 | 3,87E-01 |
| Q9Z1T1 | 4 | 6 | 31,76 | 11774 | Ap3b1 | 0 | 0,98 | 9,76E-01 |
| Q6PA06 | 8 | 3 | 10,7 | 56298 | Atl2 | 0 | 0,81 | 7,71E-01 |
| Q8BSZ2 | 21 | 4 | 4,28 | 11778 | Ap3s2 | 0 | 1,15 | 8,77E-01 |
| O35593 | 16 | 4 | 33,14 | 59029 | Psmd14 | 0 | 1,03 | 9,99E-01 |
| Q3UU96 | 3 | 2 | 10,03 | 226751 | Cdc42bpa | 0 | 0,93 | 9,76E-01 |
| O89051 | 14 | 3 | 31,08 | 16432 | Itm2b | 0 | 0,82 | 6,70E-01 |
| B2RXC1 | 4 | 4 | 6,36 | 320714 | Trappc11 | 0 | 1,19 | 8,91E-01 |
| Q922J3 | 4 | 5 | 0 | 56430 | Clip1 | 0 | 1,30 | 8,24E-01 |
| Q9EPK2 | 11 | 4 | 21,51 | 19889 | Rp2 | 0 | 0,98 | 9,79E-01 |

| | | | | | | | | |
|---|---|---|---|---|---|---|---|---|
| P02089 | 21 | 2 | 70,19 | 15130 | Hbb-b2 | 0 | 1,10 | 9,56E-01 |
| P49446 | 7 | 4 | 20,16 | 19267 | Ptpre | 0 | 0,83 | 6,94E-01 |
| P24472 | 22 | 5 | 42,65 | 14860 | Gsta4 | 0 | 0,99 | 9,76E-01 |
| Q99KW9 | 8 | 4 | 19,96 | 71927 | Itfg1 | 0 | 1,32 | 6,98E-01 |
| O08989 | 29 | 5 | 5,64 | 17532 | Mras | 0 | 1,18 | 8,59E-01 |
| Q9CRY7 | 14 | 4 | 14,61 | 66569 | Gdpd1 | 0 | 1,51 | 3,12E-01 |
| Q9JKF6 | 8 | 3 | 21,05 | 58235 | Nectin1 | 0 | 0,88 | 9,02E-01 |
| Q8BWM0 | 10 | 3 | 16,23 | 96979 | Ptges2 | 0 | 1,10 | 9,76E-01 |
| P97772 | 4 | 5 | 5,01 | 14816 | Grm1 | 0 | 0,84 | 8,25E-01 |
| Q8BH58 | 18 | 4 | 13,79 | 226591 | Tiprl | 0 | 0,92 | 9,56E-01 |
| Q9WTM5 | 9 | 4 | 21,96 | 20174 | Ruvbl2 | 0 | 1,19 | 8,25E-01 |
| Q9CQT1 | 11 | 3 | 25,27 | 67873 | Mri1 | 0 | 1,53 | 5,91E-01 |
| Q80TA1 | 5 | 2 | 13,65 | 28042 | Selenoi | 0 | 0,83 | 9,05E-01 |
| Q9JJV4 | 17 | 5 | 6,85 | 54377 | Cacng4 | 0 | 1,04 | 9,78E-01 |
| Q9DCJ1 | 13 | 3 | 13,78 | 56716 | Mlst8 | 0 | 1,13 | 9,64E-01 |
| Q922R1 | 10 | 4 | 7,66 | | | 0 | 1,67 | 2,64E-01 |
| O08759 | 6 | 4 | 21,53 | 22215 | Ube3a | 0 | 1,16 | 9,40E-01 |
| Q9CQ22 | 22 | 3 | 18,53 | 66508 | Lamtor1 | 0 | 0,80 | 7,52E-01 |
| P63242 | 25 | 5 | 9,83 | 276770 | Eif5a | 0 | 0,90 | 9,29E-01 |
| Q60803 | 8 | 4 | 8,91 | 22031 | Traf3 | 0 | 0,94 | 9,76E-01 |
| Q8BHK1 | 14 | 3 | 6,62 | 233280 | Nipa1 | 0 | 0,92 | 9,76E-01 |
| Q61205 | 17 | 3 | 15,03 | 18476 | Pafah1b3 | 0 | 1,23 | 8,21E-01 |
| Q9JHU9 | 10 | 5 | 10,15 | 71780 | Isyna1 | 0 | 1,38 | 6,15E-01 |
| Q58A65 | 3 | 4 | 23,5 | 70834 | Spag9 | 0 | 1,17 | 8,76E-01 |
| Q05816 | 30 | 5 | 27,21 | 16592 | Fabp5 | 0 | 0,90 | 8,88E-01 |
| P28867 | 5 | 3 | 22,66 | 18753 | Prkcd | 0 | 0,72 | 6,61E-01 |
| Q99PS0 | 7 | 2 | 27,69 | 94179 | Krt23 | 0 | 1,76 | 7,67E-02 |
| AMYS_HUMAN | 7 | 3 | 8,31 | 276; 277; 278 | AMY1A; AMY1B; AMY1C | 0 | 1,67 | 3,99E-01 |
| Q921Q7 | 7 | 4 | 9,06 | 225870 | Rin1 | 0 | 1,09 | 9,78E-01 |
| P17809 | 9 | 4 | 26,56 | 20525 | Slc2a1 | 0 | 0,74 | 3,90E-01 |
| Q64704 | 14 | 4 | 3,24 | 20908 | Stx3 | 0 | 1,41 | 7,33E-01 |
| CAH2_HUMAN | 11 | 2 | 4,95 | | | 0 | 28,98 | 1,60E-16 |
| Q52KR3 | 2 | 4 | 21,96 | 353211 | Prune2 | 0 | 1,22 | 8,70E-01 |
| Q9WVA4 | 11 | 2 | 64,29 | 21346 | Tagln2 | 0 | 2,01 | 7,01E-05 |
| P59823 | 7 | 4 | 9,9 | | Il1rapl1 | 0 | 0,78 | 8,42E-01 |
| Q9WUR2 | 10 | 3 | 15,29 | 23986 | Eci2 | 0 | 0,86 | 9,16E-01 |
| Q62095 | 6 | 4 | 9,36 | 26900 | Ddx3y | 0 | 0,95 | 9,76E-01 |
| FABPH_HUMAN | 30 | 4 | 42,69 | | | 0 | 1,13 | 8,76E-01 |
| P63073 | 20 | 4 | 1,71 | 13684 | Eif4e | 0 | 1,05 | 9,88E-01 |
| P47941 | 17 | 3 | 3,6 | 12929 | Crkl | 0 | 1,02 | 1,00E+00 |
| Q9R0H0 | 6 | 3 | 19,38 | 11430 | Acox1 | 0 | 0,85 | 8,91E-01 |
| P70670 | 1 | 2 | 5,93 | 17938 | Naca | 0 | 1,85 | 4,83E-01 |
| Q60817 | 13 | 2 | 5,93 | 17938 | Naca | 0 | 1,85 | 4,83E-01 |
| P62331 | 27 | 3 | 16,42 | 11845 | Arf6 | 1 | 1,25 | 7,80E-01 |

| | | | | | | | | |
|---|---|---|---|---|---|---|---|---|
| Q8BGD5 | 5 | 3 | 2,5 | 78070 | Cpt1c | 0 | 1,10 | 9,76E-01 |
| Q9D6Y7 | 26 | 5 | 15,81 | 110265 | Msra | 0 | 1,36 | 5,97E-01 |
| Q9D7X8 | 21 | 4 | 5,57 | 110175 | Ggct | 0 | 0,98 | 9,91E-01 |
| Q8VHI6 | 7 | 3 | 19,89 | 245880 | Wasf3 | 0 | 1,24 | 7,86E-01 |
| Q8BG02 | 6 | 2 | 41,31 | 269643 | Ppp2r2c | 0 | 0,87 | 7,62E-01 |
| A2AHG0 | 6 | 4 | 10,41 | 241638 | Lzts3 | 0 | 1,55 | 3,95E-01 |
| Q9CYR6 | 8 | 4 | 15,38 | 109785 | Pgm3 | 0 | 0,91 | 9,61E-01 |
| Q99J08 | 13 | 5 | 3,53 | 67815 | Sec14l2 | 0 | 1,14 | 9,21E-01 |
| Q921H9 | 14 | 3 | 22,24 | 69893 | Coa7 | 0 | 1,02 | 1,00E+00 |
| Q8BK72 | 10 | 4 | 11,47 | 218506 | Mrps27 | 0 | 0,53 | 1,37E-01 |
| P61967 | 16 | 2 | 18,95 | 11769 | Ap1s1 | 0 | 1,41 | 8,07E-01 |
| Q8R0W0 | 2 | 2 | 9,75 | 223650 | Eppk1 | 0 | 1000,00 | 1,60E-16 |
| P51655 | 9 | 3 | 1,92 | 14735 | Gpc4 | 1 | 1,13 | 9,69E-01 |
| Q922D4 | 5 | 3 | 6,29 | 52036 | Ppp6r3 | 0 | 1,08 | 9,78E-01 |
| A2BDX3 | 10 | 3 | 5,12 | 69372 | Mocs3 | 0 | 0,99 | 9,85E-01 |
| Q9CQJ6 | 26 | 4 | 13,33 | 68184 | Denr | 0 | 1,01 | 1,00E+00 |
| Q9DC16 | 14 | 4 | 16,83 | 67458 | Ergic1 | 0 | 0,72 | 3,33E-01 |
| Q9DD18 | 15 | 2 | 13,46 | 66044 | Dtd1 | 0 | 2,06 | 1,28E-01 |
| O08688 | 10 | 5 | 0 | 12337 | Capn5 | 0 | 1,19 | 8,70E-01 |
| Q5XJV6 | 4 | 5 | 0 | 381983 | Lmtk3 | 0 | 0,77 | 8,34E-01 |
| Q640M6 | 6 | 3 | 7,27 | 233552 | Gdpd5 | 0 | 0,64 | 4,15E-01 |
| O88704 | 6 | 3 | 0 | 15165 | Hcn1 | 2 | 0,66 | 4,69E-01 |
| P97798 | 4 | 5 | 9,02 | | Neo1 | 0 | 1,07 | 9,91E-01 |
| Q9DCC4 | 11 | 2 | 25,74 | 66194 | Pycr3 | 0 | 0,77 | 6,32E-01 |
| Q9D1Q6 | 12 | 3 | 5,62 | 76299 | Erp44 | 0 | 0,82 | 8,54E-01 |
| Q3UGR5 | 15 | 4 | 29,15 | 76987 | Hdhd2 | 0 | 0,92 | 9,34E-01 |
| O54950 | 12 | 2 | 24,51 | 19082 | Prkag1 | 0 | 1,02 | 1,00E+00 |
| Q9D164 | 27 | 2 | 25,19 | 59095 | Fxyd6 | 0 | 1,98 | 2,91E-01 |
| A2ALU4 | 4 | 4 | 5,38 | 110380 | Shroom2 | 0 | 0,92 | 9,71E-01 |
| P70232 | 7 | 6 | 5,62 | 12661 | Chl1 | 0 | 1,13 | 9,67E-01 |
| Q59J78 | 27 | 4 | 2,3 | 75597 | Ndufaf2 | 0 | 0,73 | 6,09E-01 |
| P67871 | 13 | 3 | 29,02 | 13001 | Csnk2b | 0 | 1,27 | 6,23E-01 |
| Q6DFV3 | 3 | 4 | 9,91 | 71435 | Arhgap21 | 0 | 1,19 | 9,39E-01 |
| Q9CQZ6 | 18 | 3 | 51,03 | 66495 | Ndufb3 | 0 | 1,97 | 2,99E-04 |
| Q62351 | 6 | 4 | 2,51 | 22042 | Tfrc | 0 | 1,15 | 9,74E-01 |
| Q811I0 | 13 | 4 | 15,02 | | Atpaf1 | 0 | 0,96 | 9,76E-01 |
| Q80XG9 | 6 | 3 | 26,88 | 243499 | Lrrtm4 | 0 | 0,88 | 9,34E-01 |
| Q8BGY9 | 7 | 4 | 23,61 | 63993 | Slc5a7 | 0 | 0,90 | 9,34E-01 |
| Q9JIG8 | 22 | 3 | 32,01 | 54637 | Praf2 | 0 | 1,24 | 6,93E-01 |
| Q8K3G9 | 7 | 3 | 5,67 | 216190 | Appl2 | 0 | 0,68 | 6,58E-01 |
| O35083 | 18 | 4 | 19,33 | 55979 | Agpat1 | 0 | 0,85 | 8,93E-01 |
| Q8K3P0 | 11 | 4 | 7,98 | 70061 | Sdr9c7 | 0 | 1,22 | 8,85E-01 |
| Q91XL9 | 4 | 2 | 27 | 64291 | Osbpl1a | 1 | 1,04 | 9,85E-01 |
| Q9ESE1 | 1 | 4 | 9,26 | 80877 | Lrba | 0 | 1,16 | 9,34E-01 |
| Q3KNM2 | 10 | 2 | 21,19 | 69104 | Marchf5 | 0 | 1,10 | 9,76E-01 |
| P97952 | 19 | 4 | 7,88 | 20266 | Scn1b | 0 | 1,25 | 8,42E-01 |

| | | | | | | | | |
|---|---|---|---|---|---|---|---|---|
| OVAL_CHICK | 9 | 2 | 24,71 | 396058 | OVA; SERPINB14 | 0 | 0,31 | 2,63E-04 |
| Q6PDY2 | 10 | 2 | 29,52 | 211488 | Ado | 0 | 0,55 | 3,26E-01 |
| Q9D6K5 | 23 | 3 | 33,36 | 105940408; 24071 | Synj2bp | 0 | 0,94 | 9,74E-01 |
| P97823 | 14 | 3 | 16,79 | 18777 | Lypla1 | 0 | 1,62 | 4,21E-02 |
| O08842 | 7 | 3 | 16,42 | 14586 | Gfra2 | 0 | 0,94 | 9,74E-01 |
| Q8BMI3 | 9 | 5 | 0 | 260302 | Gga3 | 0 | 1,42 | 6,61E-01 |
| P62192 | 8 | 2 | 17,35 | 19179 | Psmc1 | 0 | 0,27 | 2,51E-03 |
| Q9Z2D0 | 8 | 5 | 9,11 | 210376 | Mtmr9 | 0 | 0,90 | 9,56E-01 |
| Q9WVG6 | 7 | 4 | 13,28 | 59035 | Carm1 | 0 | 1,07 | 9,79E-01 |
| Q922E4 | 11 | 4 | 7,43 | 68671 | Pcyt2 | 0 | 1,32 | 7,49E-01 |
| Q8BG18 | 6 | 2 | 10,78 | 69352 | Necab1 | 0 | 0,73 | 7,77E-01 |
| Q91WG5 | 7 | 3 | 14,83 | 108099 | Prkag2 | 1 | 0,96 | 9,76E-01 |
| Q9JIY5 | 8 | 3 | 5,47 | 64704 | Htra2 | 0 | 0,68 | 6,27E-01 |
| Q99KV1 | 14 | 3 | 6,22 | 67838 | Dnajb11 | 0 | 1,27 | 8,70E-01 |
| Q8BGN3 | 8 | 4 | 9,94 | 320981 | Enpp6 | 0 | 0,97 | 9,78E-01 |
| Q9CPW4 | 21 | 2 | 29,31 | 67771 | Arpc5 | 0 | 0,81 | 7,52E-01 |
| Q8VHL1 | 11 | 3 | 19,88 | 73251 | Setd7 | 0 | 0,78 | 7,18E-01 |
| P97467 | 6 | 6 | 8,66 | 18484 | Pam | 0 | 1,20 | 8,85E-01 |
| Q8K377 | 6 | 3 | 13,95 | 74342 | Lrrtm1 | 0 | 0,98 | 9,85E-01 |
| Q9DBL1 | 10 | 5 | 13,08 | 66885 | Acadsb | 0 | 0,63 | 4,21E-02 |
| D3YZU1 | 3 | 3 | 10,61 | 243961 | Shank1 | 0 | 0,95 | 9,77E-01 |
| Q8R1I1 | 38 | 2 | 22,72 | 66152 | Uqcr10 | 0 | 1,12 | 9,47E-01 |
| Q8BGC4 | 10 | 3 | 10,41 | 225791 | Zadh2 | 0 | 0,76 | 7,28E-01 |
| P68040 | 14 | 4 | 8,16 | 14694 | Rack1 | 0 | 0,83 | 8,57E-01 |
| Q5DU41 | 4 | 3 | 6,15 | 433926 | Lrrc8b | 0 | 1,31 | 8,07E-01 |
| Q60870 | 10 | 2 | 31,79 | | Reep5 | 0 | 1,26 | 6,42E-01 |
| P62869 | 47 | 4 | 17,55 | 67673 | Elob | 0 | 1,33 | 5,89E-01 |
| Q8K0C9 | 11 | 3 | 15,08 | 218138 | Gmds | 0 | 1,05 | 9,83E-01 |
| Q8BIK4 | 3 | 5 | 3,25 | | Dock9 | 0 | 1,08 | 9,79E-01 |
| Q3UVK0 | 5 | 4 | 2,13 | 226090 | Ermp1 | 0 | 0,83 | 9,17E-01 |
| B1AS29 | 5 | 3 | 1,66 | 14807 | Grik3 | 0 | 1,46 | 6,63E-01 |
| Q8CI78 | 9 | 4 | 7,48 | 66084 | Rmnd1 | 0 | 0,91 | 9,56E-01 |
| Q8R4G0 | 6 | 4 | 18,92 | 80883 | Ntng1 | 0 | 1,00 | 9,96E-01 |
| Q8BGR6 | 15 | 3 | 21,56 | 218639 | Arl15 | 0 | 0,92 | 9,56E-01 |
| Q9ERL9 | 7 | 4 | 6,3 | 60596 | Gucy1a1 | 0 | 0,76 | 7,51E-01 |
| O70166 | 16 | 4 | 28,55 | 20262 | Stmn3 | 0 | 1,14 | 9,12E-01 |
| Q8BGY7 | 12 | 3 | 34,11 | 108654 | Fam210a | 0 | 0,85 | 7,86E-01 |
| Q3V038 | 17 | 3 | 16,27 | 69480 | Ttc9 | 0 | 0,91 | 9,59E-01 |
| Q8BWY3 | 9 | 4 | 6,42 | 225363 | Etf1 | 0 | 0,65 | 3,57E-01 |
| Q9D2Q8 | 21 | 2 | 19 | 66166 | S100a14 | 0 | 1,44 | 2,26E-01 |
| Q5HZI2 | 13 | 4 | 3,85 | 237397 | C2cd4c | 0 | 1,13 | 9,56E-01 |
| Q8CGC7 | 3 | 4 | 1,99 | 107508 | Eprs1 | 0 | 0,81 | 8,97E-01 |
| P60521 | 28 | 4 | 21,39 | 93739 | Gabarapl2 | 0 | 0,99 | 9,85E-01 |

| | | | | | | | | |
|---|---|---|---|---|---|---|---|---|
| Q7TNF0 | 7 | 2 | 14,16 | 13446 | Doc2a | 0 | 1,29 | 8,52E-01 |
| B1AY13 | 2 | 4 | 2,46 | 329908 | Usp24 | 0 | 0,95 | 9,76E-01 |
| Q62059 | 1 | 3 | 8,06 | | Vcan | 0 | 1,83 | 1,88E-01 |
| Q8CG76 | 10 | 3 | 22,52 | 110198 | Akr7a2 | 0 | 1,20 | 9,22E-01 |
| Q8R3B1 | 7 | 4 | 4,55 | 18799 | Plcd1 | 0 | 0,56 | 3,85E-01 |
| Q5SUC9 | 11 | 3 | 14,48 | 52892 | Sco1 | 0 | 0,93 | 9,56E-01 |
| O55135 | 17 | 2 | 2,92 | 16418 | Eif6 | 0 | 0,00 | 1,60E-16 |
| Q80WQ2 | 5 | 5 | 10,2 | 234729 | Vac14 | 0 | 1,03 | 9,85E-01 |
| Q91W90 | 10 | 4 | 1,69 | 105245 | Txndc5 | 0 | 0,84 | 9,18E-01 |
| Q9QUG9 | 4 | 2 | 10,54 | 19395 | Rasgrp2 | 0 | 0,97 | 9,78E-01 |
| Q61418 | 4 | 2 | 16,61 | 12727 | Clcn4 | 0 | 0,80 | 8,85E-01 |
| Q9JKF7 | 15 | 5 | 3,32 | 27393 | Mrpl39 | 0 | 0,65 | 4,67E-01 |
| Q60759 | 8 | 3 | 19,81 | 270076 | Gcdh | 0 | 1,07 | 9,76E-01 |
| P51830 | 4 | 5 | 1,87 | 11515 | Adcy9 | 0 | 0,85 | 8,43E-01 |
| Q8R146 | 8 | 4 | 3,28 | 235606 | Apeh | 0 | 0,87 | 9,23E-01 |
| O55003 | 18 | 3 | 4,47 | 12176 | Bnip3 | 0 | 0,65 | 4,82E-01 |
| Q91W86 | 6 | 5 | 10,63 | 71732 | Vps11 | 0 | 0,94 | 9,74E-01 |
| Q9D6U8 | 16 | 2 | 10,7 | 70186 | Fam162a | 0 | 0,84 | 8,53E-01 |
| Q8BPM0 | 3 | 4 | 6,1 | 208846 | Daam1 | 0 | 1,01 | 9,99E-01 |
| Q9Z2Y8 | 15 | 5 | 10,88 | 114863 | Plpbp | 0 | 1,08 | 9,76E-01 |
| P03930 | 34 | 2 | 40,23 | 17706 | Mtatp8 | 0 | 1,05 | 9,87E-01 |
| Q6RHR9 | 3 | 3 | 9,7 | 14924 | Magi1 | 0 | 1,10 | 9,76E-01 |
| Q8K009 | 3 | 3 | 17,08 | 216188 | Aldh1l2 | 0 | 0,89 | 8,70E-01 |
| P32020 | 7 | 4 | 5,49 | 20280 | Scp2 | 0 | 0,96 | 9,76E-01 |
| P61022 | 16 | 3 | 24,33 | 56398 | Chp1 | 0 | 1,12 | 9,61E-01 |
| Q9QZC2 | 2 | 3 | 33,64 | 54712 | Plxnc1 | 0 | 0,83 | 8,78E-01 |
| Q9EPU0 | 5 | 5 | 1,8 | 19704 | Upf1 | 0 | 0,81 | 8,57E-01 |
| P22933 | 12 | 4 | 3,74 | 14403 | Gabrd | 0 | 1,23 | 8,91E-01 |
| P97461 | 18 | 3 | 16,8 | 20103 | Rps5 | 0 | 0,94 | 9,74E-01 |
| Q8BG40 | 9 | 4 | 2,19 | 74187 | Katnb1 | 0 | 1,67 | 4,27E-01 |
| Q9JM13 | 7 | 3 | 5,6 | 56715 | Rabgef1 | 0 | 1,18 | 9,48E-01 |
| P52825 | 5 | 3 | 6,67 | 12896 | Cpt2 | 0 | 0,70 | 1,11E-01 |
| E9Q4Z2 | 2 | 4 | 1,85 | 100705 | Acacb | 0 | 2,32 | 3,09E-02 |
| Q3UTH8 | 6 | 3 | 14,15 | 236915 | Arhgef9 | 0 | 1,00 | 9,91E-01 |
| Q8VEJ9 | 12 | 5 | 1,74 | 116733 | Vps4a | 0 | 1,01 | 1,00E+00 |
| O35607 | 3 | 2 | 11,34 | 12168 | Bmpr2 | 0 | 0,91 | 9,50E-01 |
| Q9CYW4 | 16 | 4 | 19,97 | 72748 | Hdhd3 | 0 | 0,59 | 2,71E-02 |
| Q8BR63 | 15 | 3 | 15 | 100101807; 73385 | Fam177a1 | 0 | 0,97 | 9,76E-01 |
| P46978 | 5 | 4 | 1,98 | 16430 | Stt3a | 0 | 1,11 | 9,33E-01 |
| Q9CXW4 | 13 | 2 | 10,18 | 67025 | Rpl11 | 0 | 0,84 | 8,70E-01 |
| O88602 | 11 | 2 | 21,88 | 12300 | Cacng2 | 1 | 0,63 | 1,36E-01 |
| P10518 | 12 | 3 | 18,35 | 17025 | Alad | 0 | 0,78 | 7,73E-01 |
| P16283 | 6 | 6 | 0 | 20536 | Slc4a3 | 0 | 0,70 | 6,86E-01 |
| Q99MB7 | 14 | 3 | 10,94 | 67150 | Rnf141 | 0 | 1,40 | 6,97E-01 |
| P38060 | 11 | 3 | 12,77 | 15356 | Hmgcl | 0 | 0,55 | 1,81E-01 |

| | | | | | | | | |
|---|---|---|---|---|---|---|---|---|
| Q61923 | 6 | 2 | 27,94 | 16494 | Kcna6 | 0 | 1,19 | 9,39E-01 |
| Q62132 | 7 | 3 | 7,09 | 19279 | Ptprr | 0 | 1,15 | 9,54E-01 |
| Q9WV98 | 39 | 3 | 3,67 | 30056 | Timm9 | 0 | 1,05 | 9,84E-01 |
| Q9D6K8 | 16 | 3 | 5,76 | 67391 | Fundc2 | 0 | 1,29 | 8,91E-01 |
| Q80XA6 | 10 | 3 | 6,25 | 194590 | Reps2 | 1 | 1,23 | 8,91E-01 |
| Q8CGQ8 | 5 | 3 | 5,42 | 238384 | Slc24a4 | 0 | 0,89 | 8,85E-01 |
| Q923D2 | 17 | 3 | 4,21 | 233016 | Blvrb | 0 | 0,93 | 9,72E-01 |
| Q61626 | 4 | 3 | 4,13 | 14809 | Grik5 | 0 | 0,79 | 8,81E-01 |
| Q8R4F1 | 5 | 3 | 5,04 | 171171 | Ntng2 | 0 | 0,82 | 8,56E-01 |
| Q69ZF3 | 4 | 3 | 10,57 | 230101 | Gba2 | 0 | 0,86 | 9,16E-01 |
| Q99LF4 | 7 | 3 | 9,4 | 28088 | Rtcb | 0 | 0,76 | 7,41E-01 |
| Q9WVL0 | 11 | 2 | 14,28 | 14874 | Gstz1 | 0 | 1,20 | 9,13E-01 |
| Q9CQ19 | 17 | 3 | 8,9 | 98932 | Myl9 | 0 | 1,08 | 9,74E-01 |
| Q8VHQ9 | 11 | 5 | 5,04 | 329910 | Acot11 | 0 | 1,24 | 7,89E-01 |
| Q8VCL2 | 10 | 2 | 14,56 | 100126824 | Sco2 | 0 | 0,78 | 8,35E-01 |
| Q9CZB0 | 22 | 3 | 8,37 | 66052 | Sdhc | 0 | 0,63 | 2,65E-01 |
| Q8C9H6 | 6 | 3 | 16,22 | 320609 | Strip2 | 1 | 1,28 | 6,98E-01 |
| Q9JHG6 | 10 | 2 | 14,71 | 54720 | Rcan1 | 0 | 0,59 | 2,86E-01 |
| Q9DBS1 | 9 | 3 | 16,46 | 74122 | Tmem43 | 0 | 1,02 | 9,99E-01 |
| Q8BFQ8 | 14 | 3 | 16,16 | 213350 | Gatd1 | 0 | 1,12 | 9,69E-01 |
| Q6PEB6 | 16 | 3 | 8,59 | 19070 | Mob4 | 0 | 0,84 | 8,41E-01 |
| P35290 | 17 | 3 | 20,9 | 19336 | Rab24 | 0 | 1,56 | 3,64E-01 |
| P56546 | 7 | 4 | 44,82 | 13017 | Ctbp2 | 0 | 1,25 | 6,07E-01 |
| Q8VE88 | 5 | 2 | 13,33 | 67726 | Fam114a2 | 0 | 0,71 | 6,10E-01 |
| P21995 | 9 | 2 | 12,97 | 13723 | Emb | 0 | 1,08 | 9,76E-01 |
| Q9WTL7 | 18 | 4 | 27,6 | 26394 | Lypla2 | 0 | 1,18 | 8,17E-01 |
| Q3URE9 | 4 | 2 | 18,34 | 242384 | Lingo2 | 0 | 1,98 | 2,26E-03 |
| Q6P2B1 | 6 | 5 | 5,02 | 320938 | Tnpo3 | 0 | 0,81 | 7,67E-01 |
| Q91V36 | 8 | 4 | 4,95 | | Nrbp2 | 0 | 0,96 | 9,76E-01 |
| Q8VBY2 | 6 | 2 | 3,02 | 55984 | Camkk1 | 0 | 0,00 | 1,60E-16 |
| Q61425 | 9 | 3 | 13,02 | 15107 | Hadh | 0 | 0,60 | 2,86E-01 |
| Q8K4Z3 | 13 | 3 | 12,1 | 246703 | Naxe | 0 | 0,96 | 9,74E-01 |
| Q921E2 | 16 | 3 | 10,46 | 106572 | Rab31 | 0 | 0,73 | 6,59E-01 |
| Q6PDC8 | 5 | 2 | 6,84 | 213006 | Mfsd4a | 0 | 22,23 | 1,60E-16 |
| Q7TT45 | 8 | 3 | 5,57 | 52187 | Rragd | 0 | 1,14 | 8,85E-01 |
| Q8BZA9 | 9 | 2 | 8,5 | 319801 | Tigar | 0 | 2,22 | 5,23E-02 |
| Q3UH93 | 2 | 3 | 28,79 | 67784 | Plxnd1 | 0 | 2,27 | 1,22E-01 |
| Q91W50 | 5 | 5 | 1,69 | 229663 | Csde1 | 0 | 0,87 | 8,17E-01 |
| P47791 | 5 | 2 | 34,09 | 14782 | Gsr | 0 | 0,89 | 9,16E-01 |
| Q8BXT1 | 18 | 3 | 8,29 | 67792 | Rgs8 | 0 | 0,69 | 6,06E-01 |
| O08586 | 8 | 4 | 10,78 | 19211 | Pten | 0 | 1,35 | 6,42E-01 |
| O88703 | 6 | 2 | 1,61 | 15166 | Hcn2 | 0 | 0,00 | 1,60E-16 |
| Q8VE62 | 7 | 4 | 10,95 | 218693 | Paip1 | 0 | 1,21 | 8,82E-01 |
| P97492 | 6 | 3 | 22,4 | 51791 | Rgs14 | 0 | 0,93 | 9,74E-01 |
| Q9JL62 | 12 | 2 | 12,2 | 56356 | Gltp | 0 | 0,74 | 7,90E-01 |
| Q5Y4Y6 | 5 | 3 | 17,95 | 450219 | Gsdma3 | 0 | 0,94 | 9,59E-01 |

| | | | | | | | | |
|---|---|---|---|---|---|---|---|---|
| Q9WVP1 | 7 | 3 | 32,03 | 11768 | Ap1m2 | 0 | 0,76 | 5,29E-01 |
| Q3ULF4 | 4 | 3 | 6,68 | 234847 | Spg7 | 0 | 0,71 | 6,42E-01 |
| A2AP18 | 3 | 5 | 3,7 | 269615 | Plch2 | 0 | 0,61 | 1,82E-01 |
| Q06335 | 7 | 3 | 8,88 | 11804 | Aplp2 | 0 | 1,42 | 1,91E-01 |
| Q8BHJ7 | 6 | 2 | 26,29 | 110886 | Gabra5 | 0 | 1,17 | 8,37E-01 |
| O35215 | 22 | 2 | 5,44 | 13202 | Ddt | 0 | 1,27 | 9,02E-01 |
| Q923L3 | 1 | 3 | 1,63 | 94109 | Csmd1 | 1 | 1,47 | 6,61E-01 |
| Q8R4I7 | 10 | 3 | 7,13 | 246317 | Neto1 | 0 | 0,98 | 9,85E-01 |
| Q9JK42 | 10 | 3 | 9,95 | 18604 | Pdk2 | 0 | 1,07 | 9,76E-01 |
| P69566 | 4 | 2 | 6,22 | 56705 | Ranbp9 | 0 | 1,16 | 9,48E-01 |
| Q9DB27 | 12 | 2 | 9,32 | 68995 | Mcts1 | 0 | 1,70 | 2,12E-01 |
| Q9Z2D1 | 7 | 4 | 5,16 | 77116 | Mtmr2 | 0 | 1,25 | 9,02E-01 |
| P35505 | 8 | 3 | 14,34 | 14085 | Fah | 0 | 0,80 | 7,99E-01 |
| Q80Y14 | 17 | 2 | 2,89 | 73046 | Glrx5 | 0 | 0,86 | 9,45E-01 |
| Q61334 | 10 | 2 | 12,31 | 12033 | Bcap29 | 0 | 1,14 | 9,71E-01 |
| Q6ZQ82 | 3 | 2 | 16,77 | 71302 | Arhgap26 | 0 | 1,25 | 8,85E-01 |
| Q9D428 | 14 | 3 | 8,16 | 71146 | GOLGA7 B | 0 | 1,54 | 4,13E-01 |
| Q8C1Y8 | 6 | 2 | 20,3 | 231874 | Ccz1 | 0 | 0,96 | 9,76E-01 |
| Q8K0D0 | 7 | 2 | 33,32 | 237459 | Cdk17 | 1 | 1,34 | 8,07E-01 |
| Q04735 | 8 | 2 | 33,32 | 18555 | Cdk16 | 0 | 1,34 | 8,07E-01 |
| Q0VBD0 | 5 | 4 | 5,74 | 320910 | Itgb8 | 0 | 1,49 | 5,91E-01 |
| Q8JZR6 | 3 | 2 | 9,44 | 59033 | Slc4a8 | 0 | 0,47 | 1,55E-01 |
| Q6NXK8 | 6 | 3 | 14,85 | 11419 | Asic1 | 0 | 1,07 | 9,76E-01 |
| Q7TNG5 | 7 | 3 | 8,12 | 72205 | Eml2 | 0 | 1,74 | 4,33E-01 |
| O54916 | 5 | 2 | 6,09 | 19707 | Reps1 | 0 | 1,25 | 9,25E-01 |
| Q6NS82 | 11 | 3 | 4,62 | 227298 | Retreg2 | 0 | 1,14 | 9,71E-01 |
| Q9WV07 | 5 | 3 | 8,09 | 23801 | Aloxe3 | 0 | 1,48 | 6,46E-01 |
| Q8R5J9 | 14 | 3 | 22,01 | 65106 | Arl6ip5 | 0 | 1,19 | 7,77E-01 |
| Q99KF1 | 17 | 3 | 20,88 | 67511 | Tmed9 | 0 | 2,28 | 1,21E-06 |
| P97447 | 11 | 3 | 18,5 | 14199 | Fhl1 | 0 | 1,74 | 2,16E-02 |
| Q9EPR4 | 6 | 3 | 2,06 | 54338 | Slc23a2 | 0 | 0,74 | 7,12E-01 |
| P61963 | 13 | 4 | 5,46 | 71833 | Dcaf7 | 0 | 0,86 | 9,11E-01 |
| Q9JLT4 | 4 | 2 | 14,18 | 26462 | Txnrd2 | 0 | 0,78 | 6,66E-01 |
| Q8JZR0 | 3 | 2 | 40,58 | 433256 | Acsl5 | 0 | 0,79 | 6,58E-01 |
| Q8VI75 | 4 | 3 | 2,25 | 75751 | Ipo4 | 0 | 1,10 | 9,76E-01 |
| Q80VC9 | 6 | 4 | 0 | 69697 | Camsap3 | 0 | 1,54 | 6,94E-01 |
| O35114 | 8 | 4 | 9,41 | 12492 | Scarb2 | 0 | 0,75 | 2,68E-01 |
| Q8BTG3 | 7 | 3 | 10,37 | 320554 | Tcp11l1 | 0 | 1,55 | 5,85E-01 |
| Q9JI39 | 4 | 3 | 2,07 | 56199 | Abcb10 | 0 | 0,91 | 9,59E-01 |
| Q8JZW5 | 10 | 4 | 0 | 230863 | Sh2d5 | 0 | 0,71 | 6,52E-01 |
| O88958 | 11 | 3 | 9,26 | 26384 | Gnpda1 | 0 | 1,09 | 9,74E-01 |
| Q8BUY5 | 13 | 3 | 4,39 | 76916 | Timmdc1 | 0 | 0,63 | 5,80E-01 |
| Q9Z0L0 | 7 | 3 | 8,55 | 21983 | Tpbg | 0 | 0,85 | 8,93E-01 |
| P53702 | 10 | 2 | 10,16 | 15159 | Hccs | 0 | 0,00 | 1,60E-16 |
| Q9CRB8 | 21 | 3 | 25,71 | 67900 | Mtfp1 | 0 | 0,98 | 9,78E-01 |
| Q80ZW2 | 17 | 4 | 7,58 | 223626 | Them6 | 0 | 1,39 | 7,52E-01 |

| | | | | | | | | |
|---|---|---|---|---|---|---|---|---|
| Q14C51 | 5 | 3 | 5,4 | 69956 | Ptcd3 | 0 | 0,81 | 8,41E-01 |
| Q9QZB9 | 12 | 2 | 9,18 | 59288 | Dctn5 | 0 | 0,94 | 9,76E-01 |
| Q3TRJ4 | 5 | 3 | 41,58 | 320864 | Krt26 | 0 | 1,61 | 4,64E-02 |
| Q9ET30 | 5 | 3 | 13,19 | 107358 | Tm9sf3 | 0 | 1,05 | 9,76E-01 |
| P24547 | 6 | 2 | 9,86 | 23918 | Impdh2 | 1 | 1,14 | 9,13E-01 |
| Q9CY18 | 5 | 2 | 5,66 | | Snx7 | 0 | 0,72 | 6,74E-01 |
| Q3UMB5 | 4 | 4 | 1,76 | 237782 | Smcr8 | 0 | 1,08 | 9,85E-01 |
| Q9D8Y1 | 15 | 3 | 29,04 | 66271 | Tmem126 a | 0 | 0,70 | 1,22E-01 |
| Q9CQI6 | 23 | 4 | 39,08 | 72042 | Cotl1 | 0 | 0,92 | 9,12E-01 |
| P00158 | 7 | 3 | 32,84 | 17711 | Mt-Cyb | 0 | 0,77 | 4,00E-01 |
| Q61249 | 7 | 2 | 8,35 | 18518 | Igbp1 | 0 | 1,22 | 9,09E-01 |
| O55229 | 9 | 2 | 5,44 | 12651 | Chkb | 0 | 1,34 | 8,44E-01 |
| Q8BGT1 | 5 | 3 | 4,3 | 71436 | Flrt3 | 0 | 1,00 | 9,85E-01 |
| Q99M51 | 11 | 3 | 0 | 17973 | Nck1 | 0 | 0,90 | 9,66E-01 |
| Q6NVF0 | 3 | 3 | 2,16 | 320634 | Ocrl | 0 | 1,56 | 7,23E-01 |
| Q8K4I3 | 3 | 2 | 22,69 | 73341 | Arhgef6 | 0 | 0,84 | 8,36E-01 |
| P17047 | 6 | 3 | 9,14 | 16784 | Lamp2 | 0 | 0,79 | 7,97E-01 |
| Q91VT4 | 11 | 2 | 5,68 | 234309 | Cbr4 | 0 | 0,80 | 8,12E-01 |
| Q9D415 | 3 | 2 | 5,85 | 224997 | Dlgap1 | 0 | 1,40 | 7,86E-01 |
| Q921W4 | 10 | 3 | 4,02 | 66609 | Cryzl1 | 0 | 1,08 | 9,78E-01 |
| Q3URE1 | 4 | 2 | 10,82 | 257633 | Acsf3 | 0 | 1,39 | 7,63E-01 |
| Q8BWR2 | 12 | 2 | 7,64 | 66193 | Pithd1 | 0 | 1,14 | 9,02E-01 |
| Q9DCZ1 | 12 | 4 | 3,73 | 66355 | Gmpr | 0 | 0,86 | 7,52E-01 |
| Q8BL66 | 2 | 3 | 1,87 | 216238 | Eea1 | 0 | 1,40 | 7,49E-01 |
| Q3TVA9 | 2 | 2 | 4,42 | 232664 | Ccdc136 | 0 | 0,63 | 6,23E-01 |
| Q9CYN9 | 6 | 2 | 15,17 | 70495 | Atp6ap2 | 0 | 1,24 | 7,50E-01 |
| P61620 | 6 | 2 | 22,64 | 53421 | Sec61a1 | 1 | 1,24 | 6,93E-01 |
| Q9D832 | 11 | 3 | 4,87 | 67035 | Dnajb4 | 0 | 1,23 | 9,09E-01 |
| P54830 | 7 | 4 | 13,87 | 19259 | Ptpn5 | 0 | 0,82 | 6,07E-01 |
| Q64291 | 6 | 2 | 4,75 | | Krt12 | 0 | 160,05 | 1,60E-16 |
| Q9D1P4 | 16 | 4 | 0 | 66917 | Chordc1 | 0 | 1,12 | 9,76E-01 |
| Q8QZV4 | 6 | 2 | 6,61 | 57740 | Stk32c | 0 | 0,91 | 9,71E-01 |
| Q3TUH1 | 11 | 3 | 16,2 | | Tamm41 | 0 | 0,94 | 9,76E-01 |
| Q8BGX3 | 7 | 3 | 1,99 | 211187 | Lrtm2 | 0 | 0,91 | 9,70E-01 |
| P97792 | 10 | 4 | 11,05 | 13052 | Cxadr | 0 | 1,05 | 9,85E-01 |
| Q91VR7 | 17 | 2 | 7,59 | 66734 | Map1lc3a | 0 | 0,75 | 2,74E-01 |
| Q6ZPR4 | 5 | 4 | 1,96 | 227632 | Kcnt1 | 0 | 0,72 | 8,12E-01 |
| Q9EPB7 | 11 | 2 | 22,56 | 64378 | Gpr88 | 0 | 1,01 | 1,00E+00 |
| P70236 | 11 | 3 | 6,04 | 26399 | Map2k6 | 0 | 1,28 | 9,02E-01 |
| Q8JZU2 | 8 | 2 | 15,23 | 13358 | Slc25a1 | 0 | 1,00 | 9,96E-01 |
| Q6P4S6 | 2 | 3 | 15,11 | 70661 | Sik3 | 0 | 1,13 | 9,34E-01 |
| Q8BHH2 | 11 | 2 | 17 | 319642 | Rab9b | 0 | 1,34 | 4,34E-01 |
| P83940 | 20 | 2 | 9,1 | 67923 | Eloc | 0 | 0,95 | 9,76E-01 |
| O55091 | 15 | 3 | 0 | 16210 | Impact | 0 | 1,55 | 5,36E-01 |
| Q9CQX5 | 11 | 2 | 15,44 | 224250 | Cldnd1 | 0 | 0,73 | 6,59E-01 |
| Q9JI19 | 6 | 2 | 12,62 | 58249 | Fibp | 0 | 1,05 | 9,94E-01 |

| | | | | | | | | |
|---|---|---|---|---|---|---|---|---|
| Q99KN9 | 5 | 3 | 4,71 | | Clint1 | 0 | 1,36 | 7,89E-01 |
| P59281 | 4 | 4 | 5,49 | 223666 | Arhgap39 | 0 | 1,03 | 1,00E+00 |
| Q3TCJ1 | 5 | 2 | 8,18 | 109359 | Abraxas2 | 0 | 1,08 | 9,76E-01 |
| A2ADY9 | 8 | 3 | 7,33 | 68817 | Ddi2 | 0 | 1,11 | 9,76E-01 |
| Q91Z38 | 8 | 2 | 10,58 | 66827 | Ttc1 | 0 | 2,02 | 2,62E-01 |
| Q78IK4 | 16 | 3 | 8,07 | 68117 | Apool | 0 | 0,90 | 9,71E-01 |
| Q9DCT1 | 9 | 2 | 21,94 | 56043 | Akr1e2 | 0 | 0,95 | 9,76E-01 |
| P62627 | 23 | 2 | 2,02 | 67068 | Dynlrb1 | 0 | 0,52 | 2,38E-01 |
| P61759 | 14 | 3 | 9,49 | 22327 | Vbp1 | 0 | 1,12 | 9,70E-01 |
| Q01149 | 2 | 3 | 13,56 | 12843 | Col1a2 | 0 | 0,76 | 7,41E-01 |
| Q8BGA3 | 5 | 3 | 11,2 | 107065 | Lrrtm2 | 0 | 0,75 | 7,38E-01 |
| Q99JG2 | 6 | 3 | 17,52 | 171469 | Gpr37l1 | 0 | 1,10 | 9,41E-01 |
| Q6ZWX6 | 6 | 2 | 23,56 | 13665 | Eif2s1 | 0 | 1,01 | 1,00E+00 |
| Q5XKN4 | 13 | 2 | 1,91 | 67767 | Jagn1 | 0 | 1,46 | 7,37E-01 |
| Q99J77 | 9 | 2 | 6,84 | 94181 | Nans | 0 | 0,90 | 9,56E-01 |
| P19639 | 11 | 3 | 28,37 | 14864 | Gstm3 | 0 | 1,02 | 9,99E-01 |
| Q9QZS3 | 7 | 2 | 8,17 | 18222 | Numb | 0 | 1,45 | 6,37E-01 |
| Q9WUC3 | 15 | 2 | 6,41 | 23934 | Ly6h | 0 | 0,06 | 1,60E-16 |
| P97324 | 9 | 4 | 0 | 14380 | G6pd2 | 0 | 0,93 | 9,70E-01 |
| Q91YM4 | 6 | 3 | 2,5 | 21379 | Tbrg4 | 0 | 0,74 | 7,74E-01 |
| P23492 | 15 | 3 | 9,52 | 18950 | Pnp | 0 | 0,94 | 9,74E-01 |
| Q9JIZ9 | 10 | 4 | 1,69 | 70310 | Plscr3 | 0 | 0,85 | 7,17E-01 |
| Q8R123 | 7 | 3 | 14,28 | 319945 | Flad1 | 0 | 1,19 | 9,22E-01 |
| Q9ER72 | 4 | 3 | 1,85 | 27267 | Cars1 | 0 | 1,04 | 1,00E+00 |
| Q9D154 | 6 | 2 | 6,33 | 66222 | Serpinb1a | 0 | 0,83 | 6,97E-01 |
| Q8CG72 | 6 | 2 | 10,43 | 100206 | Adprs | 0 | 1,04 | 1,00E+00 |
| Q2TPA8 | 6 | 3 | 9,06 | 72479 | Hsdl2 | 0 | 0,63 | 3,86E-01 |
| Q3UUI3 | 16 | 3 | 4,28 | 75778 | Them4 | 0 | 0,55 | 1,93E-01 |
| Q91VK4 | 16 | 3 | 3,81 | 64294 | Itm2c | 0 | 0,97 | 9,76E-01 |
| Q91V57 | 6 | 2 | 3,59 | 108699 | Chn1 | 0 | 1,22 | 9,60E-01 |
| Q8BYH7 | 4 | 2 | 9,65 | 233204 | Tbc1d17 | 0 | 1,02 | 1,00E+00 |
| Q8BFY6 | 8 | 3 | 10,54 | 67898 | Pef1 | 0 | 0,58 | 2,91E-01 |
| P58021 | 3 | 2 | 13,52 | 68059 | Tm9sf2 | 0 | 0,92 | 9,74E-01 |
| Q9CQE1 | 12 | 3 | 12,09 | 66536 | Nipsnap3 b | 0 | 1,10 | 9,56E-01 |
| Q8BIG7 | 8 | 2 | 24,65 | 69156 | Comtd1 | 0 | 0,92 | 9,25E-01 |
| O54781 | 5 | 3 | 12,85 | 20817 | Srpk2 | 0 | 1,12 | 9,76E-01 |
| Q9JKX6 | 14 | 3 | 10,06 | 53893 | Nudt5 | 0 | 1,68 | 5,07E-01 |
| Q5NCE8 | 5 | 2 | 14 | 380836 | Mrs2 | 0 | 0,77 | 7,77E-01 |
| Q8BJ03 | 5 | 2 | 12,92 | 226139 | Cox15 | 0 | 0,71 | 7,33E-01 |
| Q91VM3 | 7 | 2 | 6,02 | 54636 | Wdr45 | 0 | 1,59 | 5,21E-01 |
| P47758 | 13 | 2 | 4,45 | 20818 | Srprb | 0 | 0,00 | 1,60E-16 |
| Q91V76 | 8 | 2 | 5,86 | | | 0 | 0,94 | 9,76E-01 |
| O35495 | 7 | 2 | 31,59 | 18647 | Cdk14 | 0 | 1,47 | 7,13E-01 |
| Q9R1C6 | 4 | 2 | 6,93 | 56077 | Dgke | 0 | 0,89 | 9,59E-01 |
| P51791 | 4 | 3 | 10,45 | 12725 | Clcn3 | 1 | 0,72 | 2,37E-01 |
| Q7TMS5 | 3 | 2 | 12,11 | 26357 | Abcg2 | 0 | 0,67 | 5,32E-01 |

| | | | | | | | | |
|---|---|---|---|---|---|---|---|---|
| D3YZP9 | 9 | 4 | 0 | 76551 | Ccdc6 | 0 | 1,33 | 8,07E-01 |
| Q9WTP6 | 15 | 3 | 10,01 | 11637 | Ak2 | 0 | 1,15 | 9,56E-01 |
| Q2TBE6 | 8 | 4 | 3,36 | 84095 | Pi4k2a | 0 | 1,03 | 1,00E+00 |
| Q6PE15 | 9 | 3 | 3,74 | 213012 | Abhd10 | 0 | 1,63 | 4,51E-01 |
| Q3UHG7 | 9 | 3 | 0 | | Dennd11 | 0 | 0,76 | 8,09E-01 |
| Q03173 | 5 | 4 | 9,25 | 13800 | Enah | 0 | 0,97 | 9,78E-01 |
| O08576 | 4 | 2 | 12,77 | 51799 | Rundc3a | 0 | 0,81 | 8,85E-01 |
| Q7TSV4 | 6 | 4 | 0 | 66681 | Pgm2 | 0 | 1,25 | 9,23E-01 |
| Q9JM63 | 7 | 2 | 9,53 | 16513 | Kcnj10 | 0 | 0,98 | 9,88E-01 |
| Q5U4E0 | 12 | 3 | 1,85 | 269788 | Lhfpl4 | 0 | 1,20 | 9,16E-01 |
| O55137 | 5 | 2 | 11,11 | 26897 | Acot1 | 0 | 0,97 | 9,76E-01 |
| Q9QYR9 | 5 | 2 | 11,11 | 171210 | Acot2 | 0 | 0,97 | 9,76E-01 |
| Q8BX10 | 11 | 3 | 12,25 | 72542 | Pgam5 | 0 | 0,84 | 8,85E-01 |
| Q8VE09 | 6 | 3 | 5,73 | 72747 | Ttc39c | 0 | 1,11 | 9,78E-01 |
| Q9D3P8 | 14 | 2 | 9,47 | 67759 | Plgrkt | 0 | 0,75 | 7,93E-01 |
| Q8BFZ2 | 5 | 2 | 11,76 | 272031 | Plppr1 | 0 | 1,13 | 9,11E-01 |
| SODC_HUMAN | 20 | 2 | 11,28 | | | 0 | 0,73 | 7,69E-01 |
| P49945 | 25 | 3 | 0 | | Ftl2 | 0 | 0,74 | 7,77E-01 |
| Q9JJ59 | 4 | 2 | 7,86 | 56325 | Abcb9 | 0 | 0,53 | 3,85E-01 |
| P70349 | 17 | 2 | 12,8 | 15254 | Hint1 | 0 | 0,97 | 9,76E-01 |
| P56873 | 19 | 2 | 2,77 | 56390 | Znrd2 | 0 | 1,69 | 4,83E-01 |
| Q9EP53 | 3 | 3 | 6,15 | 64930 | Tsc1 | 0 | 1,17 | 9,41E-01 |
| P17439 | 4 | 2 | 3,59 | 14466 | Gba | 0 | 1,28 | 8,70E-01 |
| Q9DBX6 | 7 | 3 | 2,44 | 74134 | Cyp2s1 | 0 | 1,16 | 9,62E-01 |
| Q9JI90 | 5 | 2 | 11,74 | 56736 | Rnf14 | 0 | 1,04 | 1,00E+00 |
| P56395 | 19 | 2 | 4,5 | 109672 | Cyb5a | 0 | 1,11 | 9,76E-01 |
| A2APY7 | 7 | 2 | 4,15 | 69487 | Ndufaf5 | 0 | 0,78 | 7,97E-01 |
| Q9D020 | 10 | 3 | 5,92 | 107569 | Nt5c3a | 0 | 0,95 | 9,78E-01 |
| P0C7L0 | 7 | 3 | 4,12 | | Wipf3 | 0 | 1,28 | 8,10E-01 |
| D3Z7H4 | 7 | 2 | 18,08 | 269994 | Gsg1l | 0 | 1,07 | 9,85E-01 |
| P62301 | 9 | 2 | 4,15 | 68052 | Rps13 | 0 | 0,00 | 1,60E-16 |
| O09131 | 13 | 3 | 1,83 | 14873 | Gsto1 | 0 | 0,93 | 9,56E-01 |
| Q9ES56 | 15 | 3 | 2,2 | 60409 | Trappc4 | 0 | 1,29 | 7,86E-01 |
| P68037 | 17 | 3 | 5,17 | 22195 | Ube2l3 | 0 | 1,44 | 6,79E-01 |
| Q80T79 | 1 | 2 | 1,63 | 239420 | Csmd3 | 0 | 1,10 | 9,77E-01 |
| Q3U0D9 | 3 | 3 | 1,93 | 209462 | Hace1 | 0 | 1,49 | 6,59E-01 |
| Q9DBR3 | 4 | 3 | 11,18 | 74125 | Armc8 | 0 | 1,35 | 5,25E-01 |
| Q8BYC6 | 3 | 3 | 19,04 | 330177 | Taok3 | 0 | 1,06 | 9,74E-01 |
| P63216 | 29 | 2 | 11,07 | 14704 | Gng3 | 0 | 1,00 | 1,00E+00 |
| Q80YV4 | 4 | 3 | 1,68 | 269614 | Pank4 | 0 | 1,07 | 9,85E-01 |
| P16110 | 9 | 2 | 2,28 | | Lgals3 | 0 | 1,12 | 9,76E-01 |
| Q9D9V3 | 9 | 3 | 6,95 | 52665 | Echdc1 | 0 | 0,68 | 2,19E-01 |
| Q8BMK4 | 7 | 4 | 0 | 216197 | Ckap4 | 0 | 0,29 | 4,18E-03 |
| Q9WTK7 | 7 | 3 | 6,31 | 20869 | Stk11 | 0 | 1,40 | 7,33E-01 |
| P47743 | 6 | 3 | 8,07 | 14823 | Grm8 | 0 | 1,20 | 9,56E-01 |
| Q99KC8 | 4 | 3 | 9,47 | 67776 | Vwa5a | 0 | 0,76 | 4,28E-01 |

| | | | | | | | | |
|---|---|---|---|---|---|---|---|---|
| Q8CIB5 | 5 | 3 | 0 | 218952 | Fermt2 | 0 | 0,69 | 6,86E-01 |
| O54946 | 7 | 2 | 9,32 | 23950 | Dnajb6 | 0 | 1,28 | 9,11E-01 |
| P70699 | 4 | 3 | 3,38 | 14387 | Gaa | 0 | 0,70 | 6,90E-01 |
| Q64475 | 20 | 3 | 5,86 | 319178 | H2bc3 | 0 | 0,68 | 1,50E-01 |
| Q8CGP2 | 20 | 3 | 5,86 | 319188 | Hist1h2b p | 0 | 0,68 | 1,50E-01 |
| Q64525 | 20 | 3 | 5,86 | 319189 | Hist2h2b b | 0 | 0,68 | 1,50E-01 |
| Q8CGP1 | 20 | 3 | 5,86 | 319184 | H2bc12 | 0 | 0,68 | 1,50E-01 |
| P10854 | 20 | 3 | 5,86 | 319186 | H2bc14 | 0 | 0,68 | 1,50E-01 |
| Q64478 | 20 | 3 | 5,86 | 319182 | H2bc9 | 0 | 0,68 | 1,50E-01 |
| Q6ZWY9 | 20 | 3 | 5,86 | 319179; 319181; 68024 | H2bc4; H2bc6; H2bc8 | 0 | 0,68 | 1,50E-01 |
| P10853 | 20 | 3 | 5,86 | 319180; 319183; 319185; 319187; 665596; 665622 | H2bc11; H2bc13; H2bc15; H2bc7 | 0 | 0,68 | 1,50E-01 |
| Q91XD7 | 5 | 2 | 9,05 | 171508 | Creld1 | 0 | 0,82 | 8,53E-01 |
| Q9JL26 | 2 | 2 | 10,15 | 57778 | Fmnl1 | 0 | 1,23 | 9,05E-01 |
| P70188 | 4 | 3 | 5,82 | 16579 | Kifap3 | 0 | 1,29 | 8,85E-01 |
| Q6P5E4 | 2 | 3 | 3,65 | 320011 | Uggt1 | 0 | 0,58 | 4,13E-01 |
| Q91VR5 | 3 | 2 | 6,21 | 104721 | Ddx1 | 0 | 0,93 | 9,76E-01 |
| Q99KI3 | 15 | 3 | 0 | 66087 | Emc3 | 0 | 1,12 | 9,76E-01 |
| Q91YD9 | 5 | 3 | 15,77 | 73178 | Wasl | 0 | 1,07 | 9,74E-01 |
| Q7TPS5 | 2 | 2 | 1,83 | 74741 | C2cd5 | 0 | 1,17 | 9,64E-01 |
| Q9JIF7 | 5 | 3 | 0 | 70349 | Copb1 | 0 | 0,95 | 9,76E-01 |
| Q8VD62 | 8 | 2 | 9,3 | 107242 | Bles03 | 0 | 1,05 | 9,91E-01 |
| P15920 | 2 | 2 | 34,07 | 21871 | Atp6v0a2 | 0 | 0,89 | 8,42E-01 |
| P42225 | 3 | 2 | 1,85 | | Stat1 | 0 | 1,23 | 9,50E-01 |
| Q8BYN5 | 5 | 2 | 6,9 | 319636 | Fsd1l | 0 | 0,90 | 9,56E-01 |
| Q9JIM1 | 5 | 2 | 20,46 | 63959 | Slc29a1 | 0 | 1,13 | 9,65E-01 |
| Q99LB2 | 10 | 3 | 1,78 | 28200 | Dhrs4 | 0 | 1,22 | 9,56E-01 |
| Q9DCC8 | 17 | 2 | 2,41 | 67952 | Tomm20 | 0 | 1,43 | 3,20E-01 |
| P36536 | 10 | 2 | 22,72 | | Sar1a | 0 | 1,23 | 7,60E-01 |
| Q61234 | 5 | 2 | 7,95 | | Snta1 | 1 | 0,70 | 3,58E-01 |
| Q80U19 | 3 | 3 | 3,95 | 76441 | Daam2 | 0 | 0,53 | 3,19E-01 |
| Q9JMK2 | 6 | 2 | 2,07 | 27373 | Csnk1e | 0 | 1,68 | 4,08E-01 |
| Q923Z0 | 9 | 3 | 0 | 64297 | Gprc5b | 0 | 1,09 | 9,76E-01 |
| Q9CXI0 | 11 | 3 | 0 | 52064 | Coq5 | 0 | 0,57 | 3,44E-01 |
| P40630 | 11 | 3 | 3,68 | 21780 | Tfam | 0 | 0,98 | 9,94E-01 |
| E9Q8I9 | 1 | 4 | 0 | 320365 | Fry | 0 | 1,27 | 8,93E-01 |
| Q5XJY5 | 7 | 3 | 0 | 213827 | Arcn1 | 0 | 1,34 | 5,88E-01 |
| P24668 | 8 | 2 | 10,7 | 17113 | M6pr | 0 | 0,45 | 1,77E-02 |
| O70274 | 13 | 2 | 2,14 | 19244 | Ptp4a2 | 0 | 1,19 | 9,36E-01 |
| Q63739 | 13 | 2 | 2,14 | 19243 | Ptp4a1 | 0 | 1,19 | 9,36E-01 |
| Q7TSI3 | 5 | 2 | 0 | 243819 | Ppp6r1 | 0 | 1,10 | 9,76E-01 |

| | | | | | | | | |
|---|---|---|---|---|---|---|---|---|
| Q8C0J2 | 3 | 2 | 5,88 | 77040 | Atg16l1 | 0 | 1,42 | 7,74E-01 |
| Q0VGU4 | 7 | 3 | 1,92 | 381677 | Vgf | 0 | 1,10 | 9,76E-01 |
| P70280 | 11 | 3 | 12,46 | 20955 | Vamp7 | 0 | 1,82 | 1,23E-01 |
| Q8VIM9 | 4 | 2 | 11,41 | 210146 | Irgq | 0 | 1,22 | 9,19E-01 |
| Q9WVJ5 | 15 | 3 | 1,63 | 12960 | Crybb1 | 0 | 1,49 | 1,14E-01 |
| Q80TM9 | 2 | 2 | 7,09 | 64652 | Nisch | 0 | 0,99 | 9,92E-01 |
| O54734 | 7 | 3 | 4,21 | 13200 | Ddost | 0 | 1,00 | 9,96E-01 |
| P97742 | 5 | 3 | 3,41 | 12894 | Cpt1a | 0 | 0,90 | 9,56E-01 |
| Q8VDK1 | 9 | 3 | 0 | 27045 | Nit1 | 0 | 1,94 | 1,93E-01 |
| Q8BTZ7 | 9 | 3 | 5,73 | 331026 | Gmppb | 0 | 0,80 | 8,21E-01 |
| Q8K2P7 | 4 | 2 | 1,93 | 105727 | Slc38a1 | 0 | 1,72 | 3,40E-01 |
| Q8BX94 | 11 | 2 | 5 | 228983 | Osbpl2 | 0 | | |
| Q9CXV1 | 11 | 2 | 2,17 | 66925 | Sdhd | 0 | 1,09 | 9,76E-01 |
| Q9D1D4 | 11 | 2 | 0 | 68581 | Tmed10 | 0 | 1,12 | 9,76E-01 |
| Q8CJ61 | 9 | 2 | 17,04 | 97487 | Cmtm4 | 0 | 1,24 | 8,66E-01 |
| Q8BGK5 | 11 | 2 | 3,32 | 215085 | Slc35f1 | 0 | 0,76 | 8,08E-01 |
| Q9D328 | 14 | 2 | 2,08 | 67564 | Tmem35a | 0 | 1,10 | 9,74E-01 |
| P56376 | 27 | 2 | 7,97 | 66204 | Acyp1 | 0 | 1,34 | 8,07E-01 |
| Q80Y55 | 8 | 3 | 1,69 | 100383 | Bsdc1 | 0 | 1,00 | 9,97E-01 |
| Q8VCM8 | 5 | 3 | 7,28 | 103425 | Ncln | 0 | 1,07 | 9,76E-01 |
| Q8VD75 | 4 | 2 | 1,69 | 215114 | Hip1 | 0 | | |
| Q8R2Y8 | 18 | 2 | 1,6 | 217057 | Ptrh2 | 0 | 1,10 | 9,76E-01 |
| P26516 | 7 | 2 | 4,11 | 17463 | Psmd7 | 0 | 1,01 | 1,00E+00 |
| Q9JJR8 | 15 | 3 | 12,27 | 56786 | Tmem9b | 0 | 1,19 | 9,43E-01 |
| P61979 | 6 | 2 | 2,52 | 15387 | Hnrnpk | 0 | 1,01 | 9,98E-01 |
| P05555 | 3 | 3 | 0 | | Itgam | 0 | 0,49 | 1,83E-01 |
| Q8C5W3 | 8 | 3 | 1,62 | 272589 | Tbcel | 0 | 1,14 | 9,24E-01 |
| Q6GYP7 | 1 | 2 | 1,78 | 56784 | Ralgapa1 | 0 | 1,34 | 8,95E-01 |
| Q8R0Y8 | 10 | 3 | 13,35 | 73095 | Slc25a42 | 0 | 0,56 | 2,79E-02 |
| Q5RKR3 | 5 | 3 | 1,89 | 320563 | Islr2 | 0 | 0,96 | 9,78E-01 |
| Q6PD26 | 4 | 2 | 3,87 | 276846 | Pigs | 0 | 1,04 | 9,94E-01 |
| P56382 | 44 | 3 | 34,39 | 67126 | Atp5f1e | 0 | 1,04 | 9,85E-01 |
| O08914 | 5 | 3 | 3,68 | 14073 | Faah | 0 | 0,68 | 6,07E-01 |
| P61211 | 11 | 2 | 9,88 | 104303 | Arl1 | 0 | 1,11 | 9,74E-01 |
| P21300 | 9 | 2 | 22,48 | 11997 | Akr1b7 | 0 | 0,92 | 9,66E-01 |
| P62069 | 6 | 2 | 11,34 | 69727 | Usp46 | 0 | 1,26 | 8,07E-01 |
| P31230 | 9 | 2 | 3,7 | | Aimp1 | 0 | 0,32 | 2,16E-02 |
| Q920P5 | 4 | 2 | 9,26 | 229949 | Ak5 | 0 | 1,08 | 9,83E-01 |
| Q8K3W0 | 10 | 3 | 0 | 107976 | Babam2 | 0 | 1,08 | 9,87E-01 |
| Q9CZN7 | 5 | 3 | 0 | 108037 | Shmt2 | 0 | 1,33 | 7,84E-01 |
| Q9DB72 | 8 | 2 | 2,15 | 72014 | Btbd17 | 0 | 0,00 | 1,60E-16 |
| O70585 | 4 | 3 | 7,41 | 13528 | Dtnb | 0 | 1,06 | 9,76E-01 |
| O35598 | 3 | 2 | 1,87 | 11487 | Adam10 | 0 | 0,57 | 3,61E-01 |
| Q62186 | 12 | 2 | 12,77 | 20832 | Ssr4 | 0 | 1,41 | 7,10E-01 |
| P0C027 | 18 | 2 | 1,65 | 102954; 58242 | Nudt10 | 0 | 1,29 | 8,42E-01 |
| Q8C080 | 7 | 2 | 3,72 | 74718 | Snx16 | 0 | 1,04 | 9,87E-01 |

| | | | | | | | | |
|---|---|---|---|---|---|---|---|---|
| Q8QZY1 | 6 | 3 | 0 | 223691 | Eif3l | 0 | 0,99 | 9,85E-01 |
| Q9CPS6 | 15 | 2 | 23,04 | 66847 | Hint3 | 0 | 1,20 | 8,64E-01 |
| Q9JIA7 | 6 | 2 | 1,91 | 56632 | Sphk2 | 0 | 1000,00 | 1,60E-16 |
| Q9D8B7 | 9 | 3 | 7,29 | 83964 | Jam3 | 0 | 1,37 | 4,35E-01 |
| O55029 | 4 | 3 | 2,12 | 50797 | Copb2 | 0 | 1,18 | 9,63E-01 |
| Q922H2 | 5 | 2 | 19,19 | 236900 | Pdk3 | 0 | 0,94 | 9,76E-01 |
| Q6PHU5 | 3 | 2 | 5,53 | 20661 | Sort1 | 0 | 0,76 | 8,17E-01 |
| Q6V4S5 | 2 | 4 | 0 | 237979 | Sdk2 | 0 | 1,21 | 9,34E-01 |
| Q810J8 | 5 | 3 | 1,65 | 217695 | Zfyve1 | 0 | 1,08 | 9,74E-01 |
| Q6ZPF4 | 3 | 3 | 0 | 22379 | Fmnl3 | 0 | 0,97 | 9,77E-01 |
| Q5SSH7 | 1 | 3 | 0 | 195018 | Zzef1 | 0 | 1,23 | 9,09E-01 |
| Q9CWU6 | 9 | 2 | 5,48 | 56046 | Uqcc1 | 0 | 0,76 | 7,49E-01 |
| Q8K2C6 | 7 | 2 | 0 | 68346 | Sirt5 | 0 | 1,37 | 7,63E-01 |
| Q8K394 | 3 | 3 | 4,19 | 224860 | Plcl2 | 0 | 1,26 | 8,91E-01 |
| Q921G8 | 4 | 3 | 0 | 74237 | Tubgcp2 | 0 | 1,41 | 8,52E-01 |
| P35979 | 15 | 2 | 20,5 | 269261 | Rpl12 | 0 | 0,83 | 8,68E-01 |
| Q9ES89 | 7 | 2 | 1,61 | 58193 | Extl2 | 0 | 1,15 | 9,34E-01 |
| Q8K353 | 11 | 2 | 9,16 | | Cystm1 | 0 | 1,11 | 9,76E-01 |
| Q8VCR2 | 8 | 3 | 1,81 | 243168 | Hsd17b13 | 0 | 0,91 | 9,74E-01 |
| Q9DBX3 | 2 | 2 | 16,16 | 71733 | Susd2 | 0 | 0,95 | 9,76E-01 |
| Q9D7V2 | 10 | 2 | 5,42 | 70082 | Lysmd2 | 0 | 1,24 | 8,85E-01 |
| Q8BK63 | 9 | 3 | 0 | | Csnk1a1 | 0 | 1,05 | 9,87E-01 |
| Q9WUP7 | 10 | 3 | 1,63 | 56207 | Uchl5 | 0 | 0,91 | 9,71E-01 |
| P35285 | 11 | 2 | 10,46 | 19334 | Rab22a | 0 | 0,77 | 7,93E-01 |
| Q8BGY2 | 18 | 3 | 6,05 | 208691 | Eif5a2 | 0 | 0,84 | 8,82E-01 |
| Q64455 | 3 | 3 | 0 | 19271 | Ptprj | 0 | 0,72 | 7,49E-01 |
| Q64523 | 12 | 2 | 12,7 | 319176 | H2ac20 | 0 | 0,93 | 9,64E-01 |
| Q8BFU2 | 12 | 2 | 12,7 | 319162 | H2aw | 0 | 0,93 | 9,64E-01 |
| Q3THW5 | 13 | 2 | 12,7 | 77605 | H2az2 | 0 | 0,93 | 9,64E-01 |
| C0HKE3 | 12 | 2 | 12,7 | 319164; 319165; 319166; 319167; 319170; 319171; 319172; 319191; 665433 | H2ac7 | 0 | 0,93 | 9,64E-01 |
| P0C0S6 | 13 | 2 | 12,7 | 51788 | H2az1 | 0 | 0,93 | 9,64E-01 |
| Q8CGP5 | 12 | 2 | 12,7 | 319173 | Hist1h2af | 0 | 0,93 | 9,64E-01 |
| Q8R1M2 | 12 | 2 | 12,7 | 232440 | H2aj | 0 | 0,93 | 9,64E-01 |
| P27661 | 11 | 2 | 12,7 | 15270 | H2ax | 0 | 0,93 | 9,64E-01 |
| Q8CGP7 | 12 | 2 | 12,7 | 319169 | H2ac15 | 0 | 0,93 | 9,64E-01 |
| Q8CGP6 | 13 | 2 | 12,7 | 319168 | H2ac12 | 0 | 0,93 | 9,64E-01 |
| Q6GSS7 | 12 | 2 | 12,7 | 15267; 319192 | Hist2h2aa1; Hist2h2aa2 | 0 | 0,93 | 9,64E-01 |
| Q9QYB1 | 8 | 2 | 18,21 | 29876 | Clic4 | 0 | 1,07 | 9,83E-01 |
| Q7TMC8 | 2 | 2 | 5,36 | 234730 | Fcsk | 0 | 1000,00 | 1,60E-16 |

| | | | | | | | | |
|---|---|---|---|---|---|---|---|---|
| Q91ZH7 | 6 | 2 | 5,61 | 106861 | Abhd3 | 0 | | |
| Q99MK8 | 3 | 2 | 3,54 | 110355 | Grk2 | 0 | 0,94 | 9,74E-01 |
| Q3UYH7 | 3 | 2 | 3,54 | 320129 | Adrbk2 | 0 | 0,94 | 9,74E-01 |
| P47963 | 9 | 2 | 0 | 270106 | Rpl13 | 0 | 1,05 | 9,94E-01 |
| P59764 | 1 | 2 | 1,66 | 238130 | Dock4 | 0 | 0,68 | 7,09E-01 |
| Q8VDZ4 | 4 | 2 | 1,84 | 228136 | Zdhhc5 | 0 | 1000,00 | 1,60E-16 |
| Q9DB15 | 13 | 2 | 4,32 | 56282 | Mrpl12 | 0 | 2,74 | 6,20E-05 |
| P63213 | 41 | 2 | 8,52 | 14702 | Gng2 | 0 | 0,81 | 8,85E-01 |
| Q78JN3 | 8 | 2 | 10,77 | 69123 | Eci3 | 0 | 0,88 | 9,56E-01 |
| P21661 | 4 | 2 | 0 | 18549 | Pcsk2 | 0 | 0,99 | 9,96E-01 |
| Q9CQ65 | 10 | 2 | 3,93 | 66902 | Mtap | 0 | 1,40 | 7,86E-01 |
| Q920R6 | 2 | 2 | 32,38 | 140494 | Atp6v0a4 | 0 | 0,85 | 7,61E-01 |
| Q8R0F8 | 13 | 2 | 0 | 68636 | Fahd1 | 0 | 1,25 | 9,16E-01 |
| Q60770 | 6 | 3 | 0 | 20912 | Stxbp3 | 0 | 0,85 | 9,34E-01 |
| Q8BGB7 | 8 | 2 | 18,94 | 67870 | Enoph1 | 0 | 1,07 | 9,76E-01 |
| Q9CQE8 | 10 | 2 | 1,75 | 68045 | RTRAF | 0 | 1,74 | 4,49E-01 |
| P70333 | 7 | 2 | 0 | 56258 | Hnrnph2 | 0 | 0,74 | 7,89E-01 |
| O35737 | 7 | 2 | 0 | 59013 | Hnrnph1 | 0 | 0,74 | 7,89E-01 |
| Q8BHG2 | 18 | 2 | 4,2 | 74098 | Czib | 0 | 0,83 | 8,24E-01 |
| Q80Y98 | 4 | 3 | 1,75 | 72108 | Ddhd2 | 0 | 1,11 | 9,76E-01 |
| Q8BMD8 | 5 | 2 | 9,76 | 229731 | Slc25a24 | 0 | 1,16 | 9,66E-01 |
| P0C5J4 | 5 | 2 | 0 | 620246 | Gpr52 | 0 | 1,75 | 2,49E-01 |
| P70211 | 2 | 3 | 1,73 | 13176 | Dcc | 0 | 1,53 | 6,94E-01 |
| Q9CZX7 | 14 | 2 | 1,7 | 72519 | Pip4p2 | 0 | 0,65 | 5,79E-01 |
| Q9D1E8 | 6 | 2 | 13,67 | 52123 | Agpat5 | 0 | 0,78 | 7,74E-01 |
| Q8BH86 | 6 | 3 | 0 | 217830 | Dglucy | 0 | 1,07 | 9,76E-01 |
| O88597 | 4 | 2 | 5,72 | 56208 | Becn1 | 0 | 0,60 | 2,31E-01 |
| P70663 | 3 | 2 | 0 | 13602 | Sparcl1 | 0 | 0,79 | 8,52E-01 |
| Q3TFQ1 | 11 | 2 | 12,86 | 66674 | Spryd7 | 0 | 1,34 | 8,01E-01 |
| P63056 | 4 | 2 | 10,04 | 229759 | Olfm3 | 0 | 1,05 | 9,97E-01 |
| P00848 | 8 | 2 | 9,97 | 17705 | Mtatp6 | 0 | 0,78 | 7,50E-01 |
| Q9QUH0 | 31 | 3 | 0 | 93692 | Glrx | 0 | 1,09 | 9,76E-01 |
| Q7TN79 | 9 | 3 | 0 | 432442 | Akap7 | 0 | 1,34 | 7,61E-01 |
| O55074 | 36 | 3 | 0 | 432442 | Akap7 | 0 | 1,34 | 7,61E-01 |
| Q9JKW0 | 9 | 2 | 9,41 | 54208 | Arl6ip1 | 0 | 0,96 | 9,74E-01 |
| Q8BGP6 | 11 | 3 | 1,71 | 319653 | Slc25a40 | 0 | 1,05 | 9,96E-01 |
| Q8BP00 | 4 | 2 | 0 | 320299 | Iqcb1 | 0 | 1,56 | 6,72E-01 |
| Q9JLQ2 | 2 | 2 | 5,61 | 26431 | Git2 | 0 | 1,11 | 9,56E-01 |
| Q8CCN5 | 3 | 2 | 0 | 192197 | Bcas3 | 0 | 0,47 | 1,70E-02 |
| Q8K1A6 | 3 | 2 | 3,27 | 212139 | Cc2d1a | 0 | 0,92 | 9,27E-01 |
| Q9Z1Q9 | 2 | 2 | 1,65 | 22321 | Vars1 | 0 | 0,85 | 9,56E-01 |
| Q6ZPF3 | 2 | 2 | 1,82 | 24001 | Tiam2 | 0 | 1,26 | 9,37E-01 |
| Q8K1S4 | 3 | 2 | 3,51 | 107448 | Unc5a | 0 | 0,83 | 9,35E-01 |
| Q9D2N9 | 4 | 3 | 0 | 77573 | Vps33a | 0 | 1,32 | 7,37E-01 |
| Q8K010 | 3 | 3 | 0 | 75475 | Oplah | 0 | 0,70 | 6,98E-01 |
| Q9Z1K5 | 5 | 2 | 6,01 | 23806 | Arih1 | 0 | | |
| P70290 | 6 | 2 | 2,17 | 17524 | Mpp1 | 0 | 0,61 | 5,27E-01 |

| | | | | | | | | |
|---|---|---|---|---|---|---|---|---|
| P08030 | 13 | 2 | 0 | 11821 | Aprt | 0 | 1,07 | 9,76E-01 |
| P27659 | 7 | 3 | 0 | 27367 | Rpl3 | 0 | 0,88 | 9,56E-01 |
| P61804 | 19 | 2 | 0 | 13135 | Dad1 | 0 | 1,00 | 9,99E-01 |
| Q9D1M0 | 11 | 2 | 8,74 | 110379 | Sec13 | 0 | 1,13 | 9,74E-01 |
| Q91XD6 | 6 | 2 | 2,05 | 70160 | Vps36 | 0 | 1,21 | 9,34E-01 |
| Q99LS3 | 9 | 2 | 0 | 100678 | Psph | 0 | 1,12 | 9,76E-01 |
| P97412 | 1 | 3 | 0 | 17101 | Lyst | 0 | 1,74 | 5,10E-01 |
| O08739 | 4 | 3 | 0 | 11717 | Ampd3 | 0 | 0,95 | 9,77E-01 |
| P18581 | 3 | 2 | 11,03 | 11988 | Slc7a2 | 0 | 0,89 | 9,56E-01 |
| Q91VA6 | 7 | 2 | 2,16 | 67811 | Poldip2 | 0 | 0,84 | 9,16E-01 |
| Q80UP3 | 4 | 3 | 0 | 104418 | Dgkz | 0 | 0,94 | 9,76E-01 |
| Q8BNJ6 | 4 | 2 | 0 | 74513 | Neto2 | 0 | 0,81 | 9,02E-01 |
| O54818 | 11 | 2 | 1,85 | 21987 | Tpd52l1 | 0 | 29,12 | 1,60E-16 |
| Q9DBH5 | 6 | 2 | 3,45 | 66890 | Lman2 | 0 | 1,30 | 8,91E-01 |
| P56528 | 8 | 2 | 3,89 | 12494 | Cd38 | 0 | 1,55 | 6,58E-01 |
| P07146 | 15 | 2 | 1,82 | 22072 | Prss2 | 0 | 1000,00 | 1,60E-16 |
| P62075 | 26 | 2 | 1,67 | 30055 | Timm13 | 0 | 1,08 | 9,71E-01 |
| P09055 | 2 | 2 | 9,86 | 16412 | Itgb1 | 0 | 0,68 | 5,68E-01 |
| Q9JJ28 | 2 | 2 | 1,99 | 14248 | Flii | 0 | 1,08 | 9,78E-01 |
| Q9CPT4 | 14 | 2 | 0 | 28106 | Mydgf | 0 | 0,80 | 8,10E-01 |
| Q05915 | 11 | 3 | 1,8 | 14528 | Gch1 | 0 | 2,48 | 1,77E-02 |
| Q9QYJ3 | 6 | 2 | 3,7 | 81489 | Dnajb1 | 0 | 0,81 | 8,81E-01 |
| Q78JW9 | 6 | 2 | 3,67 | 28018 | Ubfd1 | 0 | 1,07 | 9,81E-01 |
| P97930 | 9 | 2 | 1,93 | 21915 | Dtymk | 0 | 0,46 | 1,19E-01 |
| Q9Z2B2 | 6 | 2 | 5,47 | 20523 | Slc25a14 | 0 | 0,89 | 9,60E-01 |
| Q3TWI9 | 2 | 2 | 1,7 | 224807 | Tmem63b | 0 | 0,00 | 1,60E-16 |
| Q5F2E8 | 2 | 2 | 13,13 | 216965 | Taok1 | 0 | 1,18 | 9,54E-01 |
| Q08642 | 4 | 2 | 3,87 | 18600 | Padi2 | 0 | 0,62 | 5,46E-01 |
| Q8BWT5 | 1 | 2 | 9,89 | 64451 | Dip2a | 0 | 1,13 | 9,34E-01 |
| P36916 | 3 | 2 | 0 | 14670 | Gnl1 | 0 | 0,91 | 9,56E-01 |
| Q8CA71 | 9 | 2 | 2,01 | 77552 | Shisa4 | 0 | 1,26 | 8,82E-01 |
| Q9WUA6 | 5 | 2 | 0 | 23797 | Akt3 | 0 | 1,22 | 9,03E-01 |
| Q8BLV3 | 2 | 2 | 0 | 236727 | Slc9a7 | 0 | 1,14 | 9,72E-01 |
| Q8BGF9 | 10 | 3 | 0 | 229517 | Slc25a44 | 0 | 0,60 | 3,26E-02 |
| Q8C3W1 | 9 | 2 | 0 | | | 0 | 1,12 | 9,70E-01 |
| Q91YX5 | 6 | 2 | 9,4 | 226856 | Lpgat1 | 0 | 1,08 | 9,76E-01 |
| Q64520 | 14 | 2 | 3,44 | 14923 | Guk1 | 0 | 0,76 | 8,37E-01 |
| Q8VDU0 | 3 | 2 | 1,66 | 76123 | Gpsm2 | 0 | 1,34 | 8,07E-01 |
| Q9D2R6 | 19 | 2 | 0 | 52469 | Coa3 | 0 | 0,77 | 7,39E-01 |
| Q3TYX3 | 5 | 2 | 0 | 232187 | Smyd5 | 0 | 1,19 | 9,54E-01 |
| P46467 | 5 | 2 | 1,74 | 20479 | Vps4b | 0 | 1,06 | 9,85E-01 |
| Q9ERK7 | 4 | 2 | 4,3 | 11444 | Chrnb2 | 0 | 2,98 | 4,58E-02 |
| Q99J39 | 4 | 2 | 1,83 | 56690 | Mlycd | 0 | 0,95 | 9,74E-01 |
| Q9CZM2 | 11 | 3 | 0 | 66480 | Rpl15 | 0 | 1,10 | 9,79E-01 |
| P60229 | 5 | 2 | 0 | 16341 | Eif3e | 0 | 0,03 | 1,60E-16 |
| Q9WTN0 | 6 | 2 | 1,78 | 14593 | Ggps1 | 0 | 0,97 | 9,78E-01 |
| P83887 | 5 | 2 | 10,92 | 103733 | Tubg1 | 0 | 1,43 | 7,12E-01 |

| | | | | | | | | |
|---|---|---|---|---|---|---|---|---|
| Q8VCK3 | 5 | 2 | 10,92 | 103768 | Tubg2 | 0 | 1,43 | 7,12E-01 |
| Q80W54 | 7 | 3 | 0 | 230709 | Zmpste24 | 0 | 0,54 | 1,66E-01 |
| Q9CWX2 | 8 | 2 | 1,77 | 69702 | Ndufaf1 | 0 | 0,71 | 7,10E-01 |
| Q8R1Y2 | 10 | 2 | 3,23 | 67254 | Bmerb1 | 0 | 1,85 | 4,72E-02 |
| Q6PHZ8 | 8 | 2 | 2,22 | 80334 | Kcnip4 | 0 | 1,49 | 6,43E-01 |
| Q8K0T4 | 4 | 2 | 2,11 | 231912 | Katnal1 | 0 | 0,98 | 9,87E-01 |
| Q9JKL4 | 18 | 2 | 1,7 | 66706 | Ndufaf3 | 0 | 0,79 | 8,59E-01 |
| Q62077 | 2 | 2 | 1,72 | 18803 | Plcg1 | 0 | 0,61 | 1,22E-01 |
| Q3V3Q7 | 2 | 2 | 6,33 | 217893 | Pacs2 | 0 | 0,65 | 3,85E-01 |
| Q5F285 | 33 | 2 | 5,88 | 69186 | Tmem256 | 0 | 1,02 | 1,00E+00 |
| O55028 | 6 | 2 | 0 | 12041 | Bckdk | 0 | 0,87 | 9,17E-01 |
| Q3UI43 | 6 | 2 | 4,93 | 68251 | Babam1 | 0 | 1,23 | 8,95E-01 |
| Q3UJP5 | 13 | 2 | 0 | | | 0 | 0,88 | 9,71E-01 |
| Q64373 | 8 | 2 | 5,47 | 12048 | Bcl2l1 | 0 | 1,17 | 9,48E-01 |
| Q99P31 | 6 | 2 | 0 | 66245 | Hspbp1 | 0 | 1,12 | 9,72E-01 |
| Q8VE22 | 11 | 2 | 1,8 | 64656 | Mrps23 | 0 | 1000,00 | 1,60E-16 |
| P00015 | 14 | 3 | 3,6 | 13067 | Cyct | 0 | 0,93 | 9,40E-01 |
| Q8BP40 | 6 | 2 | 2,03 | 66659 | Acp6 | 0 | 0,82 | 8,40E-01 |
| Q920A7 | 2 | 2 | 3,85 | 114896 | Afg3l1 | 0 | 0,00 | 1,60E-16 |
| Q9CY50 | 7 | 2 | 3,38 | 107513 | Ssr1 | 0 | 0,76 | 6,06E-01 |
| Q3U1F9 | 7 | 2 | 0 | 94212 | Pag1 | 0 | 0,31 | 4,18E-03 |
| P26450 | 3 | 2 | 5,49 | 18708 | Pik3r1 | 0 | 0,76 | 7,50E-01 |
| Q8BJH1 | 10 | 2 | 0 | 67306 | Zc2hc1a | 0 | 2,43 | 5,80E-02 |
| Q6TEK5 | 12 | 2 | 3,8 | 69568 | Vkorc1l1 | 0 | 1,23 | 9,48E-01 |
| Q9CR09 | 11 | 2 | 8,01 | 66155 | Ufc1 | 0 | 1,04 | 9,94E-01 |
| Q9QZD9 | 6 | 2 | 1,65 | 54709 | Eif3i | 0 | 1,01 | 1,00E+00 |
| Q60766 | 5 | 2 | 1,72 | 15944 | Irgm1 | 0 | 1,08 | 9,85E-01 |
| P09925 | 6 | 2 | 2,36 | 20930 | Surf1 | 0 | 0,74 | 7,86E-01 |
| P70696 | 13 | 2 | 5,86 | 319177 | H2bc1 | 0 | 0,68 | 1,66E-01 |
| Q9WTL4 | 1 | 2 | 59,67 | 23920 | Insrr | 0 | 0,99 | 9,91E-01 |
| Q8C052 | 3 | 2 | 11,86 | 270058 | Map1s | 0 | 1,25 | 9,11E-01 |
| O35417 | 6 | 2 | 8,99 | | Pdyn | 0 | 1,21 | 9,25E-01 |
| Q9WTQ8 | 13 | 2 | 3,8 | | Timm23 | 0 | 1,02 | 9,91E-01 |
| O88851 | 15 | 2 | 0 | 26450 | Rbbp9 | 0 | 1000,00 | 1,60E-16 |
| Q8BHL8 | 8 | 2 | 0 | 228769 | Psmf1 | 0 | 1,02 | 1,00E+00 |
| Q8BH70 | 3 | 2 | 3,39 | 269514 | Fbxl4 | 0 | 1,22 | 8,91E-01 |
| Q8CGV2 | 5 | 2 | 0 | 216343 | Tph2 | 0 | 0,62 | 5,45E-01 |
| Q6ZWN5 | 9 | 2 | 4,67 | 76846 | Rps9 | 0 | 0,59 | 4,80E-01 |
| Q80VP9 | 7 | 2 | 0 | 72898 | Asphd2 | 0 | 0,01 | 1,60E-16 |
| O55242 | 9 | 2 | 2,01 | 18391 | Sigmar1 | 0 | 0,04 | 1,60E-16 |
| P51175 | 5 | 2 | 0 | 19044 | Ppox | 0 | 0,92 | 9,74E-01 |
| Q9R1J0 | 7 | 2 | 1,71 | 18194 | Nsdhl | 0 | 1,04 | 9,96E-01 |
| P58044 | 7 | 2 | 1,65 | 102635781; 319554 | Idi1 | 0 | 1,32 | 8,12E-01 |
| Q8BH00 | 2 | 2 | 9,05 | 237320 | Aldh8a1 | 0 | 0,00 | 1,60E-16 |
| S4R1M9 | 3 | 2 | 1,67 | 74486 | Osbpl10 | 0 | 1,20 | 9,44E-01 |

| | | | | | | | | |
|---|---|---|---|---|---|---|---|---|
| P70335 | 1 | 2 | 4,21 | 19877 | Rock1 | 0 | 0,77 | 8,42E-01 |
| Q3UHD2 | 5 | 2 | 0 | 328232 | Gfod1 | 0 | 1,01 | 1,00E+00 |
| O88569 | 7 | 2 | 1,62 | 53379 | Hnrnpa2b1 | 0 | 0,63 | 5,69E-01 |
| Q9D0E3 | 13 | 2 | 1,7 | 217779 | Lysmd1 | 0 | 1,01 | 1,00E+00 |
| Q5DU31 | 7 | 2 | 0 | 320495 | Ipcef1 | 0 | 1,33 | 8,45E-01 |
| Q08274 | 3 | 2 | 6,73 | 13401 | Dmwd | 0 | 1,65 | 5,13E-01 |
| Q61733 | 4 | 2 | 0 | 57312 | Mrps31 | 0 | 0,64 | 4,24E-01 |
| Q80V42 | 5 | 2 | 3,66 | 70574 | Cpm | 0 | 0,59 | 4,83E-01 |
| Q08460 | 2 | 2 | 5,34 | 16531 | Kcnma1 | 0 | 0,80 | 8,40E-01 |
| Q3TMH2 | 6 | 2 | 0 | 74616 | Scrn3 | 0 | | |
| Q61361 | 2 | 2 | 0 | 12032 | Bcan | 0 | 1,32 | 8,17E-01 |
| A2RSJ4 | 2 | 2 | 1,75 | 75089 | Uhrf1bp1l | 0 | 1,19 | 9,34E-01 |
| Q6PDI6 | 5 | 2 | 0 | 235461 | Mindy2 | 0 | 2,09 | 3,39E-01 |
| Q8C8T8 | 9 | 2 | 1,6 | 69499 | Tsr2 | 0 | 1,98 | 1,71E-01 |
| P70124 | 5 | 2 | 1,64 | 20724 | Serpinb5 | 0 | 0,33 | 3,07E-03 |
| Q64444 | 7 | 2 | 0 | 12351 | Ca4 | 0 | 4,76 | 4,83E-06 |
| Q4JIM5 | 2 | 2 | 3,66 | | Abl2 | 0 | 1,05 | 9,85E-01 |
| Q69ZS8 | 3 | 2 | 0 | 71529 | Kazn | 0 | | |
| Q9R0M6 | 12 | 2 | 3,29 | 56382 | Rab9a | 0 | 0,96 | 9,76E-01 |
| P61329 | 7 | 2 | 17,09 | 14167 | Fgf12 | 0 | 0,95 | 9,76E-01 |
| P70379 | 6 | 2 | 17,09 | 14169 | Fgf14 | 0 | 0,95 | 9,76E-01 |
| Q91WB7 | 9 | 2 | 0 | 226122 | Ubtd1 | 0 | 0,57 | 4,76E-01 |
| Q6PGH0 | 9 | 2 | 0 | 327900 | Ubtd2 | 0 | 0,57 | 4,76E-01 |
| P54818 | 4 | 2 | 2,11 | 14420 | Galc | 0 | 0,34 | 2,46E-02 |
| Q8VI51 | 1 | 2 | 5,83 | 66673 | Sorcs3 | 0 | 3,04 | 2,03E-03 |
| P54726 | 5 | 2 | 7,29 | 19358 | Rad23a | 0 | 0,98 | 9,87E-01 |
| Q9CQ91 | 21 | 2 | 0 | 66091 | Ndufa3 | 0 | 0,81 | 8,91E-01 |
| Q63829 | 10 | 2 | 1,82 | 12238 | Commd3 | 0 | 1,88 | 3,18E-01 |
| Q8CF89 | 5 | 2 | 0 | 66513 | Tab1 | 0 | 0,55 | 4,46E-01 |
| Q8R1X6 | 5 | 2 | 0 | 229285 | Spart | 0 | 1,01 | 9,99E-01 |
| P46737 | 9 | 2 | 14,79 | 210766 | Brcc3 | 0 | 0,96 | 9,76E-01 |
| Q3U186 | 3 | 2 | 1,68 | 109093 | Rars2 | 0 | 0,37 | 4,69E-03 |
| Q8BGF6 | 6 | 2 | 0 | 244548 | Elmod2 | 0 | 0,65 | 4,76E-01 |
| Q921W0 | 9 | 2 | 1,74 | 234852 | Chmp1a | 0 | 1,04 | 9,96E-01 |
| Q8BQM8 | 1 | 2 | 1,65 | 319670 | Eml5 | 0 | 0,88 | 9,56E-01 |
| P97429 | 6 | 2 | 0 | 11746 | Anxa4 | 0 | 0,00 | 1,60E-16 |
| Q9D061 | 7 | 2 | 0 | 72482 | Acbd6 | 0 | 1,26 | 9,12E-01 |
| P70452 | 7 | 2 | 0 | 20909 | Stx4 | 0 | 3,36 | 9,45E-04 |
| O35393 | 6 | 2 | 0 | 13643 | Efnb3 | 0 | 0,45 | 1,12E-02 |
| O89116 | 8 | 2 | 5,13 | 53611 | Vti1a | 0 | 0,89 | 9,34E-01 |
| Q6P3D0 | 9 | 2 | 0 | 75686 | Nudt16 | 0 | 1,46 | 8,08E-01 |
| Q922H4 | 6 | 2 | 2,04 | 69080 | Gmppa | 0 | 1,26 | 8,65E-01 |
| Q9D6E4 | 12 | 2 | 0 | 73032 | Ttc9b | 0 | 1,10 | 9,76E-01 |
| Q91W43 | 2 | 2 | 0 | 104174 | Gldc | 0 | 0,00 | 1,60E-16 |
| Q8K1J6 | 7 | 2 | 0 | 70047 | Trnt1 | 0 | | |
| O35459 | 6 | 2 | 1,73 | 51798 | Ech1 | 0 | 0,51 | 8,31E-02 |

| | | | | | | | | |
|---|---|---|---|---|---|---|---|---|
| P59325 | 4 | 2 | 0 | 217869 | Eif5 | 0 | 1,84 | 2,31E-01 |
| Q3USB7 | 4 | 2 | 1,69 | 227120 | Plcl1 | 0 | 0,00 | 1,60E-16 |
| Q99PW8 | 3 | 2 | 0 | 16559 | Kif17 | 0 | 0,91 | 9,74E-01 |
| P25444 | 6 | 2 | 0 | 16898 | Rps2 | 0 | 0,30 | 8,66E-05 |
| O89001 | 1 | 2 | 0 | 12874 | Cpd | 0 | 0,00 | 1,60E-16 |
| Q9WUT3 | 3 | 2 | 0 | 20112 | Rps6ka2 | 0 | 1,96 | 2,47E-01 |
| P18653 | 3 | 2 | 0 | 20111 | Rps6ka1 | 0 | 1,96 | 2,47E-01 |
| P18654 | 3 | 2 | 0 | 110651 | Rps6ka3 | 0 | 1,96 | 2,47E-01 |
| Q9CQ80 | 10 | 2 | 0 | 28084 | Vps25 | 0 | 0,90 | 9,67E-01 |
| Q91VH6 | 5 | 2 | 1,69 | 76890 | Memo1 | 0 | 1,97 | 2,41E-01 |
| Q6PAM0 | 7 | 2 | 0 | 108097 | Prkab2 | 0 | 0,87 | 9,55E-01 |
| P15388 | 4 | 2 | 1,64 | 16502 | Kcnc1 | 0 | 0,92 | 9,76E-01 |
| Q9D273 | 8 | 2 | 0 | 77697 | Mmab | 0 | 25,88 | 1,60E-16 |
| Q8BR90 | 6 | 2 | 10,68 | | | 0 | 0,99 | 9,91E-01 |
| Q8BR86 | 3 | 2 | 1,62 | 67703 | Kirrel3 | 0 | 1,25 | 8,82E-01 |
| Q3UQ84 | 3 | 2 | 0 | 71807 | Tars2 | 0 | 1,23 | 8,95E-01 |
| Q68FE2 | 4 | 2 | 0 | 245860 | Atg9a | 0 | 0,34 | 1,32E-02 |
| Q8HW98 | 8 | 2 | 0 | 210094 | Iglon5 | 0 | 0,98 | 9,87E-01 |
| Q9CR98 | 12 | 2 | 7,66 | 66488 | Fam136a | 0 | 0,73 | 6,93E-01 |
| Q9Z1Q5 | 11 | 2 | 0 | 114584 | Clic1 | 0 | 0,00 | 1,60E-16 |
| Q9D0J4 | 11 | 2 | 0 | 56327 | Arl2 | 0 | 1,44 | 8,31E-01 |
| Q3UUF8 | 5 | 2 | 0 | 218440 | Ankrd34b | 0 | 4,25 | 6,22E-06 |
| Q8C4Q6 | 9 | 2 | 0 | 108909 | Aida | 0 | 1,18 | 9,60E-01 |
| P59759 | 3 | 2 | 0 | 239719 | Mrtfb | 0 | 52,33 | 1,60E-16 |
| Q9DC63 | 6 | 2 | 0 | 57443 | Fbxo3 | 0 | 1,64 | 5,92E-01 |
| SRPP_HEVBR | 5 | 2 | 0 | | SRPP | 0 | 0,99 | 9,96E-01 |
| P50543 | 10 | 2 | 3,59 | 20195 | S100a11 | 0 | 2,18 | 8,48E-02 |
| Q80Y24 | 3 | 2 | 0 | 243548 | Prickle2 | 0 | 0,99 | 9,91E-01 |
| Q99JW1 | 5 | 2 | 3,27 | 216169 | Abhd17a | 0 | 0,84 | 7,19E-01 |
| Q6P5E8 | 2 | 2 | 0 | 110524 | Dgkq | 0 | 1000,00 | 1,60E-16 |
| Q80XU8 | 4 | 2 | 1,72 | 225875 | Lrfn4 | 0 | 0,80 | 7,93E-01 |
| O35454 | 2 | 2 | 1,67 | 26372 | Clcn6 | 0 | 0,82 | 8,52E-01 |
| Q9D967 | 18 | 2 | 3,23 | 67881 | Mdp1 | 0 | 1,01 | 1,00E+00 |
| Q8K0V2 | 6 | 2 | 0 | 233805 | Dcun1d3 | 0 | 0,94 | 9,74E-01 |
| P84244 | 10 | 2 | 3,39 | 15078; 15081 | H3-3a; H3-3b | 0 | 1,16 | 9,48E-01 |
| P02301 | 10 | 2 | 3,39 | | H3-5 | 0 | 1,16 | 9,48E-01 |
| P68433 | 10 | 2 | 3,39 | 319152; 319153; 360198; 97908 | H3c1; H3c10; H3c11; H3c8 | 0 | 1,16 | 9,48E-01 |
| P84228 | 10 | 2 | 3,39 | 15077; 260423; 319148; 319149; 319150; 319151; 319154; 97114 | H3c13; H3c14; H3c15; H3c2; H3c3; H3c4; H3c6; H3c7 | 0 | 1,16 | 9,48E-01 |

| | | | | | | | | |
|---|---|---|---|---|---|---|---|---|
| Q9ER35 | 7 | 2 | 0 | 63828 | Fn3k | 0 | 0,91 | 9,74E-01 |
| Q9CYN2 | 8 | 2 | 3,59 | 66624 | Spcs2 | 0 | 1,02 | 1,00E+00 |
| Q9JI75 | 10 | 2 | 0 | 18105 | Nqo2 | 0 | 0,80 | 5,62E-01 |
| Q9R0A0 | 5 | 2 | 5,18 | 56273 | Pex14 | 0 | 0,91 | 9,56E-01 |
| Q5IRJ6 | 3 | 2 | 0 | 109108 | Slc30a9 | 0 | 0,62 | 3,63E-01 |
| Q8K2L8 | 3 | 2 | 0 | 217449 | Trappc12 | 0 | 0,95 | 9,76E-01 |
| O88512 | 2 | 2 | 0 | 11766 | Ap1g2 | 0 | 0,82 | 8,25E-01 |
| P14115 | 13 | 2 | 0 | 26451 | Rpl27a | 0 | 0,74 | 8,01E-01 |
| Q8CI51 | 3 | 2 | 0 | 56376 | Pdlim5 | 0 | 0,80 | 5,34E-01 |
| Q8K4R4 | 5 | 2 | 0 | 71795 | Pitpnc1 | 0 | 0,75 | 7,58E-01 |
| P14576 | 5 | 2 | 0 | 24067; 665155 | Srp54 | 0 | 1,21 | 9,52E-01 |
| Q80ZJ6 | 3 | 2 | 0 | 227693 | Zer1 | 0 | 1,05 | 9,94E-01 |
| Q9QXK3 | 2 | 2 | 0 | 54160 | Copg2 | 0 | 0,91 | 9,41E-01 |
| Q80YQ8 | 7 | 2 | 0 | 68477 | Rmnd5a | 0 | 1,11 | 9,76E-01 |
| Q80X72 | 3 | 2 | 0 | 74488 | Lrrc15 | 0 | 1000,00 | 1,60E-16 |
| Q91WL8 | 4 | 2 | 0 | 80707 | Wwox | 0 | 0,74 | 3,52E-01 |
| Q9WV85 | 13 | 2 | 0 | 79059 | Nme3 | 0 | 1,11 | 9,76E-01 |
| Q02819 | 3 | 2 | 1,65 | 18220 | Nucb1 | 0 | 0,81 | 8,95E-01 |
| Q8R4H2 | 1 | 2 | 0 | | Arhgef12 | 0 | 1,27 | 9,32E-01 |
| Q8C7K6 | 3 | 2 | 0 | 240334 | Pcyox1l | 0 | 0,76 | 7,46E-01 |
| Q61271 | 5 | 2 | 0 | 11479 | Acvr1b | 0 | 0,89 | 9,64E-01 |
| Q8C547 | 1 | 2 | 1,61 | 320473 | Heatr5b | 0 | 1000,00 | 1,60E-16 |

**Supplementary Table 4: Proteomics comparison with Sharma et al.**

| All proteome | | |
|---|---|---|
| Common | Mouse Proteome Residual | DA-FASS Proteome Residual |
| Ruvbl2 | Sergef | H2bc3 |
| Gstm2 | Dhx9 | Selenoi |
| Psma4 | Ssu72 | Septin8 |
| Capzb | Eif2b2 | Atp5f1b |
| Ppia | Dcun1d2 | Plppr4 |
| Syt2 | Hebp2 | Srprb |
| Actn4 | Vps28 | Eloc |
| Tigar | Polr3c | Fcsk |
| Pithd1 | Selh | Maip1 |
| Pmm1 | Pex19 | Acacb |
| Dnajc11 | Pkia | Cmtm4 |
| Rab9b | Bcl7a | Ca4 |
| Prkag2 | Bnip3l | Vps50 |
| Nos1 | Shc2 | Afdn |
| Sod2 | Cachd1 | Atp5md |
| Camk4 | Trmt5 | Adprs |
| Tnpo1 | Ptgr1 | Acaa1b |
| Atp6v1e1 | B9d2 | Mtatp6 |
| Spata2l | Phax | Nudt10 |
| Asap1 | Spock2 | Gstp2 |
| Eef1g | Atp13a1 | Tgm3 |
| Vamp2 | Rplp2 | Rps2 |
| Pfkl | Hmgn3 | Gsdma2 |
| Ndufs4 | Strbp | Oga |
| Plp1 | Psmg2 | Nipsnap2 |
| Tst | 11-Sep | Prune1 |
| Nln | Med4 | Septin2 |
| Pi4k2a | Mief1 | Atp5f1e |
| Aimp1 | Gtf2e2 | Septin4 |
| Gnaq | Steap3 | Aloxe3 |
| Gbe1 | Tango2 | Ap1g2 |
| Aida | Kcnh1 | Kiaa0513 |
| Ctbp2 | Slc25a15 | H2ac20 |
| Fahd1 | Rnf115 | Pc |
| Ap1s1 | Hdgf | Mt-Cyb |
| Ptprs | Arhgap35 | Mtco2 |
| Gprc5b | Lzts1 | Czib |
| Lta4h | C1qtnf4 | Bles03 |
| C1qbp | Fam73b | Selenbp2 |
| Rasgrp2 | Pitpnm3 | Gatd1 |

| | | |
|---|---|---|
| Agpat3 | Sowahc | Adrm1 |
| Eef1d | Nup188 | Slc35f1 |
| Ppp2ca | Grk5 | Eprs1 |
| Ocrl | Def8 | Mrtfb |
| Ckb | Bbx | Tmem35a |
| Ndufb3 | Pea15;Pea15a | Ptpa |
| Dab2ip | Mrps9 | Hspa1b |
| Tomm40 | Samd10 | Uba1y |
| Ube2m | Nmt1 | Adgrl1 |
| Arfgef3 | Arhgap29 | H4c1; H4c11; H4c12; H4c14; H4c2; H4c3; H4c4; H4c6; H4c8; H4c9; H4f16; Hist1h4m |
| Bcat1 | Mp68 | Pcdhga4 |
| Tecpr1 | Slc47a1 | H2ac15 |
| Mboat7 | Nup210 | Ckmt2 |
| Ppm1h | Sharpin | Septin9 |
| Ran | Nol4l | Hsd17b8 |
| Tars2 | Sh3rf1 | Plppr1 |
| Grik3 | Mad1l1 | Ret |
| Stxbp5 | Tcaim;TCAIM | H2az1 |
| Crtac1 | Pla2g4e | Hnrnpk |
| Gcdh | Sorl1 | Atp6v0a4 |
| Calr | Pcbd2 | Hba |
| Ddah2 | Emsy | Atp2a1 |
| Hbb-b1 | Rpl37 | Atp5f1c |
| Wasf3 | Armcx2 | H3-5 |
| Snap29 | Dda1 | Pof1b |
| Csnk2b | Mdc1 | Psma8 |
| Suclg1 | Ptprk | Pycr3 |
| Map2 | Mzt2 | Ajm1 |
| Xkr4 | Kit | Znrd2 |
| Actc1 | Sult1a1 | Prps1 |
| Fam171a2 | Apex1 | Cyria |
| Kctd16 | Flywch1 | Adgrb1 |
| Htt | Taf5l | Hsd17b13 |
| Got1 | Ppp1r1c | Septin11 |
| Grin2b | Nop9 | Hars1 |
| Pgm2 | Rbbp6 | Ass1 |
| Stam | Gbf1 | Gapdhs |
| Crip2 | Cinp | Rack1 |
| Osbpl1a | Fam117b | Dsg1b |
| Cacng2 | Nid2 | Pip4p2 |
| Cpt1a | Mrpl38 | Grik5 |
| Elmo1 | Crabp2 | Aldh8a1 |

| | | |
|---|---|---|
| Arl6ip5 | Ehmt1 | Acta1 |
| Gga3 | Ubr3 | H2bc4; H2bc6; H2bc8 |
| Pld3 | Dnajc7 | H3-3a; H3-3b |
| Wdr47 | Tor3a | H3c1; H3c10; H3c11; H3c8 |
| Ptpn23 | Ccdc134 | Rasl2-9 |
| Atp1a2 | Nup37 | Prxl2b |
| Ptpn11 | Sppl2b | Gatd3a |
| Cacna2d3 | Mrpl48 | Atp5mf |
| Tomm22 | Rpl24 | Mtnd5 |
| Snap47 | Phospho1 | Tomm70 |
| Syne1 | Mlf2 | Dars1 |
| Cox15 | Dock6 | NARS1 |
| Coro1b | Khdrbs3 | H2ac12 |
| Atp6ap1 | Ntn1 | Naxd |
| Uba1 | Wls | Nectin1 |
| Itgb2 | Larp7 | H2az2 |
| Pde4d | Wdr3 | Pkp1 |
| Slc25a25 | Baz1a | Gsdma |
| Copg2 | Cep76 | Fbxl4 |
| Galk1 | Tubb4b | Eif3j2 |
| Rabgap1 | Ddx42 | Insrr |
| Mthfd1 | Rasa2 | Scn2a |
| Nsdhl | Eed | Prss2 |
| Psmd5 | Mrc2 | H2aw |
| Dpp3 | Med23 | Atp5f1a |
| Pak3 | Icmt | Spart |
| Rab22a | Tk2 | Gars1 |
| Cpne2 | Fgg | F8a1 |
| Hspa12b | Brms1l | Tufm |
| Shisa4 | Sh3bp4 | Rida |
| Shank2 | Nmral1 | Hist1h2bp |
| Cyfip2 | Ppp2r5b | Tprg1l |
| Pgrmc2 | Mtx3 | Mindy2 |
| Poldip2 | Rpl27 | Rab11b |
| Asrgl1 | Usp13 | H2aj |
| Slc7a10 | Tecr | Atp5pd |
| Robo2 | Hist1h1a | Acot10 |
| Cacna2d1 | Caprin1 | H2bc14 |
| Aifm1 | Tusc3 | Hacd3 |
| Numb | Zcchc18 | Gk2 |
| P2ry12 | Rtf1 | Mfsd4a |
| Necap1 | Senp3 | Hist2h2aa1; Hist2h2aa2 |
| Lman2 | Cab39l | Adgrl2 |

| | | |
|---|---|---|
| Tubb3 | Dxo | Tuba3a; Tuba3b |
| Dtd1 | Mrpl1 | Tmem121b |
| Clptm1 | Sidt1 | Palm2 |
| Nt5c | Cbll1 | Sdr9c7 |
| Stx6 | Trim24 | Nccrp1 |
| Dock10 | Mrps33 | Atp5pb |
| Txnl1 | Pf4 | Atp6v1e2 |
| Arfgap1 | Pir | Nme2 |
| Map2k1 | Elavl3 | Gucy1a1 |
| Kbtbd11 | Pm20d1 | RTRAF |
| Clcn4 | Pon2 | H2bc11; H2bc13; H2bc15; H2bc7 |
| Appl2 | Ankrd49 | Wars1 |
| Ndrg4 | Cox7b | H2bc12 |
| Serpinb1a | Bicd2 | Septin5 |
| Ndrg2 | Gclm | Pdyn |
| Cand1 | Rbm25 | Akr1e2 |
| Atg5 | Mapk7 | D1Pas1 |
| Hspa5 | Mtus2 | Rhot2 |
| Gng3 | Dbndd1 | Hist1h2af |
| Rabggtb | Mcat | Mtco1 |
| Slc12a6 | Dmap1 | Stum |
| Mapt | Whsc1 | Timm29 |
| Cndp2 | Rufy1 | Marchf5 |
| Atl1 | Dpy19l1 | Retreg2 |
| Cryz | 5730455P16Rik | Bmerb1 |
| Ppa2 | Nop56 | Serpinb6 |
| Pigs | Zhx1 | Itpr1 |
| Cacng3 | Gbas | Gpr88 |
| Pdha1 | Tuba8 | Sars1 |
| Bcap31 | Ptprz1 | Rab9a |
| Tollip | Sptbn2 | Atp5po |
| Rab3b | Brwd3 | Aars1 |
| Lrrc4b | Isca2 | Kiaa1549 |
| Mpp3 | Sfxn2 | Septin3 |
| Arl8a | Heca | Adgrb3 |
| Plxna2 | Kctd15 | Serpinb5 |
| Vps26b | Paf1 | Lnpk |
| Scai | Rfx3 | S100a14 |
| Ppp2r1a | Sil1 | Babam2 |
| Pde1a | Luzp2 | Hbb-b2 |
| Pdcd10 | Tmco1 | Epb41l1 |
| Hsd17b10 | Plekhh1 | Gstm3 |
| Trappc4 | Fam89b | H2bc9 |

| | | |
|---|---|---|
| Kif21a | Qtrt1 | Abraxas2 |
| Cdipt | Taf6l | Dglucy |
| Fkbp8 | Ostc | Pgm1 |
| Acsl3 | Fam92a1;Fam92a | Septin7 |
| Rnf214 | Nufip2 | Cars1 |
| Ak1 | Nelfa | Rpl11 |
| Cct6b | Serpina3n | Adgrl3 |
| Slc32a1 | RtcA | Plppr3 |
| Pgbd5 | Srp68 | Uchl4 |
| Mat2b | Ttf2 | Fgf14 |
| Cct8 | Ltf | C2cd4c |
| Mical3 | Akr1c18 | Yars1 |
| Mrs2 | Alg1 | Ctnna2 |
| Camk2a | Tfeb | Dsg1a |
| Abcb8 | Ppp4r1 | H2bc1 |
| Gdpd1 | Cdyl | Eif3j1 |
| Glrx3 | Tut1 | H2ax |
| Hagh | Mfn1 | Lrrc15 |
| Mdh1 | Pvrl1 | Mmut |
| Mapre2 | Atp7a | Hist2h2bb |
| Cox6c | Qrsl1 | Relch |
| Sh3glb2 | Cd97 | Acta2 |
| Fbxl16 | Tpr | Adora2a |
| Kif2a | Asph | Calm2 |
| Arrb1 | Lrrc20 | Gsdma3 |
| Ociad1 | Atat1 | Skt |
| Ppp2r2c | Rpusd4 | Elob |
| Cpe | Trafd1 | Rars1 |
| Actr1a | Tfb1m | Mydgf |
| Irgm1 | Prepl | Ubtd1 |
| Rab11a | Tmem106a | Adss2 |
| Dap3 | Sox10 | Actg2 |
| Gpsm2 | Ggcx | Atp5f1d |
| Prdx3 | Cers6 | Atp5mg |
| Lancl1 | Map3k19 | Plpp3 |
| Ap2a1 | Fmr1 | Gpr52 |
| Ppp2r5c | Adamts4 | Mtnd4 |
| Ntng2 | Akr1b8 | Eipr1 |
| Lrrc7 | Otud6b | Dsg1c |
| Prkar2a | C5 | Calml3 |
| Hnrnpa2b1 | Plekha2 | Naxe |
| Rab33b | Kbtbd4 | Hal |
| Cops3 | Rab2b | Septin6 |

| | | |
|---|---|---|
| Capn1 | Mrps26 | Chrnb2 |
| Gabra2 | Yes1 | Eci3 |
| Tomm40l | Polr2c | Tars1 |
| Ndufb7 | Ccdc28b | Dennd11 |
| Tpm2 | Dpyd | Cycs |
| Fth1 | Hbs1l | Gucy1b1 |
| Cmpk1 | Abcb6 | Grk2 |
| Stxbp3 | Tbc1d4 | Atp5me |
| Clic4 | Rbm15 | G6pd2 |
| Prep | Atrnl1 | Cldnd1 |
| Rab24 | Agrn | Plpbp |
| Trpv2 | Txndc15 | Kif17 |
| Slc25a20 | mt-Co1;Mtco1 | Ftl2 |
| Dnaja2 | Pbk | H3c13; H3c14; H3c15; H3c2; H3c3; H3c4; H3c6; H3c7 |
| Dbnl | Card9 | Cyrib |
| Msn | Spry2 | Vars1 |
| Dnajb4 | Rexo2 | Rpl15 |
| Picalm | Dhcr7 | Phf24 |
| Irgq | Eif3g | Prxl2a |
| Fam177a1 | Lig1 | Mtatp8 |
| Vdac1 | Pqbp1 | H2ac7 |
| Psmc5 | Kcna4 | Mtarc2 |
| Exoc5 | Msh6 | Rab1A |
| Hspd1 | Nkrf | Get3 |
| Soga3 | Kdm5d | |
| Isyna1 | Acn9 | |
| Tnr | Cux1 | |
| Stim1 | Magee1 | |
| Mlc1 | Smyd3 | |
| Babam1 | Ssfa2 | |
| Kif1b | Heatr3 | |
| Mtmr2 | Gtpbp1 | |
| Tiam2 | Kiaa0100 | |
| Rap2a | Sp4 | |
| Lin7a | Vps39 | |
| Brinp1 | Nol10 | |
| Mrpl12 | Stk38l | |
| Crat | Fbxo4 | |
| Nefm | Rabl2 | |
| Clip2 | Rad54l2 | |
| Fsd1 | Fbxo45 | |
| Ptpn9 | Tsen34 | |

| | |
|---|---|
| Sar1a | Gjb2 |
| Cadps2 | Fam3c |
| Kif5a | Ostm1 |
| Dctn5 | Zyg11b |
| Tubg1 | Smu1 |
| Gsto1 | Cpn2 |
| Sec61a1 | Man1a2 |
| Erp44 | Samhd1 |
| Ptk2 | Nubp1 |
| Cops7a | Ctdspl2 |
| Pgam2 | Tbc1d22a |
| Slc6a1 | Sel1l |
| Kcnt1 | Nkiras2 |
| Gnb1 | Apoa1bp |
| Ndufs6 | Bax |
| Impact | Lrrc10b |
| Copb2 | Chd2 |
| Psat1 | Ints1 |
| Hdhd3 | Itih3 |
| Lmtk2 | Galnt7 |
| Grm3 | Morc2a |
| Cit | Shpk |
| Anxa3 | Amdhd2 |
| Ctnnb1 | Bclaf1 |
| Acvr1b | Scyl1 |
| Csnk1e | D8Ertd738e |
| Trap1 | Rims3 |
| Pcbp1 | Lama1 |
| Cldn11 | Ik |
| Plxnb2 | Wdhd1 |
| Hint2 | Wdr83 |
| Hint3 | Neu2 |
| Igbp1 | Nxt2 |
| Micu1 | 02-Sep |
| Strap | Chmp2a |
| Arfgef1 | Htatip2 |
| Psma2 | Zfand1 |
| Ngef | Lilrb4 |
| Sh3kbp1 | H2afy |
| Lgalsl | Glra2 |
| Ggct | Arhgef2 |
| Ndrg1 | Arhgef1 |
| Fbxo2 | Rell1 |

| | |
|---|---|
| Inpp4a | Sltm |
| Atp6v0a1 | Znf365 |
| Lrp1 | Prkcdbp |
| Synpo | Map4k2 |
| Ina | Mpc1 |
| Stx16 | Pbdc1 |
| Baiap2 | Urb1 |
| Rnh1 | Meis2 |
| Mrps31 | Nudt11;Nudt10 |
| Srcin1 | Anks1a;Anks1 |
| Ap2m1 | Zbtb40 |
| Snx2 | Tpcn1 |
| Add3 | Gtf3c1 |
| Cfl1 | Thoc7 |
| Asphd2 | Frmpd4 |
| Ppib | Npas3 |
| Pitpnm1 | Fpgt |
| Pik3c3 | Ept1 |
| Adcy9 | Pde8a |
| Rcn2 | Wdr92 |
| Prdx2 | Smarcd3 |
| Slc7a2 | Arhgdib |
| Ncs1 | Diap1;Diaph1 |
| Tenm4 | Leo1 |
| Map1b | Ints8 |
| Prmt1 | Ighv1-31 |
| Por | Apbb1ip |
| Kcna2 | Hnrnpf |
| Aspa | Alkbh6 |
| Scp2 | Wdr36 |
| Mapk3 | Morf4l1 |
| Vgf | Tnfaip8 |
| Hsp90b1 | Slc38a10 |
| Cbr4 | Clec12a |
| Srgap3 | Luc7l3 |
| Igsf8 | Slc22a6 |
| Arf4 | Cog8 |
| Rplp0 | Bhlhe22 |
| Ivd | Uprt |
| Rabgef1 | Epha5 |
| Gstk1 | Prkcz |
| Tsfm | Panx2 |
| Agap3 | Fads2 |

| | |
|---|---|
| Dhrs4 | Lima1 |
| Vim | Hira |
| Tamm41 | Plekhm2 |
| Lzts3 | Piezo1 |
| Slc38a3 | Psmd10 |
| Flot2 | Frmd8 |
| Hyou1 | Pus7l |
| Psmb5 | Eps8 |
| Rps6ka2 | Pphln1 |
| Slc25a13 | Rps27a |
| Gch1 | Dph5 |
| Neto2 | Mrpl33 |
| Ryr3 | Hnrnpdl |
| Arhgef7 | Esyt1 |
| Fam126b | Prdm16 |
| Aco2 | Zfp280d;Znf280d |
| Asic1 | Mapkbp1 |
| Nrbp2 | Ncbp1 |
| Abhd12 | Stard5 |
| Pam | Pyhin1 |
| Rab7a | Wwc1 |
| Phyhipl | Tyw3 |
| Heatr5b | Lamtor3 |
| Ache | Bst2 |
| Entpd2 | Man1c1 |
| Agpat5 | Dip2c |
| Cpne9 | Hps3 |
| Fxyd6 | Snd1 |
| Ptprn2 | Men1 |
| Alcam | Nacc2 |
| Lamp2 | Ankrd27 |
| Sorbs2 | Mta2 |
| Ube2v2 | Pts |
| Gmpr | Cdc123 |
| Arhgap32 | Fmo1 |
| Csnk2a1 | Frg1 |
| Ube2o | Znf316;Zfp316 |
| Stx1b | Rangap1 |
| Stip1 | Shisa6 |
| Elmo2 | Tardbp |
| Celsr2 | Mybbp1a |
| Gabbr2 | Riok2 |
| Ppm1f | Map7d1 |

| | |
|---|---|
| Prkcsh | Ears2 |
| Hexb | Pcna |
| Napa | Arhgap6 |
| Atp2b4 | Fadd |
| Rufy3 | Dhtkd1 |
| Avl9 | Ammecr1l |
| Cltb | Nfyb |
| Elmod2 | Gm20547;Cfb |
| Ppp2r5e | Lias |
| Tmem163 | Ipo13 |
| Oxsr1 | Lepre1 |
| Arpc5 | Aktip |
| Endod1 | Postn |
| Rdh14 | Nploc4 |
| Gaa | Cd2ap |
| Gsn | Tomm7 |
| Tdrkh | Eri3 |
| Ehd1 | Smg5 |
| Mapk10 | H6pd |
| Cpt1c | Alg10b |
| Kcnab2 | Znf428;Zfp428 |
| Slc44a2 | Cox7a2 |
| Exoc7 | Sec61g |
| P4hb | Brd8 |
| Plcl2 | Ensa |
| Ablim2 | Pomgnt2 |
| Itsn1 | Jmy |
| Psmd3 | Golm1 |
| Actr3 | Hic2 |
| Lasp1 | Smarca2 |
| Vti1b | Wdr34 |
| Stx1a | Crot |
| Pcsk2 | Ppm1k |
| Bckdk | Acads |
| Mtch2 | Prpf39 |
| Erc1 | Mien1 |
| Ipo7 | Myo7a |
| Rab1b | Rwdd1 |
| Apoe | Tom1l1 |
| Cntfr | Rexo1 |
| Agfg1 | Nol4 |
| Ddhd2 | Zmym2 |
| Fmnl2 | Mcee |

| | |
|---|---|
| Plcxd3 | Ppp1r3g |
| Ckap5 | Tmed7 |
| Gltp | Kcnq2 |
| Rab35 | Lgi2 |
| Hgs | Smad5 |
| Slc25a44 | Cplx3 |
| Apmap | 1810009A15Rik |
| Creld1 | Nek7 |
| Pafah1b2 | 1700112E06Rik |
| Cask | Sqle |
| Daam2 | Isoc2b |
| Syn3 | Aplp1 |
| Gabrd | Tmem167a;Tmem167 |
| Gnpda1 | Sumf1 |
| Acox1 | Sclt1 |
| Grik2 | Nt5dc2 |
| Cacna1e | Sh3pxd2a |
| Ppp2r2a | B630019K06Rik |
| Srr | Sumf2 |
| Rps3 | Pde7b |
| Reps2 | Igsf11 |
| Eif3i | Pcdh9 |
| Atp6v1c1 | Cdk19 |
| Tspan7 | Recql |
| Tmx2 | Amz2 |
| Rab11fip 2 | Idh3b |
| Hsph1 | Helb |
| Tagln3 | Hist3h2ba;Hist3h2bb |
| Anxa1 | Phlda3 |
| Atg9a | Ring1 |
| Clpp | Neurl4 |
| Fn3k | Khsrp |
| Guk1 | Enpep |
| Comtd1 | Pa2g4 |
| Rras2 | Ttc19 |
| Tmod2 | Grtp1 |
| Pcca | Plekha5 |
| Susd2 | Acbd5 |
| Cacnb1 | Tor1aip1 |
| Dgkz | Rabif |
| Tmed9 | Lyz1 |
| Sncb | Cnot11 |
| Ddah1 | Fgfr1 |

| | |
|---|---|
| Pacsin1 | Wdyhv1 |
| Ergic1 | Khnyn |
| Dgkq | Plod1 |
| Ctnnd1 | Pcif1 |
| Rpl27a | Pcdh17 |
| Ptp4a1 | Minos1 |
| Mal2 | Trpm3 |
| Ampd2 | Lrrc75a |
| Gsk3b | Mxra8 |
| Igsf21 | Znrf2 |
| Hibch | Tmem151a |
| Cdip1 | Shf |
| Vbp1 | Taok2 |
| Plxna4 | Dennd6a |
| Spryd7 | Hax1 |
| Atg3 | Matr3 |
| Rab4b | Tmem38a |
| Hcn1 | Cacna1c |
| Arhgap1 | Hist2h2ab |
| Dctn4 | Ccdc91 |
| Plgrkt | Dcaf11 |
| Acsl1 | Rab11b;Rab11a |
| Slc8a1 | Pola1 |
| Aldh4a1 | Gnl2 |
| Sars2 | Aga |
| Ppp3cb | Eif3b |
| Syngr3 | Thap11 |
| Syt1 | Igha |
| Tsc1 | Acox3 |
| Agap2 | Crnkl1 |
| Ppp1cc | Igfbp5 |
| Slc4a4 | Ptdss2 |
| Ubfd1 | Fam50a |
| Huwe1 | Rbp1 |
| Akr1a1 | Zc3h6 |
| Ccdc6 | Gorab |
| Prkacb | Tmtc3 |
| Ndufs3 | Cdv3 |
| Gpd1l | Rwdd2a |
| Ap2b1 | Gstt1 |
| Dmxl2 | Limk1 |
| Eif4h | Ugt1a6 |
| Eif4a2 | Tstd3 |

| | |
|---|---|
| Oscp1 | Lrrc73 |
| Faah | Arap2 |
| Rhog | Ctage5 |
| Prkca | Helz2 |
| Mgll | Serpinb9 |
| Efr3a | Vtn |
| Pdcd6 | Eif2s2 |
| Kif5c | Pbxip1 |
| Pdp1 | Desi2 |
| Eppk1 | Nudcd2 |
| Rab14 | Adap1 |
| Bckdha | Pias4 |
| Rgs8 | Fam32a |
| Idh1 | Txn |
| Ly6h | Zfp943 |
| Me2 | Rcc1 |
| Gart | Lcorl |
| Ncam2 | Adar |
| Itm2c | Nosip |
| Hsd17b1 1 | Dlg5 |
| Cntnap1 | Sh3pxd2b |
| Gmds | Xrcc4 |
| Acot2 | Serhl |
| Ddc | Muc5b |
| Vipas39 | Plin4 |
| Ermn | Stk25 |
| Fry | Pex3 |
| Ank3 | Fam49a |
| Armc6 | Hck |
| Atcay | Ppp2r3a |
| Ap3m1 | Ppp1r1a |
| Aldh9a1 | P4ha2 |
| Cttn | Ifi35 |
| Stxbp1 | Snrpa1 |
| Zc2hc1a | Adcy3 |
| Wdr44 | Rai14 |
| Ndufv2 | Tomm34 |
| Rasal1 | Ppfia4 |
| Ndufaf5 | Ccar1 |
| Cntn2 | Abracl |
| Tpp2 | Ggact |
| Pkp4 | H3f3a;H3f3c |
| Gstp1 | Parl |

| | |
|---|---|
| Pef1 | Rabep2 |
| Cyb5a | Ociad2 |
| Lrrc8a | Ndufaf7 |
| Ppme1 | Pask |
| Shisa9 | Rad21 |
| Slc25a22 | Pvr |
| Dnajb6 | Prex1 |
| Lrrtm3 | Tgfb1i1 |
| Gnai2 | Prmt7 |
| Wwox | Rnf34 |
| Vcl | Lipe |
| Pdxp | Bai3 |
| Ywhaq | AW551984 |
| Coq9 | Cbln1 |
| Ncald | Igf2 |
| Aldh2 | Disp2 |
| Ywhah | Lsm6 |
| Camsap3 | Evi2b |
| Psmc1 | Cnot1 |
| Actr3b | Hnrnpa0 |
| Ahcyl2 | Anapc5 |
| Ap1b1 | Sybu |
| Clvs2 | Cyb5r4 |
| Arpc1a | Gatc |
| Ppm1e | Efna3 |
| Snap91 | Rraga |
| Rap1a | Gtf2a2 |
| Astn1 | Eif1b |
| Ppp5c | Fam193a |
| Psph | Stx18 |
| Slc38a1 | Znf687 |
| Arih1 | Itgal |
| Slc17a6 | Crocc |
| Gad1 | Mef2c |
| Omg | Ghdc |
| Gss | Sipa1l2 |
| Pank4 | Nhsl2 |
| Tmem33 | Prelp |
| Arf2 | Myo1c |
| Nrcam | Ccdc109b |
| Vps53 | Coq7 |
| Txndc5 | Ilf2 |
| Ttc39c | Zfp318 |

| | |
|---|---|
| Camk1d | Ppif |
| Psmf1 | Fam206a |
| Cdk5 | Zdhhc21 |
| Letmd1 | Mcm7 |
| Dlgap3 | Ufl1 |
| Strn4 | Nup54 |
| Arpc5l | Kdm1a |
| Grid1 | Sestd1 |
| Hepacam | Hist1h3b |
| Napb | Oxa1l |
| Ldhb | Rpl11;Gm10036 |
| Cystm1 | Thoc1 |
| Sv2c | Nup107 |
| Pdia3 | Glt8d1 |
| Syn1 | Stat5b |
| Cadm4 | Hmgn2 |
| Sord | Ston2 |
| Rab3gap1 | Atg10 |
| Zdhhc5 | Stag1 |
| Psmd2 | Tex264 |
| Gmfb | Trub1 |
| Iqsec1 | Kars |
| Ipo5 | Nudt2 |
| Usp46 | Mrpl53 |
| Lrba | Igsf5;Pcp4 |
| Pi4ka | Rps16 |
| Snx7 | Sema3a |
| Emd | Taf5 |
| Cpne7 | Tanc1 |
| Pebp1 | Atp5c1 |
| Uba5 | Fam69a |
| Tbc1d17 | Pknox1 |
| Arl6ip1 | Mtatp8;mt-Atp8 |
| Apeh | Ubac1 |
| Ndel1 | Gusb |
| Scamp1 | Lman2l |
| Mdga2 | Zc3h11a |
| Asl | Ralbp1 |
| Grm7 | Slc14a1 |
| Rundc3a | Trmt10c |
| Aldh18a1 | Mrpl54 |
| Slc3a2 | Iws1 |
| Ppm1a | Zfp91 |

| | |
|---|---|
| Mark2 | Kif13a |
| Mlycd | Cbs |
| Efnb3 | Reep1 |
| Abhd3 | Ppp1r13b |
| Prickle2 | Ncapg |
| Zer1 | Klkb1 |
| Idh2 | Tax1bp1 |
| Katnal1 | Clasrp |
| Purb | Mug1 |
| Vps13c | Sec11a |
| Rpn1 | Nipal4 |
| Tbcel | Ecd |
| Dpp10 | Tulp4 |
| Cd38 | Atox1 |
| Dhodh | Phactr3 |
| Cacnb3 | Slc25a27 |
| Acadsb | Pde5a |
| Ppp1cb | Tdg |
| Lpcat4 | Exosc6 |
| Slc7a5 | Ctps2 |
| Nrxn2 | Fen1 |
| Aprt | Uba6 |
| Cyc1 | Trrap |
| Adrbk2 | Fundc1 |
| Prkab2 | Klhl11 |
| Snta1 | Mmp12 |
| Acad9 | Far1 |
| Crmp1 | Nfkb2 |
| Mtap | Son |
| Gna11 | Ift81 |
| Ndufb11 | Entpd7 |
| Sgta | Irg1 |
| Uggt1 | Cdh1 |
| Atp6v1g2 | Rps26 |
| Ubr4 | Chadl |
| Lingo2 | Camkmt |
| Dgkb | Kctd3 |
| Gstm7 | Ptpn1 |
| Abhd6 | Asb6 |
| Smap1 | Dolk |
| Anxa2 | Syndig1 |
| Map6 | Nbn |
| Cc2d1a | Hist2h2ac;Hist2h2aa1;Hist1h2al |

| | |
|---|---|
| Dhrs7 | Ptpn18 |
| Rars2 | Spryd4 |
| Vac14 | Sfmbt1 |
| Vamp1 | Slc35f3 |
| Prrt3 | Fam160a2 |
| Rtn3 | Ncoa2 |
| Pgam5 | Tbce |
| Abi1 | Fhod3 |
| Tenm2 | Txn2 |
| Pdlim5 | Rrp12 |
| Ndufa2 | Fam98a |
| Nedd4l | Triap1 |
| Mri1 | Iscu |
| Snx30 | Qil1 |
| Ankrd34b | Cpsf6 |
| Cdh10 | Ubxn7 |
| Csde1 | Ca11 |
| Acsf3 | Tfip11 |
| Pak1 | Ccdc47 |
| Naca | Snrpd2;Gm5449 |
| Kcnj10 | Rftn1 |
| Ubqln2 | Slitrk4 |
| Tsg101 | Prdm8 |
| Fyn | Mki67 |
| Kirrel3 | Eml6 |
| Cops5 | Arhgap20 |
| Cadps | Tuba3a |
| Apoo | Cd59a |
| Ncstn | Dcp1a |
| Pccb | Lancl3 |
| Fabp5 | Gstt2 |
| Pmpca | Gjc2 |
| Asah1 | Nudt18 |
| Kcnma1 | Gdf10 |
| Gnb5 | Psme3 |
| Snx4 | Cthrc1 |
| Pcyt2 | Elac1 |
| Slc2a13 | Ethe1 |
| Ampd3 | Sbds |
| Unc13b | Txlna |
| Actbl2 | Trove2 |
| Nck1 | Tmem245 |
| Lcp1 | Patl1 |

| | |
|---|---|
| Tubg2 | Prpf4b |
| Slc6a9 | Sugct |
| Arpc3 | N6amt1 |
| Cntn4 | Armcx4 |
| Kctd12 | Kdm2a |
| Surf1 | Svil |
| Micu3 | Epg5 |
| Wdr7 | Cyp27a1 |
| Rtcb | Cd84 |
| Hace1 | Abcg1 |
| Arg1 | Traf6 |
| Dnajb1 | Dao |
| Tuba1a | Tacc1 |
| Psmc3 | Cnnm2 |
| Ogt | Thoc3 |
| Pdk3 | Sec14l3 |
| Cct3 | Myh1 |
| Palm | Ep400 |
| Isoc1 | BC037034 |
| Bcap29 | Btbd11 |
| Rmnd1 | Prkaa1 |
| Nptx1 | Fbxo22 |
| Rab18 | Urgcp |
| Txnrd1 | Mcam |
| Dclk1 | Pycr1 |
| Arfip2 | Polr2a |
| Tcp1 | Cyp7b1 |
| Gnal | Rprd1b |
| Vcpip1 | Gsta3 |
| Emc1 | Tmco3 |
| Arhgap23 | I830012O16Rik |
| Ptpn5 | Cmbl |
| Adam22 | Rassf2 |
| Tiprl | Ikbke |
| Mrps23 | Cyth1 |
| Diras1 | Lsm14b |
| Ndufa6 | Crbn |
| Acot9 | Zc3h14 |
| Bcl2l13 | Chmp6 |
| Gpr158 | Brinp3 |
| Abhd17a | Rab11fip1 |
| Ndufc2 | Tesk1 |
| Nebl | Obp1a |

| | |
|---|---|
| Slc25a42 | Bet1 |
| Shank3 | Kdsr |
| Nefh | Wdr4 |
| Cdc37 | Usp11 |
| Svop | Nutf2 |
| Gapdh | Myl12a |
| Aldh1a7 | Hdac9 |
| Ech1 | Cntnap5a |
| Cplx2 | Taf7 |
| Gfra2 | Gucy1a2 |
| Cyld | Actl6b |
| Lrp1b | Caap1 |
| Cox5a | Pfdn2 |
| Armc8 | Dnttip2 |
| Sucla2 | Jund |
| Cyp46a1 | Ulk1 |
| Atp2b2 | Ophn1 |
| Shisa7 | Filip1l |
| Commd3 | Syne3 |
| Gsta4 | S100a13 |
| Myo6 | Afm |
| Arl15 | AI837181;Bles03 |
| Afg3l2 | Eif4e2 |
| Hecw2 | Nkiras1 |
| Gnas | Acad12 |
| Jup | Taf10 |
| Gja1 | Ufd1l |
| Erlin2 | Prkab1 |
| Lysmd2 | Spg21 |
| Tmem256 | E2f3 |
| Fermt2 | Nmnat3 |
| Smyd5 | Ints6 |
| Eef1a2 | Sdc4 |
| Mfsd6 | Col4a2 |
| Slc6a6 | Polr1d |
| Ndufaf3 | Pes1 |
| Rgs6 | Wbscr27 |
| Zzef1 | Dclk2 |
| Gria2 | Rpl7 |
| Chordc1 | Rbm45 |
| Hpcal1 | Skiv2l |
| C2cd5 | Sgcd |
| Hnrnph2 | Fbxw11 |

| | |
|---|---|
| Iglon5 | Zbtb20 |
| Lrrtm1 | Dnal4 |
| Ntrk3 | Map9 |
| Slc2a3 | Commd2 |
| Cops8 | Atxn10 |
| Gmppa | Ccdc120 |
| Elfn2 | Ctsh |
| Atg2b | Stk10 |
| Abr | D3Ertd751e |
| Slc6a17 | Cdadc1 |
| Cnnm1 | Ankrd29 |
| Pitrm1 | Psd |
| Adam11 | Wbscr17 |
| Gabrg2 | Apoc1 |
| Dpysl4 | Nat10 |
| Gpd2 | Map3k5 |
| Dctn1 | Pltp |
| Gmps | Tbc1d12 |
| Ctnnd2 | Sap30l |
| Sorcs3 | Slit2 |
| Ddx1 | Obsl1 |
| Fgf12 | Slc16a3 |
| Fibp | Cwc27 |
| Stmn2 | Tubb1 |
| Ak5 | Celf5 |
| Glod4 | Tfe3 |
| Uqcrq | Gle1 |
| Vps25 | Dvl1 |
| Ezr | Mcl1 |
| Dync1li1 | Uevld |
| Nap1l1 | Sirt1 |
| Rps13 | Nt5m |
| Tm9sf3 | Dnajc13 |
| Tesc | Fam163b |
| Grpel1 | Grwd1 |
| Eea1 | Chka |
| Camkk2 | Rsph1 |
| Gdi2 | Eif4a3;Gm8994 |
| Cul2 | Hmgn5 |
| Ndufs1 | Clasp1 |
| Pde2a | Prkrip1 |
| Gpm6b | Mrpl42 |
| Epb41 | Gm16039 |

| | |
|---|---|
| Mtmr1 | Kiaa0513;6430548M08Rik |
| Nlgn2 | Csrp3 |
| Coq3 | Apc2 |
| Agk | Mndal |
| Pip4k2c | Syvn1 |
| Exoc8 | Nnt |
| Thnsl1 | Plin2 |
| Vsnl1 | Emc9 |
| Git1 | Phf5a |
| Acsl6 | Ddx5 |
| Opa1 | Rgp1 |
| Dbn1 | Fam13c |
| Vps33b | Usp29 |
| Hsp90aa1 | Suds3 |
| Arpc2 | Myl4 |
| Pnp | Akap10 |
| Sh3gl1 | Rpl39 |
| Olfm2 | Nabp2 |
| Dlg1 | Zfyve9 |
| Them4 | Frmd4a |
| Tmem30a | Xpo6 |
| Sema7a | Fbll1 |
| Brsk1 | Flnb |
| Ptrh2 | Mrps7 |
| Fkbp1a | Hpgds |
| Tppp3 | Limch1 |
| Mlec | Tmem177 |
| Slc12a9 | Upf3b |
| Thop1 | Bach1 |
| Slc12a2 | Kin |
| Aldh1l1 | Ict1 |
| Jam3 | Gpc5 |
| Kalrn | Fstl5 |
| Vkorc1l1 | Ctdsp1 |
| Ddx19a | Zfp384 |
| Sugt1 | Arhgap27 |
| Vps26a | Fgf1 |
| Hspa12a | Ebp |
| Dlat | BC100530 |
| Cd200 | Pam16 |
| Gba2 | Lonp2 |
| Arcn1 | Spata13 |
| Aldh7a1 | U2surp |

| | |
|---|---|
| Atp2a3 | Serpinb6;Serpinb6a |
| Slk | Vav2 |
| Mtx1 | S100a9 |
| Cyb5r1 | Grk6 |
| Iqcb1 | Nup35 |
| Gabrb2 | Pdia5 |
| Ist1 | Timp3 |
| Xpo1 | F13a1 |
| Coro1a | Foxk1 |
| Sco1 | Bpifa2 |
| Epm2aip 1 | Rpl8 |
| Pdpr | Rab3il1 |
| Nme1 | Lsm2 |
| Abcg2 | Serpinf2 |
| Slc25a11 | Sap130 |
| Lmbrd2 | Sowaha |
| Uqcc1 | Lmnb1 |
| Caskin1 | Sts |
| Wdr54 | 2310035C23Rik;Kiaa1468 |
| Mtfr1l | Stx5;Stx5a |
| Tmem65 | H2afy2 |
| C2cd2l | Lig3 |
| Sipa1l1 | Krit1 |
| Mccc1 | Peli1 |
| Blvra | Arfip1 |
| Erp29 | Wdsub1 |
| Ube2k | Smarcc2 |
| Hsdl1 | P2rx7 |
| Rnpep | Ylpm1 |
| Epn1 | Nfat5 |
| Rap1gap | Med17 |
| Sdhd | Ostf1 |
| Aqp4 | Ebna1bp2 |
| Cnr1 | Rps4l |
| Lamtor1 | AI848285 |
| Bsn | Bcam |
| Lrtm2 | Gtf3c3 |
| Pde1b | Inip |
| Tbcb | Znf512 |
| Dnajc5 | Ktn1 |
| Hk2 | Ntpcr |
| Rab6a | Kiaa1109 |
| Diras2 | Pros1 |

| | |
|---|---|
| Pik3r4 | Ptbp2 |
| Ncdn | Slc6a8 |
| Pck2 | Nell1 |
| Psmb1 | Smchd1 |
| Synpr | Dpp9 |
| Rhot1 | Mthfd2l |
| Atg16l1 | Birc2 |
| Twf2 | Armc9 |
| Ftl1 | Scd2;Scd3 |
| Hpcal4 | Shoc2 |
| Eif4b | Stk16 |
| Maoa | Atp9b |
| Map1lc3a | Arhgef11 |
| Vps51 | Tbc1d1 |
| Sv2a | Kdelc1 |
| Ado | Slc15a3 |
| Glud1 | Ssb |
| Psmd7 | Atxn3 |
| Ephx2 | H2-D1 |
| S1pr1 | Fars2 |
| Cad | Gtpbp3 |
| Actb | Pcnt |
| Eno1 | Meaf6 |
| Actr2 | Ccrn4l |
| Prrt1 | Neu1 |
| Ap3b1 | Arhgef4 |
| Pip5k1b | Urb2 |
| Slc4a8 | Ubl7 |
| Mpi | Sphk1 |
| Adam10 | Mettl1 |
| Wdr1 | Cplx1 |
| Spg7 | Mfge8 |
| Nptxr | Tssc1 |
| Ndufa13 | Gfod2 |
| Bdh1 | Bbox1 |
| Tpd52l1 | Sh3bgrl3 |
| Ndufa4 | Epha3 |
| Eef1b | Grid2ip |
| Ap1g1 | Nono |
| Memo1 | Zfyve28 |
| Cacna1a | Serpinb10 |
| Snx1 | Mturn |
| Ap1m1 | Ahsg |

| | |
|---|---|
| Usp15 | Gid8 |
| Eml5 | Zfand6 |
| Rapgef2 | Aebp1 |
| Klc1 | Stard3 |
| Mpst | Stt3b |
| Spcs2 | Ttc4 |
| Pfn1 | Lppr2 |
| Ube3a | Agfg2 |
| App | R3hdm2 |
| Ndufa11 | Notch2 |
| Atp6v1a | Tmem214 |
| Plxnd1 | Clcn2 |
| Cct5 | Hmgb1 |
| Exoc3 | Avp |
| Ndufb5 | Ankrd44 |
| Fah | Zfyve21 |
| Basp1 | Smarcd1 |
| Hip1r | 2810408M09Rik |
| Cap2 | Oas3 |
| Dad1 | Mt2 |
| Fundc2 | Ctnnbl1 |
| Fkbp4 | Zfp62 |
| Gng7 | Hps5 |
| Xpnpep1 | Synj2 |
| Mras | Dnajc24 |
| Wdr13 | Enpp4 |
| Necab1 | Gnptab |
| Sirt5 | Hif1an |
| M6pr | Fcgr2 |
| Tsc2 | Sumo2 |
| Ugp2 | Prim1 |
| Hspa8 | Ctnna1 |
| Tmem11 | Igf2bp3 |
| Agpat1 | Cox20 |
| Pcyox1l | Rpl10 |
| Kifap3 | H1f0 |
| Tnpo3 | Alkbh8 |
| Kif1a | Ddx20 |
| Abl2 | Lrrc4 |
| Epb41l2 | Zc3hav1l |
| Eif5a2 | Elavl4 |
| Nap1l4 | Fbn2 |
| Kcna6 | Tyw5 |

| | |
|---|---|
| Itpa | Dkc1 |
| Nme3 | Pla2g15 |
| Acsbg1 | Hgsnat |
| Ciapin1 | Znf207;Zfp207 |
| Psma6 | Gtf2f2 |
| Tppp | Hist1h1d |
| Tfam | Ankrd13a |
| Cxadr | Btbd1 |
| Acp1 | Zfp292 |
| Pgap1 | Raph1 |
| Ppp6r2 | Unc80 |
| Dbt | Mapkapk2 |
| Armc10 | U2af1 |
| Ptpre | Mrpl23 |
| Arhgap44 | Golga4 |
| Cdk17 | Yipf3 |
| Ica1 | Mthfr |
| Atpaf1 | Dnm2 |
| Upf1 | Map2k7 |
| Lrrc4c | Gabarapl1 |
| Gng2 | Serac1 |
| Hadhb | Cd99 |
| Man2c1 | Tmem68 |
| Etfdh | Mettl10 |
| Ap3s2 | Ube2f |
| Capza1 | Cers2 |
| Rragd | Ufsp2 |
| Rnf14 | Impdh1 |
| Atp1b2 | Rbm15b |
| Hmgcl | Rcsd1 |
| S100a11 | Psap |
| Anxa6 | Frs3 |
| Scrn1 | Thra |
| Ric8a | Sfxn4 |
| Pclo | Tra2b |
| Pip4k2a | Fut8 |
| Nudt16 | Aox1 |
| Sparcl1 | Cryba4 |
| Rab5b | Grasp |
| Rock1 | Samd4b |
| Slc25a24 | Cog3 |
| Abcd3 | Ptpmt1 |
| Lrrtm4 | Dnajc9 |

| | |
|---|---|
| Ndufaf1 | Tmsb15l;Tmsb15b2 |
| Acadl | Pram1 |
| Itfg1 | Rpl22 |
| Kctd8 | Rpl36a |
| Rhoc | Sntb1 |
| Prpsap1 | Copz1 |
| Ccdc127 | Spcs3 |
| Myo5b | Irf8 |
| Hspa4l | Kcmf1 |
| Syngap1 | Sec62 |
| Gria4 | Pias2 |
| Vwa5a | Cox17 |
| Gpi | Ibtk |
| Dstn | Chchd4 |
| Sdr39u1 | Cdc26 |
| Ppp1r21 | Abcc1 |
| Ssr4 | Slc22a4 |
| Cdc42ep4 | Lypd1 |
| Dip2a | Kifc2 |
| Emc3 | Mavs |
| Grhpr | Ccs |
| Extl2 | Mina |
| Gclc | Atp5l |
| Dmtn | Tax1bp3 |
| Auh | Dgkh |
| Trio | Aip |
| Cntnap4 | Arnt2 |
| Pnpla8 | Fndc3a |
| Ap3d1 | Ikbkg |
| Dock4 | Telo2 |
| Mog | Fam107a |
| Lypla2 | Alpk1 |
| Rdh11 | Nt5c3b |
| Th | Stap1 |
| Mrpl39 | Xylt1 |
| Nomo1 | Ccdc93 |
| Ndufs8 | Vangl1 |
| Snx16 | Fkbp3 |
| Dnajb11 | Rpl18 |
| Lin7b | Rfc5 |
| Vcam1 | Nrgn |
| Prdx5 | Cnn2 |
| Trim9 | Lphn1 |

| | |
|---|---|
| Adcy5 | Map4k4 |
| Itm2b | Zkscan1 |
| Gad2 | Eln |
| Pgd | Rab29 |
| Ndufa8 | Prpf8 |
| Dync1i1 | Rnf2 |
| Flad1 | Ptn |
| Akr1b1 | Acbd3 |
| Acaa2 | Lrfn3 |
| Itpr3 | Crtap |
| Enpp6 | Scn8a |
| Nae1 | Mboat2 |
| Ddb1 | Gcn1l1 |
| Pgk1 | Rit2 |
| Oxr1 | Cln5 |
| Hspbp1 | 09-Sep |
| Trim2 | Epb4.1l1 |
| Gpx1 | Specc1 |
| Mblac2 | Tsc22d1 |
| Etfb | Tspan6 |
| Clu | Mprip |
| Ufc1 | Fam213a |
| Lhfpl4 | Mef2a |
| Canx | Pcdh10 |
| Coq5 | Stac2 |
| Lrrc47 | Ovca2 |
| Timm44 | Ppih |
| Kcnip2 | Farp2 |
| Emb | Uckl1 |
| Shank1 | Plcb4 |
| Abhd16a | Qars |
| Dlst | Cptp;Gltpd1 |
| Uqcrc1 | Spred3 |
| Scn1b | Fxr1 |
| Gabra4 | Endog |
| Gfap | Rbmxl1 |
| Cadm2 | Plekha7 |
| Rps9 | Ttc21b |
| Sh3gl3 | Slc20a2 |
| Fnbp1 | Gan |
| Cpne5 | Shd |
| Dlg3 | Wdr18 |
| Psmb3 | Pik3ap1 |

| | |
|---|---|
| Efhd1 | Celf4 |
| Eml2 | Polb |
| Camk2g | Lamtor5 |
| Phgdh | Mpp5 |
| Nqo2 | Acat3 |
| Pdia6 | Bzw1 |
| Magi2 | Wdfy2 |
| Ralgapa1 | Apc |
| Crym | Unc13d |
| Trappc12 | Mrpl15 |
| Slc25a1 | Akap9 |
| Arl3 | Tsr1 |
| Nceh1 | Gls2 |
| Rab2a | Serbp1 |
| Zmpste24 | Kptn |
| Slc2a1 | Bcas2 |
| Cep170b | Nvl |
| Eci2 | Slitrk2 |
| Scyl2 | Rab3d |
| Serinc1 | Tpm4 |
| Rab31 | Naa15 |
| Enoph1 | Kcnj3 |
| Ranbp1 | Tmed1 |
| Dpysl2 | Diablo |
| Mocs3 | Dynlt3 |
| Fnbp1l | Ift122 |
| Cdc42bpb | Cnnm4 |
| Clybl | Cdc42ep2 |
| Frrs1l | Pex1 |
| Tubgcp2 | Rpgr |
| Rab8a | Syap1 |
| Them6 | Mon1a |
| Brcc3 | Tmem55b |
| Tubb2b | Mmaa |
| Psd3 | Slc1a5 |
| Psmd9 | Taf2 |
| Scamp3 | Snapin |
| Gspt1 | Dtx3l |
| Nf1 | Sema6a |
| Prmt5 | Gm15800 |
| Qdpr | Tab2 |
| Atl2 | Lrrc58 |
| Pafah1b1 | Ddx59 |

| | |
|---|---|
| Rpl12 | Wdr11 |
| Sco2 | Uimc1 |
| Usp5 | Phka2 |
| Lmtk3 | Myo10 |
| Uhrf1bp1 l | Ugt8 |
| Abcb7 | Lhx2 |
| Tln2 | Ube2g2 |
| Ak3 | Ogdhl |
| Mpp1 | Prkcq |
| Snph | Ireb2 |
| Rab4a | Stmn4 |
| Ube2l3 | Uck2 |
| Tmod1 | Gstm4 |
| Stxbp5l | Clptm1l |
| Cyb5r3 | Kmt2d |
| Otub1 | Tmsb4x |
| Blvrb | Nudt9 |
| Sar1b | Ahnak2 |
| Ak2 | 3110043O21Rik |
| Sarm1 | Mob1a |
| Dctn3 | Coro6 |
| Hsd17b4 | Dpp7 |
| Smpd3 | Ncapd2 |
| Ermp1 | Baalc |
| Psmd11 | Rps11 |
| Glo1 | Snrpa;Gm5145 |
| Ntrk2 | Angel2 |
| Dhrs7b | Mvb12a |
| Gpr37l1 | Eif2b5 |
| Trappc3 | Wnk3 |
| Tom1l2 | Itfg3 |
| Etfa | Gprasp2 |
| Cend1 | Pml |
| Stk24 | Dcaf13 |
| Vps4b | Rrp7a |
| Stt3a | C8a |
| Necab2 | Phf3 |
| Adam23 | Sptlc1 |
| Pick1 | Atp11b |
| Spag9 | Rbm28 |
| Drd1 | Krt36 |
| Gabra1 | Vldlr |
| Kcnc1 | Ralgps1 |

| | |
|---|---|
| Rac3 | Ptgfrn |
| Arl8b | Mroh1 |
| Mpp2 | Isg15 |
| Sec13 | Zgpat |
| Vcan | Yap1 |
| Stx8 | Vrk2 |
| Capns1 | Serpinb6b |
| Dock3 | Nudt12 |
| Cacng4 | Pcmtd1 |
| Rab3a | Pde1c |
| Magi1 | Naaa |
| Macf1 | Lurap1 |
| Lyst | Ddx19b |
| Bcl2l1 | Wipf1 |
| Myo5a | Icam1 |
| Cpd | Rqcd1 |
| Eef2 | Btd |
| Kcnip4 | Lpar1 |
| Ubqln1 | Ptcd2 |
| Atp6ap2 | Ahsa2 |
| Cryl1 | Rbx1 |
| Gpsm1 | Rmdn2 |
| Eif2s3x | Brix1 |
| Srp54 | Otub2 |
| Rabggta | Ccdc22 |
| Cct6a | Atp5j2 |
| Ppfia3 | Mex3d |
| Cops4 | Taf9 |
| Dst | Vstm2l |
| Cadm1 | Nek9 |
| Trnt1 | Slc7a4 |
| Plxna1 | Kcnc2 |
| Acbd6 | Wdr62 |
| Ttc7b | Setd1a |
| Ddx3x | Zc3hc1 |
| Uqcrb | Crabp1 |
| Flii | Dvl3 |
| Pls3 | Prkd1 |
| Lrfn4 | Snx25 |
| Slc12a5 | Gemin5 |
| Begain | Ddx51 |
| Ykt6 | Aars2 |
| Dlgap4 | Nrbf2 |

| | |
|---|---|
| Scn1a | Nudt4 |
| Sh3glb1 | Fam103a1;Gm10767 |
| Cacna1b | Znf800;Zfp800 |
| Mthfd1l | Polr3f |
| Gprin3 | Smarca4 |
| Gak | Cntf |
| Psmc6 | Mcf2l |
| Wfs1 | Palm2Akap2;Akap2 |
| Ndufs7 | Lcmt2 |
| Coq6 | Mcm4 |
| Ndufa9 | Col1a1 |
| Pag1 | Dynlrb2 |
| Mrps27 | Sec11c |
| Sort1 | Glipr2 |
| Tanc2 | Ncf1 |
| Shmt2 | Tceb2 |
| Syt17 | P4htm |
| Ttc9b | Snx19 |
| Lrpprc | Asns |
| Trim3 | Ttbk1 |
| Dcun1d3 | Ppfia1 |
| Mapre3 | Kiaa0907 |
| Ahcy | Pde4dip |
| Ndufs2 | Atp5o |
| Fahd2 | Rpl31 |
| Akap5 | Uhrf2 |
| Grin1 | Zyx |
| Gpx4 | Nudt21 |
| Rptor | Rgs10 |
| Usp9x | Exosc8 |
| Timm9 | Casd1 |
| Ubxn6 | Atp5i |
| Katnb1 | Klhdc4 |
| Cdh2 | Ncoa7 |
| Vdac2 | Rpl15;Gm10020 |
| Chmp4b | Rab9a;Rab9 |
| Gmppb | Gmeb1 |
| Eif6 | Sat2 |
| Ddt | Pnisr |
| Cacnb4 | Sars |
| Bphl | Fbxl20 |
| Ggt7 | Fam21 |
| Pygb | Kank1 |

| | |
|---|---|
| Chrm4 | Kcnn2 |
| Nfs1 | Man1b1 |
| Dnaja3 | Scn2a1 |
| Aldh1l2 | Hnrnpul1 |
| Arhgap21 | Kif3b |
| Epdr1 | Hcfc1 |
| Cct7 | Unc119 |
| Copb1 | Faim |
| Slc9a7 | Bend6 |
| Dmwd | Klhl4 |
| Vat1 | Tbcc |
| Pik3r1 | Rps24 |
| Scamp5 | Mrpl20 |
| Acot1 | Plg |
| Nisch | Morc3 |
| Galc | Garnl3 |
| Me3 | Aldh1a2 |
| Rab15 | Spg11 |
| Plxnb1 | Magoh;Magohb |
| Nipsnap3b | Sema4b |
| Lrrtm2 | Smad3 |
| Scn2b | Hnrnpr |
| Rhoa | Lpl |
| Ryr2 | Zc3hav1 |
| Mlst8 | Vrk1 |
| Gps1 | Phf8 |
| Prrt2 | Gpt |
| Calb1 | Slitrk5 |
| Pgm2l1 | Lum |
| GOLGA7B | Chrm3 |
| Adh5 | Serpine2 |
| Agpat4 | Naa16 |
| Tubb4a | Ncor1 |
| Mff | Tnfrsf21 |
| Tomm20 | Trak1 |
| Mtor | Ptrhd1 |
| Glul | Acsm4 |
| L2hgdh | Fam129b |
| Epb41l3 | Mtfmt |
| Psma3 | Itgb5 |
| Vps29 | Scube1 |
| Clip1 | Ccm2 |

| | |
|---|---|
| Gspt2 | Rac2 |
| Tmem63b | Adcy2 |
| Sncg | St3gal4 |
| Vamp7 | Nucb2 |
| Pgm3 | Gng12 |
| Syt3 | Cpeb3 |
| Carm1 | Gm20498 |
| Gsr | Kctd5 |
| Wipf3 | Fam134c |
| Sult4a1 | Klhdc2 |
| Gas7 | Fbxw9 |
| Itgb8 | Srsf1 |
| Gprin1 | Xrn2 |
| Xpo7 | Megf9 |
| Git2 | Dtx3 |
| Ndufb10 | Tfdp1 |
| Aldh1b1 | Hp |
| Tjp1 | Rpa2 |
| Rgs9 | Fbln2 |
| Ndufb4 | Nop58 |
| Pdhx | Xpo4 |
| Mapk8 | Usp35 |
| Grin2a | Tia1 |
| Nt5e | Pbrm1 |
| Fam136a | Sap30 |
| Stk32c | Gatad2a |
| Akr1b7 | Chmp5 |
| Tmem143 | Clstn3 |
| Synj2bp | Helz |
| Psmb6 | Med29 |
| Rimbp2 | Limd1 |
| Map4 | Lsm4 |
| Sfxn1 | Prps2 |
| Bin1 | Nsun5 |
| Rap2b | Tcp11l2 |
| Srgap2 | Anapc7 |
| Arhgdia | Tcof1 |
| Slc6a11 | Cbln3 |
| Napg | Yjefn3 |
| Spr | Nwd1 |
| Paics | Edc3 |
| Rtn1 | Supv3l1 |

| | |
|---|---|
| Dgke | Cnot7 |
| Slc30a9 | Spns1 |
| Adprh | Pggt1b |
| Itgam | 15-Sep |
| Coa3 | 9930111J21Rik2 |
| Myl12b | Zfp871 |
| Gabra3 | Pycrl |
| Acp6 | Arsb |
| Prmt8 | Sf3b2 |
| Ap1m2 | Slc39a6 |
| Arf5 | Gorasp1 |
| Prkcg | Kcnh7 |
| Dnm3 | Ddx17 |
| Acat1 | Abca9 |
| Coa7 | Fuk |
| Tpi1 | Ddn |
| Ap2s1 | Gla |
| Cnksr2 | Supt5h |
| Pkm | Mast3 |
| Arhgef12 | Rint1 |
| Rgs20 | Wars |
| Pdia4 | Mmgt1 |
| Ttc1 | Ube2q1 |
| Fbxo3 | Leng8 |
| Elmod1 | Slc5a3 |
| Psmd6 | Anapc1 |
| Rnf141 | Nsrp1 |
| Fbxo41 | Itga7 |
| Slc44a1 | Ppp1r8 |
| Osbpl2 | Gng5 |
| Mfn2 | Lims1 |
| Ptges3 | Rcor1 |
| Tmx1 | Arsk |
| Alg2 | 5031439G07Rik |
| Pgam1 | Cdk12 |
| Chmp1a | Tbc1d23 |
| Maob | Pid1 |
| Slmap | Kif19;Kif19a |
| Wbp2 | Pcdhgc5 |
| Pex5l | Cep192 |
| Negr1 | Slu7 |
| Sri | Stfa3 |
| Tpbg | Adat3 |

| | |
|---|---|
| Uchl5 | Smg7 |
| Itpka | Malt1 |
| Setd7 | Dhcr24 |
| Timm13 | Phldb1 |
| Ca2 | Anp32a |
| Sec23a | Aagab |
| Taok1 | Runx1t1 |
| Wdr48 | Kpna4 |
| Atp1a3 | Slc25a45 |
| Tkt | Stat3 |
| Uso1 | Lhpp |
| Pdpk1 | Atp5a1 |
| Vps13a | Pik3r2 |
| Akr7a2 | Nat9 |
| Acaca | Enc1 |
| Psmd13 | Eif3d |
| Ccny | Camk2n2 |
| Cisd1 | Enpp2 |
| Vapa | Mmp17 |
| Cdc42 | Znf143 |
| Slc25a3 | Ptpn3 |
| Ctbp1 | Cdc42se2 |
| Gap43 | Aqr |
| Appl1 | Ankfy1 |
| Clcn3 | N4bp1 |
| Cpne3 | Bbs1 |
| Ndrg3 | Mcm3ap |
| Dip2b | ORF11;0610007P14Rik |
| Lrpap1 | Smek2 |
| Chkb | Maged2 |
| Gba | Pih1d1 |
| Cryzl1 | Pde3a |
| Mat2a | Fdx1l |
| Clic1 | Dpf2 |
| Lmna | Fgd3 |
| Pak2 | Mrpl51 |
| Sgtb | Nav1 |
| Capn2 | Arih2 |
| Ywhab | Morn4 |
| Timm50 | Commd4 |
| Map2k6 | Bcar1 |
| Cyfip1 | Fbxo31 |
| Sec31a | Cd93 |

| | |
|---|---|
| Eif4e | Cdh7 |
| Madd | Maea |
| Cacna2d2 | Supt16;Supt16h |
| Sdhb | Cul9 |
| Selenbp1 | Syt13 |
| Osbp | Gtf3c5 |
| Pabpc1 | Spata2 |
| Gnl1 | Fam84b |
| Nagk | Pik3c2a |
| Bcas3 | Cyp2j9 |
| Ssr1 | Tatdn1 |
| Pacs1 | Wdr65 |
| Inpp5a | Usp20 |
| Dgki | Islr |
| Iqsec3 | Supt7l |
| Ttpal | Nup50 |
| Unc13c | Rps6 |
| Mmab | Nova1 |
| Prkar2b | Dysf |
| Pde10a | Cyp4x1 |
| Rab3c | Hat1 |
| Dld | Zfyve26 |
| Bcr | Daglb |
| Nit1 | Lmnb2 |
| Plscr3 | Nyap1 |
| Tab1 | Cdc27 |
| Slc1a6 | Atg13 |
| Lrrc57 | Nsg1 |
| Camk2b | Mettl9 |
| Islr2 | Krt84 |
| Tagln2 | Irf5 |
| Grm2 | Trim56 |
| Bmpr2 | Plcxd2 |
| Atad3 | Uqcr11 |
| Pafah1b3 | Dus2 |
| Ppa1 | Cbr2 |
| Pdhb | Rbm3 |
| Ndufb8 | Camsap1 |
| Synrg | Mrpl41;Gm6434 |
| Hpca | Ppm1l |
| Acaa1a | Hdac3 |
| Uqcrfs1 | Gyg1;Gyg |
| Psmc2 | Qsox2 |

| | |
|---|---|
| Gabarapl 2 | Rtkn |
| Mdh2 | Ppp1r12b |
| Ntng1 | Plxdc2 |
| Abcb9 | Ddx39a |
| Pls1 | Clpb |
| Cops6 | Trip11 |
| Atp9a | Klhl2 |
| Crkl | Tubb6 |
| Nol3 | Chdh |
| Rpsa | Tlr2 |
| Ap2a2 | Actr5 |
| Ptcd3 | Dnph1 |
| Cspg5 | Ing4 |
| Gk | Pcdha9 |
| Ogdh | Vps41 |
| Reps1 | Zfyve19 |
| Lgi1 | Trmt61a |
| Pcyox1 | Chd6 |
| Anks1b | Map1lc3b |
| Itgav | Metap1 |
| Araf | Akr1c14 |
| Col1a2 | Tap2 |
| Pdcd6ip | Chd3 |
| Slc4a3 | Nudt8 |
| Tmx3 | Rictor |
| Efr3b | Ints5 |
| Agap1 | Polr2i |
| Nsf | Adam17 |
| Cpm | Pcbd1 |
| Tm9sf2 | Pxdn |
| Prkar1a | Pigt |
| Synj1 | Ust |
| Aco1 | Tmem63a |
| Snca | Il6st |
| Taldo1 | Slc25a29 |
| Hspa4 | Eftud2 |
| Gstm5 | Rrp1b |
| Slc22a23 | Vmp1 |
| Rab23 | Polr2h |
| Aldoa | Pop1 |
| Tom1 | Ppp4c |
| Pgls | 2900011O08Rik |
| Actr10 | C3 |

| | |
|---|---|
| Exog | Itpkb |
| Akap7 | Krt222 |
| Myo18a | Fam110b |
| Cntnap2 | Rgs17 |
| Vat1l | Klc3 |
| Npepps | Pdcl |
| Wdr37 | Fat1 |
| Ccdc136 | Ube2e3 |
| Myadm | Gigyf2 |
| Hccs | Dab2 |
| Arhgef6 | Pdf |
| Ank2 | Calu |
| Anxa4 | Lmf2 |
| Plcg1 | Rhot2;Gm20683 |
| Ap3m2 | Ptprg |
| Sec22b | Phip |
| Prkcd | Poldip3 |
| Hip1 | Plce1 |
| Gnaz | Zeb2 |
| Ldha | Klc4 |
| Cul1 | Eif2a |
| Eif4a1 | Clcn7 |
| Ppp1r9b | Gpx7 |
| Ywhag | Gpam |
| Nefl | Golim4 |
| Hsd17b12 | Trmt1 |
| Nif3l1 | Trim30a |
| Syt7 | Stk4 |
| Gnb4 | Prc1 |
| Src | Yipf5 |
| Slc25a46 | Taf4a |
| Tbcd | Mta1 |
| Vwa8 | Gab1 |
| Trappc9 | Camlg |
| Me1 | Rgs12 |
| Neo1 | Ca4;Car4 |
| Crk | Coil |
| Atp6v0d1 | Gpr50 |
| Sgip1 | Aaas |
| Exoc4 | Carns1 |
| Plec | Top3b |
| Hdhd2 | Qpct |
| Ndufs5 | Prpf40a |

| | |
|---|---|
| Dynll2 | Slc13a3 |
| Sigmar1 | Man2b1 |
| Prkaca | Ehhadh |
| Nudt3 | Fam105a |
| Sptb | Mrpl4 |
| Pgrmc1 | Ggt1 |
| Reep5 | Dpm1 |
| Gpc4 | Gtf3c2 |
| Pfkm | Rnf170 |
| Pitpnb | BC005561 |
| Nfasc | Tead1 |
| Kpna3 | Rpl30 |
| Mbp | Mtm1 |
| Ckmt1 | Prpf6 |
| Stk11 | Rbm19 |
| Gripap1 | Phka1 |
| Nadk2 | Smn1 |
| Cyb5b | Naa20 |
| Wdfy3 | Ypel5 |
| Cmpk2 | Ccpg1 |
| Nlgn3 | Ergic3 |
| Ppp1r7 | Abca3 |
| Mtnd1 | Ntsr1 |
| Rgs7 | Rps6kb2 |
| Fh | Cisd2 |
| Ptk2b | Chpf2 |
| Eps15 | Ube4a |
| Strn | Stx2 |
| Pgp | Cmc2 |
| Hspa1a | Fyb |
| Lrrc8b | Sardh |
| Nipsnap1 | Porcn |
| Vps16 | Pnn |
| Ptprj | Fkbp2 |
| Ppt1 | Trappc6b |
| Samm50 | Bfar |
| Sfxn3 | Polr1e |
| Map2k4 | Nup133 |
| Nlgn1 | Tbl2 |
| Gnai1 | Ap4s1 |
| Ranbp9 | Mrpl37 |
| Ndufb9 | 2610301B20Rik |
| Zadh2 | Mocos |

| | |
|---|---|
| Bpnt1 | Cd82 |
| Ptpra | Serpina3g |
| Slc23a2 | Erap1 |
| Pcmt1 | Camk1 |
| Cpne1 | Apob |
| Kif5b | Brat1 |
| Add2 | Commd8 |
| Sfn | Pak6 |
| Vps36 | St7 |
| Prdx1 | Dhx32 |
| Tcp11l1 | Dcx |
| Dnm1 | Wdr17 |
| Ptprr | Tdrd3 |
| Pycr2 | Gtf2h1 |
| Arfgef2 | Zbp1 |
| Rph3a | Msh3 |
| Anxa11 | Arhgap31 |
| Arf3 | Dner |
| St13 | Stard13 |
| Flot1 | Abhd11 |
| Slc25a14 | Fam184b |
| Cse1l | Nfix |
| Bnip3 | Fbxl18 |
| Ppp1r12a | Mrps17 |
| Pdk1 | Ctss |
| Chl1 | Afap1l2 |
| Sec14l2 | Pcmtd2 |
| Atp6v0a2 | Itgb1bp1 |
| Slc17a7 | Sorcs1 |
| Lonp1 | Mrps6 |
| Hspa2 | Srbd1 |
| Prune2 | Ctsz |
| Vamp3 | Rela |
| Hadha | Pla2g4a |
| Neto1 | Mrpl44 |
| Gsg1l | Zfp185;Znf185 |
| Mecr | Ehbp1l1 |
| Rbbp9 | Dcun1d5 |
| Gna13 | Flrt2 |
| Vta1 | Etl4;Skt |
| Nucb1 | Slc25a19 |
| Vti1a | B2m |
| Clasp2 | Cox6a1 |

| | |
|---|---|
| Arhgap26 | Selo |
| Park7 | Atp8a2 |
| Abcb10 | Sash1 |
| Wasf1 | Setd2 |
| Bcan | Cx3cl1 |
| Map1a | Cstf2 |
| Prkcb | Prrc2b |
| Hebp1 | Chrna4 |
| Slc4a10 | Ncapd3 |
| Ppp2cb | Smpdl3a |
| Phb | Rpl5 |
| Ppp1r1b | Nfia |
| Sirt2 | Gpc2 |
| Psma1 | Rlbp1 |
| Jagn1 | Slco1c1 |
| Coasy | Echdc2 |
| Slc18a2 | Pianp |
| Oat | Vamp8 |
| Apbb1 | Actg1 |
| Ncam1 | Ppt2 |
| Cttnbp2 | Psen1 |
| Hmox2 | Nav2 |
| Pex14 | Cep68 |
| Myh14 | Tmub1 |
| Gfod1 | Gemin4 |
| Rmdn3 | Ptp4a3 |
| Chp1 | Hcfc2 |
| Vps45 | Poc1a |
| Esd | Acan |
| Tpm1 | Gosr2 |
| Dlgap1 | Tep1 |
| Gabbr1 | Rrp1 |
| Unc5a | Sirt3 |
| Snx5 | Lrp4 |
| Ehd4 | Mctp1 |
| Snap25 | Col4a5 |
| Kcnab1 | Ptpro |
| Arpc4 | Cuedc1 |
| Csnk2a2 | Nup214 |
| Nrxn1 | Aarsd1 |
| Sptbn1 | Ttbk2 |
| Nptn | Haus6 |
| Slc1a4 | Hexdc |

| | |
|---|---|
| Armc1 | Edil3 |
| Glrx | Pfdn4 |
| Praf2 | Rps6ka4 |
| Slc6a7 | Prrc1 |
| Plxna3 | Krt86 |
| Chchd3 | Kif1c |
| Gnao1 | Arhgap9 |
| Nampt | Cep97 |
| Ccz1 | Zcrb1 |
| Stx7 | Cyth4 |
| Oxct1 | Mest |
| Sorbs1 | Rab39a |
| Ube2v1 | Pygl |
| Gapvd1 | Bpgm |
| Adsl | Snx12 |
| F3 | Nedd1 |
| Ap3b2 | Hcn3 |
| Taok3 | Fam171b |
| Psmd4 | Csnk1g3 |
| Erc2 | Ccdc101 |
| Coro1c | Pik3ca |
| Ggps1 | Phf2 |
| Sccpdh | Pold3 |
| Bag6 | Cetn2 |
| Kcna1 | Ascc3 |
| Pin1 | Phkg1 |
| Fmnl3 | Lsm7 |
| Dock9 | Lama5 |
| Nt5c3a | Arhgef17 |
| Kazn | Xrcc6bp1 |
| Numbl | Apobec1 |
| Slc16a1 | Coro7 |
| Ruvbl1 | Krtap13-1 |
| Dnajc6 | Phc3 |
| Rab21 | Gpt2 |
| Ipcef1 | Akr1b10 |
| Tmem43 | C1qtnf5 |
| Prkag1 | Eral1 |
| Sphk2 | Ankh |
| Syt12 | Ano6 |
| Lamp1 | Nf2 |
| Nckipsd | 2210016F16Rik |
| Ntm | Med19 |

| | |
|---|---|
| Dnaja1 | Spag1 |
| Ganab | Fam102b |
| Slc29a1 | Rab3ip |
| Fdps | U2af2 |
| Rp2 | Sdpr |
| Rheb | Os9 |
| Tph2 | Mpped2 |
| Abi2 | Myrf |
| Dmxl1 | Gramd1a |
| Ndufa5 | Mycbp2 |
| Lingo1 | Tmem132e |
| Nt5dc3 | Parva |
| Shroom2 | Fdft1 |
| Gldc | Themis2 |
| Mcu | Cbx3 |
| Gphn | Psmb8 |
| Mrps22 | Irf2bp1 |
| Tmed10 | Drap1 |
| Tbc1d24 | Timm8b |
| Olfm3 | Sema6b |
| Faf2 | Xkr7 |
| Dcc | Acap3 |
| Rhob | Cc2d1b |
| Atp1a1 | Ago1 |
| Impdh2 | Bola3 |
| Immt | Fhl2 |
| Slc30a1 | Mrpl28 |
| Lypla1 | Kank4 |
| Cct2 | Yy1 |
| Idh3g | Zc3h4 |
| Cox4i1 | Rpl37a |
| Chchd6 | Snrpg |
| Calb2 | Fbxo30 |
| Cds2 | Mrps14 |
| Txnrd2 | Fam76b |
| Cdh11 | Srsf11 |
| Rab12 | Nrd1 |
| Pacsin2 | G6pc3 |
| Idi1 | Rpl34 |
| Clint1 | Kng1 |
| Paip1 | Gatm |
| Mdp1 | Trim28 |
| Dcaf7 | Smpx |

| | |
|---|---|
| Scn4b | S100a16 |
| Doc2a | Dgcr14 |
| Unc13a | Gas6 |
| Pip4k2b | Dcaf8 |
| Ywhae | Drg1 |
| Dpp6 | Dhx58 |
| Arc | Tfdp2 |
| Nudc | 2010300C02Rik |
| Ndufaf2 | Golga2 |
| Crybb1 | Des |
| Rock2 | Slc35g2 |
| Fech | Lrfn5 |
| Tpm3 | Kif4 |
| Eif2s1 | Serpina1c |
| Plcl1 | Inpp5b |
| Wasl | Eml3 |
| Akt3 | Ids |
| Apool | Bdnf |
| Eef1a1 | Smndc1 |
| Hnrnph1 | Prkg1 |
| Gria1 | Arx |
| Psmc4 | Znf22 |
| Vapb | Ctu1 |
| Coro2b | Aven |
| Hprt1 | Rbfox3 |
| Nrxn3 | Soat1 |
| Atic | Mcm6 |
| Cdk16 | Rai1 |
| Ppid | Pet112 |
| Atp1b1 | Cutc |
| Vps18 | Plekho1 |
| Vps11 | Sp1 |
| Vcp | Foxred1 |
| Anxa7 | Lrch3 |
| Fasn | Rsrc1 |
| Gpd1 | Kdm5a |
| Sorcs2 | Spef2 |
| Mgst3 | Exosc4 |
| Grm8 | Ap5z1 |
| Pgs1 | Myof |
| Uchl3 | Nsmf |
| Stmn3 | Cdh5 |
| Sh2d5 | Cradd |

| | |
|---|---|
| Scarb2 | Npc1 |
| Mtmr9 | Pxn |
| Cd81 | Fat3 |
| Dync1li2 | Bre |
| Pip5k1a | Lrrc8c |
| Nudt5 | Med27 |
| Iqsec2 | Prcp |
| Gdi1 | Spta1 |
| Tsr2 | Tfap2a |
| Atp2a2 | Pdzd8 |
| Dctn2 | Cth |
| Dsp | Sqstm1 |
| Capn5 | C1qb |
| Slc8a2 | Loh12cr1 |
| Cnrip1 | Tcea1 |
| Cacybp | Reep2 |
| Sirpa | Pacsin3 |
| Gsk3a | Jak1 |
| Gabra5 | Kcnh2 |
| Rab8b | Obfc1 |
| Ppp3ca | Tbc1d8 |
| Phactr1 | Nip7 |
| Aldoc | Hnrnpa3 |
| Calcoco1 | Acss3 |
| Tmem13<br>2a | 05-Mar |
| Dpysl5 | Csl |
| Ankrd63 | Colgalt1 |
| Vps33a | Ankrd52 |
| Pptc7 | Srxn1 |
| Aak1 | Mobp |
| Ehd3 | Sf3a1 |
| Aplp2 | Usp54 |
| Slc10a4 | Tspyl4 |
| Camk2d | Nenf |
| Icam5 | Sf1 |
| Acad8 | Pdcd4 |
| Pdk2 | Zmiz1 |
| Tln1 | Prkdc |
| Dlg2 | Usp33 |
| Syn2 | Hsd17b8;H2-Ke6 |
| Dtymk | Hdlbp |
| Grm1 | Oxnad1 |
| Rmnd5a | Snx14 |

| | |
|---|---|
| Syt5 | Slc25a32 |
| Pfkp | Rbms3 |
| Ppfia2 | Rars |
| Gpm6a | Cbln4 |
| Mpp6 | Prkx |
| Rcan1 | Fli1 |
| Eci1 | Hacl1 |
| Eif3l | Top2b |
| Tgm1 | Rc3h1 |
| Rab30 | Unc79 |
| Wnk2 | Lbh |
| Snx27 | Gpkow |
| Pacs2 | Alg5 |
| Ncan | Nrp2 |
| Atp6v1f | Pcdh7 |
| Gabrb1 | Ccnt1 |
| Slc25a5 | Fam168a |
| Actn1 | Akna |
| Mink1 | Slc39a12 |
| Abat | Morf4l2 |
| Dlgap2 | Azi2 |
| Rims1 | Csad |
| Rpl13 | Pdap1 |
| Strn3 | Tmem2 |
| Slc25a10 | Kcnb1 |
| G6pdx | Cox7a2l |
| Chn1 | Acy1 |
| Sh3gl2 | Glyctk |
| C1ql3 | Nr3c1 |
| Rad23b | Lclat1 |
| Psmb7 | Yars2 |
| Hsdl2 | Ercc5 |
| Slc1a3 | Kpna2 |
| Sv2b | Impad1 |
| Hsp90ab1 | Hist1h2af;Hist1h2ab;Hist1h2ak;Hist1h2ah |
| Ephx1 | Man2a2 |
| Slc9a1 | Dcps |
| Fmnl1 | Ttll12 |
| Chat | Col6a2 |
| Stat1 | Ormdl2 |
| Dagla | Anapc2 |
| Rab6b | 02-Mar |
| Vps4a | Mphosph8 |

| | |
|---|---|
| Nedd4 | Cul7 |
| Rps5 | Cebpzos |
| Sik3 | Znf24 |
| Cbr3 | Rpusd2 |
| Nbea | Myo9a |
| Rdx | Fbrsl1 |
| Syngr1 | Phykpl |
| Rab27b | Ptgs1 |
| Blmh | Plekhb1 |
| Slc24a4 | Zic2 |
| Cntn1 | Exosc2 |
| Mark1 | Trpm7 |
| Aldh1a1 | Wdr20 |
| Rpn2 | Kras |
| Eif5a | Ccdc85b |
| Traf3 | Atf2 |
| Slc1a2 | Cwf19l2 |
| Hcn2 | Elp6 |
| Mag | Nsd1 |
| Rogdi | Eif4ebp2 |
| Cacng8 | Taco1 |
| Mapre1 | Vars |
| Ttyh3 | Rnmt |
| Uqcr10 | Sec24c |
| Tenm1 | Mkrn1 |
| Cpt2 | Gtpbp6 |
| Vps35 | Mapk14 |
| Cfl2 | Prdm2 |
| Csmd1 | Tbc1d8b |
| Sgsm1 | Exosc9 |
| Pip5k1c | Ppic |
| Suclg2 | Nell2 |
| Epn2 | Syf2 |
| Timm23 | Nrn1l |
| Aifm3 | Pcp2 |
| Ppox | Gfm2 |
| Myh9 | Pkn2 |
| Gde1 | Ptpn13 |
| Ppp6c | Arfgap3 |
| Ccdc51 | Znf148 |
| Lap3 | Fam134b |
| Acyp1 | Cfdp1 |
| Psmd14 | Txlng |

| | |
|---|---|
| Rps6ka1 | Mto1 |
| Stoml2 | St3gal6 |
| Cpne8 | Podxl |
| Apba1 | Chuk |
| Nckap1 | Cyp2a5 |
| Grb2 | Hnrnpll |
| Mpc2 | Rhoq |
| Smcr8 | Rgs7bp |
| Tsn | Prkrir |
| Kpnb1 | Snrpd3 |
| Btbd17 | Fig4 |
| Ahsa1 | Arfrp1 |
| Rab39b | Rpf2 |
| Atp6v1h | Itga5 |
| Mapk1 | Eif4g1 |
| Amph | Ankhd1 |
| Nlgn4l | Dhx8 |
| Aldh3a2 | Aldh3b1 |
| Nck2 | Fez1 |
| Nfu1 | Pstpip1 |
| Hspa9 | Arhgap12 |
| Tpd52 | Nudt14 |
| Ccdc177 | Fntb |
| Ipo4 | Cpsf1 |
| Cdk14 | Vps8 |
| Il1rapl1 | Sec23b |
| Psma7 | Epb4.1l4b;Epb41l4b |
| Ppp6r1 | Bod1l |
| Atp6v1g1 | Sema6d |
| Sod1 | Jakmip3 |
| Ppp1ca | Map3k7 |
| Tmem9b | Usp34 |
| Rab5a | Smarcc1 |
| Pura | Slmo1 |
| Flna | Nek1 |
| Tenm3 | Supt6h |
| Mccc2 | Fkbpl |
| Plxnc1 | Serpina1a |
| Msra | Akt2 |
| Flrt3 | Plcb2 |
| Pitpna | Eno3 |
| Arhgap39 | Celf2 |
| Echdc1 | Rbm38 |

| | |
|---|---|
| Akap12 | Pus7 |
| Rps6ka3 | Mrpl17 |
| Dync1h1 | Uba3 |
| Mtfp1 | Ildr2 |
| Plaa | Ctnna3 |
| Abhd10 | Lrrn1 |
| Prnp | Bnip1 |
| Ctsd | Cxx1b |
| Cox6b1 | Smarca5 |
| Usp7 | Msto1 |
| Acat2 | Pla2g6 |
| Trim32 | Rnf40 |
| Scfd1 | Mon2 |
| Rap1gap2 | Csf1r |
| Cd47 | C530008M17Rik;Kiaa1211 |
| Ckap4 | Tex2 |
| Slc18a3 | Mvp |
| Epha4 | Cpsf2 |
| Smpdl3b | Cog4 |
| Clcn6 | Ero1l |
| Cat | Kat8 |
| Get4 | Ank1 |
| Slc25a4 | Slc4a1 |
| Psmb2 | Hddc3 |
| Fabp3 | Ube3b |
| Csrp1 | Slc5a6 |
| Rapgef4 | Rnf11 |
| Rala | Hist1h2bc;Hist1h2bh |
| Cryab | Ninj1 |
| Ptges2 | Golt1b |
| Myl6 | Tmed3 |
| Hint1 | Kifc5b |
| Cltc | Akt1s1 |
| Padi2 | Igsf1 |
| Tpd52l2 | Osbpl3 |
| Ide | Tmem41b |
| Srm | Rbm26 |
| Ube2n | Rae1 |
| Nsfl1c | Glb1l |
| Ahcyl1 | Abcb1a |
| Impa1 | Aspscr1 |
| Farp1 | Pcf11 |
| Pitpnc1 | Tshz1 |

| | |
|---|---|
| Sfxn5 | Ighm |
| Adk | Mocs1 |
| Ncln | Rpl23 |
| Mtpn | Diexf |
| Sptan1 | Ift46 |
| Aldh5a1 | Mcur1 |
| Tpt1 | Dennd4a |
| Acadm | Cspg4 |
| Cyp2s1 | Mknk1 |
| Rab3gap2 | Pgm1;Pgm2 |
| Srpk2 | Atg4c |
| Camkv | Dkk3 |
| Lancl2 | Sdf2l1 |
| Wdr45 | Dym |
| Dnpep | Cep131 |
| Eps15l1 | Chst2 |
| Prpsap2 | Fubp3 |
| Arf1 | Wiz |
| Hadh | Pwp1 |
| Atp6v1d | Hpx |
| Pcbp2 | Dnajc8 |
| Pepd | Thtpa |
| Stx4 | Erbb4 |
| Acadvl | Sphkap |
| Rftn2 | Krtap15-1 |
| Arl1 | Smg9 |
| Uqcrc2 | Rpap1 |
| Tmx4 | Utp11l |
| Atp2b1 | Sepn1 |
| Dynll1 | Brd4 |
| Rad23a | Tnip1 |
| Rtn4 | Slc27a2 |
| Cep170 | Ythdc2 |
| Fhl1 | Mipep |
| Afg3l1 | Nolc1 |
| Capza2 | Serpina3m |
| Hars2 | Tmem14c |
| Sacm1l | Lifr |
| Gfm1 | Rrs1 |
| Lysmd1 | Atad2 |
| Marcks | Thsd7a |
| Pmpcb | Zcchc8 |
| Scn3b | Cdc34 |

| | |
|---|---|
| Atp1b3 | Kifc3 |
| Strip2 | Nol8 |
| Mapk8ip3 | Map6d1 |
| Psme1 | Pllp |
| Ndufa7 | Vav1 |
| Opa3 | Arhgap17 |
| Actn2 | Dock2 |
| Dync1i2 | Orm1 |
| Oplah | Abca5 |
| Cap1 | Pcp4l1 |
| Stub1 | Saal1 |
| Plcb1 | Utp18 |
| Rab5c | Tarsl2 |
| Ppp3r1 | Msi2 |
| Prodh | Dnajc1 |
| Rabep1 | Dos |
| Tuba4a | Bloc1s5 |
| Gnai3 | Cd2bp2 |
| Emc2 | Zcchc11 |
| Prkar1b | Naa10 |
| Acot11 | Rhpn2 |
| Daam1 | Ifi47 |
| Dtnb | Higd2a |
| Rab10 | Zbed5 |
| Wdr77 | Rps27l |
| Idh3a | Eif3f |
| Dnajb2 | Sgsm2 |
| Slc30a3 | Lrrc16b |
| Cct4 | Ifngr2 |
| Glrx5 | Khdrbs1 |
| Timmdc1 | Fetub |
| Pten | Gadd45gip1 |
| Ndufv1 | Ikbip |
| Cs | Kcnab3 |
| Slc25a23 | Dohh |
| Lin7c | Las1l |
| Acot7 | Mgea5 |
| Klc2 | Mpp7 |
| Mtch1 | AI607873 |
| Adcy6 | Smc2 |
| Rac1 | Gabra6 |
| Thy1 | Slc8a3 |
| Arl2 | Tmod3 |

| | |
|---|---|
| Osbpl10 | Trappc10 |
| Hibadh | Ephb3 |
| Psmb4 | Lyn |
| Tfrc | Noc2l |
| L1cam | Sdf2 |
| Cops2 | Eif5b |
| Kcnj4 | Lyrm9 |
| Psmd1 | Gfpt1 |
| Dlg4 | Pum1 |
| Plcd1 | Lppr1 |
| Psmd12 | Mgat5 |
| Cnp | Cast |
| Tmem126a | Med8 |
| Dynlrb1 | Tsc22d3 |
| Cul3 | Kdm6a |
| Gstm1 | 2010005H15Rik |
| Vps52 | Lrrc49 |
| Lsamp | Macrod1 |
| Glg1 | Obp1b;Gm14743 |
| Phyhip | Cd9 |
| Snx6 | Rilp |
| Gdap1 | Rnd3 |
| Rcn1 | Slc15a4 |
| Aldh6a1 | Dixdc1 |
| Cadm3 | Trim14 |
| Eif5 | Kdm4c |
| Prdx6 | Btk |
| Gabrb3 | Adprhl2 |
| Nans | Krr1 |
| Csnk1a1 | Gm996 |
| Becn1 | Aldh16a1 |
| Eif3e | Ifi204 |
| Plch2 | Pigk |
| Stmn1 | Wdfy4 |
| Olfm1 | Ckm |
| Dusp3 | S100a4 |
| Zfyve1 | Bloc1s1 |
| Fam162a | Creg2 |
| Atg7 | Gk5 |
| Uchl1 | Mettl7a1 |
| Rgs14 | Btf3 |
| Ak4 | D430041D05Rik |
| Exoc6b | Fam20b |

| | |
|---|---|
| Sdha | Tbr1 |
| Rin1 | Pmvk |
| Slc25a51 | Plch1 |
| Lgals3 | Sec14l4 |
| Tnik | Cbx6 |
| Hspe1 | Casc3 |
| Htra2 | Pnpla6 |
| Dpysl3 | Rims2 |
| Gdpd5 | Mad2l1 |
| Evl | Eif3k |
| Decr1 | 1810043G02Rik |
| Cdc42bpa | Zhx2 |
| Pdxk | Phpt1 |
| Dnm1l | Galnt2 |
| Acsl5 | Hmgcs2 |
| Myh10 | Ssh3 |
| Tubb2a | Copz2 |
| Acly | Znf592 |
| Cpne4 | Zfp219 |
| Prkce | Pxmp2 |
| Lpgat1 | Sepp1 |
| Osbpl8 | Siglec1 |
| Slc7a8 | Fam63b |
| Gnb2 | Arid1a |
| Fam131b | Aar2 |
| Csmd3 | Aamp |
| Map1s | Ilk |
| Alad | Lyrm7 |
| Sdk2 | Med10 |
| Etf1 | Rbp4 |
| Sdhc | Wdr81 |
| Ppp2r1b | Slc2a6 |
| Trappc11 | Luc7l2 |
| Dhrs1 | Cmtm5 |
| Acsf2 | Zdhhc3 |
| Ttyh1 | Kcnd3 |
| Itgb1 | Dennd5b |
| Gls | Uhrf1bp1 |
| Clta | Elavl1 |
| Adck1 | Ldb1 |
| Dnaja4 | Rngtt |
| Nit2 | Kirrel |
| Fscn1 | Zfp462 |

| | |
|---|---|
| Cpne6 | Mrpl18 |
| Slc6a3 | Diap2;Diaph2 |
| Cab39 | Rpl23a |
| Ppp2r5a | Hrsp12 |
| Mark3 | Med12 |
| Vdac3 | Rab27a |
| Ppm1b | Cecr6 |
| Brsk2 | Als2 |
| Ddx3y | Cdc42ep1 |
| Ywhaz | Uba7 |
| Ddost | Arpin |
| Atad1 | Epb4.1 |
| Mtx2 | Rfc2 |
| Grm5 | Gltscr1 |
| Myl9 | Snrpe |
| Tubb5 | Dph6 |
| Fam114a2 | Zswim8 |
| Ralb | Rabl6 |
| Dtna | 2310061I04Rik |
| Slc25a12 | Sh3bp5l |
| Lamp5 | Esf1 |
| Twf1 | Samsn1 |
| Denr | Gnpnat1 |
| Kcnd2 | Lats1 |
| Gstz1 | Smarce1 |
| Skp1 | Slfn5 |
| Fam210a | Mkl2 |
| Smap2 | Insr |
| Add1 | Wdr35 |
| Eno2 | Plekhf1 |
| Nipa1 | Limd2 |
| Efhd2 | Stk3 |
| Slc5a7 | Srp72 |
| Cul5 | Ddx24 |
| Rragc | Snx9 |
| Iba57 | Chrac1 |
| Ubtd2 | Kct2 |
| Nwd2 | Itih2 |
| Gdap1l1 | Stradb |
| Slc9a3r1 | Srsf6 |
| Clvs1 | Rsph4a |
| Camkk1 | Zfml;Znf638 |
| Rap1b | Gpr108 |

| | |
|---|---|
| Atp8a1 | Ppp1r10 |
| Psmd8 | Mrps12 |
| Tbrg4 | Mrps2 |
| Mob4 | Slc1a1 |
| Sbf1 | Tor1aip2 |
| Slc7a14 | Prpf4 |
| Ola1 | Nid1 |
| Gda | Inf2 |
| Ddi2 | Ubr7 |
| Stx12 | Derl1 |
| Ndufb6 | Ankrd40 |
| Ttc9 | Plod2 |
| Exoc2 | Ubl3 |
| Ndufa10 | Plcg2 |
| Got2 | Ppap2b |
| Acap2 | Degs1 |
| Hapln1 | Zfand2b |
| Cox5b | Klhl8 |
| Ptprn | Mrpl11 |
| Rpl3 | Rpp38 |
| Pfn2 | mt-Co3 |
| Fis1 | Slc9a3r2 |
| Arf6 | Fus |
| Bsg | Rnf220 |
| Ptprf | Nupl2 |
| Mif | Micall1 |
| Stk39 | Nbeal1 |
| Itpr2 | Rpl14 |
| Arhgef9 | Hectd1 |
| Ndufa3 | Clcn5 |
| Bcs1l | Fam192a |
| Inpp1 | Mbd3 |
| Gpc1 | Pin4 |
| Faim2 | Rgs19 |
| Ptp4a2 | Tial1 |
| Stx3 | Csmd2 |
| Atp6v1b2 | Tmem35 |
| Ndufa12 | Eif3m |
| Lxn | Tor1a |
| Anxa5 | Wdr19 |
| Pygm | Mcmbp |
| Phb2 | Tnrc6c |
| Gria3 | Krt8 |

| | |
|---|---|
| Usp24 | Rchy1 |
| Psma5 | RBM8;Rbm8a |
| Ipo9 | Atp6v0d2 |
| Bsdc1 | Rbm12;Gm28036 |
| Iars2 | Rbm5 |
| Letm1 | Shmt1 |
| Echs1 | Ing3 |
| Slc27a4 | Ksr2 |
| Ppp6r3 | Hexim1 |
| Mcts1 | Fes |
| Hk1 | Tceal5 |
| Ptprd | Hmbs |
| Usp14 | Atp1a4 |
| Cdh13 | Chd8 |
| Lactb | Mrps28 |
| Scrn3 | Tbc1d10b |
| Fsd1l | Ppp1r17 |
| Enah | 6330403A02Rik |
| Syp | Tmem87a |
| Homer1 | Ttc8 |
| Bag5 | Adnp2 |
| Ap3s1 | Cars2 |
| Slc25a40 | Smpd1 |
| Cbr1 | Robo1 |
| Actr1b | Matn4 |
| Cotl1 | Peak1 |
| Ctsb | Rpl7l1 |
| | Irak1 |
| | Champ1 |
| | Fbxo6 |
| | Nras |
| | Plxdc1 |
| | Stk38 |
| | Tjp2 |
| | Trpc4 |
| | Ube2b |
| | Ccdc132 |
| | Rbbp4 |
| | Cand2 |
| | Rundc3b |
| | Dnajb5 |
| | Polr2f |
| | Nicn1 |

| |
|---|
| Gfra1 |
| Pom121 |
| Dok1 |
| Erf |
| Prpf19 |
| Ifi205b |
| Ints3 |
| Hnrnpk;Gm7964 |
| Tnks |
| Vars2 |
| Nos1ap |
| Abcc5 |
| Rgl3 |
| Wdr59 |
| Coprs |
| Erlec1 |
| Tapt1 |
| Bcl11b |
| Pcyt1b |
| Nup93 |
| Mark4 |
| Sik2 |
| Thbs1 |
| Stat6 |
| Ascc1 |
| Cox7a1 |
| Flcn |
| Cdc40 |
| Fam114a1 |
| Eif1a;Gm8300;Gm2016;Gm21936;Gm2035 |
| Fam210b |
| Zbtb43 |
| Gskip |
| Tle4 |
| Narfl |
| Rsu1 |
| Gcsh |
| Coa6 |
| Dnajc17 |
| Lsp1 |
| Slc9a9 |
| Tpm3-rs7 |
| Dync2h1 |

| |
|---|
| Zdhhc13 |
| Col4a3bp |
| Hopx |
| Ap4b1 |
| Bag4 |
| Noc4l |
| Wdr45b |
| Nucks1 |
| Itgb4 |
| Rbl2 |
| Amacr |
| Dcaf6 |
| Arhgef33 |
| Pja1 |
| Scrn2 |
| Rasa4 |
| Ifit3 |
| Senp7 |
| Cbx1 |
| Ddr1 |
| Rcbtb2 |
| Dph1 |
| Arid4a |
| Large |
| Tbc1d15 |
| Tns1 |
| Fkbp1b |
| Htra1 |
| Kank2 |
| Kxd1;Uba52 |
| Acad11 |
| Rbfox2 |
| Serpinb9b |
| Cd151 |
| Zwilch |
| Spin1 |
| Rps15a |
| Snrnp200 |
| Zfp407 |
| Dus3l |
| Usp19 |
| Suox |
| Mrps10 |

| |
|---|
| Eif2b1 |
| Arhgef28 |
| Frs2 |
| Mapk9 |
| Eomes |
| Cep250 |
| Ncf4 |
| Ctsl |
| Ipo11 |
| Dock8 |
| Minpp1 |
| Ccdc124 |
| Cycs;Gm10108 |
| Hdac5 |
| Fabp7 |
| Lyrm5 |
| Nprl3 |
| Osbpl5 |
| Bin2 |
| Cpox |
| Yeats2 |
| Synpo2 |
| Spp1 |
| Akap13 |
| Sbf2 |
| Scgn |
| Cntn6 |
| Serpina1e |
| Inpp5d |
| Ednrb |
| Enpp1 |
| Ranbp10 |
| Commd6 |
| Apba2 |
| Snrpb2 |
| Pdzrn3 |
| Nup85 |
| Ttc27 |
| Hbbt1;Hbb-bs |
| Trim33 |
| Fam175b |
| Sf3b6 |
| Lamb2 |

| |
|---|
| Rb1cc1 |
| Cog6 |
| Amigo1 |
| Dido1 |
| Tsnax |
| Kdm5c |
| Mrps18a |
| Rabac1 |
| Cfh |
| Ada |
| Cdkl5 |
| Cox8a |
| Snf8 |
| Mrps18c |
| Rpl35a |
| Ddx46 |
| Mrpl47 |
| Mia3 |
| Syncrip |
| Slc7a7 |
| Nfya |
| Psme4 |
| Swi5 |
| Fabp4 |
| Rasip1 |
| Sox5 |
| Abcf2 |
| Mib1 |
| B3gnt1 |
| Esam |
| Ranbp3 |
| Csnk1g1 |
| Wwp1 |
| Optn |
| Irs2 |
| Stim2 |
| Mb21d2 |
| Ano10 |
| Clstn1 |
| Polr1a |
| Tubgcp3 |
| Rbm8a |
| Mnat1 |

| |
|---|
| Hm13;H13 |
| Ankrd11 |
| Smarcal1 |
| Akap11 |
| Afp |
| Psmg4 |
| Ctsc |
| Pcdhga9 |
| Elovl4 |
| Dpy19l3 |
| C330007P06Rik |
| Ngly1 |
| Mtif3 |
| Pank2 |
| Thada |
| Dbi |
| Nifk |
| Scfd2 |
| Atp5f1 |
| Ephb1 |
| Lrrfip1 |
| Pcdhb1 |
| Hook3 |
| Rem2 |
| 9230110C19Rik |
| Ano8 |
| Ddx18 |
| Cd99l2 |
| Ldhd |
| Higd1a |
| Usp25 |
| D6Wsu163e |
| Gemin2 |
| Ppwd1 |
| Slc29a2 |
| Rps15 |
| Arhgef38 |
| Cep41 |
| Seh1l |
| Spryd3 |
| Lsm5 |
| Fau |
| Try10 |

| |
|---|
| Ptprc |
| Nars |
| Vamp4 |
| Pcdhb20 |
| Cdk5r1 |
| Etnk1 |
| Chtf8 |
| Klhl42 |
| Sbno1 |
| Eif4enif1 |
| Snx10 |
| Iars |
| Cdc73 |
| Pkig |
| Vps9d1 |
| Slc25a17 |
| Trim47 |
| Pomgnt1 |
| Tmem209 |
| Plrg1 |
| Mterf2 |
| Cog1 |
| Mrps36 |
| Kdm3b |
| Abtb2 |
| Fstl1 |
| Parp9 |
| Palm3 |
| Hnmt |
| Pcbp3 |
| Rasgrf1 |
| D17Wsu104e |
| Gm9242;Hnrnpa3;Gm6793 |
| Mrpl2 |
| Hnrnpl |
| Ppcdc |
| Ssbp1 |
| Tomm70a |
| Klhl22 |
| Jph1 |
| Fdx1 |
| Tefm |
| Nop16 |

| |
|---|
| Sympk |
| Msmo1 |
| Snrpd1 |
| Flywch2 |
| Lppr3 |
| Vwa1 |
| Gtpbp8 |
| Snx32 |
| Atp5e |
| Znf536 |
| Tgfbr3 |
| Psme2 |
| Kiaa1429;1110037F02Rik |
| Hspa14 |
| Spred2 |
| Trmt6 |
| Sec22a |
| Galk2 |
| Dhdh |
| Tbpl1 |
| Golgb1 |
| Adcy8 |
| Pdcd2 |
| Cdk11b |
| Llgl2 |
| Dusp15 |
| Arl10 |
| Smarcad1 |
| Scn3a |
| Ica1l |
| Nanp |
| Prex2 |
| Shc1 |
| Phf21a |
| Agbl4 |
| Gjb6 |
| Noc3l |
| 4933434E20Rik |
| Med22 |
| Plekha1 |
| Natd1 |
| Npr2 |
| Tmem261 |

| |
|---|
| Tulp3 |
| Cdk2ap1 |
| Lss |
| Supt4h1a;Supt4h1b |
| Acyp2 |
| Alg13 |
| Sin3b |
| Timm21 |
| Nsun6 |
| Btbd2 |
| Ftsj3 |
| Stard7 |
| Pdgfra |
| Ppip5k2 |
| Srpk1 |
| Timm17a |
| Sqrdl |
| Ehbp1 |
| Inpp5k |
| Mapkapk5 |
| Gopc |
| Rspry1 |
| Pnck |
| Epcam |
| Rps18;Gm10260 |
| Ndst1 |
| Bbs4 |
| Cpsf4 |
| Bysl |
| Acss1 |
| Parp14 |
| D1Ertd622e |
| Cpa3 |
| Scoc |
| Rasd2 |
| Klk6 |
| Ttc13 |
| Epn3 |
| Rnf25 |
| Xrcc5 |
| Oasl2 |
| Mtss1l |
| Dennd4c |

| |
|---|
| Map2k3 |
| Smc5 |
| Usp38 |
| Coro2a |
| Slc12a4 |
| Pld2 |
| Gng13 |
| Ptma |
| Armcx3 |
| Rbks |
| Gtdc1 |
| Tars |
| Gipc2 |
| Bag2 |
| Lgals9 |
| Fmod |
| Nfatc1 |
| Anp32e |
| Adrm1;Gm9774 |
| Slc43a2 |
| Ptrf |
| Mbnl2 |
| Pfas |
| 08-Sep |
| Cdkal1 |
| Prmt9;Prmt10 |
| Glcci1 |
| Gtf2i |
| Ces1c |
| mt-Nd4;Mtnd4 |
| Usf1 |
| Smarcb1 |
| Ankrd50 |
| Abcc3 |
| Hm13 |
| Rnaseh2a |
| Faap100 |
| Adam8 |
| Abcc8 |
| Mapk8ip2 |
| Xylb |
| Fam98c |
| Itga3 |

| |
|---|
| Gcc1 |
| Jmjd6 |
| Homer2 |
| Mrps25 |
| Dennd2a |
| Puf60 |
| Wdr46 |
| Fam65a |
| Ercc3 |
| Hdgfrp3 |
| Prps1l1 |
| Abhd17c |
| Gga2 |
| Emc7 |
| Sf3a3 |
| Dgkd |
| Heatr1 |
| Faf1 |
| Fermt3 |
| Hnrnpab |
| Abi3 |
| Inpp5f |
| Tbc1d5 |
| G3bp2 |
| Pcm1 |
| Mical1 |
| Bcl9 |
| Ston1 |
| Otud4 |
| Aim1 |
| Lzic |
| Ablim3 |
| Shroom3 |
| Mettl17 |
| Cd44 |
| Ncoa5 |
| Fuca1 |
| Palld |
| Nup160 |
| Gramd4 |
| Svip |
| Nars2 |
| Ugdh |

| |
|---|
| Plekhd1 |
| Pold1 |
| Atg101 |
| Hhatl |
| Ints9 |
| Smc4 |
| Dnajc3 |
| Smc1a |
| Lnp |
| Pde4a |
| Wnk1 |
| Fkbp10 |
| Ifi202 |
| Ccdc97 |
| Col6a3 |
| Ppie |
| Rhbdf1 |
| Trim16 |
| U2af1l4 |
| Rnf126 |
| Bicd1 |
| ptplad1 |
| Sh2b1 |
| Nol11 |
| Ppp1r16b |
| Mak16 |
| Gucy1a3 |
| Uqcrh |
| Wdr91 |
| Peg3 |
| Pigo |
| Rcan2 |
| Sumo3 |
| Haghl |
| Trim65 |
| Pnmal2 |
| Pex6 |
| Unc5c |
| Cdh19 |
| Mslnl |
| Arhgef26 |
| Galnt16 |
| Fam188b |

| |
|---|
| Tmem259 |
| Elovl1 |
| Naa30 |
| Osbpl11 |
| Sun1 |
| Mfsd4 |
| Adck4 |
| Gm20521 |
| Itga2b |
| Neu3 |
| Tmem181a |
| Zfpl1 |
| Cherp |
| Wipi2 |
| Chd11 |
| Lpin2 |
| Pdlim7 |
| Atp5h;Gm10250 |
| Pex13 |
| Ubqln4 |
| Scrg1 |
| Ppil2 |
| Shprh |
| Brd2 |
| Ddx55 |
| Rnaset2 |
| Ccdc88b |
| Srgap1 |
| Sst |
| Habp4 |
| Klhl25 |
| Lars |
| Fdxr |
| Pars2 |
| Vps37c |
| Usp31 |
| Polg2 |
| Cops7b |
| Ngdn |
| 2010107G23Rik |
| Ssh1 |
| Casq2 |
| Cttnbp2nl |

| |
|---|
| Akap8l |
| Rps29 |
| Dhps |
| Inppl1 |
| Txndc16 |
| Nxn |
| Sephs2 |
| Krtap6-1;Krtap6-5;Gm10229 |
| Lage3 |
| Rbm17 |
| Myo1e |
| Serpinc1 |
| Heph |
| Gnl3 |
| Serpina3k |
| Fam96b |
| Ubtf |
| Abca7 |
| Lipt2 |
| Ccdc58 |
| Phf14 |
| Tgoln1;Tgoln2 |
| Hnrnpu |
| Igsf3 |
| Rrm1 |
| Tgfbr2 |
| Tp53bp2;Trp53bp2 |
| Naga |
| Myh11 |
| Ppp2r4 |
| Ptms |
| Trim35 |
| Pnkp |
| Tgfbrap1 |
| Arhgap5 |
| Usp53 |
| Tomm5 |
| Sugp1 |
| Ythdf2 |
| Krt76 |
| Gbp5 |
| Slirp |
| Serpinb2 |

| |
|---|
| Dcxr |
| Ssrp1 |
| Bloc1s3 |
| Usf2 |
| Ppp1r3d |
| Bms1 |
| Crlf3 |
| Dpp4 |
| Nckap1l |
| Itih4 |
| Unc45a |
| Rnf31 |
| Pan3 |
| Nol6 |
| Srsf4 |
| Ms4a15 |
| Mkln1 |
| Lrrk2 |
| Grip2 |
| Cdc16 |
| Sfswap |
| Tmed5 |
| Bri3bp |
| Def6 |
| Dolpp1 |
| Wbp4 |
| Lrrc41 |
| Mtmr6 |
| Usp48 |
| Chst14 |
| Clec4a1 |
| Ccsap |
| Vprbp |
| Nek4 |
| Lrrc14 |
| Fuom |
| Vcpkmt |
| Ubap2 |
| Cav2 |
| Grsf1 |
| Nt5c1a |
| Ttc9c |
| Rin2 |

| |
|---|
| Rab7b;5430435G22Rik |
| Gkap1 |
| Dr1 |
| Ube2i |
| Mtmr10 |
| Sorbs3 |
| Srrm1 |
| Cd3eap |
| Mettl13 |
| Prosc |
| Scaf11 |
| Sptlc2 |
| Rybp |
| Bbs9 |
| Wdr33 |
| Pcdh1 |
| Mon1b |
| Alg11 |
| Apobr |
| Herc3 |
| Cept1 |
| Gm20390;Nme2 |
| St5 |
| Acta2;Actg2 |
| Stk11ip |
| Gdpd2 |
| Psd4 |
| Fcrls |
| Npm1;Gm5611 |
| Mcm3 |
| Max |
| Grm4 |
| Slc41a3 |
| Glrx2 |
| Lsg1 |
| Acsl4 |
| Utp20 |
| Myo1b |
| Dctn6 |
| Fads1 |
| Ggh |
| Arl6 |
| Spon1 |

| |
|---|
| Rnaseh2c |
| Lgi3 |
| Ddx52 |
| Gbp7 |
| Tprg1l;Tprgl |
| Ralgapb |
| Tmed8 |
| Snrpb;Snrpn |
| Pcdh8 |
| Snx24 |
| Ppp4r2 |
| Uri1 |
| Scn7a |
| Kcnj2 |
| Cnot4 |
| Mlip |
| Utrn |
| Serpinb1b |
| Rbm22 |
| Hscb |
| Pnpt1 |
| Dzank1 |
| Cerk |
| Tomm6 |
| Pole3 |
| Snrk |
| Fam81a |
| Riok3 |
| Arl5a |
| Vangl2 |
| Dock11 |
| Meis1 |
| Adal |
| Ppp1r37 |
| Zc3h12b |
| Tceal3 |
| Cbfb |
| Fkbp9 |
| Cstb |
| Ccp110 |
| Tfcp2 |
| Tmcc1 |
| Selt |

| |
|---|
| Atad5 |
| Glyr1 |
| Sh3d19 |
| Fnbp4 |
| Xxylt1 |
| Ppp1r14b |
| Brd9 |
| Nsa2 |
| Moap1 |
| Rpp14 |
| Cx3cr1 |
| Ptcd1 |
| Dag1 |
| Pnkd |
| Ppig |
| Nedd8 |
| Fam49b |
| Rnaseh2b |
| Rragb |
| Pcdhgb6 |
| Wdfy1 |
| Acox2 |
| Smyd2 |
| Eif3h |
| Med14 |
| Tmem106b |
| Cggbp1 |
| Ssr3 |
| Fbp1 |
| Szrd1 |
| Bcas1 |
| Serpinb8 |
| Aif1 |
| Nudcd3 |
| Cnpy2 |
| Lztfl1 |
| Ep300 |
| Lrrc59 |
| Srsf9 |
| Hapln4 |
| Prdx4 |
| Cpeb4 |
| Atf7;Gm28047 |

| |
|---|
| Hlcs |
| Tns3 |
| Gatsl2 |
| Ldb3 |
| Tmtc4 |
| Trim67 |
| Sfr1 |
| Nexn |
| Rfc4 |
| Jakmip1 |
| Mdm1 |
| Cpsf3l |
| H2-Q10 |
| Lphn2 |
| Plekhb2 |
| Cartpt |
| Slc31a1 |
| C2cd2 |
| Tnks1bp1 |
| Rab34 |
| Creld2 |
| Xylt2 |
| Sdk1 |
| Sh2b2 |
| Arrb2 |
| Mt1 |
| Dhrs11 |
| Lars2 |
| Atrn |
| Prom1 |
| Snx18 |
| Rps8 |
| Rcc2 |
| Rdh10 |
| Upf2 |
| Dhrs13 |
| Stag2 |
| Sdf4 |
| Ccdc32 |
| Gne |
| Fa2h |
| Exosc10 |
| Prph |

| |
|---|
| Spred1 |
| Mtpap |
| Nr2c2 |
| Lemd3 |
| Dimt1 |
| Kcna5 |
| Gars |
| Phf10 |
| Nab2 |
| Znf423;Zfp423 |
| Sipa1l3 |
| Tab3 |
| Fkbp15 |
| Smc6 |
| Herpud2 |
| Cmas |
| Scaf1 |
| Npl |
| Cenpv |
| Banf1 |
| Ralyl |
| Trpc4ap |
| Rapgefl1 |
| Ilf3 |
| Reln |
| Prkci |
| Camk2n1 |
| Lpcat3 |
| Rpl26 |
| Gpc6 |
| Smad4 |
| Rer1 |
| Nmd3 |
| Papss2 |
| Usp30 |
| Slc15a2 |
| Gas2l1 |
| Gng11 |
| Cyhr1 |
| Msrb2 |
| Trappc2l |
| Mtmr3 |
| mt-Nd2;Mtnd2 |

| |
|---|
| Tnk2 |
| Rb1 |
| Cdk1 |
| Ncaph |
| Decr2 |
| Ube2g1 |
| Stk32a |
| Wrb |
| Fastkd2 |
| Nsun2 |
| Cope |
| Naa38 |
| Anp32b |
| Tuba1b |
| Fam76a |
| Emc8 |
| FAM120A |
| Jak2 |
| Arsa |
| Ghitm |
| Strip1 |
| Mtg1 |
| Sms |
| Dhx37 |
| Bmp2k |
| Uros |
| Elavl2 |
| Taf6 |
| Eif4g3 |
| Opcml |
| Pet100 |
| P4ha1 |
| Rnps1;Gm9825 |
| Phyh |
| Rps19 |
| Pmm2 |
| Srrm2 |
| Nfatc2 |
| Ankib1 |
| Tjap1 |
| Itfg2 |
| Kiaa1549;D630045J12Rik |
| Evi2a |

| |
|---|
| Rbpj |
| Rnf20 |
| Avil |
| Oasl1 |
| Dusp12 |
| Srp19 |
| Klhl6 |
| Thoc2 |
| Gm2a |
| Ece1 |
| Ighg2c |
| Pdcl3 |
| Fcgrt |
| Nup153 |
| Nfyc |
| Bmi1 |
| Usp12 |
| Commd1;Gm28048 |
| Anapc4 |
| Lamc1 |
| 07-Sep |
| Kiaa1432;C030046E11Rik |
| Wdr41 |
| Arsg |
| Psmb10 |
| Dnmt1 |
| Fbln5 |
| Gpalpp1 |
| Smg8 |
| Smarca1 |
| Jph3 |
| Kiaa0020 |
| Athl1 |
| Atp10d |
| Syk |
| Trmt1l |
| Ss18l1 |
| Traf7 |
| Sos1 |
| Dcp1b |
| Slitrk1 |
| Pon3 |
| Nudt19 |

| |
|---|
| Zcchc6 |
| Gstt3 |
| Mllt4 |
| Atp11a |
| Vezf1 |
| Itga1 |
| Phkb |
| Chmp7 |
| Tmem205 |
| Ccar2 |
| Fam185a |
| Olig1 |
| Parp12 |
| Tmem246 |
| Tnfaip8l3 |
| Nup205 |
| Wwc2 |
| Dnaaf3 |
| Mtr |
| Sdsl |
| Vil1 |
| Csdc2 |
| Dnajb14 |
| Pard3b |
| Cldn10 |
| Plcd3 |
| Trim23 |
| Mrpl13 |
| Cnn3 |
| Crtc3 |
| Ccdc23 |
| Luzp1 |
| Phospho2 |
| Msr1 |
| Maged1 |
| Mtnd5;mt-Nd5 |
| Fip1l1 |
| Myef2 |
| Brap |
| Osbpl7 |
| Sf3b4 |
| Odf2 |
| Casp6 |

| |
|---|
| Adcy1 |
| Ift80 |
| Rhof |
| Tmem56 |
| Agps |
| Pds5b |
| Rnf130 |
| B4galt3 |
| Cda |
| Napepld |
| Fyttd1 |
| Fryl |
| Kat7 |
| Tldc1 |
| Entpd6 |
| Nhp2 |
| Phactr4 |
| Smim8 |
| Fam219a |
| Exosc5 |
| Med25 |
| Ighg1 |
| Polr3b |
| Pde6d |
| Fam98b |
| Mvk |
| Mapk8ip1 |
| Clpx |
| Uhrf1 |
| Ddx25 |
| Parp4 |
| Pkp3 |
| Slc25a16 |
| Asf1a |
| Iah1 |
| Arl6ip4 |
| Tnpo2 |
| Ccdc90b |
| Sall2 |
| Larp1 |
| Fap |
| Ly75 |
| Polr1c |

| |
|---|
| Lrrc40 |
| Sdc3 |
| Plat |
| Rpl9 |
| Prpf38b |
| Rps17 |
| Amt |
| Fibcd1 |
| Paox |
| Aatf |
| Rere |
| Sod3 |
| Zscan21 |
| Metap2 |
| Mul1 |
| Pfkfb2 |
| Ttl |
| Ube2d1 |
| Suz12 |
| Cluh |
| Ftsj1 |
| Yrdc |
| Gpr124 |
| Asic4 |
| Aimp2 |
| Noa1 |
| Naa25 |
| S100a6 |
| Cald1 |
| Tmf1 |
| Panx1 |
| Nrbp1 |
| Ubr1 |
| Emc10 |
| Ica;1300017J02Rik |
| Pgm5 |
| Rps20 |
| Tfg |
| Nup62 |
| Ca10;Car10 |
| Psmg1 |
| Eif2b3 |
| Amdhd1 |

| |
|---|
| Ptgis |
| Lsm3 |
| Clmn |
| Fn1 |
| Mex3a |
| Golph3 |
| Rusc2 |
| Polr3d |
| Rabl3 |
| Creb1 |
| Ulk3 |
| Cyb5d2 |
| Nfkb1 |
| Dvl2 |
| Copg1 |
| Ptgr2 |
| Slc26a2 |
| 2810428I15Rik |
| Zfp651 |
| Cd109 |
| Mecp2 |
| Rpl36 |
| Kctd12b |
| Nop2 |
| Ipo8 |
| Npepl1 |
| Ptpn4 |
| Zdhhc20 |
| Carkd |
| Gpr162 |
| Lman1 |
| Sf3b1 |
| Zfp759 |
| Cdkn1b |
| Smap;1110004F10Rik |
| Sgpl1 |
| Mcm2 |
| Dnajc10 |
| Kiaa2013 |
| Mgat1 |
| Arl2bp |
| Terf2 |
| Trim36 |

| |
|---|
| Tgfb2 |
| Rbm42 |
| Celsr3 |
| Elof1 |
| Ncoa3 |
| Cul4b |
| Wasf2 |
| Thoc6 |
| Rtn4rl1 |
| Tecpr2 |
| Bai2 |
| Carhsp1 |
| Nfrkb |
| Tdrd7 |
| Rsrc2 |
| Esyt2 |
| Secisbp2 |
| Zbtb21 |
| Lztr1 |
| Mrpl9 |
| Ip6k1 |
| Nhlrc2 |
| Cmip |
| Stxbp4 |
| Pkib |
| Gsdmdc1 |
| Fcgr1 |
| D17h6s53e |
| Ifit1 |
| Lamb1 |
| Pvrl2 |
| Rpa3 |
| Caskin2 |
| Mrps18b |
| Hspb8 |
| Pja2 |
| Kctd14 |
| B3galnt1 |
| Ptprt |
| Toe1 |
| Mrps34 |
| Hdac6 |
| Afap1 |

| |
|---|
| Cd34 |
| Itch |
| Ppp2r2d |
| Wdr70 |
| Baz1b |
| Tbc1d13 |
| Ankmy2 |
| Ifitm3 |
| Fbxo21 |
| Pcid2 |
| Tbxas1 |
| Bub3 |
| Umps |
| Rsph9 |
| Tnfaip8l2 |
| Ankle2 |
| Cdh15 |
| Hist1h4a |
| Rpl10a |
| Rwdd4;Rwdd4a |
| Rbbp7 |
| Rgl2 |
| Foxg1 |
| Qtrtd1 |
| Bud31 |
| Mrc1 |
| Pofut1 |
| Cd180 |
| Spock1 |
| Dap |
| Ift140 |
| Bbs2 |
| Alkbh3 |
| Lrfn1 |
| Abcd1 |
| Ndufaf6 |
| Josd2 |
| Tacc2 |
| Pfdn5 |
| Rps27 |
| Slc7a6os |
| Tbc1d16 |
| Utp14a |

| |
|---|
| Zdhhc17 |
| Dicer1 |
| Gh1 |
| Ptprm |
| Chm |
| Lyplal1 |
| Zmat2 |
| Srpr |
| Ptpn12 |
| Atxn2l |
| Serpinf1 |
| Zc3h13 |
| Atr |
| Magee2 |
| P33monox;4833439L19Rik |
| Ca3 |
| Tgm2 |
| Polr2e |
| Cirh1a |
| Hmg20a |
| Pomk |
| Spcs1 |
| Heatr2 |
| Zfyve20 |
| Gm561 |
| Slc7a11 |
| Tusc5 |
| Ift172 |
| Anpep |
| Tor4a |
| Arap1 |
| Slc25a35 |
| Chid1 |
| Frmd7 |
| Ephb2 |
| S100b |
| Mov10 |
| Chd7 |
| Nat2 |
| Man1a1 |
| Nubp2 |
| Matn2 |
| Sema3c |

| |
|---|
| Shc3 |
| Snrnp70 |
| Oas1a |
| Dck |
| Serinc3 |
| Mthfd2 |
| Strada |
| Commd10 |
| Mea1 |
| Erh |
| Dazap1 |
| Zmynd8 |
| Gnpda2 |
| Lamtor4 |
| Ap5b1 |
| Fam57b |
| Snrpc |
| Mrpl24 |
| Snrnp27 |
| Nrip3 |
| Ext2 |
| Fhit |
| Eif3j1;Eif3j2 |
| Igf1r |
| Cdk9 |
| Pik3cg |
| Arhgef40 |
| Nprl2 |
| Kbtbd3 |
| Adarb1 |
| Col3a1 |
| Fcho2 |
| Rpl17 |
| Map4k3 |
| Trit1 |
| Ing1 |
| Gm3839 |
| Adssl1 |
| Ablim1 |
| Mrpl32 |
| Chn2 |
| Chchd1 |
| Srsf7 |

| |
|---|
| Ppp2r5d |
| Dok4 |
| Polr2d |
| Dera |
| Maneal |
| Tpgs1 |
| Pld4 |
| Fbxl15 |
| Sgk2 |
| Myg1 |
| Rbm7 |
| Hist1h1c |
| Acad10 |
| Sgsh |
| Ints7 |
| Rnf114 |
| Mettl3 |
| Snx3 |
| Srsf3;Gm12355 |
| Phf6 |
| Arhgap33 |
| Dzip1;Syce3 |
| Cpped1 |
| Txndc9 |
| Apoa2 |
| Gpcpd1 |
| Znf346;Zfp346 |
| Ccdc115 |
| Api5 |
| Elmo3 |
| Saraf |
| Adra2a |
| Fndc3b |
| Lcat |
| Pbx1 |
| Rbm14 |
| Cul4a |
| Nme4 |
| Clec2l |
| Serpina1d |
| Lcp2 |
| Fam151b |
| Trmt2a |

| |
|---|
| Anapc11 |
| Stard4 |
| Timm8a1 |
| Eprs |
| Stard10 |
| mt-Atp6;Mtatp6 |
| Rpe |
| Smim20 |
| Fam169a |
| Ehd2 |
| Nle1 |
| Gnl3l |
| Arfgap2 |
| Med20 |
| Arglu1 |
| Myt1 |
| Sumo1 |
| Mbd2 |
| Dph7 |
| Pabpc4;Gm10110 |
| Ube2j1 |
| Ddx23 |
| Jmjd1c |
| Alkbh5 |
| Pard6a |
| Ahi1 |
| Mrps11 |
| Atp5b |
| Parvb |
| Depdc5 |
| Alox5 |
| Kiaa1671 |
| Ehmt2 |
| Tssc4 |
| Tcirg1 |
| Tnc |
| Osbpl9 |
| Fxyd7 |
| Ube2r2 |
| Pde8b |
| Chmp2b |
| Ugt1a7c |
| Mx1 |

| |
|---|
| Bgn |
| Il16 |
| Commd7 |
| Ifih1 |
| Map3k12 |
| Arid4b |
| Prpf3 |
| Mtif2 |
| Tmem62 |
| Lyz2 |
| Ppcs |
| Golga7 |
| Dfna5 |
| Znf330 |
| Lgals1 |
| Tdp1 |
| Dpf3 |
| Vps37a |
| Entpd5 |
| Arpp21 |
| Raf1 |
| Spata6 |
| Pxk |
| Zfc3h1 |
| Vasp |
| Rapgef1 |
| Abl1 |
| Ccdc39 |
| Fmn1 |
| Ncapg2 |
| Foxp1 |
| Dis3l2 |
| Cnpy4 |
| Plin3 |
| Cep128 |
| Gm21992;Rbm4 |
| Gabpa |
| Rbm10 |
| Birc6 |
| Fam107b |
| Myo1g |
| Slc13a5 |
| Tsta3 |

| |
|---|
| Stat2 |
| Samd9l |
| Tha1 |
| Nmnat1 |
| Rps12 |
| Rapgef5 |
| Sdad1 |
| Fuca2 |
| Prpf31 |
| Rnf213 |
| Sugp2 |
| Casp8 |
| Pla2g16 |
| Rtp1 |
| Ube4b |
| Nudcd1 |
| Krt33a |
| Gm20441 |
| Eif1 |
| Znf622 |
| Tmeff1 |
| Pctp |
| Med28 |
| Kif21b |
| Ssbp3 |
| Ppil3 |
| Cnot10 |
| Ddx58 |
| Tbc1d9b |
| Rprd2 |
| Tmed2 |
| Ddx21 |
| Calm1 |
| Cdh8 |
| Tapbp |
| Rab36 |
| Cd48 |
| Lnpep |
| Trub2 |
| Wdr75 |
| Kiaa0196 |
| Alb |
| Plekha6 |

| |
|---|
| Jam2 |
| Chchd7 |
| Ttc17 |
| Gbp6;Gbp10 |
| Pde3b |
| Xrcc1 |
| Znf609 |
| Tcerg1 |
| Ckap2l |
| Eepd1 |
| Tbc1d7 |
| Cenpc |
| Brox |
| Snw1 |
| Rps7;Gm9493 |
| Dhx29 |
| Glce |
| Ppp2r2b |
| Srek1 |
| Dchs1 |
| Alg9 |
| Vill |
| Kirrel2 |
| Hspg2 |
| Gale |
| Thbs4 |
| Slc12a7 |
| Ambp |
| Prcc |
| Senp6 |
| B3galtl |
| Tmem260 |
| Tmem186 |
| Ccdc94 |
| Evi5 |
| Slc20a1 |
| Ppp1r12c |
| Vrk3 |
| Tmem240 |
| Fgfr3 |
| Gbp9 |
| Azgp1 |
| Fam171a1 |

| |
|---|
| Ufsp1 |
| Asun |
| Raver1 |
| Rfk |
| Bad |
| Clip3 |
| Kcnc3 |
| Dusp19 |
| Thnsl2 |
| Tmbim6 |
| Mospd2 |
| Fgd1 |
| Rmdn1 |
| Gar1 |
| Cd63 |
| Znf598 |
| Desi1 |
| Ldlr |
| Kiaa1033 |
| Zmym3 |
| Sox2 |
| Nacc1 |
| Swap70 |
| Enpp5 |
| Kctd21 |
| Rpap3 |
| Selm |
| Polr3e |
| Mvb12b |
| Nudt13 |
| Ddx50 |
| Prorsd1 |
| Naa40 |
| Wdr61 |
| Cnot2 |
| Tpmt |
| Ncf2 |
| Deptor |
| Pecr |
| Ass1;Gm5424 |
| B4galnt1 |
| Larp4 |
| Brd7 |

| |
|---|
| Herc6 |
| Skiv2l2 |
| Ints12 |
| Man2a1 |
| Hgh1 |
| Uck1 |
| Xpr1 |
| Mad2l1bp |
| Plau |
| Dpp8 |
| Rap2c |
| Otud7b |
| Ccdc9 |
| Eif2b4 |
| Traf2 |
| Lgmn |
| Skap2 |
| Cdk5rap2 |
| Sgsm3 |
| Riok1 |
| Top1 |
| Fut11 |
| Herc1 |
| Sulf2 |
| Dgat1 |
| Eif3a |
| Fam91a1 |
| Trappc2 |
| Ripk1 |
| Hdac11 |
| Fubp1 |
| Atp11c |
| Acot8 |
| Snupn |
| Ash2l |
| Tbl1x |
| Ncor2 |
| Rab13 |
| Chrm2 |
| Cluap1 |
| Pcx;Pc |
| Tf |
| Pon1 |

| |
|---|
| Znf326;Zfp326 |
| Slit3 |
| Srsf2 |
| Itih5 |
| Ndufaf4 |
| Dpcd |
| Timm22 |
| Edc4 |
| Trim25 |
| Cybb |
| Dennd1a |
| Tbck |
| Xpo5 |
| Znf521 |
| Glt1d1 |
| Dnajc28 |
| Hltf |
| Kctd4 |
| Lmcd1 |
| Rprd1a |
| Tnxb |
| Dync2li1 |
| Adh1 |
| Ciao1 |
| Rassf8 |
| Mup2;Mup10 |
| Gnb2l1 |
| Apoa1 |
| Kctd6 |
| Sec14l1 |
| Pdlim1 |
| Prox1 |
| Eif1ad |
| Csnk1d |
| Xrn1 |
| Gatad2b |
| Pum2 |
| Pogz |
| Entpd1 |
| Dab1 |
| Rnase4 |
| Exosc7 |
| Akr1e2;Akr1e1 |

| |
|---|
| Tceb3 |
| Grcc10 |
| Kif3a |
| St3gal3 |
| Prkaa2 |
| Slain1 |
| Isoc2a |
| Lrch1 |
| Rpl32 |
| Adpgk |
| Pdcd5 |
| Smim12 |
| Palm2;Palm2Akap2 |
| Rgs18 |
| Unc93b1 |
| Hnrnpul2 |
| Surf6 |
| Sec61a2 |
| Plxnb3 |
| Gdap2 |
| Naglu |
| Sema4c |
| Rcan3 |
| Bbs7 |
| Fam122a |
| Klhl5 |
| Rasgef1a |
| Smad2 |
| Raly |
| Tbc1d25 |
| Pdzd2 |
| Mef2d |
| Ca7;Car7 |
| Slc30a7 |
| Hist1h3a |
| 0610011F06Rik |
| Mrpl50 |
| Evi5l |
| Ndnl2 |
| Abca1 |
| Dennd4b |
| Col5a1 |
| Kbtbd7;Zbtbd6 |

| |
|---|
| Mrpl21 |
| Leprel1 |
| Dpf1 |
| Mesdc1 |
| Baiap3 |
| Hbs1l;Gm9923 |
| 2410002F23Rik |
| Parn |
| Saa3 |
| Cd1d1;Cd1d2 |
| Myo16 |
| Fam126a |
| Asna1 |
| Uap1 |
| Wdr12 |
| Ccdc12 |
| Ei24 |
| Chpt1 |
| Rab43 |
| Elac2 |
| Arhgef10l |
| Galnt1 |
| Nhlrc3 |
| Osbp2 |
| Lpcat1 |
| Urm1 |
| Fxn |
| Cd36 |
| Mtmr14 |
| Tmem126b |
| Zmynd11 |
| 1700037H04Rik |
| Txnrd3 |
| Sez6l |
| Slc25a21 |
| Ddx41 |
| Gfpt2 |
| Tmcc3 |
| Bola1 |
| Hs2st1 |
| Fam134a |
| Ndufv3 |
| Gorasp2 |

| |
|---|
| Rsbn1 |
| Mpeg1 |
| Antxr1 |
| Csk |
| Pter |
| Barhl2 |
| Stxbp2 |
| Klhdc8b |
| Cyth2 |
| Rps23 |
| Elp2 |
| Ppat |
| Mrps21 |
| Gng4 |
| Col4a1 |
| 05-Sep |
| Gtf2a1 |
| Cpsf7 |
| Khk |
| Zc3h7b |
| Acbd7 |
| Wdr43 |
| Fam160b1 |
| Ubl4a |
| Cdk13 |
| Rnf13 |
| Ttc21a |
| Celf3 |
| Baz2a |
| Rfc1 |
| Rps10 |
| Gm10094;Sap18 |
| Ankrd28 |
| Dctpp1 |
| Ppp4r4 |
| Nat14 |
| Cox7c |
| Spg20 |
| Zpr1 |
| Htatsf1 |
| Stx17 |
| Ttc33 |
| Usp8 |

| |
|---|
| Rsl1d1 |
| Cwc15 |
| Sema4a |
| Flnc |
| Etv6 |
| Pald1 |
| Nmnat2 |
| Hist1h1b |
| Uvrag |
| Igfbp7 |
| Nelfb |
| Ints10 |
| Nde1 |
| Foxk2 |
| Commd5 |
| Osgep |
| Klhdc10 |
| Map3k4 |
| Pdzd11 |
| Igsf9b |
| Abca8b |
| Exoc1 |
| Fbxl14 |
| Fchsd2 |
| Tubal3 |
| Btaf1 |
| Prr7 |
| Dguok |
| Wbscr16 |
| Med6 |
| Rasal2 |
| Bcl10 |
| Mrpl49 |
| Dgkg |
| Celf6 |
| Usp1 |
| Nudt16l1 |
| Set |
| Cyp51a1 |
| Zmat4 |
| Cbx8 |
| Top2a |
| Znf706 |

| |
|---|
| Arhgef10 |
| A830010M20Rik;Kiaa1107 |
| Chil3 |
| Ano3 |
| Ivns1abp |
| Ppfibp2 |
| Sepsecs |
| Rasgrp1 |
| Terf2ip |
| Zc3h15 |
| Heatr5a |
| Ccdc53 |
| Pak7 |
| Galns |
| Ptrh1 |
| Hdac8 |
| Usp28 |
| Fmn2 |
| Actl6a |
| Rps25 |
| St18 |
| Nup98 |
| Pex11b |
| Ctnnbip1 |
| Znf385b;Zfp385b |
| Cblb |
| Cdc37l1 |
| Ncoa1 |
| Lsm14a |
| Mdn1 |
| Nub1 |
| Epha6 |
| Pdlim3 |
| Dnttip1 |
| Nfib |
| Rbbp5 |
| Aif1l |
| Trhde |
| Ulk2 |
| Sirt7 |
| Slc39a7 |
| Wdr5 |
| Rbm12b2 |

| |
|---|
| Khdrbs2 |
| Ugcg |
| Necap2 |
| Sart1 |
| Slit1 |
| Cdh23 |
| Dach1 |
| Scly |
| Pspc1 |
| Nmt2 |
| Bag1 |
| Klhl3 |
| Vav3 |
| Blnk |
| Trim27 |
| Znfx1 |
| Tubgcp6 |
| Arhgap18 |
| Gtpbp4 |
| Arhgap25 |
| Cp |
| Cdh20 |
| Btf3l4 |
| Mios |
| Ogn |
| Tcf20 |
| Lsm1 |
| Rad50 |
| Sez6 |
| Tceb1 |
| Sgms1 |
| Hist1h1e |
| Timp2 |
| Txndc12 |
| Ankrd17 |
| Marcksl1 |
| Atp2c1 |
| S100a8 |
| Mtrf1l |
| Ubr2 |
| Larp6 |
| Clmp |
| Rdh13 |

| |
|---|
| Pdzd4 |
| Secisbp2l |
| Chtop |
| Zbtb8os |
| Gnpat |
| Otulin |
| Slc33a1 |
| Mrpl30 |
| Clstn2 |
| Tprn |
| Sub1 |
| Gm20671;Pisd |
| Yeats4 |
| Ighg2b;Igh-3 |
| S100a1 |
| 06-Mar |
| Mrpl40 |
| Etnppl |
| Mex3c |
| Nme7 |
| Mphosph10 |
| Slc7a3 |
| Ikbkb |
| Gmeb2 |
| Usp10 |
| Tmem263 |
| Plekhg5 |
| Rnf123 |
| Ubap2l |
| Cnnm3 |
| Rplp1 |
| Agpat6 |
| Sobp |
| Rpl38 |
| B3gat3 |
| Mapk4 |
| Rnf181 |
| Csrp2 |
| Nbas |
| Ptk7 |
| Brinp2 |
| Rnf219 |
| Ikbkap |

| |
|---|
| Surf4 |
| Cds1 |
| Rpa1 |
| Iqgap1 |
| Lzts2 |
| Serping1 |
| Fam120c |
| Vash1 |
| Alkbh7 |
| Golga5 |
| Igf2bp2 |
| Wipf2 |
| Gpld1 |
| Dhx35 |
| Pelo |
| Aifm2 |
| Smoc1 |
| Prim2 |
| Lrsam1 |
| Chgb |
| F8a;F8a1 |
| Ift74 |
| Ltn1 |
| Dnajc25 |
| Wsb2 |
| Dnajc19 |
| Hcls1 |
| Lrrc1 |
| Xpot |
| Comt |
| Hexa |
| Unc119b |
| Gys1 |
| Dffa |
| Nasp |
| Dapk1 |
| Elp4 |
| Nadk |
| Smarcd2 |
| R3hdm1 |
| Apbb2 |
| Mb |
| Cbwd1 |

| |
|---|
| Hps6 |
| Nxph1 |
| Adss |
| Rimkla |
| Rbmx |
| Hapln2 |
| D10Jhu81e |
| Rab33a |
| Sae1 |
| Tceal1 |
| Tufm;Gm9755 |
| Acvr1 |
| Pnpo |
| Ptdss1 |
| Nhsl1 |
| H2afv;H2afz |
| Emp2 |
| Fam65b |
| Fhod1 |
| Lmf1 |
| Kynu |
| Ptpdc1 |
| Snrnp40 |
| Chml |
| Oxsm |
| Sh2d3c |
| Pgpep1 |
| Sap30bp |
| Rims4 |
| Xpnpep3 |
| Fga |
| Lgals3bp |
| Trp53i11 |
| Hirip3 |
| H2afx |
| Ndor1 |
| Cmtr1 |
| Gtf2f1 |
| Cars |
| G3bp1 |
| Rbms1 |
| Entpd3 |
| Ptbp1 |

| |
|---|
| Pelp1 |
| Dyrk1a |
| Zdhhc6 |
| Apip |
| Enox2 |
| Pak4 |
| Adamts5 |
| Nes |
| Kdelc2 |
| Dnmbp |
| Slc22a17 |
| Sel1l3 |
| Kif13b |
| Trabd |
| Rasa3 |
| Thumpd1 |
| Fgb |
| Satb1 |
| Siae |
| Glra1 |
| Omp |
| Zfp532;Znf532 |
| R3hcc1 |
| Irak4 |
| Syne2 |
| Pias1 |
| Efnb1 |
| Mars2 |
| Ttr |
| Bzw2 |
| Map4k5 |
| Cdk7 |
| Phactr2 |
| Inpp4b |
| Ankzf1 |
| Hnrnpa1 |
| Efcab14 |
| Adipoq |
| Actr6 |
| Cog2 |
| Tbc1d9 |
| Wdr55 |
| Sec63 |

| |
|---|
| Tbc1d22b |
| Sh3bgrl2 |
| Sssca1 |
| Cd1d1 |
| Scg2 |
| Ngp |
| Fbxo44 |
| Kiaa1045 |
| Tma7 |
| Gab2 |
| Vimp |
| Hivep2 |
| Lpin1 |
| Ddhd1 |
| Pcsk1 |
| Cpq |
| Ttc3 |
| Apod |
| Kiaa1598 |
| Ccdc141 |
| Pecam1 |
| Med16 |
| Chd1 |
| Bmpr1a |
| Lcmt1 |
| Dgcr8 |
| Sppl2a |
| Mrpl22 |
| Mif4gd |
| Scaf8 |
| Pcnxl3 |
| Ccnh |
| Tes |
| Cck |
| Naprt |
| Hspb6 |
| Polr2l;Gm10774 |
| Ctc1 |
| Mapk11 |
| Pus10 |
| Cdk5r2 |
| Exoc6 |
| Apoa4 |

| |
|---|
| Polr2k |
| Pde12 |
| Ccdc28a |
| Sgpp1 |
| Itsn2 |
| Nup155 |
| Romo1 |
| Ubxn2b |
| Polr2b |
| Yipf4 |
| Agt |
| Slc39a10 |
| Rpl19 |
| Mut |
| Pcsk1n |
| Prrc2a |
| Fam120b |
| Copa |
| Npdc1 |
| Gpr37 |
| Lrch4 |
| Pdp2 |
| Sra1 |
| Ptplb |
| Tmem19 |
| Rif1 |
| Iap |
| Plcb3 |
| Nfx1 |
| Nav3 |
| Celf1 |
| Bap18;0610010K14Rik |
| Lpxn |
| Mbnl1 |
| Ppfibp1 |
| Abcd4 |
| Atad2b |
| Cabp1 |
| Parp1 |
| Edf1 |
| Acin1 |
| Atp5j |
| Myrip |

| |
|---|
| Ndufa1 |
| Ubxn4 |
| Rnd2 |
| Kpna1 |
| Rab1 |
| Gm20604;Gm28051;AK010878 |
| Mrto4 |
| Trmt10a |
| Fam45a |
| Aftph |
| Abhd17b |
| Hba-a2;Hba |
| Ndufc1 |
| Gpr17 |
| Setd3 |
| Bnip2 |
| Ca13 |
| Ebf3 |
| Qrich1 |
| Dcn |
| Mrpl57 |
| Mrps30 |
| Ercc4 |
| Dis3 |
| Nrp1 |
| Mblac1 |
| Urod |
| Egfr |
| Emc4 |
| Mtmr12 |
| Rpia |
| Ahctf1 |
| C1qa |
| Vwa9 |
| Garem |
| Atp5d |
| Fam195b |
| Rpl13a |
| Akap1 |
| Cog5 |
| Ppp1r2 |
| Glrb |
| Ppp1r11 |

| |
|---|
| L7rn6;l7Rn6 |
| Prpf38a |
| Gtf2b |
| 03-Sep |
| Ythdc1 |
| Mrgbp |
| Exosc1 |
| Nptx2 |
| Hs1bp3 |
| Itpk1 |
| Lrrc8d |
| Ganc |
| Flrt1 |
| Hdgfrp2 |
| Slc25a31 |
| Hmbox1 |
| Nmi |
| Eif3c |
| Clec16a |
| Cbln2 |
| Nr2c2ap |
| Rbms2 |
| Itga6 |
| Slc30a5 |
| Snx15 |
| Serf2;Serf1 |
| Gatad1 |
| Ctsf |
| Ppapdc1a |
| Ilkap |
| Gng10;Gm20503 |
| Akap8 |
| Engase |
| Fez2 |
| Rbm33 |
| Pou3f3 |
| Scpep1 |
| Trip4 |
| Dbr1 |
| Hddc2 |
| Ppp1r9a |
| Neurod1 |
| Sephs1 |

| |
|---|
| Mrrf |
| Ptpn6 |
| Hn1l |
| Cemip |
| Hmgcs1 |
| Opalin |
| Sirt4 |
| Bptf |
| Ifggb5 |
| Gdpgp1 |
| Sf3a2 |
| Acss2 |
| Rtn4rl2 |
| Jph4 |
| Tmem132b |
| Pla2g7 |
| Serpini1 |
| Gpbp1 |
| Cstf2t |
| Vezt |
| Srp14 |
| Cant1 |
| Gcat |
| Adprm |
| Pdlim2 |
| Pcgf2 |
| Col6a1 |
| Uba2 |
| Eogt |
| Rimklb |
| Braf |
| Arvcf |
| Pik3r5 |
| Tada1 |
| Tpst1 |
| Cdr2l |
| Kif3c |
| Ctgf |
| Zic1 |
| Ttc37 |
| Reep3 |
| Mrpl14 |
| Map2k5 |

| |
|---|
| Glb1 |
| Cdh6 |
| Sec24b |
| Fos |
| Fbn1 |
| Hmgcr |
| St3gal5 |
| Mkrn2 |
| Dak |
| Ift22 |
| Col12a1 |
| Megf8 |
| Fhl3 |
| Tbl3 |
| Gpatch11 |
| Pkn1 |
| Adcy7 |
| Sh3bgrl |
| Arhgap24 |
| Ybx1 |
| Matk |
| St8sia3 |
| Eif4g2 |
| Dnajc27 |
| Kdm2b |
| Ubl5;Gm16381 |
| Atp2b3 |
| Ufm1 |
| Rasal3 |
| Irf3 |
| Mertk |
| Parp3 |
| Sema4d |
| Ahdc1 |
| Stam2 |
| 9030617O03Rik |
| Atxn1 |
| Dusp23 |
| As3mt |
| Rps6kb1 |
| Brk1 |
| Tll1 |
| Kiaa0319l;AU040320 |

| |
|---|
| Homer3 |
| Gas2 |
| Smdt1 |
| Prpf40b |
| Scamp2 |
| Ogfod1 |
| Phldb2 |
| Rps2;Gm5786;Gm18025;Gm6576;Gm8225 |
| Bloc1s2 |
| Tmed4 |
| Edem3 |
| Ergic2 |
| Ccser2 |
| Chmp1b1 |
| Ormdl3 |
| Ripk2 |
| Sacs |
| Unk |
| Lonrf2 |
| Sypl1;Sypl |
| Maf1 |
| Smek1 |
| Ctsa |
| Mettl16 |
| Crhbp |
| Pcbp4 |
| Ppp1r14c |
| Xrcc6 |
| C2cd4cC2CD4 family |
| Myo1f |
| Casp1 |
| Tm9sf4 |
| Ncl |
| Sde2 |
| Rps14 |
| Pomp |
| Tinagl1 |
| Col18a1 |
| Sipa1 |
| Rsf1 |
| Lrp8 |
| Rnf7;Gm7075 |
| Elp3 |

| |
|---|
| Fam73a |
| F2 |
| Itgax |
| Sptbn4 |
| Tpp1 |
| Uxt |
| Rpp25l |
| Atg2a |
| Ppp1r16a |
| Hmha1 |
| Atrx |
| Atg4b |
| Atp5g2;Atp5g3 |
| Gucy1b3 |
| Tpk1 |
| Atl3 |
| Gprasp1 |
| Patz1 |
| Herc4 |
| Hn1 |
| Ilvbl |
| S1pr5 |
| Ppdpf |
| Ppip5k1 |
| Cnot8 |
| Raver2 |
| Rrm2 |
| Amer2 |
| Agl |
| Dcakd |
| Ythdf1 |
| Fbxo42 |
| A2m;Pzp |
| Gbp2 |
| Zkscan3 |
| Prps1;Prps1l3 |
| Tcf25 |
| Plcd4 |
| Nacad |
| Eml1 |
| Alpl |
| Pmch |
| Ccdc88a |

| |
|---|
| Tmppe |
| Tarbp2 |
| Ube2a |
| Cebpz |
| 4931406P16Rik |
| Paxbp1 |
| Pwp2 |
| Ssh2 |
| Farsb |
| Cbx5 |
| Snn |
| N6amt2 |
| Ube3c |
| Ddx56 |
| Tsc22d4 |
| Eef1e1 |
| Nipbl |
| Cnn1 |
| Ccdc85a |
| Iigp1 |
| Fgd2 |
| Wbp11 |
| Fam63a |
| Kcnj6 |
| Gfer |
| Clcc1 |
| Cdc5l |
| Zbtb7a |
| Ecsit |
| Yars |
| Macrod2 |
| Camsap2 |
| Gns |
| Amotl1 |
| Gpaa1 |
| Tle1 |
| Heg1 |
| Cwc22 |
| Elfn1 |
| Kdm4b |
| Scg3 |
| Ap4e1 |
| Tprkb |

| |
|---|
| Phlpp2 |
| Was |
| Gabpb1 |
| Yif1b |
| Lppr4 |
| Smg1 |
| Slc27a1 |
| Atp6v0c |
| Mtdh |
| Gamt |
| Sdhaf2 |
| Arpc1b |
| Mepce |
| Zc3h7a |
| Cdk5rap3 |
| Bace1 |
| Pnpla7 |
| Gpr56 |
| Ift27 |
| Naf1 |
| Tspan2 |
| Prmt3 |
| Arhgef25 |
| Amot |
| Trip10 |
| Fastkd5 |
| Cdk4 |
| Golga3 |
| Grid2 |
| Tgfb1 |
| Ttc39b |
| Tmem165 |
| Palmd |
| Casp7 |
| Rab28 |
| Fgd4 |
| Gid4 |
| Hdac1;Gm10093 |
| Tap1 |
| Ap4m1 |
| Asphd1 |
| Eif4e3 |
| Gmip |

| |
|---|
| Chga |
| Abce1 |
| Fut9 |
| Chd4 |
| Kiaa1161 |
| Trappc1 |
| Ptbp3 |
| Hmgb2 |
| Tub |
| Slc35b2 |
| Bai1 |
| Ddx6 |
| Abcc4 |
| Megf10 |
| Fbxo7 |
| Papss1 |
| Polr2j |
| Galt |
| Lipa |
| Phf1 |
| Capg |
| Eftud1 |
| Snap23 |
| Emilin1 |
| Ndc1 |
| Slc4a1ap |
| Cnksr3 |
| Fat2 |
| Ttc5 |
| Sh3bp1 |
| Tmcc2 |
| Bche |
| Galm |
| Trappc8 |
| Kidins220 |
| Renbp |
| Ephb6 |
| Mob2 |
| Nyap2 |
| Zfpm2 |
| Micu2 |
| Naa35 |
| Sytl2 |

Mrpl19
Abca2
Chd9
Kiaa1841;0610010F05Rik
C4b
Ntan1
Zfyve27
Mocs2
Clns1a
Cxxc1
Mcc
Tbc1d19
Tra2a
Lrrc25
Sp9
Krt85
Med1
Slc38a2
Tnfaip2
Cdc23
Cers1
Pi4kb
Tubgcp4
Cdan1
Gtf2h2
Dhx30
Pank3
Ddx39b
Cacul1
Gtf3c4
Efnb2
Snrpf
Hkdc1
Lphn3
06-Sep
P2rx4
Rbck1
Ddx27
Sf3b5
Cxx1c
Il1rap
Kcnip1
Rpl29;Gm17669;Gm10709;Gm3550

| |
|---|
| Zfand5 |
| Dapk3 |
| Ctso |
| Igf2bp1 |
| Samd14 |
| Ptpn21 |
| 4930402H24Rik |
| Gabarap |
| Cfi |
| Serpind1 |
| Atpif1 |
| Vps13d |
| Plekho2 |
| Ccdc186 |
| Fam115a |
| Ccnk |
| Rnf113a2 |
| Arg2 |
| Msi1 |
| Atf7ip |
| Tyms |
| Manf |
| Srd5a3 |
| Slain2 |
| Cmc1 |
| Lemd2 |
| Rpl7a |
| Nupl1 |
| Nfxl1 |
| Tmem178b |
| Plek |
| Fkbp5 |
| Upp1 |
| Hars |
| Ctbs |
| Otud7a |
| Ankrd13d |
| Dhx33 |
| Col14a1 |
| Podxl2 |
| Micall2 |
| Tbc1d2b |
| Smug1 |

| |
|---|
| Gmfg |
| Med13 |
| Nova2 |
| Slfn9 |
| Zeb1 |
| Eif2d |
| Nop14 |
| Pkd2 |
| Pus1 |
| Clip4 |
| Dars |
| Erbb2ip |
| Hectd3 |
| Cyp2d22 |
| Coq4 |
| Cst3 |
| Sfpq |
| Mid1ip1 |
| Med11 |
| Usp47 |
| Trp53bp1 |
| Bckdhb |
| Mycbp |
| Larp4b |
| Cldnd1;Cldn25 |
| Hk3 |
| Fer |
| Aacs |
| Mcfd2 |
| Rpl18a |
| Frmd4b |
| Tgs1;Gm5117 |
| Sarnp |
| Nr2f1 |
| Aamdc |
| Smpd2 |
| Tgfbi |
| Stambpl1 |
| Mrpl16 |
| Nol9 |
| Thumpd3 |
| Plekhf2 |
| Klf13 |

| |
|---|
| Rnf157 |
| Ints4 |
| Clca1 |
| Lbr |
| Mrpl27 |
| Ppil4 |
| Rpp30 |
| Ctr9 |
| Mrpl3 |
| Mars |
| Cgnl1 |
| Rasa1 |
| Acy3 |
| Hist2h2bb;Hist1h2bm |
| Dph2 |
| Txnip |
| Zc3h18 |
| Hspb1 |
| Chmp3 |
| Fnta |
| Ubr5 |
| Tmem222 |
| Kif15 |
| Agtpbp1 |
| Gna12 |
| Pik3cb |
| Nap1l5 |
| Srsf12 |
| Mast1 |
| Usp22 |
| Adam9 |
| Cry2 |
| Col4a6 |
| Atxn2 |
| Hsd11b1 |
| Abhd4 |
| Dock7 |
| Haus3 |
| H1fx |
| Cnga2 |
| Snx13 |
| Mpz |
| Aass |

| |
|---|
| Ctu2 |
| Abcf3 |
| Scn9a |
| Rrp9 |
| Dtnbp1 |
| Bid |
| Mst4;2610018G03Rik |
| Rnpc3 |
| Syt6 |
| Lama4 |
| Magt1 |
| Phc2 |
| Nemf |
| Ttc28 |
| Nubpl |
| Zfr2 |
| Rel |
| Eefsec |
| Fam184a |
| Mt3 |
| H2-K1 |
| Jmjd7;Gm28042 |
| Arhgap30 |
| Atp13a5 |
| Afap1l1 |
| Kti12 |
| Nop10 |
| Cacnb2 |
| Unkl |
| Mesdc2 |
| Lgals8 |
| Sp110 |
| Creg1 |
| Hmgb3;Gm6104 |
| Xdh |
| Hmox1 |
| Mrps16 |
| Pddc1 |
| Bag3 |
| Crebbp |
| Mfap1 |
| Ell |
| Plbd2 |

| |
|---|
| Zfr |
| Ptar1 |
| Rc3h2 |
| Jakmip2 |
| Myl6b |
| Wibg |
| Fbl |
| Slc7a6 |
| Spata5 |
| Fto |
| Wapal |
| Alyref |
| Kctd10 |
| Trmt11 |
| Satb2 |
| Pak1ip1 |
| Mrps35 |
| Mrpl43 |
| Mpdz |
| Tifa |
| Lrig1 |
| Hp1bp3 |
| Kcnb2 |
| Ralgps2 |
| Ago3 |
| Rilpl1 |
| Nfil3 |
| Ogfr |
| Eif2ak4 |
| Relb |
| Ace |
| Bcat2 |
| Mtss1 |
| Lrch2 |
| Kdm4a |
| Prrc2c |
| Igf2r |
| Eef2k |
| Wdr74 |
| Pyroxd2 |
| Cdkn2aip |
| Tusc2 |
| 9330182L06Rik |

| |
|---|
| Bola2 |
| Tbc1d2 |
| Rrbp1 |
| Osbpl6 |
| Qki |
| Ncbp2 |
| Fbxo38 |
| Phyhd1 |
| Cygb |
| Golph3l |
| Slc37a2 |
| Nup88 |
| Smad1 |
| Mcm5 |
| Serpinh1 |
| Ppm1g |
| Rap1gds1 |
| Slc16a2 |
| Arid2 |
| Msh2 |
| Adnp |
| Pax6 |
| Pycard |
| Kmt2a |
| Rbm47 |
| Itgb3 |
| Bod1 |
| Arhgef18 |
| Nat8l |
| Bdh2 |
| Pvrl3 |
| Ptgds |
| Ddx49 |
| Dusp14 |
| Dhx38 |
| Alox15 |
| Asap2 |
| Fam102a |
| Rtn2 |
| Cbx4 |
| Rbpms |
| Pdlim4 |
| Klhl9 |

| |
|---|
| Impa2 |
| Fam213b |
| Dek |
| Syt11 |
| Acot13 |
| Slc6a15 |
| Adrbk1 |
| Tmem223 |
| Senp8 |
| Irf2bp2 |
| Phkg2 |
| Rpl4 |
| Nudt7 |
| Kiaa1467 |
| Dact3 |
| Ubxn2a |
| Acp2 |
| Spats2 |
| Lyrm4 |
| Kcnj11 |
| Dars2 |
| Sart3 |
| Slc9a6 |
| Clgn |
| Hdac2 |
| Pfdn1 |
| Hnrnph3 |
| Apoc3 |
| Gpatch1 |
| Pitpnm2 |
| Cstf3 |
| Ccbl2 |
| Tsc22d2 |
| Col15a1 |
| Zfp644 |
| Ago2 |
| Appbp2 |
| Fv1 |
| Dhx57 |
| Naip2 |
| Ca5b |
| 2700097O09Rik |
| Cntn3 |

| |
|---|
| Penk |
| Gpc3 |
| Adap2 |
| Suv420h2 |
| Lactb2 |
| Rbm39 |
| Hecw1 |
| Fn3krp |
| Pkp2 |
| Tyk2 |
| Gcc2 |
| Tram1 |
| Cdk18 |
| Esrra |
| Inpp5j |
| Ppapdc2 |
| Sin3a |
| Bccip |
| Nelfcd |
| Egln1 |
| Hdhd2;Ier3ip1;Gm10784 |
| Ybx3 |
| Dnlz |
| Tor2a |
| Ca14 |
| Rnf41 |
| Epb42 |
| Mpv17 |
| Trappc5 |
| Mms19 |
| Abhd14b |
| Pdgfrb |
| Rpl35;Gm10269 |
| Amfr |
| Hypk |
| Odr4 |
| Stau1 |
| Bop1 |
| Akt1 |
| Rps28 |
| Cpeb2 |
| Ccdc85c |
| Slc6a5 |

| |
|---|
| Hid1 |
| Tcf4 |
| Neurod2 |
| Cpsf3 |
| Zhx3 |
| Nt5c2 |
| Aup1 |
| Fzd3 |
| Ltbp4 |
| Stau2 |
| Utp15 |
| Tbl1xr1 |
| Dnajb12 |
| Rtp2 |
| Col11a1 |
| Chchd10 |
| Scg5 |
| Sos2 |
| Rtn4ip1 |
| Atpaf2 |
| Tlr13 |
| Epha7 |
| Ric8b |
| Ewsr1 |
| Gipc1 |
| Smurf1 |
| Maz |
| Ntmt1 |
| Dscr3 |
| Hspa13 |
| Focad |
| Gulp1 |
| Exosc3 |
| Lpp |
| Heatr6 |
| A2mp |
| Cirbp |
| Rcl1 |
| Mtcl1 |
| Ssbp2 |
| Gjc3 |
| Tiam1 |
| Sec24d |

| |
|---|
| Crtc1 |
| Gbp4 |
| Rps6kc1 |
| Hook1 |
| Scaper |
| Mre11a |
| Rnasel |
| Cactin |
| Cyp20a1 |
| Cdh4 |
| Rasgrf2 |
| Kctd1 |
| Dopey2 |
| Sash3 |
| Ubac2 |
| Dnajc30 |
| Tspan15 |
| Hnrnpm |
| Gpn1 |
| Xab2 |
| Cog7 |
| Triobp |
| Myo1d |
| Mrps24 |
| Wrnip1 |
| Cox19 |
| Safb |
| Emc6 |
| Timm10b |
| Cetn3 |
| Pex10 |
| Accs |
| Pard3 |
| Ctps1 |
| Tmem109 |
| Abcd2 |
| Kcnq3 |
| Ctcf |
| Lingo3 |
| Myo9b |
| Ten1 |
| Parg |
| L3hypdh |

| |
|---|
| Rxrb |
| Papola |
| Pold2 |
| Fam69b |
| Mthfsl |
| Atp5s |
| Pgk2 |
| Wdr26 |
| Elp5 |
| Mettl14 |
| Emr1 |
| Usp32 |
| Ncoa6 |
| Gca |
| Orc4 |
| Llgl1 |
| Kpna6 |
| Mapk15 |
| Mta3 |
| Sall1 |
| Npc2 |
| Pknox2 |
| Fmo5 |
| Usp39 |
| Cobl |
| Gng8 |
| Fbxo11 |
| Fat4 |
| Psip1 |
| Rgs3 |
| Map2k2 |
| Tlr3 |
| Scaf4 |
| Sec61b |
| Coq10a |
| Rps4x;Gm15013 |
| Pikfyve |
| Cabin1 |
| Snx33 |
| Cwf19l1 |
| Atxn7l3b |
| Smg6 |
| Extl3 |

| |
|---|
| Tmpo |
| Znf512b |
| Kif16b |
| Itga4 |
| Speg |
| Snx17 |
| Ces1d |
| C1qc |
| Uaca |
| Rell2 |
| Mb21d1 |
| Fam217b |
| Tspan9 |
| Sun2 |
| Tstd1 |
| Rpp25 |
| Adck3 |
| Limk2 |
| Aatk |
| Elovl5 |
| Cecr5 |
| Pstpip2 |
| Nqo1 |
| Dok2 |
| S100a10 |
| Lrrc16a |
| Ints2 |
| Pex5 |
| Shb |
| Brd3 |
| Trmt112 |
| Lrfn2 |
| Aff4 |
| Tpgs2 |
| Dhx16 |
| Erlin1 |
| Dock1 |
| Casc4 |
| Fgfr1op2 |
| Ddx54 |
| Trappc13 |
| Met |
| Dnal1 |

Ubxn1
Xiap
Hsd17b7
Apaf1
Ralgapa2
Snx29
Slc4a2
Mttp
Bloc1s4
Tti1
Gvin1
Spag7
Dzip3
Mgrn1
Uxs1
Wipi1
Mrpl55
Spats2l
Thrap3
Thg1l
Hvcn1
Zwint
Glmn
Ranbp2
Ctif
Ift52
Ccdc25
Dnajc2
Smc3
Inadl
Pex7
Manba
Ercc1
Rpl22l1
Pde4b
Pld1
Dhx15
Thoc5
Cnpy3
Numa1
Mylk
Ate1
Sdcbp

| |
|---|
| Inpp5e |
| Mvd |
| Whsc1l1 |
| F11r |
| Fam188a |
| Cr1l |
| Ddrgk1 |
| D2hgdh |
| Rfx1 |
| Ifi203 |
| Arhgap4 |
| Apoh |
| Wash1 |
| Emg1 |
| Hap1 |
| Rab26 |
| Pign |
| Rps21 |
| Unc5b |
| Wars2 |
| Slitrk3 |
| Ndufb2 |
| Dnase2;Dnase2a |
| Preb |
| Igkc |
| Arhgap22 |
| Fam129a |
| Hnrnpd |
| Uqcc2 |
| Sec16a |
| Arid1b |
| Map7d2 |
| Eif4ebp1 |
| Ldlrap1 |
| Aoc3 |
| Grip1 |
| Srsf5 |
| Usp4 |
| Lgi4 |
| Anln |
| Colec12 |
| Znf385a |
| Sox6 |

| |
|---|
| Yme1l1 |
| Tmlhe |
| Tradd |
| Nol7 |
| No66 |
| Exd2 |
| Farsa |
| Irs1 |
| Tlr7 |
| Mtmr7 |
| Ctdp1 |
| Luc7l |
| Dennd5a |
| Elf2 |
| Gtf2e1 |
| Cav1 |
| Rps3a |
| Cnbp |
| Ubap1 |
| Ngb |
| Ublcp1 |
| Msl1 |
| Eny2 |
| Casq1 |
| Fgfr1op |
| Lurap1l |
| Pigg |
| Ppp1r14a |
| Casp3 |
| Ppp3cc |
| Gpnmb |
| Ube2h |
| Keap1 |
| Pde9a |
| Use1 |
| Phlpp1 |
| Kbp;2510003E04Rik |
| Dut |
| Capn7 |
| Cnot6 |
| Herc2 |
| BC024978 |
| Slc7a1 |

| |
|---|
| Ptpn2 |
| Sparc |
| Mogs |
| Atg14 |
| Trmu |
| Dusp28 |
| Yod1 |
| Nudt17 |
| Spast |
| Endou |
| Vwf |
| Tagln |
| Tyro3 |
| Pdcd11 |
| Ythdf3 |
| Ccdc50 |
| Ttc38 |
| Ccdc104 |
| Lsm12 |
| Psd2 |
| Chmp1b2 |
| Mapkap1 |
| Rufy2 |
| Pard6b |
| Cd74 |
| Gm10704 |
| Gm20425 |
| Stxbp6 |
| Ccdc40 |
| Pcnp |
| Mllt11 |
| Gpr107 |
| Serpina1b |
| Ctnnal1 |
| Rpl6;Gm5428 |
| Tmem160 |
| Eif2s3y |
| Snx8 |
| Stom |
| Rras |
| Tdrp |
| Prkra |
| Thyn1 |

| |
|---|
| Mrpl10 |
| Purg |
| Cisd3 |
| Ksr1 |
| Map3k10 |
| Taf15 |
| Baz2b |
| Wdr82 |
| Naa50 |
| Slc6a13 |
| Cobll1 |
| Itih1 |
| Ddx31 |
| Gm11568;Krtap9-1;Gm11559;Gm11567 |
| Npm3 |
| Foxj3 |
| Hmga1 |
| Fggy |
| Ddx10 |
| Vps37b |
| Kcnip3 |
| Pofut2 |
| Sec24a |
| Tmem55a |
| Ttll1 |
| Stab1 |
| Hras |
| Nphp4 |
| Nup43 |
| Cmtm6 |
| Utp6 |
| Zw10 |
| Ankrd34a |
| Il18 |
| Prr12 |
| Atp5sl |
| Hspb11 |
| Tbk1 |
| Rab32 |
| Dbh |
| Cenpf |
| Eif1ax |
| Cuedc2 |

| |
|---|
| Cnot3 |
| Syngr2;Gm20708 |
| Fcho1 |
| Hsbp1 |
| Rabepk |
| Lbp |
| Lrrfip2 |
| Pbx3 |
| Sntb2 |
| Prune |
| Man2b2 |
| Cic |
| Glyatl3 |
| Dpy30 |
| 8030462N17Rik |
| Zfyve16 |
| Dock5 |
| Ube2j2 |
| Magi3 |
| Cramp1l |
| Polr2g |
| Golga1 |
| Mdga1 |
| Tex10 |
| Cdk2 |
| Irf2bpl |
| Chd5 |
| Samd4a |
| Wac |
| Ddx60 |
| Kbtbd2 |
| Gm20431;Ube2v1 |
| Ca1 |
| Med15 |
| Adcyap1r1 |
| Hnrnpc |
| Rpl21 |
| Paqr4 |
| Cd14 |
| Pdxdc1 |
| Nhp2l1 |
| Astn2 |
| Ciz1 |

| |
|---|
| Wtap |
| Adamts1 |
| Ccnyl1 |
| Bcl11a |
| Gpatch8 |
| Frmd5 |
| Ranbp6 |
| Snrnp35 |
| Slc4a7 |
| Cuta |
| Dhfr |
| Slc17a8 |
| Tle3 |
| Ppp1r18 |
| Pigu |
| Dcun1d1 |
| Fxr2 |
| Adi1 |
| Nrip2 |
| Lamtor2 |
| Wwp2 |
| Gc |
| Mkl1 |
| Rbm6 |
| Gigyf1 |
| Hrg |
| Aqp1 |
| Tmem201 |
| Krt83 |
| Gga1 |
| Rpl28 |
| Mic1 |
| Eps8l2 |
| Adhfe1 |
| Ppil1 |
| Zfp37 |
| Elmsan1 |
| Ccdc43 |
| Nsg2 |
| Ubash3b |
| Hist1h2bf |
| Tyw1 |
| Cep55 |

| |
|---|
| Ube2z |
| Ca8 |
| Cnep1r1 |
| Gramd3 |
| Tbca |
| Fam172a |
| Gtf2h4 |
| Tnrc6b |
| Dmd |
| Synm |
| Lmbrd1 |
| Abcf1 |
| Txndc17 |
| Trip12 |
| Ttc30b |
| Mpdu1 |
| Srp9 |
| Soga1 |
| Znf281 |
| Gm16286;Naa11 |
| Sepw1 |
| Lchn |
| Atm |
| Chrm1 |
| Lace1 |
| Atg12 |
| Lynx1 |
| Zmym4 |
| Specc1l |
| Folh1 |
| Zak |
| Eml4 |
| Hdac4 |
| Sike1 |
| Spen |
| Trex1 |
| Rtn4r |
| Kank3 |
| Med24 |
| Tmem57 |
| Rabgap1l |
| Rab11fip5 |
| Aasdhppt |

| |
|---|
| Usp18 |
| Ndufab1 |
| Pfdn6 |
| Pik3ip1 |
| Timm10 |
| Sirt6 |
| Ankrd46 |
| Map7 |
| Rgma |
| Hcn4 |
| Txnl4a |
| Cln6 |
| Polr3a |
| Foxo3 |
| Plod3 |
| Pcdh19 |
| Bbs5 |
| Prdm10 |
| Isy1 |
| Epb41l5 |
| Ankrd24 |
| Tmem237 |
| Prickle1 |
| Thsd4 |
| Kif11 |
| Ascc2 |
| Lsm8 |
| Uap1l1 |
| Pvalb |
| Rbm27 |
| Isca1;AK157302 |
| Chac2 |
| Ubp1 |
| Cfap20 |
| Tor1b |
| Cnst |
| Arid5b |
| 04-Sep |
| Kndc1 |
| 10-Sep |
| Dtd2 |
| Fbxo18 |
| Inmt |

| |
|---|
| Drg2 |
| Grb10 |
| Poglut1 |
| Psmb9 |
| Usmg5 |
| Ifit2 |
| Ddx47 |
| Kcnj13 |
| Coa5 |
| Gramd1b |
| Timm17b |
| Pipox |
| Fem1b |
| Rcbtb1 |
| BC068157 |
| Pds5a |
| Igtp |
| Kctd13 |
| Stambp |
| Ccdc92 |
| Grn |
| Ap1s2 |
| Cstf1 |
| Slc24a2 |
| Lmo7 |
| Itga2 |
| Tlk2 |
| Commd9 |
| Safb2 |
| Fam208a |
| Gmpr2 |
| Nxf1 |
| Eif2ak2 |
| BC048403 |
| Rbfox1 |
| Vhl |
| Nbeal2 |
| Asah2 |
| Thrsp |
| Cnot6l |
| Mast4 |
| Zranb2 |
| Mtco2;mt-Co2 |

| |
|---|
| 0610037L13Rik |
| S100a5 |
| Kcnj9 |
| Phf20l1 |
| Mgst1 |
| Sp100 |
| Aes |
| Mcrs1 |
| Igfbp2 |
| Jcad |
| Uggt2 |
| Clic6 |
| Nob1 |
| Cyth3 |
| Ispd |
| Pcyt1a |
| Nrn1 |
| Gpx3 |
| Mrps15 |
| Ecm29;AI314180 |
| Dynlt1 |
| Sf3b3 |
| Sult1d1 |
| Ccbl1 |
| Ube2d3 |
| Smpd4 |
| Tox4 |
| Lama2 |
| Wdr6 |
| Slc25a18 |
| Nelfe |
| Iqgap2 |
| Gnptg |
| Radil |
| Snx11 |
| Hoxd10 |
| B3gnt2 |
| Cilp |
| Kdm5b |
| Arpp19 |
| Sez6l2 |
| Ppp1r3f |
| Cbl |

| | |
|---|---|
| Srrt<br>Ahnak<br>Guf1<br>Csnk1g2<br>Trim46<br>Tbc1d10a<br>Slc35e1<br>Zbtb18<br>Atp13a3<br>Dcaf5<br>Fbxl17<br>Setdb1<br>Crip1<br>Mrpl46<br>Lpcat2<br>Aars<br>Dnajc16<br>Mrpl45<br>Spire1<br>Pik3cd<br>Mib2<br>Tenc1<br>Abhd5<br>Plekhg1<br>Dnmt3a<br>Ttc26<br>Nrf1<br>Rps6ka5<br>Dcp2<br>Dhx36<br>Fyco1<br>Polr1b<br>Rltpr<br>Nlrx1<br>Srsf10<br>Znf830<br>Mrps5<br>Sec23ip<br>Gosr1<br>Psmg3<br>Ogfrl1<br>H2bc3 | |
| Striatum enriched | |

| Common | Mouse Proteome Residual | DA-FASS Proteome Residual |
|---|---|---|
| Tmod1 | B9d2 | Rab4a |
| Smpd3 | Gdpd2 | Abi2 |
| Necab2 | Ercc4 | Ndufa5 |
| Dlgap3 | 03-Sep | Dmxl1 |
| Camk4 | Mlf2 | Clvs2 |
| Spata2l | Ppp1r9a | Ube2l3 |
| Scn4b | Nexn | Ruvbl2 |
| Cacng4 | St8sia3 | Lingo1 |
| Dpp6 | Wdr17 | Nt5dc3 |
| Rasgrp2 | Alg1 | Shroom2 |
| Gprin3 | Kcna4 | H2bc3 |
| Sh2d5 | Rps6ka4 | Arpc1a |
| Syt17 | Mpped2 | Gldc |
| Cacna2d3 | Znf365 | Mcu |
| Akap5 | Mrps14 | Gstm2 |
| Phactr1 | 2010300C02Rik | Gphn |
| Ankrd63 | Ept1 | C2cd4c |
| Dgkb | Asic4 | Psma4 |
| Icam5 | Atf2 | Ppm1e |
| Chrm4 | Rgs7bp | Mrps22 |
| Me3 | Clip4 | Tmed10 |
| Dlgap2 | Pomk | Stxbp5l |
| Mff | Dtnbp1 | Capzb |
| Gnal | Mst4;2610018G03Rik | Cyb5r3 |
| Ptpn5 | Ext2 | Tbc1d24 |
| Plxna2 | Cacnb2 | Septin9 |
| Chat | Kbtbd3 | Otub1 |
| Cbr3 | Mtatp8;mt-Atp8 | Syt2 |
| Shank3 | Ace | Snap91 |
| Rgs9 | Hist2h2ac;Hist2h2aa1;Hist1h2al | Ppia |
| Stk32c | Slc35f3 | Blvrb |
| Cyld | Pde8b | Rap1a |
| Shisa7 | Arpp21 | Hsd17b8 |
| Fbxl16 | Penk | Sar1b |
| Lrrc7 | Foxp1 | Sarm1 |
| Rgs20 | Tesk1 | Astn1 |
| Elmod1 | Pes1 | Ak2 |
| Flrt3 | Inf2 | Dctn3 |
| Itpka | Ckap2l | Plppr1 |
| Tiam2 | Psd | Olfm3 |
| Pde2a | Myl4 | Ret |
| Gpm6b | Bcl11b | Faf2 |

| | | |
|---|---|---|
| Smpdl3b | Krit1 | Hsd17b4 |
| Cyp2s1 | Tspan9 | Actn4 |
| Ngef | Synpo2 | Ppp5c |
| Pde10a | Rasgef1a | Dcc |
| Synpo | Slc7a7 | Rhob |
| Baiap2 | Mesdc1 | Psmd11 |
| Grm2 | Impdh1 | Ermp1 |
| Pcbp2 | Pcdhga9 | Psph |
| Hpca | Rem2 | Glo1 |
| Sipa1l1 | 4933434E20Rik | Tigar |
| Strip2 | Fcho1 | Ntrk2 |
| Rap1gap | Lhx2 | Dhrs7b |
| Actn2 | Trim27 | Slc38a1 |
| Pde1b | Sez6 | Gpr37l1 |
| Anks1b | Sptlc1 | Trappc3 |
| Lzts3 | Nsg2 | Tom1l2 |
| Rps6ka2 | Mapk4 | Arih1 |
| Slc30a3 | Ccm2 | Slc17a6 |
| Ache | Entpd3 | Gad1 |
| Kcnj4 | Nucb2 | Omg |
| Prkcd | Adamts5 | Yars1 |
| Rcn1 | Srgap1 | Gss |
| Gng7 | Lmo7 | Impdh2 |
| Ablim2 | Jcad | Atp1a1 |
| Rgs14 | Mast3 | Etfa |
| Rin1 | Radil | Immt |
| Ptk2b | Sgpp1 | Pank4 |
| Gabrd | | Tmem33 |
| Cacna1e | | Tprg1l |
| Dnm1 | | Cend1 |
| Gls | | Arf2 |
| Slc6a3 | | Pmm1 |
| Syngap1 | | Pithd1 |
| Grm5 | | Dnajc11 |
| Gsg1l | | Stk24 |
| Th | | Vps4b |
| Adcy5 | | Rab9b |
| Ppp1r1b | | Slc30a1 |
| Slc18a2 | | Prkag2 |
| Kcnip2 | | Stt3a |
| Rgs8 | | Nrcam |
| Kcnab1 | | Vps53 |
| Ddc | | Lypla1 |

| | |
|---|---|
| Cpne5 | Txndc5 |
| Crym | H2az1 |
| Rasal1 | Ttc39c |
| Homer1 | Camk1d |
| | Psmf1 |
| | Nos1 |
| | Letmd1 |
| | Cdk5 |
| | Sod2 |
| | Cct2 |
| | Adam23 |
| | Idh3g |
| | Pick1 |
| | Strn4 |
| | Tnpo1 |
| | Cox4i1 |
| | Atp6v1e1 |
| | Chchd6 |
| | Cds2 |
| | Calb2 |
| | Spag9 |
| | Drd1 |
| | Asap1 |
| | Txnrd2 |
| | Arpc5l |
| | Grid1 |
| | Gabra1 |
| | Kcnc1 |
| | Rac3 |
| | Eef1g |
| | Arl8b |
| | Vamp2 |
| | Cdh11 |
| | Rab12 |
| | Mindy2 |
| | Pfkl |
| | Pacsin2 |
| | Idi1 |
| | Hepacam |
| | Napb |
| | Mpp2 |
| | Sec13 |
| | Plp1 |

| |
|---|
| Ndufs4 |
| Ldhb |
| Clint1 |
| Vcan |
| Tst |
| Hnrnpk |
| Paip1 |
| Nln |
| Stx8 |
| Mdp1 |
| Cystm1 |
| Sv2c |
| Dcaf7 |
| Pdia3 |
| Capns1 |
| Syn1 |
| Cadm4 |
| Pi4k2a |
| Doc2a |
| Aimp1 |
| Gnaq |
| Dock3 |
| Unc13a |
| Pip4k2b |
| Rab3a |
| Ywhae |
| Atp6v0a4 |
| Magi1 |
| Gbe1 |
| Aida |
| Sord |
| Macf1 |
| Ctbp2 |
| Fahd1 |
| Lyst |
| Ap1s1 |
| Bcl2l1 |
| Rab3gap1 |
| Ptprs |
| Myo5a |
| Gprc5b |
| Arc |
| Lta4h |

| |
|---|
| Zdhhc5 |
| Rab11b |
| Psmd2 |
| C1qbp |
| Cpd |
| Eef2 |
| Kcnip4 |
| H2aj |
| Ubqln1 |
| Agpat3 |
| Gmfb |
| Iqsec1 |
| Eef1d |
| Atp6ap2 |
| Nudc |
| Cryl1 |
| Ipo5 |
| Usp46 |
| Lrba |
| Ndufaf2 |
| Gpsm1 |
| Ppp2ca |
| Ocrl |
| Pi4ka |
| Srp54 |
| Eif2s3x |
| Rock2 |
| Fech |
| Crybb1 |
| Ckb |
| Atp5pd |
| Snx7 |
| Tpm3 |
| Rabggta |
| Emd |
| Eif2s1 |
| Ndufb3 |
| Dab2ip |
| Plcl1 |
| Wasl |
| Akt3 |
| Tomm40 |
| Cct6a |

| | |
|---|---|
| | Apool |
| | Cpne7 |
| | Pebp1 |
| | Uba5 |
| | Ppfia3 |
| | Ube2m |
| | Eef1a1 |
| | Ctnna2 |
| | Tbc1d17 |
| | Hba |
| | Arl6ip1 |
| | Cops4 |
| | Dsg1a |
| | Arfgef3 |
| | Hnrnph1 |
| | Dst |
| | Apeh |
| | Bcat1 |
| | Tecpr1 |
| | Mboat7 |
| | Gria1 |
| | Ndel1 |
| | Scamp1 |
| | Ppm1h |
| | Ran |
| | Psmc4 |
| | Cadm1 |
| | Vapb |
| | Trnt1 |
| | Mdga2 |
| | Plxna1 |
| | Grm7 |
| | Asl |
| | Rundc3a |
| | Acbd6 |
| | Ttc7b |
| | Tars2 |
| | Coro2b |
| | Aldh18a1 |
| | Grik3 |
| | Ddx3x |
| | Slc3a2 |
| | H2bc1 |

| |
|---|
| Stxbp5 |
| Crtac1 |
| Uqcrb |
| Ppm1a |
| Hprt1 |
| Gcdh |
| Ddah2 |
| Calr |
| Hbb-b1 |
| Mark2 |
| Wasf3 |
| Mlycd |
| Flii |
| Snap29 |
| Nrxn3 |
| Pls3 |
| Csnk2b |
| Efnb3 |
| Atic |
| Lrfn4 |
| Cdk16 |
| Ppid |
| Abhd3 |
| Slc12a5 |
| Begain |
| Suclg1 |
| Prickle2 |
| Ykt6 |
| Zer1 |
| Dlgap4 |
| Atp1b1 |
| Map2 |
| Xkr4 |
| Scn1a |
| Acot10 |
| H2bc14 |
| Vps18 |
| Sh3glb1 |
| Vps11 |
| Actc1 |
| Vcp |
| Mthfd1l |
| Cacna1b |

| | |
|---|---|
| | Idh2 |
| | Anxa7 |
| | Katnal1 |
| | Fam171a2 |
| | Selenoi |
| | Purb |
| | Kctd16 |
| | Vps13c |
| | Htt |
| | Got1 |
| | Rpn1 |
| | Tbcel |
| | Atp2a1 |
| | Grin2b |
| | Fasn |
| | Dpp10 |
| | Gak |
| | Psmc6 |
| | Cd38 |
| | Wfs1 |
| | Gpd1 |
| | Dhodh |
| | Pgm2 |
| | Ndufs7 |
| | Coq6 |
| | Cacnb3 |
| | Sorcs2 |
| | Mgst3 |
| | Acadsb |
| | Hacd3 |
| | Gk2 |
| | Grm8 |
| | Ndufa9 |
| | Pgs1 |
| | Ppp1cb |
| | Stam |
| | Pag1 |
| | Uchl3 |
| | Osbpl1a |
| | Mrps27 |
| | Crip2 |
| | Cacng2 |
| | Stmn3 |

| |
|---|
| Lpcat4 |
| Slc7a5 |
| Sort1 |
| Nrxn2 |
| Aprt |
| Eif3j1 |
| Cyc1 |
| Septin8 |
| Adrbk2 |
| Tanc2 |
| Scarb2 |
| Mtmr9 |
| Shmt2 |
| Mfsd4a |
| Cd81 |
| Dync1li2 |
| Pip5k1a |
| Ttc9b |
| Prkab2 |
| Lrpprc |
| Cpt1a |
| Atp5f1b |
| Nudt5 |
| Elmo1 |
| Arl6ip5 |
| H2ax |
| Gga3 |
| Pld3 |
| Plppr4 |
| Snta1 |
| Trim3 |
| Iqsec2 |
| Atp5f1c |
| Gdi1 |
| Dcun1d3 |
| Tsr2 |
| Atp2a2 |
| Wdr47 |
| Lrrc15 |
| Ptpn23 |
| Mmut |
| Dctn2 |
| Acad9 |

| |
|---|
| Srprb |
| Atp1a2 |
| Mapre3 |
| Crmp1 |
| Hist2h2aa1; Hist2h2aa2 |
| Ptpn11 |
| Ahcy |
| Ndufs2 |
| Fahd2 |
| Dsp |
| Mtap |
| Capn5 |
| Eloc |
| Fcsk |
| Adgrl2 |
| Gna11 |
| Slc8a2 |
| Hist2h2bb |
| Cnrip1 |
| Cacybp |
| Sirpa |
| H3-5 |
| Tomm22 |
| Ndufb11 |
| Gsk3a |
| Grin1 |
| Gabra5 |
| Sgta |
| Rab8b |
| Uggt1 |
| Ppp3ca |
| Gpx4 |
| Rptor |
| Pof1b |
| Snap47 |
| Atp6v1g2 |
| Syne1 |
| Usp9x |
| Timm9 |
| Ubr4 |
| Cox15 |
| Coro1b |
| Uba1 |

| | |
|---|---|
| | Atp6ap1 |
| | Calcoco1 |
| | Aldoc |
| | Tmem132a |
| | Psma8 |
| | Dpysl5 |
| | Lingo2 |
| | Maip1 |
| | Itgb2 |
| | Ubxn6 |
| | Gstm7 |
| | Katnb1 |
| | Abhd6 |
| | Cdh2 |
| | Vdac2 |
| | Smap1 |
| | Anxa2 |
| | Vps33a |
| | Map6 |
| | Pde4d |
| | Pptc7 |
| | Cc2d1a |
| | Aak1 |
| | Slc25a25 |
| | Dhrs7 |
| | Chmp4b |
| | Vac14 |
| | Gmppb |
| | Rars2 |
| | Copg2 |
| | Eif6 |
| | Vamp1 |
| | Galk1 |
| | Ehd3 |
| | Aplp2 |
| | Rabgap1 |
| | Nsdhl |
| | Mthfd1 |
| | Slc10a4 |
| | Ddt |
| | Cacnb4 |
| | Prrt3 |
| | Bphl |

| | |
|---|---|
| | Psmd5 |
| | Relch |
| | Dpp3 |
| | Pak3 |
| | Rab22a |
| | Rtn3 |
| | Ggt7 |
| | Pgam5 |
| | Camk2d |
| | Abi1 |
| | Pygb |
| | Cpne2 |
| | Hspa12b |
| | Tenm2 |
| | Nfs1 |
| | Pdlim5 |
| | Dnaja3 |
| | Ndufa2 |
| | Nedd4l |
| | Pycr3 |
| | Mri1 |
| | Snx30 |
| | Ankrd34b |
| | Tuba3a; Tuba3b |
| | Shisa4 |
| | Cdh10 |
| | Aldh1l2 |
| | Csde1 |
| | Arhgap21 |
| | Acacb |
| | Acsf3 |
| | Shank2 |
| | Pak1 |
| | Cyfip2 |
| | Acad8 |
| | Pdk2 |
| | Naca |
| | Kcnj10 |
| | Pgrmc2 |
| | Poldip2 |
| | Tln1 |
| | Epdr1 |
| | Ubqln2 |

| | |
|---|---|
| | Asrgl1 |
| | Tsg101 |
| | Cct7 |
| | Slc7a10 |
| | Copb1 |
| | Robo2 |
| | Fyn |
| | Cmtm4 |
| | Ca4 |
| | Kirrel3 |
| | Slc9a7 |
| | Cops5 |
| | Dmwd |
| | Syn2 |
| | Vat1 |
| | Dlg2 |
| | Cadps |
| | Cacna2d1 |
| | Acta2 |
| | Aifm1 |
| | Tmem121b |
| | Apoo |
| | Numb |
| | P2ry12 |
| | Necap1 |
| | Lman2 |
| | Adora2a |
| | Ncstn |
| | Dtymk |
| | Rmnd5a |
| | Grm1 |
| | Pik3r1 |
| | Scamp5 |
| | Syt5 |
| | Acot1 |
| | Pccb |
| | Fabp5 |
| | Ppfia2 |
| | Pfkp |
| | Tubb3 |
| | Dtd1 |
| | Clptm1 |
| | Gpm6a |

| |
|---|
| Nisch |
| Mpp6 |
| Rcan1 |
| Pmpca |
| Nt5c |
| Eci1 |
| Asah1 |
| Galc |
| Kcnma1 |
| Vps50 |
| Stx6 |
| Gnb5 |
| Afdn |
| Tgm1 |
| Eif3l |
| Calm2 |
| Snx4 |
| Pcyt2 |
| Dock10 |
| Ajm1 |
| Slc2a13 |
| Palm2 |
| Ampd3 |
| Unc13b |
| Rab15 |
| Actbl2 |
| Rab30 |
| Nck1 |
| Wnk2 |
| Lcp1 |
| Plxnb1 |
| Txnl1 |
| Nipsnap3b |
| Arfgap1 |
| Snx27 |
| Atp5md |
| Map2k1 |
| Tubg2 |
| Slc6a9 |
| Gsdma3 |
| Pacs2 |
| Ncan |
| Arpc3 |

| | |
|---|---|
| | Lrrtm2 |
| | Cntn4 |
| | Kctd12 |
| | Scn2b |
| | Atp6v1f |
| | Gabrb1 |
| | Surf1 |
| | Slc25a5 |
| | Micu3 |
| | Actn1 |
| | Kbtbd11 |
| | Wdr7 |
| | Sdr9c7 |
| | Clcn4 |
| | Adprs |
| | Rhoa |
| | Rtcb |
| | Appl2 |
| | Ndrg4 |
| | Hace1 |
| | Arg1 |
| | Mink1 |
| | Serpinb1a |
| | Acaa1b |
| | Ndrg2 |
| | Dnajb1 |
| | Ryr2 |
| | Cand1 |
| | Hspa5 |
| | Atg5 |
| | Abat |
| | Skt |
| | Rims1 |
| | Strn3 |
| | Rpl13 |
| | Tuba1a |
| | Slc25a10 |
| | Mlst8 |
| | Gps1 |
| | Psmc3 |
| | Gng3 |
| | Ogt |
| | Rabggtb |

| | |
|---|---|
| | Prrt2 |
| | Slc12a6 |
| | Pdk3 |
| | G6pdx |
| | Cct3 |
| | Calb1 |
| | Chn1 |
| | Pgm2l1 |
| | Sh3gl2 |
| | Mapt |
| | GOLGA7B |
| | Cndp2 |
| | Palm |
| | Atl1 |
| | Isoc1 |
| | Adh5 |
| | Elob |
| | C1ql3 |
| | Cryz |
| | Bcap29 |
| | Agpat4 |
| | Rmnd1 |
| | Rars1 |
| | Nptx1 |
| | Rad23b |
| | Rab18 |
| | Psmb7 |
| | Ppa2 |
| | Txnrd1 |
| | Tubb4a |
| | Nccrp1 |
| | Atp5pb |
| | Hsdl2 |
| | Pigs |
| | Slc1a3 |
| | Cacng3 |
| | Sv2b |
| | Dclk1 |
| | Hsp90ab1 |
| | Tomm20 |
| | Pdha1 |
| | Mtor |
| | Bcap31 |

| | |
|---|---|
| | Arfip2 |
| | Tcp1 |
| | Ephx1 |
| | Glul |
| | Tollip |
| | Rab3b |
| | Mpp3 |
| | Lrrc4b |
| | Mtatp6 |
| | Vcpip1 |
| | Slc9a1 |
| | Arhgap23 |
| | Emc1 |
| | Atp6v1e2 |
| | Arl8a |
| | Fmnl1 |
| | Adam22 |
| | Tiprl |
| | L2hgdh |
| | Epb41l3 |
| | Stat1 |
| | Vps26b |
| | Psma3 |
| | Mydgf |
| | Znrd2 |
| | Scai |
| | Vps29 |
| | Dagla |
| | Rab6b |
| | Vps4a |
| | Prps1 |
| | Ppp2r1a |
| | Nedd4 |
| | Mrps23 |
| | Rps5 |
| | Gspt2 |
| | Clip1 |
| | Tmem63b |
| | Pde1a |
| | Nudt10 |
| | Ndufa6 |
| | Diras1 |
| | Sik3 |

| |
|---|
| Cyria |
| Rdx |
| Nbea |
| Acot9 |
| Syngr1 |
| Bcl2l13 |
| Sncg |
| Rab27b |
| Vamp7 |
| Blmh |
| Slc24a4 |
| Pgm3 |
| Pdcd10 |
| Syt3 |
| Gpr158 |
| Carm1 |
| Gsr |
| Hsd17b10 |
| Trappc4 |
| Kif21a |
| Cdipt |
| Wipf3 |
| Sult4a1 |
| Fkbp8 |
| Adgrb1 |
| Acsl3 |
| Rnf214 |
| Gas7 |
| Cntn1 |
| Itgb8 |
| Abhd17a |
| Ndufc2 |
| Ak1 |
| Cct6b |
| Nebl |
| Mark1 |
| Slc32a1 |
| Gprin1 |
| Xpo7 |
| Git2 |
| Pgbd5 |
| Ndufb10 |
| Slc25a42 |

| | |
|---|---|
| | Nefh |
| | Mat2b |
| | Aldh1b1 |
| | Tjp1 |
| | Gstp2 |
| | Cdc37 |
| | Cct8 |
| | Svop |
| | Mical3 |
| | Mrs2 |
| | Ndufb4 |
| | Hsd17b13 |
| | Pdhx |
| | Aldh1a1 |
| | Mapk8 |
| | Grin2a |
| | Rpn2 |
| | Aldh1a7 |
| | Nt5e |
| | Gapdh |
| | Eif5a |
| | Camk2a |
| | Tgm3 |
| | Ech1 |
| | Nme2 |
| | Cplx2 |
| | Fam136a |
| | Gfra2 |
| | Traf3 |
| | Abcb8 |
| | Akr1b7 |
| | Lrp1b |
| | Slc1a2 |
| | Mag |
| | Hcn2 |
| | Cox5a |
| | Armc8 |
| | Gucy1a1 |
| | Gdpd1 |
| | Tmem143 |
| | RTRAF |
| | Rps2 |
| | Synj2bp |

| |
|---|
| Rogdi |
| Septin11 |
| Glrx3 |
| Cacng8 |
| Psmb6 |
| Hagh |
| Sucla2 |
| Cyp46a1 |
| Mdh1 |
| Mapre2 |
| Mapre1 |
| Ttyh3 |
| Gsdma2 |
| Uqcr10 |
| Tenm1 |
| Cpt2 |
| Rimbp2 |
| Atp2b2 |
| Map4 |
| Vps35 |
| H2bc11; H2bc13; H2bc15; H2bc7 |
| Hars1 |
| Sfxn1 |
| Commd3 |
| Cox6c |
| Bin1 |
| Cfl2 |
| Csmd1 |
| Rap2b |
| Gsta4 |
| Sh3glb2 |
| Srgap2 |
| Wars1 |
| Kif2a |
| Sgsm1 |
| H2bc12 |
| Pip5k1c |
| Arrb1 |
| Arhgdia |
| Suclg2 |
| Slc6a11 |
| Ociad1 |
| Septin5 |

| | |
|---|---|
| | Myo6 |
| | Epn2 |
| | Ppp2r2c |
| | Ass1 |
| | Timm23 |
| | Cpe |
| | Arl15 |
| | Pdyn |
| | Hecw2 |
| | Afg3l2 |
| | Napg |
| | Aifm3 |
| | Gapdhs |
| | Ubtd1 |
| | Spr |
| | Ppox |
| | Actr1a |
| | Paics |
| | Irgm1 |
| | Rab11a |
| | Gnas |
| | Jup |
| | Rtn1 |
| | Gja1 |
| | Dgke |
| | Erlin2 |
| | Myh9 |
| | Lysmd2 |
| | Dap3 |
| | Akr1e2 |
| | Gpsm2 |
| | Prdx3 |
| | Tmem256 |
| | Lancl1 |
| | Gde1 |
| | Fermt2 |
| | Slc30a9 |
| | Ap2a1 |
| | Ppp2r5c |
| | Adss2 |
| | Ntng2 |
| | Smyd5 |
| | Ppp6c |

| |
|---|
| Adprh |
| Eef1a2 |
| Mfsd6 |
| Prkar2a |
| Itgam |
| Slc6a6 |
| Ndufaf3 |
| Coa3 |
| Myl12b |
| Hnrnpa2b1 |
| Rab33b |
| Gabra3 |
| Ccdc51 |
| Acp6 |
| Rack1 |
| Cops3 |
| Zzef1 |
| Rgs6 |
| Lap3 |
| Acyp1 |
| Capn1 |
| Gria2 |
| Psmd14 |
| Gabra2 |
| D1Pas1 |
| Chordc1 |
| Stoml2 |
| Rps6ka1 |
| Tomm40l |
| Cpne8 |
| Prmt8 |
| Hpcal1 |
| Apba1 |
| Ap1m2 |
| Arf5 |
| Ndufb7 |
| Nckap1 |
| Prkcg |
| Dnm3 |
| Acat1 |
| C2cd5 |
| Grb2 |
| Coa7 |

| | |
|---|---|
| | Tpi1 |
| | Tpm2 |
| | Hnrnph2 |
| | Mpc2 |
| | Smcr8 |
| | Dsg1b |
| | Tsn |
| | Ap2s1 |
| | Iglon5 |
| | Kpnb1 |
| | Ahsa1 |
| | Btbd17 |
| | Oga |
| | Cnksr2 |
| | Lrrtm1 |
| | Pip4p2 |
| | Ntrk3 |
| | Rab39b |
| | Atp6v1h |
| | Rhot2 |
| | Slc2a3 |
| | Fth1 |
| | Pkm |
| | Mapk1 |
| | Cmpk1 |
| | Cops8 |
| | Hist1h2af |
| | Arhgef12 |
| | Amph |
| | Nlgn4l |
| | Aldh3a2 |
| | Pdia4 |
| | Stxbp3 |
| | Ttc1 |
| | Nck2 |
| | Nfu1 |
| | Hspa9 |
| | Fbxo3 |
| | Tpd52 |
| | Gmppa |
| | Elfn2 |
| | Clic4 |
| | Prep |

| | |
|---|---|
| | Atg2b |
| | Psmd6 |
| | Ccdc177 |
| | Rab24 |
| | Rnf141 |
| | Ipo4 |
| | Abr |
| | Cdk14 |
| | Slc6a17 |
| | Il1rapl1 |
| | Trpv2 |
| | Slc25a20 |
| | Dnaja2 |
| | Cnnm1 |
| | Psma7 |
| | Ppp6r1 |
| | Atp6v1g1 |
| | Sod1 |
| | Pitrm1 |
| | Dbnl |
| | Ppp1ca |
| | Tmem9b |
| | Msn |
| | Adam11 |
| | Fbxo41 |
| | Dnajb4 |
| | Nipsnap2 |
| | Mtco1 |
| | Rab5a |
| | Gabrg2 |
| | Picalm |
| | Slc44a1 |
| | Pura |
| | Irgq |
| | Fam177a1 |
| | Osbpl2 |
| | Dpysl4 |
| | Vdac1 |
| | Gpd2 |
| | Psmc5 |
| | Exoc5 |
| | Dctn1 |
| | Flna |

| |
|---|
| Mfn2 |
| Ptges3 |
| Tenm3 |
| Mccc2 |
| Tmx1 |
| Alg2 |
| Pgam1 |
| Hspd1 |
| Chmp1a |
| Gmps |
| Ctnnd2 |
| Sorcs3 |
| Maob |
| Slmap |
| Soga3 |
| Wbp2 |
| Plxnc1 |
| Msra |
| Tnr |
| Isyna1 |
| Pex5l |
| Negr1 |
| Sri |
| Ddx1 |
| Stim1 |
| Tpbg |
| Grik5 |
| Uchl5 |
| Pitpna |
| Mlc1 |
| Arhgap39 |
| Fgf12 |
| Fibp |
| Stmn2 |
| Ak5 |
| Echdc1 |
| Setd7 |
| Babam1 |
| Akap12 |
| Glod4 |
| Rps6ka3 |
| Kif1b |
| Dync1h1 |

| |
|---|
| Stum |
| Timm13 |
| Mtmr2 |
| Ca2 |
| Mtfp1 |
| Uqcrq |
| Sec23a |
| Rap2a |
| Vps25 |
| Ezr |
| Plaa |
| Dync1li1 |
| Nap1l1 |
| Lin7a |
| Abhd10 |
| Taok1 |
| Tm9sf3 |
| Prnp |
| Rps13 |
| Ctsd |
| Tesc |
| Grpel1 |
| Wdr48 |
| Cox6b1 |
| Actg2 |
| Usp7 |
| Eea1 |
| Acat2 |
| Camkk2 |
| Brinp1 |
| Atp1a3 |
| Tkt |
| Uso1 |
| Trim32 |
| Gdi2 |
| Mrpl12 |
| Pdpk1 |
| Atp5f1d |
| Scfd1 |
| Rap1gap2 |
| Cd47 |
| Ndufs1 |
| Cul2 |

| |
|---|
| Crat |
| Vps13a |
| Timm29 |
| Akr7a2 |
| Ckap4 |
| Nefm |
| Clip2 |
| Slc18a3 |
| Epb41 |
| Fsd1 |
| Atp5mg |
| Ptpn9 |
| Acaca |
| Psmd13 |
| Epha4 |
| Nlgn2 |
| Mtmr1 |
| Ccny |
| Coq3 |
| Sar1a |
| Cadps2 |
| Cisd1 |
| Kif5a |
| Dctn5 |
| Vapa |
| Agk |
| Tubg1 |
| Pip4k2c |
| Exoc8 |
| Thnsl1 |
| Vsnl1 |
| Cdc42 |
| Git1 |
| Acsl6 |
| Gsto1 |
| Opa1 |
| Dbn1 |
| Vps33b |
| Sec61a1 |
| Slc25a3 |
| Ctbp1 |
| Gap43 |
| Prune1 |

| |
|---|
| Erp44 |
| Hsp90aa1 |
| Appl1 |
| Cat |
| Clcn6 |
| Clcn3 |
| Arpc2 |
| Get4 |
| Cpne3 |
| Ndrg3 |
| Slc25a4 |
| Pnp |
| Dip2b |
| Ptk2 |
| Psmb2 |
| Lrpap1 |
| Sh3gl1 |
| Chkb |
| Fabp3 |
| Marchf5 |
| Olfm2 |
| Dlg1 |
| Pgam2 |
| Cops7a |
| Gba |
| Cryzl1 |
| Csrp1 |
| Septin2 |
| Them4 |
| Slc6a1 |
| Rapgef4 |
| Kcnt1 |
| Mat2a |
| Retreg2 |
| Gnb1 |
| Aldh8a1 |
| Rala |
| Ndufs6 |
| Lmna |
| Clic1 |
| Impact |
| Ptges2 |
| Pak2 |

| |
|---|
| Myl6 |
| Cryab |
| Copb2 |
| Tmem30a |
| Sema7a |
| Hint1 |
| Cltc |
| Padi2 |
| Tpd52l2 |
| Psat1 |
| Brsk1 |
| Sgtb |
| Ywhab |
| Ptrh2 |
| Capn2 |
| Timm50 |
| Ide |
| Hdhd3 |
| Lmtk2 |
| Map2k6 |
| Fkbp1a |
| Tppp3 |
| Srm |
| Ube2n |
| Nsfl1c |
| Cyfip1 |
| Mlec |
| Slc12a9 |
| Grm3 |
| Ahcyl1 |
| Thop1 |
| Slc12a2 |
| Cit |
| Sec31a |
| Anxa3 |
| Ctnnb1 |
| Impa1 |
| Farp1 |
| Aldh1l1 |
| Pitpnc1 |
| Jam3 |
| Acvr1b |
| Csnk1e |

| | |
|---|---|
| | Sfxn5 |
| | Plpp3 |
| | Kalrn |
| | Eif4e |
| | Vkorc1l1 |
| | Adk |
| | Ddx19a |
| | Acta1 |
| | Trap1 |
| | Madd |
| | Pcbp1 |
| | Cldn11 |
| | Cacna2d2 |
| | Sdhb |
| | Ncln |
| | Mtpn |
| | Sptan1 |
| | Selenbp1 |
| | Aldh5a1 |
| | Sugt1 |
| | Tpt1 |
| | H2bc4; H2bc6; H2bc8 |
| | Hint2 |
| | Osbp |
| | Plxnb2 |
| | Acadm |
| | Hint3 |
| | Pabpc1 |
| | Gnl1 |
| | Igbp1 |
| | Nagk |
| | Vps26a |
| | Bcas3 |
| | Hspa12a |
| | Ssr1 |
| | Dlat |
| | Gpr52 |
| | Micu1 |
| | Cd200 |
| | Pacs1 |
| | Strap |
| | Inpp5a |
| | Rab3gap2 |

| | |
|---|---|
| | Srpk2 |
| | Camkv |
| | Dgki |
| | Mtnd4 |
| | Iqsec3 |
| | Gba2 |
| | H3-3a; H3-3b |
| | Arcn1 |
| | Arfgef1 |
| | Psma2 |
| | Lancl2 |
| | Ttpal |
| | Unc13c |
| | Sh3kbp1 |
| | Mmab |
| | Aldh7a1 |
| | Prkar2b |
| | Slk |
| | Atp2a3 |
| | Wdr45 |
| | Lgalsl |
| | Dnpep |
| | Rab3c |
| | Bcr |
| | Dld |
| | Nit1 |
| | Ggct |
| | Mtx1 |
| | Plscr3 |
| | Ndrg1 |
| | Tab1 |
| | Eps15l1 |
| | Cyb5r1 |
| | Fbxo2 |
| | Eipr1 |
| | Slc1a6 |
| | Lrrc57 |
| | Prpsap2 |
| | Camk2b |
| | Inpp4a |
| | Iqcb1 |
| | Atp6v0a1 |
| | Lrp1 |

| |
|---|
| Islr2 |
| Ina |
| Gabrb2 |
| Stx16 |
| Bmerb1 |
| Serpinb6 |
| Dsg1c |
| Tagln2 |
| Xpo1 |
| Ist1 |
| Coro1a |
| Arf1 |
| Sco1 |
| Rnh1 |
| Calml3 |
| Bmpr2 |
| Hadh |
| Mrps31 |
| Atad3 |
| Srcin1 |
| Epm2aip1 |
| Atp5f1e |
| Atp6v1d |
| Pafah1b3 |
| Pepd |
| Stx4 |
| Pdpr |
| Septin4 |
| Ppa1 |
| Nme1 |
| Abcg2 |
| Slc25a11 |
| Ap2m1 |
| Pdhb |
| Lmbrd2 |
| Rftn2 |
| Acadvl |
| Ndufb8 |
| Snx2 |
| Uqcc1 |
| Add3 |
| Caskin1 |
| Arl1 |

| | |
|---|---|
| | Wdr54 |
| | Synrg |
| | Mtfr1l |
| | Cfl1 |
| | Uqcrc2 |
| | Tmem65 |
| | C2cd2l |
| | Tmx4 |
| | Acaa1a |
| | Uqcrfs1 |
| | Asphd2 |
| | Itpr1 |
| | Psmc2 |
| | Atp2b1 |
| | Ppib |
| | Gabarapl2 |
| | Naxe |
| | Pitpnm1 |
| | Pik3c3 |
| | Dynll1 |
| | Hal |
| | Rad23a |
| | Mdh2 |
| | Adcy9 |
| | Ntng1 |
| | Rtn4 |
| | Cep170 |
| | Fhl1 |
| | Afg3l1 |
| | Abcb9 |
| | Mccc1 |
| | Capza2 |
| | Hars2 |
| | Blvra |
| | Sacm1l |
| | Gfm1 |
| | Rcn2 |
| | Pls1 |
| | Erp29 |
| | Prdx2 |
| | Lysmd1 |
| | Cops6 |
| | Ube2k |

| | |
|---|---|
| | Slc7a2 |
| | Atp9a |
| | Marcks |
| | Hsdl1 |
| | Pmpcb |
| | Rnpep |
| | Gpr88 |
| | Epn1 |
| | Scn3b |
| | Atp1b3 |
| | Ncs1 |
| | Mapk8ip3 |
| | Psme1 |
| | Tenm4 |
| | Sdhd |
| | Crkl |
| | Map1b |
| | H3c1; H3c10; H3c11; H3c8 |
| | Prmt1 |
| | Por |
| | Kcna2 |
| | Aqp4 |
| | Sars1 |
| | Septin6 |
| | Cnr1 |
| | Ndufa7 |
| | Opa3 |
| | Aspa |
| | Lamtor1 |
| | Rasl2-9 |
| | Scp2 |
| | Nol3 |
| | Rpsa |
| | Bsn |
| | Mapk3 |
| | Vgf |
| | Lrtm2 |
| | Dync1i2 |
| | Rab9a |
| | Hsp90b1 |
| | Tbcb |
| | Oplah |
| | Ap2a2 |

| | |
|---|---|
| | Cbr4 |
| | Dnajc5 |
| | Cap1 |
| | Hk2 |
| | Stub1 |
| | Ptcd3 |
| | Plcb1 |
| | Srgap3 |
| | Cspg5 |
| | Gk |
| | Rab5c |
| | Ppp3r1 |
| | Rab6a |
| | Ogdh |
| | Reps1 |
| | Lgi1 |
| | Igsf8 |
| | Arf4 |
| | Rplp0 |
| | Pcyox1 |
| | Diras2 |
| | Ivd |
| | Rabgef1 |
| | Gstk1 |
| | Atp5po |
| | Itgav |
| | Tsfm |
| | Pik3r4 |
| | Agap3 |
| | Dhrs4 |
| | Araf |
| | Ncdn |
| | Vim |
| | Col1a2 |
| | Prodh |
| | Rabep1 |
| | Tamm41 |
| | Pdcd6ip |
| | Aloxe3 |
| | Slc38a3 |
| | Pck2 |
| | Flot2 |
| | Tuba4a |

| | |
|---|---|
| | Gnai3 |
| | Slc4a3 |
| | Emc2 |
| | Psmb1 |
| | Synpr |
| | Tmx3 |
| | Efr3b |
| | Prkar1b |
| | Rhot1 |
| | Agap1 |
| | Hyou1 |
| | Nsf |
| | Acot11 |
| | Cpm |
| | Psmb5 |
| | Daam1 |
| | Dtnb |
| | Tm9sf2 |
| | Atg16l1 |
| | Wdr77 |
| | Rab10 |
| | Aars1 |
| | Twf2 |
| | Slc25a13 |
| | Dnajb2 |
| | Idh3a |
| | Prkar1a |
| | Gch1 |
| | Neto2 |
| | Ryr3 |
| | Arhgef7 |
| | Kiaa1549 |
| | Prxl2b |
| | Ftl1 |
| | Synj1 |
| | Hpcal4 |
| | Eif4b |
| | Aco1 |
| | Fam126b |
| | Snca |
| | Taldo1 |
| | Aco2 |
| | Maoa |

| |
|---|
| Hspa4 |
| Map1lc3a |
| Asic1 |
| Nrbp2 |
| Cct4 |
| Vps51 |
| Gstm5 |
| Slc22a23 |
| Glrx5 |
| Timmdc1 |
| Sv2a |
| Rab23 |
| Ado |
| Pten |
| Glud1 |
| Abhd12 |
| Ndufv1 |
| Cs |
| Psmd7 |
| Ephx2 |
| S1pr1 |
| Chrnb2 |
| Ap1g2 |
| Slc25a23 |
| Cad |
| Actb |
| Pam |
| Rab7a |
| Eno1 |
| Actr2 |
| Prrt1 |
| Aldoa |
| Lin7c |
| Ap3b1 |
| Acot7 |
| Klc2 |
| Mtch1 |
| Phyhipl |
| Pip5k1b |
| Adcy6 |
| Heatr5b |
| Slc4a8 |
| Rac1 |

| |
|---|
| Tom1 |
| Actr10 |
| Mpi |
| Pgls |
| Adam10 |
| Wdr1 |
| Exog |
| Spg7 |
| Thy1 |
| Akap7 |
| Nptxr |
| Myo18a |
| Cntnap2 |
| Ndufa13 |
| Vat1l |
| Bdh1 |
| Entpd2 |
| Npepps |
| Arl2 |
| Wdr37 |
| Agpat5 |
| Ccdc136 |
| Tpd52l1 |
| Myadm |
| Ndufa4 |
| Hibadh |
| Osbpl10 |
| Psmb4 |
| Cpne9 |
| Eef1b |
| Ap1g1 |
| Hccs |
| Memo1 |
| Arhgef6 |
| Tfrc |
| Ank2 |
| Fxyd6 |
| Ptprn2 |
| Alcam |
| Ap3m2 |
| Anxa4 |
| Plcg1 |
| Cacna1a |

| |
|---|
| Snx1 |
| Lamp2 |
| Ap1m1 |
| L1cam |
| Usp15 |
| Eci3 |
| Eml5 |
| Sorbs2 |
| Tars1 |
| Kiaa0513 |
| Rapgef2 |
| Ube2v2 |
| Klc1 |
| Mpst |
| Spcs2 |
| Gmpr |
| Cops2 |
| Sec22b |
| Arhgap32 |
| Csnk2a1 |
| Gatd3a |
| Psmd1 |
| Ube2o |
| Dlg4 |
| Hip1 |
| Pfn1 |
| Plcd1 |
| Ube3a |
| Stx1b |
| Stip1 |
| Elmo2 |
| App |
| Gnaz |
| Ndufa11 |
| Dennd11 |
| Ldha |
| Atp6v1a |
| Celsr2 |
| Cul1 |
| Gabbr2 |
| Psmd12 |
| Cnp |
| Ppm1f |

| |
|---|
| Prkcsh |
| Hexb |
| Tmem126a |
| Eif4a1 |
| Napa |
| Ppp1r9b |
| Dynlrb1 |
| Plxnd1 |
| Atp5mf |
| H2ac20 |
| Cct5 |
| Atp2b4 |
| Cul3 |
| Exoc3 |
| Gstm1 |
| Ywhag |
| Fah |
| Ndufb5 |
| Vps52 |
| Basp1 |
| Hip1r |
| Rufy3 |
| Nefl |
| Cap2 |
| Hsd17b12 |
| Lsamp |
| Nif3l1 |
| Dad1 |
| Glg1 |
| Snx6 |
| Phyhip |
| Syt7 |
| Gnb4 |
| Mtnd5 |
| Gdap1 |
| Avl9 |
| Cltb |
| Elmod2 |
| Ppp2r5e |
| Aldh6a1 |
| Fundc2 |
| Src |
| Fkbp4 |

| |
|---|
| Tmem163 |
| Slc25a46 |
| Oxsr1 |
| Cadm3 |
| Tbcd |
| Vwa8 |
| Eif5 |
| Arpc5 |
| Trappc9 |
| Endod1 |
| Rdh14 |
| Prdx6 |
| Me1 |
| Gaa |
| Pc |
| Gabrb3 |
| Gsn |
| Neo1 |
| Crk |
| Atp6v0d1 |
| Xpnpep1 |
| Mras |
| Mt-Cyb |
| Nans |
| Tdrkh |
| Wdr13 |
| Csnk1a1 |
| Sgip1 |
| Becn1 |
| Ehd1 |
| Mapk10 |
| Exoc4 |
| Cpt1c |
| Necab1 |
| Hdhd2 |
| Plec |
| Eif3e |
| Sirt5 |
| M6pr |
| Kcnab2 |
| Ndufs5 |
| Tsc2 |
| Ugp2 |

| | |
|---|---|
| | Plch2 |
| | Dynll2 |
| | Stmn1 |
| | Sigmar1 |
| | Prkaca |
| | Nudt3 |
| | Tomm70 |
| | Sptb |
| | Olfm1 |
| | Slc44a2 |
| | Exoc7 |
| | Septin3 |
| | P4hb |
| | Hspa8 |
| | Plcl2 |
| | Pgrmc1 |
| | Reep5 |
| | Dusp3 |
| | Gpc4 |
| | Pfkm |
| | Pitpnb |
| | Tmem11 |
| | Zfyve1 |
| | Nfasc |
| | Itsn1 |
| | Agpat1 |
| | Kpna3 |
| | Tnpo3 |
| | Fam162a |
| | Kifap3 |
| | Pcyox1l |
| | Atg7 |
| | Kif1a |
| | Mbp |
| | Ckmt1 |
| | Abl2 |
| | Stk11 |
| | Uchl1 |
| | Adgrb3 |
| | Gripap1 |
| | Ak4 |
| | Exoc6b |
| | Sdha |

| |
|---|
| Epb41l2 |
| Slc25a51 |
| Lgals3 |
| Psmd3 |
| Eif5a2 |
| Cycs |
| Kcna6 |
| Nap1l4 |
| Nadk2 |
| Cyb5b |
| Mtco2 |
| Tnik |
| Hspe1 |
| Actr3 |
| Itpa |
| Lasp1 |
| Acsbg1 |
| Nme3 |
| Ciapin1 |
| Gucy1b1 |
| Wdfy3 |
| Cmpk2 |
| Vti1b |
| Htra2 |
| Czib |
| Stx1a |
| Psma6 |
| Tppp |
| Tfam |
| Pcsk2 |
| Serpinb5 |
| Nlgn3 |
| Dpysl3 |
| Cxadr |
| Ppp1r7 |
| Acp1 |
| Bckdk |
| Mtch2 |
| Dars1 |
| Mtnd1 |
| Grk2 |
| Rgs7 |
| Erc1 |

| |
|---|
| Fh |
| Ipo7 |
| Gdpd5 |
| Rab1b |
| Evl |
| Apoe |
| Cntfr |
| Decr1 |
| Cdc42bpa |
| Eps15 |
| Pdxk |
| Pgap1 |
| Strn |
| Agfg1 |
| Ppp6r2 |
| Pgp |
| Ddhd2 |
| Fmnl2 |
| Hspa1a |
| Plcxd3 |
| Dnm1l |
| Acsl5 |
| Lrrc8b |
| Nipsnap1 |
| Myh10 |
| Tubb2a |
| Dbt |
| Armc10 |
| Vps16 |
| Ptpre |
| NARS1 |
| Acly |
| Ckap5 |
| Cpne4 |
| Ppt1 |
| Ptprj |
| Gltp |
| Prkce |
| Rab35 |
| Cdk17 |
| Arhgap44 |
| Sfxn3 |
| Samm50 |

| | |
|---|---|
| | Hgs |
| | Ica1 |
| | Map2k4 |
| | Lnpk |
| | Lpgat1 |
| | Atpaf1 |
| | Slc25a44 |
| | Nlgn1 |
| | Upf1 |
| | H2ac12 |
| | Bles03 |
| | Gnai1 |
| | Naxd |
| | Lrrc4c |
| | Apmap |
| | Gng2 |
| | Hadhb |
| | Ranbp9 |
| | Creld1 |
| | Pafah1b2 |
| | Cask |
| | Daam2 |
| | Man2c1 |
| | Syn3 |
| | Atp5me |
| | Ndufb9 |
| | Etfdh |
| | Gnpda1 |
| | Acox1 |
| | Osbpl8 |
| | Slc7a8 |
| | Gnb2 |
| | Ap3s2 |
| | Grik2 |
| | Fam131b |
| | Zadh2 |
| | Bpnt1 |
| | Capza1 |
| | Csmd3 |
| | Nectin1 |
| | Map1s |
| | Rragd |
| | Ptpra |

| |
|---|
| Alad |
| Ppp2r2a |
| Rnf14 |
| Slc23a2 |
| Atp1b2 |
| Hmgcl |
| S100a11 |
| Anxa6 |
| Scrn1 |
| Srr |
| Pcmt1 |
| Ric8a |
| Cpne1 |
| Rps3 |
| Sdk2 |
| Kif5b |
| Reps2 |
| Etf1 |
| Add2 |
| S100a14 |
| Sfn |
| G6pd2 |
| Eif3i |
| Atp6v1c1 |
| Pclo |
| Pip4k2a |
| Sdhc |
| Nudt16 |
| Vps36 |
| Tspan7 |
| Tmx2 |
| Prdx1 |
| Tcp11l1 |
| Sparcl1 |
| Rab11fip2 |
| Ppp2r1b |
| Rab5b |
| Trappc11 |
| Ptprr |
| Hsph1 |
| Rock1 |
| Dhrs1 |
| Cldnd1 |

| |
|---|
| Selenbp2 |
| Slc25a24 |
| Acsf2 |
| Tagln3 |
| Abcd3 |
| Lrrtm4 |
| Ndufaf1 |
| Anxa1 |
| Acadl |
| Itfg1 |
| Ttyh1 |
| Itgb1 |
| Kctd8 |
| Rhoc |
| Atg9a |
| Plpbp |
| Pycr2 |
| Arfgef2 |
| Clta |
| Prpsap1 |
| Rph3a |
| Ccdc127 |
| Myo5b |
| Adck1 |
| Dnaja4 |
| Clpp |
| Nit2 |
| Anxa11 |
| Kif17 |
| Fscn1 |
| Arf3 |
| St13 |
| Flot1 |
| Slc25a14 |
| Cse1l |
| Fn3k |
| Hspa4l |
| Cpne6 |
| Guk1 |
| Cab39 |
| Bnip3 |
| Gatd1 |
| Ppp1r12a |

| | |
|---|---|
| | Adrm1 |
| | Comtd1 |
| | Ftl2 |
| | Ppp2r5a |
| | Gria4 |
| | Pdk1 |
| | Vwa5a |
| | Gpi |
| | Rras2 |
| | Tmod2 |
| | Chl1 |
| | H3c13; H3c14; H3c15; H3c2; H3c3; H3c4; H3c6; H3c7 |
| | Sec14l2 |
| | Pcca |
| | Atp6v0a2 |
| | Cyrib |
| | Slc17a7 |
| | Dstn |
| | Lonp1 |
| | Mark3 |
| | Vdac3 |
| | Ppm1b |
| | Vars1 |
| | Brsk2 |
| | Sdr39u1 |
| | Ddx3y |
| | Susd2 |
| | Ppp1r21 |
| | Ssr4 |
| | Hspa2 |
| | Ywhaz |
| | Cdc42ep4 |
| | Cacnb1 |
| | H2az2 |
| | Dgkz |
| | Dip2a |
| | Tmed9 |
| | Sncb |
| | Prune2 |
| | Ddost |
| | Ddah1 |
| | Atad1 |
| | Mtx2 |

| |
|---|
| Vamp3 |
| Hadha |
| Emc3 |
| Grhpr |
| Pacsin1 |
| Extl2 |
| Neto1 |
| Myl9 |
| Ergic1 |
| Gclc |
| Dgkq |
| Dmtn |
| Ctnnd1 |
| Auh |
| Trio |
| Cntnap4 |
| Rpl27a |
| Mecr |
| Ptp4a1 |
| Tubb5 |
| Ap3d1 |
| Pnpla8 |
| Mal2 |
| Ralb |
| Fam114a2 |
| Dock4 |
| Mog |
| Ampd2 |
| Dtna |
| Gsk3b |
| Rpl15 |
| Lypla2 |
| Slc25a12 |
| Rdh11 |
| Lamp5 |
| Vta1 |
| Rbbp9 |
| Gna13 |
| Nucb1 |
| Mrpl39 |
| Twf1 |
| Denr |
| Igsf21 |

| |
|---|
| Hibch |
| Kcnd2 |
| Nomo1 |
| Cdip1 |
| Vbp1 |
| Ndufs8 |
| Gstz1 |
| Plxna4 |
| Snx16 |
| Dnajb11 |
| Lin7b |
| Skp1 |
| Pkp1 |
| Fam210a |
| Vcam1 |
| Prdx5 |
| Vti1a |
| Smap2 |
| Spryd7 |
| Trim9 |
| Add1 |
| Atg3 |
| Babam2 |
| Rab4b |
| Eno2 |
| Clasp2 |
| Hcn1 |
| Gsdma |
| Arhgap26 |
| Abcb10 |
| Park7 |
| Wasf1 |
| Bcan |
| Efhd2 |
| Nipa1 |
| Itm2b |
| Slc5a7 |
| Arhgap1 |
| Dctn4 |
| Gad2 |
| Hbb-b2 |
| Plgrkt |
| Map1a |

| |
|---|
| Prkcb |
| Pgd |
| Cul5 |
| Hebp1 |
| Slc4a10 |
| Ndufa8 |
| Rragc |
| Iba57 |
| Ubtd2 |
| Phf24 |
| Acsl1 |
| Dync1i1 |
| Slc8a1 |
| Flad1 |
| Nwd2 |
| Gdap1l1 |
| Epb41l1 |
| Slc9a3r1 |
| Aldh4a1 |
| Ppp2cb |
| Sars2 |
| Ppp3cb |
| Clvs1 |
| Syngr3 |
| Rap1b |
| Camkk1 |
| Fbxl4 |
| Eif3j2 |
| Akr1b1 |
| Atp8a1 |
| Gstm3 |
| Syt1 |
| Acaa2 |
| Tsc1 |
| Phb |
| Sirt2 |
| Agap2 |
| Psmd8 |
| Psma1 |
| Itpr3 |
| Jagn1 |
| Ppp1cc |
| Enpp6 |

| |
|---|
| Tbrg4 |
| Nae1 |
| Slc4a4 |
| Coasy |
| Ubfd1 |
| Mob4 |
| Oat |
| Huwe1 |
| Sbf1 |
| Ddb1 |
| Pgk1 |
| Apbb1 |
| Akr1a1 |
| Ncam1 |
| Ccdc6 |
| Cttnbp2 |
| Oxr1 |
| Prkacb |
| Hmox2 |
| Hspbp1 |
| Ndufs3 |
| Pex14 |
| Trim2 |
| Slc7a14 |
| Gpx1 |
| Ola1 |
| Gpd1l |
| Mblac2 |
| Ap2b1 |
| Gda |
| Dmxl2 |
| Etfb |
| Clu |
| Eif4h |
| Ddi2 |
| H2bc9 |
| Ufc1 |
| Stx12 |
| Ndufb6 |
| Lhfpl4 |
| Eif4a2 |
| Prxl2a |
| Ttc9 |

| |
|---|
| Oscp1 |
| Faah |
| Rhog |
| Exoc2 |
| Prkca |
| Mgll |
| Efr3a |
| Canx |
| Pdcd6 |
| Myh14 |
| Gfod1 |
| Coq5 |
| Slc35f1 |
| Lrrc47 |
| Abraxas2 |
| Ndufa10 |
| Timm44 |
| Rmdn3 |
| Got2 |
| Kif5c |
| Chp1 |
| Vps45 |
| Esd |
| Tpm1 |
| Emb |
| Dlgap1 |
| Acap2 |
| Pdp1 |
| Eppk1 |
| Shank1 |
| Abhd16a |
| Rab14 |
| Bckdha |
| Gabbr1 |
| Eprs1 |
| Unc5a |
| Idh1 |
| Dlst |
| Ly6h |
| Uqcrc1 |
| Me2 |
| Hapln1 |
| Mtatp8 |

| | |
|---|---|
| | Gart |
| | Scn1b |
| | Snx5 |
| | Ehd4 |
| | Ncam2 |
| | Itm2c |
| | Cox5b |
| | Gabra4 |
| | Snap25 |
| | Gfap |
| | Ptprn |
| | Cadm2 |
| | Rpl3 |
| | Hsd17b11 |
| | Cntnap1 |
| | Dglucy |
| | Gmds |
| | Rps9 |
| | Insrr |
| | Acot2 |
| | Arpc4 |
| | Pfn2 |
| | Sh3gl3 |
| | Scn2a |
| | Pgm1 |
| | Csnk2a2 |
| | Fis1 |
| | Prss2 |
| | Fnbp1 |
| | Arf6 |
| | Nrxn1 |
| | Vipas39 |
| | Sptbn1 |
| | Bsg |
| | Dlg3 |
| | Nptn |
| | Psmb3 |
| | Efhd1 |
| | Ermn |
| | Eml2 |
| | Slc1a4 |
| | Camk2g |
| | Ptprf |

| | |
|---|---|
| | Armc1 |
| | Mif |
| | Phgdh |
| | Mrtfb |
| | Fry |
| | Nqo2 |
| | Pdia6 |
| | Glrx |
| | Stk39 |
| | Ank3 |
| | Armc6 |
| | Magi2 |
| | Praf2 |
| | Ralgapa1 |
| | Slc6a7 |
| | Trappc12 |
| | Slc25a1 |
| | Arl3 |
| | Itpr2 |
| | Nceh1 |
| | Tmem35a |
| | Rab2a |
| | Arhgef9 |
| | Plxna3 |
| | Ndufa3 |
| | Bcs1l |
| | Chchd3 |
| | Atcay |
| | Gnao1 |
| | Zmpste24 |
| | H2aw |
| | Nampt |
| | Slc2a1 |
| | Inpp1 |
| | Ptpa |
| | Ccz1 |
| | Gpc1 |
| | Ap3m1 |
| | Faim2 |
| | Aldh9a1 |
| | Ptp4a2 |
| | Atp5f1a |
| | Stx3 |

| | |
|---|---|
| | Cep170b |
| | Atp6v1b2 |
| | Ndufa12 |
| | Eci2 |
| | H2ac7 |
| | Scyl2 |
| | Stx7 |
| | Cttn |
| | Stxbp1 |
| | Spart |
| | Serinc1 |
| | Zc2hc1a |
| | Lxn |
| | Oxct1 |
| | Anxa5 |
| | Pygm |
| | Mtarc2 |
| | Ube2v1 |
| | Sorbs1 |
| | Gapvd1 |
| | Gria3 |
| | Phb2 |
| | Rab1A |
| | Usp24 |
| | Adsl |
| | Wdr44 |
| | F3 |
| | Ndufv2 |
| | Ap3b2 |
| | Taok3 |
| | Psma5 |
| | Rab31 |
| | Ipo9 |
| | Psmd4 |
| | Bsdc1 |
| | Iars2 |
| | Enoph1 |
| | Ndufaf5 |
| | Ranbp1 |
| | Dpysl2 |
| | Cntn2 |
| | Letm1 |
| | Echs1 |

| |
|---|
| Mocs3 |
| Erc2 |
| Tpp2 |
| Slc27a4 |
| Ppp6r3 |
| Fnbp1l |
| Coro1c |
| Pkp4 |
| Ggps1 |
| Gstp1 |
| Cdc42bpb |
| Septin7 |
| Sccpdh |
| Pef1 |
| Cyb5a |
| Frrs1l |
| Clybl |
| Gars1 |
| Lrrc8a |
| Get3 |
| Bag6 |
| Kcna1 |
| Ppme1 |
| Tubgcp2 |
| F8a1 |
| Rab8a |
| Shisa9 |
| Them6 |
| Pin1 |
| Fmnl3 |
| Dock9 |
| Mcts1 |
| Slc25a22 |
| Brcc3 |
| Hspa1b |
| Dnajb6 |
| Nt5c3a |
| Tubb2b |
| Hk1 |
| Ptprd |
| Lrrtm3 |
| Psd3 |
| Kazn |

| | |
|---|---|
| | Psmd9 |
| | Scamp3 |
| | Gnai2 |
| | Gspt1 |
| | Numbl |
| | Nf1 |
| | Uba1y |
| | Adgrl1 |
| | Wwox |
| | Slc16a1 |
| | Ruvbl1 |
| | Dnajc6 |
| | H4c1; H4c11; H4c12; H4c14; H4c2; H4c3; H4c4; H4c6; H4c8; H4c9; H4f16; Hist1h4m |
| | Usp14 |
| | Prmt5 |
| | Rab21 |
| | Cdh13 |
| | Ipcef1 |
| | Lactb |
| | Cars1 |
| | Qdpr |
| | Vcl |
| | Tufm |
| | Scrn3 |
| | Fsd1l |
| | Syp |
| | Enah |
| | Pdxp |
| | Rida |
| | Tmem43 |
| | Prkag1 |
| | Rpl11 |
| | Adgrl3 |
| | Ywhaq |
| | Atl2 |
| | Sphk2 |
| | Pcdhga4 |
| | Pafah1b1 |
| | Coq9 |
| | Bag5 |
| | Ap3s1 |
| | Rpl12 |
| | Syt12 |

| | | |
|---|---|---|
| | | H2ac15 |
| | | Sco2 |
| | | Usp5 |
| | | Lmtk3 |
| | | Ncald |
| | | Slc25a40 |
| | | Lamp1 |
| | | Uhrf1bp1l |
| | | Cbr1 |
| | | Aldh2 |
| | | Ywhah |
| | | Nckipsd |
| | | Camsap3 |
| | | Actr1b |
| | | Psmc1 |
| | | Ntm |
| | | Dnaja1 |
| | | Ganab |
| | | Slc29a1 |
| | | Cotl1 |
| | | Hist1h2bp |
| | | Abcb7 |
| | | Ctsb |
| | | Fdps |
| | | Tln2 |
| | | Plppr3 |
| | | Rp2 |
| | | Rheb |
| | | Actr3b |
| | | Ak3 |
| | | Ahcyl2 |
| | | Uchl4 |
| | | Tph2 |
| | | Ckmt2 |
| | | Ap1b1 |
| | | Mpp1 |
| | | Snph |
| | | Fgf14 |
| Primary Cultured Cell | | |
| Common | Mouse Proteome Residual | DA-FASS Proteome Residual |
| Ndufa5 | Sorbs3 | Rab4a |
| Lingo1 | Dcun1d2 | Abi2 |
| Nt5dc3 | Usp20 | Dmxl1 |

| | | |
|---|---|---|
| Gstm2 | Bbs9 | Clvs2 |
| Stxbp5l | Mon1b | Ube2l3 |
| Tbc1d24 | Ptgr1 | Ruvbl2 |
| Snap91 | Spock2 | Tmod1 |
| Astn1 | Nav3 | Shroom2 |
| Sarm1 | Dysf | H2bc3 |
| Smpd3 | Lpxn | Arpc1a |
| Ntrk2 | Apobr | Gldc |
| Slc17a6 | Strbp | Mcu |
| Gad1 | Dnahc9 | Gphn |
| Pmm1 | Ppfibp1 | C2cd4c |
| Necab2 | Fcrls | Psma4 |
| Nos1 | Nsg1 | Ppm1e |
| Adam23 | Irf5 | Mrps22 |
| Camk4 | Myo1b | Tmed10 |
| Calb2 | Ca13 | Capzb |
| Gabra1 | Gbp7 | Cyb5r3 |
| Rac3 | C1qtnf4 | Septin9 |
| Vamp2 | Tmed8 | Otub1 |
| Hepacam | Pcdh8 | Syt2 |
| Napb | Rtkn | Ppia |
| Mpp2 | Slco1a4 | Blvrb |
| Plp1 | Tmem88b | Rap1a |
| Vcan | Cdk6 | Hsd17b8 |
| Syn1 | Tlr2 | Sar1b |
| Doc2a | Vangl2 | Ak2 |
| Dock3 | Ptprk | Dctn3 |
| Unc13a | Kit | Plppr1 |
| Rab3a | Ccp110 | Olfm3 |
| Magi1 | Akr1c14 | Ret |
| Dpp6 | Tap2 | Faf2 |
| Fahd1 | Chd3 | Hsd17b4 |
| Ptprs | Ganc | Actn4 |
| Rasgrp2 | Nudt8 | Ppp5c |
| Cryl1 | Nt5dc1 | Dcc |
| Gpsm1 | Hbegf | Rhob |
| Ppfia3 | Nmi | Psmd11 |
| Arfgef3 | Il6st | Ermp1 |
| Gria1 | Nid2 | Psph |
| Mdga2 | Tor3a | Glo1 |
| Plxna1 | Baz1a | Tigar |
| Ttc7b | FRRS1 | Dhrs7b |
| Stxbp5 | Dab2 | Slc38a1 |

| | | |
|---|---|---|
| Hbb-b1 | Scpep1 | Gpr37l1 |
| Nrxn3 | Arap3 | Trappc3 |
| Efnb3 | Ptpn6 | Tom1l2 |
| Prickle2 | Tsga10 | Arih1 |
| Zer1 | Bcas1 | Omg |
| Atp1b1 | Serpinb8 | Yars1 |
| Cacna1b | Aif1 | Gss |
| Fam171a2 | Plce1 | Impdh2 |
| Kctd16 | Spnb3 | Atp1a1 |
| Dpp10 | Rtn4rl2 | Etfa |
| Grin2b | Gpam | Immt |
| Cd38 | Trim30a | Pank4 |
| Gpd1 | Pla2g7 | Tmem33 |
| Cacnb3 | Serpini1 | Tprg1l |
| Mgst3 | Tusc3 | Cend1 |
| Crip2 | Tns3 | Arf2 |
| Stmn3 | Rgs12 | Pithd1 |
| Nrxn2 | Trim67 | Dnajc11 |
| Tanc2 | Pdlim2 | Stk24 |
| Cd81 | Pcgf2 | Vps4b |
| Ttc9b | Mrps33 | Rab9b |
| Trim3 | Pf4 | Slc30a1 |
| Dcun1d3 | Pir | Prkag2 |
| Wdr47 | Jakmip1 | Stt3a |
| Crmp1 | Elavl3 | Nrcam |
| Capn5 | Man2b1 | Vps53 |
| Slc8a2 | Csf2rb | Krt16 |
| Akap5 | Gm5483 | Lypla1 |
| Cacna2d3 | Fbn1 | Txndc5 |
| Tomm22 | Mt1 | H2az1 |
| Grin1 | Hmgcr | Ttc39c |
| Phactr1 | Dhrs11 | Camk1d |
| Tmem132a | Fyb | Psmf1 |
| Dpysl5 | Sardh | Letmd1 |
| Itgb2 | Sfrp1 | Cdk5 |
| Gstm7 | Megf8 | Sod2 |
| Abhd6 | Mocos | Cct2 |
| Nedd4l | Adcy7 | Dlgap3 |
| Shank2 | Pak6 | Idh3g |
| Cyfip2 | Dcx | Pick1 |
| Kcnj10 | Atp2b3 | Strn4 |
| Robo2 | Parp3 | Tnpo1 |

| | | |
|---|---|---|
| Syn2 | Zbp1 | Krt23 |
| Dlg2 | Tab3 | Cox4i1 |
| Cadps | 9030617O03Rik | Atp6v1e1 |
| Cacna2d1 | Mrps17 | Chchd6 |
| Scamp5 | Ctss | Cds2 |
| Syt5 | Odz4 | Spata2l |
| Acot1 | Afap1l2 | Spag9 |
| Ppfia2 | Trpc4ap | Drd1 |
| Tubb3 | Scamp2 | Asap1 |
| Gpm6a | Pvrl1 | Txnrd2 |
| Kcnma1 | Pla2g4a | Arpc5l |
| Gnb5 | Atat1 | Grid1 |
| Tgm1 | Reln | Kcnc1 |
| Dock10 | Zfp185;Znf185 | Eef1g |
| Rab15 | Ehbp1l1 | Arl8b |
| Wnk2 | Gjc1 | Cdh11 |
| Lcp1 | Nfkbie | Rab12 |
| Slc6a9 | Gm5431 | Mindy2 |
| Scn2b | B2m | Pfkl |
| Ndrg4 | Sox10 | Pacsin2 |
| Arg1 | Tmem106a | Idi1 |
| Ryr2 | Atp8a2 | Sec13 |
| Rims1 | Adamts4 | Ndufs4 |
| Calb1 | Smpdl3a | Ldhb |
| Chn1 | 41888 | Clint1 |
| Pgm2l1 | Lonrf2 | Tst |
| Sh3gl2 | Slc15a2 | Hnrnpk |
| Mapt | Ctsa | Paip1 |
| Palm | Gpc2 | Nln |
| Atl1 | 6430704M03Rik | Stx8 |
| Tubb4a | Dopey1 | Mdp1 |
| Slc1a3 | Vamp8 | Cystm1 |
| Dclk1 | Myo1f | Sv2c |
| Sv2b | Sox8 | Dcaf7 |
| Ephx1 | Myt1l | Pdia3 |
| Mpp3 | Casp1 | Capns1 |
| Ptpn5 | Tep1 | Scn4b |
| Fmnl1 | Atrnl1 | Cadm4 |
| Plxna2 | Txndc15 | Pi4k2a |
| Adam22 | Card9 | Aimp1 |
| Scai | Elavl2 | Gnaq |
| Rab6b | Sipa1 | Pip4k2b |
| Tmem63b | Kcna4 | Cacng4 |

| | | |
|---|---|---|
| Pde1a | Igfbp3 | Ywhae |
| Nbea | Edil3 | Atp6v0a4 |
| Sult4a1 | Crygs | Gbe1 |
| Nebl | Sp4 | Aida |
| Mark1 | Arhgap9 | Sord |
| Slc32a1 | Nfatc2 | Macf1 |
| Gprin1 | Cd300lh;OTTMUSG00000003606 | Ctbp2 |
| Pgbd5 | Cyth4 | Lyst |
| Svop | Hmha1 | Ap1s1 |
| Grin2a | Avil | Bcl2l1 |
| Camk2a | Fbxo45 | Rab3gap1 |
| Mag | Gucy1b3 | Myo5a |
| Cacng8 | Hn1 | Gprc5b |
| Uqcr10 | Spock3 | Arc |
| Atp2b2 | Fcgrt | Lta4h |
| Csmd1 | Lama5 | Zdhhc5 |
| Gsta4 | Arhgef17 | Rab11b |
| Sgsm1 | Lamc1 | Psmd2 |
| Ass1 | C1qtnf5 | C1qbp |
| Rtn1 | Psmb10 | Cpd |
| Dgke | Galnt7 | Eef2 |
| Gja1 | Smarca1 | Kcnip4 |
| Lrrc7 | Plcd4 | H2aj |
| Eef1a2 | Athl1 | Ubqln1 |
| Itgam | Syk | Agpat3 |
| Ccdc51 | Lacc1;9030625A04Rik | Gmfb |
| Rgs6 | Traf7 | Iqsec1 |
| Gria2 | Pon3 | Eef1d |
| Gabra2 | Fdft1 | Atp6ap2 |
| Prmt8 | Themis2 | Nudc |
| Apba1 | Psmb8 | Ipo5 |
| Prkcg | 41886 | Usp46 |
| Dnm3 | Mpg | Lrba |
| Tpm2 | Htatip2 | Ndufaf2 |
| Cnksr2 | Mllt4 | Ppp2ca |
| Rab39b | Itga1 | Ocrl |
| Amph | Rcn3 | Pi4ka |
| Nlgn4l | Sltm | Srp54 |
| Nck2 | Prkcdbp | Eif2s3x |
| Elfn2 | Ell2 | Rock2 |
| Elmod1 | Mrps14 | Fech |
| Slc6a17 | Cnn1 | Crybb1 |
| Il1rapl1 | Fam20c | Ckb |

| | | |
|---|---|---|
| Trpv2 | Sdsl | Atp5pd |
| Fbxo41 | Fgd2 | Snx7 |
| Slc44a1 | S100a16 | Tpm3 |
| Maob | Hfe | Rabggta |
| Plxnc1 | Pard3b | Emd |
| Tnr | Frmpd4 | Eif2s1 |
| Pex5l | Dhx58 | Ndufb3 |
| Negr1 | Gm98;MRF | Dab2ip |
| Arhgap39 | Zbtb7a | Plcl1 |
| Fibp | Wdr66 | Wasl |
| Stmn2 | Macrod2 | Akt3 |
| Ca2 | Cnn3 | Tomm40 |
| Tiam2 | Ptbp3;Rod1 | Cct6a |
| Atp1a3 | Arhgdib | Apool |
| Rap1gap2 | Msr1 | Cpne7 |
| Pde2a | Tmem179 | Pebp1 |
| Gpm6b | Palm2;Akap2 | Uba5 |
| Nefm | Tle1 | Ube2m |
| Fsd1 | Apbb1ip | Eef1a1 |
| Epha4 | Tnfaip8 | Ctnna2 |
| Nlgn2 | Osbpl7 | Tbc1d17 |
| Smpdl3b | Phf11l;D14Ertd668e | Hba |
| Cadps2 | Clec12a | Arl6ip1 |
| Kif5a | Adcy1 | Cops4 |
| Vsnl1 | Bhlhe22 | Dsg1a |
| Dbn1 | Rhof | Hnrnph1 |
| Gap43 | Phlpp2 | Dst |
| Rapgef4 | Was | Apeh |
| Cryab | Epha5 | Bcat1 |
| Sema7a | Lppr4 | Tecpr1 |
| Padi2 | Hira | Mboat7 |
| Brsk1 | Tmem179b | Ndel1 |
| Sgtb | Epb4.9;Epb49 | Scamp1 |
| Map2k6 | Mapk8ip1 | Ppm1h |
| Tppp3 | Sp1 | Ran |
| Grm3 | Ift27 | Psmc4 |
| Slc12a2 | Parp4 | Cadm1 |
| Sfxn5 | Pyhin1 | Vapb |
| Kalrn | Trip10 | Trnt1 |
| Madd | Arhgap28 | Grm7 |
| Cldn11 | Ltbp1 | Asl |
| Cacna2d2 | Fat3 | Rundc3a |
| Hspa12a | Tgfb1 | Acbd6 |

| | | |
|---|---|---|
| Cd200 | Hbb-b1;Beta-s | Tars2 |
| Camkv | Tap1 | Coro2b |
| Ngef | Col8a1 | Aldh18a1 |
| Prkar2b | Amt | Grik3 |
| Atp2a3 | Gmip | Ddx3x |
| Pde10a | C1qb | Slc3a2 |
| Rab3c | Chga | H2bc1 |
| Fbxo2 | Loh12cr1 | Crtac1 |
| Camk2b | Fut9 | Uqcrb |
| Inpp4a | Pacsin3 | Ppm1a |
| Islr2 | Ube2d1 | Hprt1 |
| Ina | Ftsj1 | Gcdh |
| Synpo | Hba-a2;Hba-a1;Hba | Ddah2 |
| Grm2 | Lipa | Calr |
| Srcin1 | Noa1 | Mark2 |
| Arl1 | S100a6 | Wasf3 |
| Sipa1l1 | Cald1 | Mlycd |
| Scn3b | Srxn1 | Flii |
| Ncs1 | H6pd | Snap29 |
| Aqp4 | Pgm5 | Pls3 |
| Cnr1 | Sh3bp1 | Csnk2b |
| Bsn | Cybrd1 | Atic |
| Actn2 | Tmem50a | Lrfn4 |
| Oplah | C4b | Cdk16 |
| Plcb1 | Csad | Ppid |
| Diras2 | Mocs2 | Abhd3 |
| Col1a2 | Nol4 | Slc12a5 |
| Prkar1b | Atp13a2 | Begain |
| Acot11 | Kcnq2 | Suclg1 |
| Rps6ka2 | Cd109 | Ykt6 |
| Neto2 | Lrrc25 | Dlgap4 |
| Hpcal4 | Tmem123 | Map2 |
| Snca | Slc38a2 | Xkr4 |
| Nrbp2 | Igsf11 | Scn1a |
| Glrx5 | Pcdh9 | Acot10 |
| Sv2a | Amz2 | H2bc14 |
| Pten | Gtf2h2 | Vps18 |
| Pgls | Rpusd2 | Sh3glb1 |
| Exog | Mrfap1 | Vps11 |
| Cntnap2 | Ptgs1 | Actc1 |
| Bdh1 | Mgat1 | Vcp |
| Ccdc136 | Odz2 | Mthfd1l |
| Arhgef6 | Rabif | Idh2 |

| | | |
|---|---|---|
| Fxyd6 | P2rx4 | Anxa7 |
| Ptprn2 | Lyz1 | Katnal1 |
| Cacna1a | Il1rap | Selenoi |
| Lamp2 | Igf2bp1 | Purb |
| L1cam | Mapk14 | Vps13c |
| Sorbs2 | Nell2 | Htt |
| Dlg4 | Ptpn13 | Got1 |
| Stx1b | Plekho2 | Rpn1 |
| Gnaz | Cln3 | Tbcel |
| Cnp | Arg2 | Atp2a1 |
| Plxnd1 | Plek | Fasn |
| Atp2b4 | Cacna1c | Gprin3 |
| Gstm1 | Ctbs | Gak |
| Nefl | Gsdmdc1 | Psmc6 |
| Phyhip | Podxl2 | Wfs1 |
| Gdap1 | Ifit1 | Dhodh |
| Src | Hspb8 | Pgm2 |
| Tmem163 | Pstpip1 | Ndufs7 |
| Cadm3 | Acox3 | Coq6 |
| Prdx6 | Gmfg | Sorcs2 |
| Gabrb3 | Afap1 | Acadsb |
| Mras | Rbp1 | Hacd3 |
| Sgip1 | Mapksp1;Lamtor3 | Gk2 |
| Necab1 | Ifitm3 | Grm8 |
| Cpt1c | Fbxo21 | Ndufa9 |
| Sirt5 | Ccdc102a | Pgs1 |
| Kcnab2 | Tbxas1 | Ppp1cb |
| Tsc2 | Bckdhb | Stam |
| Plch2 | Sema4g | Pag1 |
| Nfasc | Cxcl14 | Uchl3 |
| Mbp | Nagpa | Osbpl1a |
| Ckmt1 | Arap2 | Mrps27 |
| Rin1 | Hk3 | Cacng2 |
| Stx1a | Iqce | Lpcat4 |
| Tppp | Mrc1 | Slc7a5 |
| Pcsk2 | Cd180 | Sort1 |
| Cxadr | Spock1 | Aprt |
| Rgs7 | Cd72 | Sh2d5 |
| Cntfr | Pbxip1 | Eif3j1 |
| Plcxd3 | Lrfn1 | Cyc1 |
| Dnm1l | Rnf157 | Septin8 |
| Cpne4 | Cxx1b | Adrbk2 |
| Gltp | Alyref2;Refbp2 | Scarb2 |

| | | |
|---|---|---|
| Prkce | Lcorl | Mtmr9 |
| Ica1 | Pla2g6 | Shmt2 |
| Lpgat1 | Csf1r | Mfsd4a |
| Nlgn1 | C530008M17Rik;Kiaa1211 | Dync1li2 |
| Slc25a44 | Fam49a | Syt17 |
| Gnai1 | Dhrs3 | Pip5k1a |
| Cask | Hck | Prkab2 |
| Daam2 | Hspb1 | Lrpprc |
| Syn3 | Tgm2 | Cpt1a |
| Fam131b | Mast1 | Atp5f1b |
| Cacna1e | Calml4 | Nudt5 |
| Add2 | Lrmp | Elmo1 |
| Pclo | Anpep | Arl6ip5 |
| Sdhc | Tor4a | H2ax |
| Acsf2 | Cspg4 | Gga3 |
| Rhoc | Ephb2 | Pld3 |
| Gls | Bai3 | Plppr4 |
| Rph3a | Aass | Snta1 |
| Cpne6 | AW551984 | Iqsec2 |
| Syngap1 | Sema3c | Atp5f1c |
| Chl1 | Erbb4 | Gdi1 |
| Brsk2 | Evi2b | Tsr2 |
| Hspa2 | Clk4 | Atp2a2 |
| Sncb | Oas1a | Lrrc15 |
| Prune2 | Sybu | Ptpn23 |
| Grm5 | Syt6 | Mmut |
| Pacsin1 | Mea1 | Srprb |
| Trio | Itgal | Dctn2 |
| Mog | Kiaa1045;N28178 | Atp1a2 |
| Kcnd2 | Rel | Acad9 |
| Plxna4 | Mt3 | Mapre3 |
| Arhgap26 | H2-K1 | Hist2h2aa1; Hist2h2aa2 |
| Adcy5 | Arhgap30 | Ptpn11 |
| Wasf1 | Thsd7a | Ahcy |
| Gad2 | Trim5 | Ndufs2 |
| Dync1i1 | Sp110 | Fahd2 |
| Slc8a1 | Creg1 | Dsp |
| Gdap1l1 | C5ar1 | Mtap |
| Epb41l1 | Xdh | Eloc |
| Ppp3cb | Vav1 | Fcsk |
| Clvs1 | Hmox1 | Adgrl2 |
| Syngr3 | Mrps16 | Gna11 |
| Camkk1 | Dock2 | Krt75 |

| | | |
|---|---|---|
| Syt1 | Pcp4l1 | Hist2h2bb |
| Sirt2 | Dnajc1 | Cnrip1 |
| Ppp1r1b | Dos | Cacybp |
| Agap2 | Ablim1 | Sirpa |
| Itpr3 | Myl6b | H3-5 |
| Enpp6 | Gusb | Ndufb11 |
| Slc4a4 | Ifi47 | Gsk3a |
| Mob4 | Lman2l | Gabra5 |
| Ncam1 | Dera | Sgta |
| Cttnbp2 | Satb2 | Rab8b |
| Trim2 | Pld4 | Uggt1 |
| Mblac2 | Alox12l;Alox15 | Ppp3ca |
| Gda | Gpr84 | Gpx4 |
| Dmxl2 | Gadd45gip1 | Rptor |
| Clu | Acad10 | Pof1b |
| Ttc9 | Reep1 | Snap47 |
| Myh14 | AI607873 | Atp6v1g2 |
| Kif5c | Mettl7a1;AB099516;Mettl7a2;Ubie;Mettl7a2-Higd1c | Syne1 |
| Tpm1 | Arhgap33 | Usp9x |
| Dlgap1 | Lyn | Timm9 |
| Shank1 | Osbpl6 | Ubr4 |
| Gabbr1 | Mmp12 | Cox15 |
| Ly6h | Pawr | Coro1b |
| Hapln1 | Irg1 | Uba1 |
| Gfap | Phyhd1 | Atp6ap1 |
| Snap25 | Lcp2 | Calcoco1 |
| Hsd17b11 | Fam151b | Aldoc |
| Acot2 | Cygb | Psma8 |
| Ddc | Btk | Maip1 |
| Nrxn1 | Timm8a1 | Lingo2 |
| Dlg3 | Stard10 | Ankrd63 |
| Itpr2 | Slc37a2 | Dgkb |
| Plxna3 | Fam169a | Ubxn6 |
| Atcay | Ptpn18 | Katnb1 |
| Gnao1 | Ifi204 | Cdh2 |
| Slc2a1 | Slfn1 | Vdac2 |
| Stxbp1 | Wdfy4 | Smap1 |
| Zc2hc1a | Pycard | Anxa2 |
| Sorbs1 | Rbm47 | Vps33a |
| Gria3 | Wdr24 | Map6 |
| F3 | Parvb | Pde4d |
| Erc2 | Rin3 | Pptc7 |

| | | |
|---|---|---|
| Clybl | Alox5 | Cc2d1a |
| Fmnl3 | 2310035C23Rik | Aak1 |
| Slc25a22 | D430041D05Rik | Slc25a25 |
| Ptprd | Pcp4;Igsf5 | Dhrs7 |
| Psd3 | Pvrl3 | Chmp4b |
| Slc16a1 | Tcirg1 | Vac14 |
| Dnajc6 | Hnrpdl | Rars2 |
| Fsd1l | Asap2 | Gmppb |
| Syp | Tbr1 | Copg2 |
| Pdxp | Cd59a | Eif6 |
| Lmtk3 | Pdlim4 | Vamp1 |
| Camsap3 | Slc6a15 | Galk1 |
| Ntm | Bgn | Ehd3 |
| Actr3b | Il16 | Aplp2 |
| Snph | Phkg2 | Rabgap1 |
| | Ifih1 | Nsdhl |
| | Rims2 | Mthfd1 |
| | Lyz2 | Slc10a4 |
| | Efha2 | Ddt |
| | Pitpnm2 | Cacnb4 |
| | Zfp644 | Prrt3 |
| | Siglec1 | Krt77 |
| | Arpp21 | Bphl |
| | Dhx57 | Psmd5 |
| | Naip2 | Relch |
| | Cd84 | Dpp3 |
| | Med10 | Pak3 |
| | Hecw1 | Krt1 |
| | Ccdc39 | Rab22a |
| | Slc2a6 | Rtn3 |
| | Mcam | Ggt7 |
| | Kirrel | Pgam5 |
| | Samd9l | Camk2d |
| | Mns1 | Icam5 |
| | Rab11fip1 | Abi1 |
| | Nmnat1 | Pygb |
| | Tyrobp | Cpne2 |
| | Kiaa1841 | Chrm4 |
| | Rnf213 | Hspa12b |
| | Sugp2 | Tenm2 |
| | Casp8 | Nfs1 |
| | Gucy1a2 | Pdlim5 |
| | Actl6b | Dnaja3 |

| | |
|---|---|
| Tmeff1 | Ndufa2 |
| Kif21b | Pycr3 |
| Qki;Qk | Mri1 |
| Lrp2 | Snx30 |
| Samsn1 | Ankrd34b |
| Slfn5 | Tuba3a; Tuba3b |
| Tapbp | Shisa4 |
| Ighmbp2 | Cdh10 |
| Cd48 | Aldh1l2 |
| Spa17 | Csde1 |
| Col11a1 | Arhgap21 |
| Alb | Acsf3 |
| Scg5 | Acacb |
| Jam2 | Pak1 |
| Gm4902 | Acad8 |
| Sdc4 | Pdk2 |
| Tlr13 | Naca |
| Rsph4a | Pgrmc2 |
| Epha7 | Poldip2 |
| Tmem151b | Tln1 |
| Nid1 | Epdr1 |
| Gulp1 | Ubqln2 |
| Ankrd40 | Asrgl1 |
| Plod2 | Tsg101 |
| Plcg2 | Cct7 |
| Anks1 | Slc7a10 |
| Map9 | Copb1 |
| Ccdc120 | Fyn |
| Glce | Cmtm4 |
| Ctsh | Kirrel3 |
| Gbp4 | Ca4 |
| Psd | Slc9a7 |
| Hspg2 | Cops5 |
| Hook1 | Dmwd |
| Map3k5 | Vat1 |
| Pltp | Aifm1 |
| Fam38a;Piezo1 | Acta2 |
| Rnasel | Tmem121b |
| Slc16a3 | Apoo |
| Rasgrf2 | Numb |
| Celf5 | P2ry12 |
| Sash3 | Necap1 |
| Tmem35 | Lman2 |

| | |
|---|---|
| Dvl1 | Adora2a |
| Mcl1 | Ncstn |
| Rsph1 | Dtymk |
| Wdr19 | Rmnd5a |
| Irgm2 | Grm1 |
| Apc2 | Pik3r1 |
| Mndal | Pccb |
| Ksr2 | Fabp5 |
| Plin2 | Pfkp |
| Tceal5 | Dtd1 |
| Cd63 | Clptm1 |
| Ldlr | Nisch |
| Myo9b | Mpp6 |
| Robo1 | Rcan1 |
| Zfyve9 | Pmpca |
| Fbll1 | Nt5c |
| Rilpl2 | Eci1 |
| Flnb | Galc |
| Hpgds | Asah1 |
| Kif6 | Vps50 |
| Plaur | Stx6 |
| Atp5s | Afdn |
| Ncf2 | Eif3l |
| Plxdc1 | Calm2 |
| Emr1 | Me3 |
| Rttn | Snx4 |
| Lilrb4;Gp49a | Pcyt2 |
| Pde7a | Ajm1 |
| Man2a1 | Slc2a13 |
| Pcdhga8 | Palm2 |
| Dok1 | Ampd3 |
| Xpr1 | Unc13b |
| Plau | Actbl2 |
| Mgat5b | Rab30 |
| Fbxo11 | Nck1 |
| Fat4 | Plxnb1 |
| Psip1 | Txnl1 |
| F13a1 | Nipsnap3b |
| Skap2 | Arfgap1 |
| Snx33 | Snx27 |
| Sulf2 | Atp5md |
| Rab3il1 | Map2k1 |
| Gna14 | Tubg2 |

| | |
|---|---|
| Bcl11b | Gsdma3 |
| Znf512b | Pacs2 |
| Pcyt1b | Ncan |
| Ripk1 | Arpc3 |
| Itga4 | Lrrtm2 |
| Chst10 | Cntn4 |
| Sts | Kctd12 |
| Kiaa1239 | Atp6v1f |
| Odz1 | Gabrb1 |
| C1qc | Surf1 |
| Uaca | Slc25a5 |
| P2rx7 | Micu3 |
| Slc9a9 | Actn1 |
| Dok2 | Kbtbd11 |
| S100a10 | Wdr7 |
| Itih5 | Sdr9c7 |
| Itgb4 | Clcn4 |
| Ptbp2 | Adprs |
| Trim25 | Rhoa |
| Marco | Rtcb |
| Cybb | Appl2 |
| Prr3 | Hace1 |
| Rasa4 | Mink1 |
| Ifit3 | Serpinb1a |
| Scd2;Scd3 | Acaa1b |
| BC006779 | Ndrg2 |
| Met | Dnajb1 |
| Xiap | Cand1 |
| H2-D1 | Hspa5 |
| Fars2 | Atg5 |
| Spin1 | Abat |
| Ccrn4l | Skt |
| Pdlim1 | Dlgap2 |
| Gvin1 | Strn3 |
| Arhgef4 | Rpl13 |
| Cplx1 | Tuba1a |
| Epha3 | Slc25a10 |
| Thg1l | Mlst8 |
| Serpinb10 | Gps1 |
| Hvcn1 | Psmc3 |
| Ly9 | Gng3 |
| Ncf4 | Ogt |
| Dock8 | Rabggtb |

| | |
|---|---|
| Minpp1 | Prrt2 |
| Notch2 | Slc12a6 |
| Pde4b | Pdk3 |
| Slc26a11 | G6pdx |
| Osbpl5 | Cct3 |
| Bin2 | GOLGA7B |
| Synpo2 | Cndp2 |
| Spp1 | Isoc1 |
| Ndnf | Adh5 |
| Unc93b1 | Elob |
| Inpp5d | C1ql3 |
| Plxnb3 | Cryz |
| Mt2 | Bcap29 |
| Kiaa0284 | Agpat4 |
| Pdzrn3 | Rmnd1 |
| Arhgap4 | Rars1 |
| Lamb2 | Nptx1 |
| Alkbh8 | Rad23b |
| Elavl4 | Rab18 |
| Fbn2 | Psmb7 |
| Fam129a | Ppa2 |
| Hnrnpd | Txnrd1 |
| Il1rn | Nccrp1 |
| Dnase2a;Dnase2 | Atp5pb |
| Leprel1 | Hsdl2 |
| Ak7 | Pigs |
| Slc7a7 | Cacng3 |
| Fabp4 | Mff |
| Colec12 | Hsp90ab1 |
| Tmem175 | Tomm20 |
| 2410002F23Rik | Pdha1 |
| Aim2 | Mtor |
| Raph1 | Bcap31 |
| Irs2 | Arfip2 |
| Saa3 | Tcp1 |
| Tlr7 | Glul |
| Mtmr7 | Tollip |
| Ano10 | Gnal |
| Cav1 | Rab3b |
| Msl1 | Lrrc4b |
| Osbp2 | Mtatp6 |
| Rcsd1 | Vcpip1 |
| Afp | Slc9a1 |

| | |
|---|---|
| Ctsc | Emc1 |
| Cd36 | Arhgap23 |
| Mtif3 | Atp6v1e2 |
| Gpnmb | Arl8a |
| Fam108c1 | Tiprl |
| Rem2 | L2hgdh |
| Ano8 | Epb41l3 |
| Cd99l2 | Chat |
| Capn7 | Stat1 |
| Ldhd | Vps26b |
| Fam134a | Psma3 |
| Pram1 | Mydgf |
| Irf8 | Znrd2 |
| Mpeg1 | Vps29 |
| Sparc | Krt5 |
| Stxbp2 | Dagla |
| Ptprc | Vps4a |
| Cyth2 | Prps1 |
| Elp2 | Ppp2r1a |
| Tax1bp3 | Nedd4 |
| Slc17a5 | Mrps23 |
| Tagln | Rps5 |
| Dgkh | Gspt2 |
| Psd2 | Clip1 |
| Fam107a | Nudt10 |
| Stap1 | Ndufa6 |
| Fstl1 | Diras1 |
| Palm3 | Sik3 |
| Dctpp1 | Cbr3 |
| Tgtp | Cyria |
| Meig1 | Rdx |
| Clca1;Clca2 | Acot9 |
| Cnn2 | Syngr1 |
| Lphn1 | Bcl2l13 |
| Ccdc40 | Sncg |
| Alox5ap | Rab27b |
| Ctnnal1 | Vamp7 |
| Flnc | Blmh |
| Stom | Slc24a4 |
| Lppr3 | Pgm3 |
| Cln5 | Pdcd10 |
| Klhdc10 | Syt3 |
| Slc16a7 | Gpr158 |

| | |
|---|---|
| Arl4c | Carm1 |
| Mfi2 | Gsr |
| Snx32 | Hsd17b10 |
| Hyal2 | Trappc4 |
| Purg | Kif21a |
| Slc11a1 | Cdipt |
| Mprip | Wipf3 |
| Mef2a | Fkbp8 |
| Cobll1 | Adgrb1 |
| Ccsmst1 | Acsl3 |
| Galk2 | Rnf214 |
| Dhdh | Gas7 |
| Plekha7 | Cntn1 |
| Brf1 | Itgb8 |
| Dusp15 | Abhd17a |
| Gan | Ndufc2 |
| Celf4 | Cct6b |
| Pik3ap1 | Ak1 |
| Ica1l | Xpo7 |
| Prex2 | Git2 |
| Ttll1 | Ndufb10 |
| Stab1 | Slc25a42 |
| Sin3b | Shank3 |
| Arhgef10 | Nefh |
| Xpa | Mat2b |
| Rab32 | Aldh1b1 |
| Pak7 | Tjp1 |
| Galns | Gstp2 |
| Pdgfra | Cdc37 |
| Kcnj3 | Rgs9 |
| Fmn2 | Cct8 |
| Dynlt3 | Mical3 |
| Fcho1 | Mrs2 |
| Sqrdl | Ndufb4 |
| Map1lc3b;Gm6055 | Hsd17b13 |
| Lbp | Pdhx |
| Man2b2 | Mapk8 |
| Fcgr2b | Aldh1a1 |
| Dtx3l | Rpn2 |
| Efemp2 | Nt5e |
| Aif1l | Gapdh |
| Bbs4 | Eif5a |
| Parp14 | Aldh1a7 |

| | |
|---|---|
| Sema6a | Tgm3 |
| Khdrbs2 | Ech1 |
| Ugt8 | Nme2 |
| Slit1 | Cplx2 |
| Magi3 | Fam136a |
| Prkcq | Stk32c |
| Chd5 | Gfra2 |
| Cp | Traf3 |
| Rbks | Cyld |
| Gtdc1 | Abcb8 |
| Lgals9 | Akr1b7 |
| Zfp238 | Lrp1b |
| Slc43a2 | Slc1a2 |
| Cd14 | Hcn2 |
| Ptrf | Cox5a |
| Wnk3 | Armc8 |
| Kcnt2;Kcnt1 | Gucy1a1 |
| Sez6 | Gdpd1 |
| Gprasp2 | Tmem143 |
| Pml | RTRAF |
| Bcl11a | Rps2 |
| Ranbp6 | Synj2bp |
| Meis2;Meis1 | Rogdi |
| Cd40 | Septin11 |
| Clmp | Glrx3 |
| Larp6 | Psmb6 |
| Faap100 | Hagh |
| Ralgps1 | Sucla2 |
| Adam8 | Cyp46a1 |
| Slc33a1 | Mdh1 |
| Yap1 | Mapre2 |
| Vrk2 | Mapre1 |
| Wipf1 | Ttyh3 |
| Gm1568 | Gsdma2 |
| Nsg2 | Tenm1 |
| Fermt3 | Cpt2 |
| Spnb4 | Rimbp2 |
| Abi3 | Map4 |
| B3gat3 | Vps35 |
| Clec7a | H2bc11; H2bc13; H2bc15; H2bc7 |
| Synm | Shisa7 |
| Gemin6 | Hars1 |
| Txndc17 | Sfxn1 |

| | |
|---|---|
| Clec4n;Clec6a | Commd3 |
| Rnf219 | Cox6c |
| Ston1 | Cfl2 |
| Dnal1;Dnalc1 | Bin1 |
| Aim1 | Rap2b |
| Mcf2l | Sh3glb2 |
| Shroom3 | Srgap2 |
| Ablim3 | Fbxl16 |
| Col1a1 | Wars1 |
| Glipr2 | Kif2a |
| Ncf1 | H2bc12 |
| Folh1 | Pip5k1c |
| Ttbk1 | Arrb1 |
| Wipf2 | Arhgdia |
| Palld | Suclg2 |
| Trex1 | Slc6a11 |
| Chgb | Ociad1 |
| Trip6 | Septin5 |
| Med24 | Myo6 |
| Wsb2 | Epn2 |
| Ifi202 | Ppp2r2c |
| Ccna2 | Timm23 |
| Nadk | Cpe |
| Cep128;4930534B04Rik | Arl15 |
| Rgma | Pdyn |
| Rbfox2;Rbfox1 | Hecw2 |
| Kank1 | Afg3l2 |
| Scn2a1 | Napg |
| Gucy1a3 | Aifm3 |
| Unc119 | Gapdhs |
| Epha2 | Ubtd1 |
| Garnl3 | Spr |
| Aldh1a2 | Ppox |
| Pvalb | Actr1a |
| Trim62 | Paics |
| Ptpn7 | Irgm1 |
| Cyba | Rab11a |
| Kndc1 | Gnas |
| Igfbpl1 | Jup |
| Zc3hav1 | Erlin2 |
| Grb10 | Myh9 |
| Wipi2 | Lysmd2 |
| Lgals3bp | Dap3 |

| | |
|---|---|
| Pdlim7 | Akr1e2 |
| Trp53i11 | Gpsm2 |
| Psmb9 | Prdx3 |
| Ncor1 | Tmem256 |
| Ifit2 | Lancl1 |
| Scube1 | Gde1 |
| Tpcn2 | Fermt2 |
| Zdhhc6 | Slc30a9 |
| Pipox | Ap2a1 |
| Rac2 | Ppp2r5c |
| Adamts5 | Adss2 |
| Ccdc88b | Ntng2 |
| Ccdc92 | Smyd5 |
| Klhdc2 | Ppp6c |
| Grn | Adprh |
| Ap1s2 | Mfsd6 |
| Pars2 | Prkar2a |
| Dtx3 | Slc6a6 |
| Hp | Ndufaf3 |
| Fbln2 | Coa3 |
| Gpsm3 | Myl12b |
| Clstn3 | Hnrnpa2b1 |
| Nbeal2 | Rab33b |
| Irak4 | Gabra3 |
| H2-Ke2;Pfdn6 | Acp6 |
| Mgst1 | Rack1 |
| Sp100 | Cops3 |
| Aes | Zzef1 |
| Igfbp2 | Lap3 |
| Adipoq | Acyp1 |
| Milr1 | Capn1 |
| Igsf3 | Psmd14 |
| Echdc3 | D1Pas1 |
| Scg2 | Chordc1 |
| Tgfbr2 | Stoml2 |
| Naga | Rps6ka1 |
| Cd86 | Krt13 |
| Steap4 | Tomm40l |
| Slc25a18 | Cpne8 |
| Snx11 | Hpcal1 |
| Cpq | Ap1m2 |
| Ttc3 | Arf5 |
| Gprin2 | Ndufb7 |

| | |
|---|---|
| Cd68 | Nckap1 |
| Serpinb2 | C2cd5 |
| Zfp334 | Acat1 |
| Trim46 | Grb2 |
| Sppl2a | Coa7 |
| Stfa3 | Tpi1 |
| 2700081O15Rik | Hnrnph2 |
| Nckap1l | Mpc2 |
| Ube2e3;Ube2e2 | Krt80 |
| Lpcat2 | Smcr8 |
| Aagab | Dsg1b |
| Grip2 | Tsn |
| Mphosph6 | Ap2s1 |
| Def6 | Iglon5 |
| Folr1 | Kpnb1 |
| Plekhg1 | Btbd17 |
| Enc1 | Ahsa1 |
| Enpp2 | Oga |
| Lilrb3 | Lrrtm1 |
| Rltpr | Pip4p2 |
| Cav2 | Ntrk3 |
| Morn4 | Atp6v1h |
| Bcor | Rhot2 |
| Tlr8 | Slc2a3 |
| Ecel1 | Krt12 |
| | Fth1 |
| | Pkm |
| | Mapk1 |
| | Cmpk1 |
| | Cops8 |
| | Krt6a |
| | Hist1h2af |
| | Arhgef12 |
| | Aldh3a2 |
| | Rgs20 |
| | Pdia4 |
| | Stxbp3 |
| | Ttc1 |
| | Nfu1 |
| | Hspa9 |
| | Fbxo3 |
| | Tpd52 |
| | Gmppa |

| |
|---|
| Clic4 |
| Prep |
| Atg2b |
| Psmd6 |
| Ccdc177 |
| Rab24 |
| Rnf141 |
| Ipo4 |
| Abr |
| Cdk14 |
| Slc25a20 |
| Dnaja2 |
| Cnnm1 |
| Psma7 |
| Ppp6r1 |
| Atp6v1g1 |
| Sod1 |
| Pitrm1 |
| Dbnl |
| Ppp1ca |
| Tmem9b |
| Msn |
| Adam11 |
| Dnajb4 |
| Nipsnap2 |
| Mtco1 |
| Rab5a |
| Gabrg2 |
| Picalm |
| Pura |
| Irgq |
| Fam177a1 |
| Osbpl2 |
| Dpysl4 |
| Vdac1 |
| Gpd2 |
| Psmc5 |
| Exoc5 |
| Dctn1 |
| Flna |
| Mfn2 |
| Ptges3 |
| Tenm3 |

| |
|---|
| Mccc2 |
| Tmx1 |
| Alg2 |
| Pgam1 |
| Hspd1 |
| Chmp1a |
| Gmps |
| Ctnnd2 |
| Sorcs3 |
| Slmap |
| Soga3 |
| Wbp2 |
| Msra |
| Isyna1 |
| Sri |
| Ddx1 |
| Stim1 |
| Flrt3 |
| Tpbg |
| Grik5 |
| Uchl5 |
| Pitpna |
| Mlc1 |
| Itpka |
| Fgf12 |
| Ak5 |
| Echdc1 |
| Setd7 |
| Babam1 |
| Akap12 |
| Glod4 |
| Rps6ka3 |
| Kif1b |
| Dync1h1 |
| Stum |
| Timm13 |
| Mtmr2 |
| Mtfp1 |
| Uqcrq |
| Sec23a |
| Rap2a |
| Vps25 |
| Ezr |

| | |
|---|---|
| | Plaa |
| | Dync1li1 |
| | Nap1l1 |
| | Lin7a |
| | Abhd10 |
| | Taok1 |
| | Tm9sf3 |
| | Rps13 |
| | Prnp |
| | Ctsd |
| | Tesc |
| | Grpel1 |
| | Wdr48 |
| | Cox6b1 |
| | Actg2 |
| | Usp7 |
| | Eea1 |
| | Camkk2 |
| | Acat2 |
| | Brinp1 |
| | Tkt |
| | Uso1 |
| | Trim32 |
| | Gdi2 |
| | Pdpk1 |
| | Mrpl12 |
| | Atp5f1d |
| | Scfd1 |
| | Cd47 |
| | Ndufs1 |
| | Cul2 |
| | Crat |
| | Vps13a |
| | Timm29 |
| | Akr7a2 |
| | Ckap4 |
| | Clip2 |
| | Slc18a3 |
| | Krt4 |
| | Epb41 |
| | Atp5mg |
| | Ptpn9 |
| | Acaca |

| | |
|---|---|
| | Psmd13 |
| | Mtmr1 |
| | Ccny |
| | Coq3 |
| | Sar1a |
| | Cisd1 |
| | Dctn5 |
| | Vapa |
| | Agk |
| | Tubg1 |
| | Pip4k2c |
| | Exoc8 |
| | Thnsl1 |
| | Git1 |
| | Cdc42 |
| | Acsl6 |
| | Gsto1 |
| | Opa1 |
| | Vps33b |
| | Sec61a1 |
| | Slc25a3 |
| | Ctbp1 |
| | Prune1 |
| | Erp44 |
| | Hsp90aa1 |
| | Appl1 |
| | Clcn6 |
| | Cat |
| | Clcn3 |
| | Arpc2 |
| | Get4 |
| | Cpne3 |
| | Ndrg3 |
| | Slc25a4 |
| | Pnp |
| | Dip2b |
| | Ptk2 |
| | Psmb2 |
| | Lrpap1 |
| | Sh3gl1 |
| | Chkb |
| | Fabp3 |
| | Marchf5 |

| | |
|---|---|
| | Olfm2 |
| | Dlg1 |
| | Pgam2 |
| | Cops7a |
| | Gba |
| | Cryzl1 |
| | Csrp1 |
| | Septin2 |
| | Them4 |
| | Slc6a1 |
| | Kcnt1 |
| | Mat2a |
| | Retreg2 |
| | Gnb1 |
| | Aldh8a1 |
| | Rala |
| | Ndufs6 |
| | Lmna |
| | Clic1 |
| | Impact |
| | Ptges2 |
| | Pak2 |
| | Myl6 |
| | Copb2 |
| | Tmem30a |
| | Hint1 |
| | Cltc |
| | Tpd52l2 |
| | Psat1 |
| | Ywhab |
| | Ptrh2 |
| | Capn2 |
| | Timm50 |
| | Ide |
| | Hdhd3 |
| | Lmtk2 |
| | Fkbp1a |
| | Srm |
| | Ube2n |
| | Nsfl1c |
| | Cyfip1 |
| | Mlec |
| | Slc12a9 |

| | |
|---|---|
| | Ahcyl1 |
| | Thop1 |
| | Cit |
| | Sec31a |
| | Anxa3 |
| | Ctnnb1 |
| | Impa1 |
| | Farp1 |
| | Aldh1l1 |
| | Pitpnc1 |
| | Jam3 |
| | Acvr1b |
| | Csnk1e |
| | Plpp3 |
| | Eif4e |
| | Vkorc1l1 |
| | Ddx19a |
| | Adk |
| | Acta1 |
| | Trap1 |
| | Pcbp1 |
| | Sdhb |
| | Ncln |
| | Mtpn |
| | Sptan1 |
| | Selenbp1 |
| | Aldh5a1 |
| | Sugt1 |
| | Krt10 |
| | Tpt1 |
| | H2bc4; H2bc6; H2bc8 |
| | Plxnb2 |
| | Osbp |
| | Hint2 |
| | Acadm |
| | Hint3 |
| | Cyp2s1 |
| | Pabpc1 |
| | Igbp1 |
| | Gnl1 |
| | Nagk |
| | Vps26a |
| | Bcas3 |

| |
|---|
| Ssr1 |
| Gpr52 |
| Dlat |
| Micu1 |
| Pacs1 |
| Strap |
| Rab3gap2 |
| Inpp5a |
| Srpk2 |
| Dgki |
| Mtnd4 |
| Iqsec3 |
| H3-3a; H3-3b |
| Gba2 |
| Arcn1 |
| Psma2 |
| Arfgef1 |
| Lancl2 |
| Ttpal |
| Unc13c |
| Sh3kbp1 |
| Mmab |
| Aldh7a1 |
| Slk |
| Wdr45 |
| Lgalsl |
| Dnpep |
| Nit1 |
| Bcr |
| Dld |
| Plscr3 |
| Mtx1 |
| Ggct |
| Ndrg1 |
| Tab1 |
| Eps15l1 |
| Cyb5r1 |
| Eipr1 |
| Slc1a6 |
| Lrrc57 |
| Krt79 |
| Prpsap2 |
| Iqcb1 |

| |
|---|
| Atp6v0a1 |
| Lrp1 |
| Gabrb2 |
| Stx16 |
| Bmerb1 |
| Serpinb6 |
| Dsg1c |
| Tagln2 |
| Xpo1 |
| Ist1 |
| Baiap2 |
| Coro1a |
| Arf1 |
| Sco1 |
| Rnh1 |
| Calml3 |
| Mrps31 |
| Hadh |
| Bmpr2 |
| Atad3 |
| Epm2aip1 |
| Atp5f1e |
| Atp6v1d |
| Pepd |
| Pcbp2 |
| Pafah1b3 |
| Stx4 |
| Pdpr |
| Septin4 |
| Ppa1 |
| Nme1 |
| Abcg2 |
| Slc25a11 |
| Ap2m1 |
| Pdhb |
| Lmbrd2 |
| Rftn2 |
| Ndufb8 |
| Acadvl |
| Snx2 |
| Uqcc1 |
| Add3 |
| Caskin1 |

| |
|---|
| Wdr54 |
| Synrg |
| Mtfr1l |
| Cfl1 |
| Uqcrc2 |
| Tmem65 |
| C2cd2l |
| Tmx4 |
| Hpca |
| Acaa1a |
| Uqcrfs1 |
| Asphd2 |
| Itpr1 |
| Psmc2 |
| Atp2b1 |
| Ppib |
| Gabarapl2 |
| Naxe |
| Pitpnm1 |
| Pik3c3 |
| Hal |
| Dynll1 |
| Rad23a |
| Mdh2 |
| Adcy9 |
| Ntng1 |
| Rtn4 |
| Cep170 |
| Fhl1 |
| Abcb9 |
| Afg3l1 |
| Mccc1 |
| Capza2 |
| Hars2 |
| Blvra |
| Sacm1l |
| Gfm1 |
| Rcn2 |
| Pls1 |
| Erp29 |
| Prdx2 |
| Lysmd1 |
| Cops6 |

| | |
|---|---|
| | Ube2k |
| | Slc7a2 |
| | Atp9a |
| | Marcks |
| | Hsdl1 |
| | Pmpcb |
| | Rnpep |
| | Gpr88 |
| | Epn1 |
| | Atp1b3 |
| | Strip2 |
| | Psme1 |
| | Mapk8ip3 |
| | Tenm4 |
| | Rap1gap |
| | Sdhd |
| | Crkl |
| | Map1b |
| | H3c1; H3c10; H3c11; H3c8 |
| | Prmt1 |
| | Por |
| | Kcna2 |
| | Sars1 |
| | Septin6 |
| | Opa3 |
| | Ndufa7 |
| | Lamtor1 |
| | Aspa |
| | Rasl2-9 |
| | Scp2 |
| | Nol3 |
| | Krt8 |
| | Rpsa |
| | Mapk3 |
| | Vgf |
| | Lrtm2 |
| | Pde1b |
| | Dync1i2 |
| | Rab9a |
| | Hsp90b1 |
| | Tbcb |
| | Ap2a2 |
| | Cbr4 |

| |
|---|
| Dnajc5 |
| Cap1 |
| Hk2 |
| Stub1 |
| Ptcd3 |
| Srgap3 |
| Rab5c |
| Gk |
| Cspg5 |
| Ppp3r1 |
| Rab6a |
| Ogdh |
| Reps1 |
| Lgi1 |
| Igsf8 |
| Arf4 |
| Rplp0 |
| Pcyox1 |
| Ivd |
| Rabgef1 |
| Gstk1 |
| Anks1b |
| Itgav |
| Atp5po |
| Tsfm |
| Pik3r4 |
| Agap3 |
| Dhrs4 |
| Araf |
| Ncdn |
| Vim |
| Rabep1 |
| Prodh |
| Tamm41 |
| Pdcd6ip |
| Aloxe3 |
| Slc38a3 |
| Lzts3 |
| Pck2 |
| Flot2 |
| Tuba4a |
| Gnai3 |
| Slc4a3 |

| |
|---|
| Emc2 |
| Psmb1 |
| Synpr |
| Tmx3 |
| Efr3b |
| Rhot1 |
| Nsf |
| Hyou1 |
| Agap1 |
| Psmb5 |
| Cpm |
| Daam1 |
| Dtnb |
| Tm9sf2 |
| Atg16l1 |
| Wdr77 |
| Rab10 |
| Aars1 |
| Twf2 |
| Slc25a13 |
| Prkar1a |
| Idh3a |
| Dnajb2 |
| Gch1 |
| Ryr3 |
| Arhgef7 |
| Kiaa1549 |
| Prxl2b |
| Ftl1 |
| Synj1 |
| Eif4b |
| Aco1 |
| Fam126b |
| Taldo1 |
| Aco2 |
| Maoa |
| Map1lc3a |
| Hspa4 |
| Asic1 |
| Slc30a3 |
| Cct4 |
| Vps51 |
| Gstm5 |

| | |
|---|---|
| | Slc22a23 |
| | Timmdc1 |
| | Rab23 |
| | Ado |
| | Glud1 |
| | Ndufv1 |
| | Abhd12 |
| | Psmd7 |
| | Cs |
| | Ephx2 |
| | S1pr1 |
| | Ap1g2 |
| | Chrnb2 |
| | Slc25a23 |
| | Cad |
| | Actb |
| | Rab7a |
| | Pam |
| | Eno1 |
| | Prrt1 |
| | Actr2 |
| | Aldoa |
| | Krt72 |
| | Lin7c |
| | Ap3b1 |
| | Acot7 |
| | Klc2 |
| | Mtch1 |
| | Pip5k1b |
| | Phyhipl |
| | Adcy6 |
| | Slc4a8 |
| | Rac1 |
| | Heatr5b |
| | Tom1 |
| | Mpi |
| | Actr10 |
| | Adam10 |
| | Wdr1 |
| | Spg7 |
| | Thy1 |
| | Akap7 |
| | Nptxr |

| |
|---|
| Myo18a |
| Ndufa13 |
| Vat1l |
| Ache |
| Npepps |
| Entpd2 |
| Arl2 |
| Wdr37 |
| Agpat5 |
| Tpd52l1 |
| Myadm |
| Ndufa4 |
| Krt26 |
| Osbpl10 |
| Hibadh |
| Psmb4 |
| Cpne9 |
| Eef1b |
| Ap1g1 |
| Hccs |
| Memo1 |
| Tfrc |
| Ank2 |
| Alcam |
| Plcg1 |
| Anxa4 |
| Ap3m2 |
| Snx1 |
| Ap1m1 |
| Usp15 |
| Eci3 |
| Eml5 |
| Tars1 |
| Kiaa0513 |
| Rapgef2 |
| Ube2v2 |
| Mpst |
| Klc1 |
| Spcs2 |
| Gmpr |
| Cops2 |
| Kcnj4 |
| Sec22b |

| | |
|---|---|
| | Arhgap32 |
| | Csnk2a1 |
| | Psmd1 |
| | Gatd3a |
| | Ube2o |
| | Prkcd |
| | Hip1 |
| | Plcd1 |
| | Pfn1 |
| | Ube3a |
| | Stip1 |
| | Elmo2 |
| | App |
| | Ndufa11 |
| | Ldha |
| | Dennd11 |
| | Atp6v1a |
| | Celsr2 |
| | Cul1 |
| | Gabbr2 |
| | Psmd12 |
| | Ppm1f |
| | Prkcsh |
| | Hexb |
| | Tmem126a |
| | Eif4a1 |
| | Ppp1r9b |
| | Napa |
| | Dynlrb1 |
| | H2ac20 |
| | Atp5mf |
| | Cct5 |
| | Cul3 |
| | Exoc3 |
| | Ywhag |
| | Ndufb5 |
| | Fah |
| | Vps52 |
| | Basp1 |
| | Hip1r |
| | Rufy3 |
| | Cap2 |
| | Hsd17b12 |

| |
|---|
| Nif3l1 |
| Lsamp |
| Dad1 |
| Glg1 |
| Snx6 |
| Syt7 |
| Mtnd5 |
| Gnb4 |
| Rcn1 |
| Avl9 |
| Cltb |
| Ppp2r5e |
| Elmod2 |
| Aldh6a1 |
| Fundc2 |
| Fkbp4 |
| Slc25a46 |
| Oxsr1 |
| Tbcd |
| Vwa8 |
| Eif5 |
| Arpc5 |
| Trappc9 |
| Endod1 |
| Rdh14 |
| Me1 |
| Pc |
| Gaa |
| Gsn |
| Neo1 |
| Gng7 |
| Crk |
| Atp6v0d1 |
| Xpnpep1 |
| Mt-Cyb |
| Nans |
| Tdrkh |
| Wdr13 |
| Csnk1a1 |
| Mapk10 |
| Becn1 |
| Ehd1 |
| Exoc4 |

| | |
|---|---|
| | Plec |
| | Hdhd2 |
| | Eif3e |
| | M6pr |
| | Ndufs5 |
| | Ugp2 |
| | Dynll2 |
| | Stmn1 |
| | Sigmar1 |
| | Prkaca |
| | Nudt3 |
| | Tomm70 |
| | Sptb |
| | Olfm1 |
| | Slc44a2 |
| | Exoc7 |
| | Septin3 |
| | P4hb |
| | Hspa8 |
| | Plcl2 |
| | Pgrmc1 |
| | Reep5 |
| | Dusp3 |
| | Pfkm |
| | Gpc4 |
| | Pitpnb |
| | Ablim2 |
| | Tmem11 |
| | Zfyve1 |
| | Itsn1 |
| | Agpat1 |
| | Kpna3 |
| | Tnpo3 |
| | Pcyox1l |
| | Kifap3 |
| | Fam162a |
| | Kif1a |
| | Atg7 |
| | Abl2 |
| | Stk11 |
| | Uchl1 |
| | Adgrb3 |
| | Gripap1 |

| |
|---|
| Rgs14 |
| Ak4 |
| Exoc6b |
| Sdha |
| Epb41l2 |
| Slc25a51 |
| Lgals3 |
| Psmd3 |
| Eif5a2 |
| Nap1l4 |
| Kcna6 |
| Cycs |
| Nadk2 |
| Mtco2 |
| Cyb5b |
| Tnik |
| Hspe1 |
| Lasp1 |
| Itpa |
| Actr3 |
| Nme3 |
| Acsbg1 |
| Gucy1b1 |
| Ciapin1 |
| Wdfy3 |
| Cmpk2 |
| Vti1b |
| Htra2 |
| Czib |
| Psma6 |
| Tfam |
| Serpinb5 |
| Nlgn3 |
| Dpysl3 |
| Ppp1r7 |
| Acp1 |
| Bckdk |
| Mtch2 |
| Dars1 |
| Mtnd1 |
| Grk2 |
| Erc1 |
| Fh |

| |
|---|
| Ipo7 |
| Ptk2b |
| Rab1b |
| Gdpd5 |
| Evl |
| Apoe |
| Decr1 |
| Pdxk |
| Cdc42bpa |
| Eps15 |
| Pgap1 |
| Strn |
| Agfg1 |
| Ppp6r2 |
| Pgp |
| Ddhd2 |
| Fmnl2 |
| Hspa1a |
| Acsl5 |
| Lrrc8b |
| Nipsnap1 |
| Myh10 |
| Tubb2a |
| Dbt |
| Armc10 |
| Vps16 |
| Ptpre |
| NARS1 |
| Acly |
| Ckap5 |
| Ptprj |
| Ppt1 |
| Krt17 |
| Rab35 |
| Arhgap44 |
| Cdk17 |
| Sfxn3 |
| Samm50 |
| Hgs |
| Map2k4 |
| Lnpk |
| Atpaf1 |
| Upf1 |

| |
|---|
| H2ac12 |
| Bles03 |
| Naxd |
| Lrrc4c |
| Apmap |
| Gng2 |
| Hadhb |
| Ranbp9 |
| Creld1 |
| Pafah1b2 |
| Man2c1 |
| Atp5me |
| Ndufb9 |
| Gabrd |
| Etfdh |
| Gnpda1 |
| Osbpl8 |
| Acox1 |
| Slc7a8 |
| Gnb2 |
| Grik2 |
| Ap3s2 |
| Zadh2 |
| Bpnt1 |
| Capza1 |
| Csmd3 |
| Nectin1 |
| Map1s |
| Rragd |
| Ptpra |
| Alad |
| Ppp2r2a |
| Rnf14 |
| Slc23a2 |
| Atp1b2 |
| Hmgcl |
| S100a11 |
| Anxa6 |
| Scrn1 |
| Srr |
| Pcmt1 |
| Ric8a |
| Cpne1 |

| | |
|---|---|
| | Rps3 |
| | Sdk2 |
| | Kif5b |
| | Reps2 |
| | Etf1 |
| | S100a14 |
| | Sfn |
| | Eif3i |
| | G6pd2 |
| | Atp6v1c1 |
| | Pip4k2a |
| | Nudt16 |
| | Vps36 |
| | Tspan7 |
| | Tmx2 |
| | Prdx1 |
| | Tcp11l1 |
| | Sparcl1 |
| | Rab11fip2 |
| | Ppp2r1b |
| | Rab5b |
| | Trappc11 |
| | Dnm1 |
| | Ptprr |
| | Hsph1 |
| | Rock1 |
| | Cldnd1 |
| | Dhrs1 |
| | Selenbp2 |
| | Slc25a24 |
| | Tagln3 |
| | Lrrtm4 |
| | Abcd3 |
| | Ndufaf1 |
| | Itfg1 |
| | Acadl |
| | Anxa1 |
| | Ttyh1 |
| | Itgb1 |
| | Kctd8 |
| | Plpbp |
| | Atg9a |
| | Pycr2 |

| |
|---|
| Arfgef2 |
| Clta |
| Prpsap1 |
| Myo5b |
| Ccdc127 |
| Adck1 |
| Dnaja4 |
| Clpp |
| Nit2 |
| Kif17 |
| Anxa11 |
| Arf3 |
| Fscn1 |
| St13 |
| Flot1 |
| Slc25a14 |
| Cse1l |
| Fn3k |
| Hspa4l |
| Guk1 |
| Slc6a3 |
| Cab39 |
| Ppp1r12a |
| Bnip3 |
| Gatd1 |
| Adrm1 |
| Comtd1 |
| Ftl2 |
| Ppp2r5a |
| Pdk1 |
| Gria4 |
| Vwa5a |
| Gpi |
| Rras2 |
| Tmod2 |
| H3c13; H3c14; H3c15; H3c2; H3c3; H3c4; H3c6; H3c7 |
| Sec14l2 |
| Pcca |
| Atp6v0a2 |
| Cyrib |
| Slc17a7 |
| Mark3 |
| Lonp1 |

| | |
|---|---|
| | Dstn |
| | Vdac3 |
| | Ppm1b |
| | Vars1 |
| | Sdr39u1 |
| | Ddx3y |
| | Susd2 |
| | Ppp1r21 |
| | Ssr4 |
| | Ywhaz |
| | H2az2 |
| | Cacnb1 |
| | Cdc42ep4 |
| | Dgkz |
| | Dip2a |
| | Tmed9 |
| | Ddost |
| | Atad1 |
| | Ddah1 |
| | Mtx2 |
| | Vamp3 |
| | Hadha |
| | Emc3 |
| | Grhpr |
| | Neto1 |
| | Extl2 |
| | Myl9 |
| | Ergic1 |
| | Gclc |
| | Dgkq |
| | Dmtn |
| | Ctnnd1 |
| | Auh |
| | Gsg1l |
| | Cntnap4 |
| | Rpl27a |
| | Mecr |
| | Ptp4a1 |
| | Tubb5 |
| | Pnpla8 |
| | Ap3d1 |
| | Mal2 |
| | Ralb |

| | |
|---|---|
| | Dock4 |
| | Fam114a2 |
| | Ampd2 |
| | Dtna |
| | Gsk3b |
| | Rpl15 |
| | Lypla2 |
| | Slc25a12 |
| | Rdh11 |
| | Lamp5 |
| | Vta1 |
| | Th |
| | Rbbp9 |
| | Gna13 |
| | Nucb1 |
| | Mrpl39 |
| | Twf1 |
| | Igsf21 |
| | Denr |
| | Hibch |
| | Nomo1 |
| | Cdip1 |
| | Vbp1 |
| | Ndufs8 |
| | Gstz1 |
| | Snx16 |
| | Dnajb11 |
| | Lin7b |
| | Skp1 |
| | Pkp1 |
| | Fam210a |
| | Vcam1 |
| | Prdx5 |
| | Vti1a |
| | Smap2 |
| | Spryd7 |
| | Trim9 |
| | Add1 |
| | Atg3 |
| | Rab4b |
| | Babam2 |
| | Hcn1 |
| | Clasp2 |

| | |
|---|---|
| | Eno2 |
| | Gsdma |
| | Park7 |
| | Abcb10 |
| | Bcan |
| | Nipa1 |
| | Efhd2 |
| | Itm2b |
| | Slc5a7 |
| | Arhgap1 |
| | Dctn4 |
| | Hbb-b2 |
| | Plgrkt |
| | Prkcb |
| | Map1a |
| | Pgd |
| | Cul5 |
| | Hebp1 |
| | Slc4a10 |
| | Ndufa8 |
| | Rragc |
| | Iba57 |
| | Ubtd2 |
| | Phf24 |
| | Acsl1 |
| | Flad1 |
| | Nwd2 |
| | Slc9a3r1 |
| | Ppp2cb |
| | Aldh4a1 |
| | Sars2 |
| | Rap1b |
| | Fbxl4 |
| | Akr1b1 |
| | Eif3j2 |
| | Gstm3 |
| | Atp8a1 |
| | Acaa2 |
| | Tsc1 |
| | Phb |
| | Psmd8 |
| | Psma1 |
| | Jagn1 |

| |
|---|
| Ppp1cc |
| Tbrg4 |
| Nae1 |
| Coasy |
| Slc18a2 |
| Ubfd1 |
| Oat |
| Huwe1 |
| Sbf1 |
| Pgk1 |
| Ddb1 |
| Apbb1 |
| Akr1a1 |
| Ccdc6 |
| Oxr1 |
| Prkacb |
| Pex14 |
| Ndufs3 |
| Hspbp1 |
| Hmox2 |
| Slc7a14 |
| Gpx1 |
| Ola1 |
| Gpd1l |
| Ap2b1 |
| Etfb |
| Ddi2 |
| Eif4h |
| H2bc9 |
| Ufc1 |
| Stx12 |
| Ndufb6 |
| Lhfpl4 |
| Prxl2a |
| Eif4a2 |
| Oscp1 |
| Faah |
| Rhog |
| Prkca |
| Exoc2 |
| Mgll |
| Canx |
| Efr3a |

| |
|---|
| Pdcd6 |
| Gfod1 |
| Coq5 |
| Slc35f1 |
| Lrrc47 |
| Ndufa10 |
| Abraxas2 |
| Timm44 |
| Rmdn3 |
| Got2 |
| Chp1 |
| Vps45 |
| Kcnip2 |
| Esd |
| Pdp1 |
| Acap2 |
| Emb |
| Eppk1 |
| Rab14 |
| Abhd16a |
| Bckdha |
| Rgs8 |
| Krt42 |
| Eprs1 |
| Unc5a |
| Idh1 |
| Dlst |
| Uqcrc1 |
| Me2 |
| Mtatp8 |
| Gart |
| Scn1b |
| Snx5 |
| Ncam2 |
| Ehd4 |
| Itm2c |
| Cox5b |
| Gabra4 |
| Ptprn |
| Cadm2 |
| Rpl3 |
| Cntnap1 |
| Kcnab1 |

| |
|---|
| Gmds |
| Dglucy |
| Rps9 |
| Insrr |
| Arpc4 |
| Pfn2 |
| Sh3gl3 |
| Scn2a |
| Pgm1 |
| Csnk2a2 |
| Fis1 |
| Prss2 |
| Arf6 |
| Fnbp1 |
| Cpne5 |
| Vipas39 |
| Sptbn1 |
| Bsg |
| Psmb3 |
| Nptn |
| Efhd1 |
| Ermn |
| Eml2 |
| Slc1a4 |
| Ptprf |
| Camk2g |
| Armc1 |
| Mif |
| Phgdh |
| Mrtfb |
| Fry |
| Pdia6 |
| Nqo2 |
| Glrx |
| Stk39 |
| Ank3 |
| Armc6 |
| Magi2 |
| Praf2 |
| Ralgapa1 |
| Crym |
| Slc6a7 |
| Trappc12 |

| | |
|---|---|
| | Slc25a1 |
| | Arl3 |
| | Nceh1 |
| | Tmem35a |
| | Rab2a |
| | Arhgef9 |
| | Ndufa3 |
| | Bcs1l |
| | Chchd3 |
| | Zmpste24 |
| | H2aw |
| | Nampt |
| | Ptpa |
| | Inpp1 |
| | Gpc1 |
| | Ccz1 |
| | Ap3m1 |
| | Aldh9a1 |
| | Faim2 |
| | Ptp4a2 |
| | Atp5f1a |
| | Stx3 |
| | Cep170b |
| | Atp6v1b2 |
| | Ndufa12 |
| | H2ac7 |
| | Eci2 |
| | Scyl2 |
| | Stx7 |
| | Cttn |
| | Spart |
| | Serinc1 |
| | Lxn |
| | Oxct1 |
| | Anxa5 |
| | Pygm |
| | Mtarc2 |
| | Krt14 |
| | Ube2v1 |
| | Phb2 |
| | Gapvd1 |
| | Rab1A |
| | Usp24 |

| | |
|---|---|
| | Adsl |
| | Wdr44 |
| | Ndufv2 |
| | Ap3b2 |
| | Taok3 |
| | Psma5 |
| | Rab31 |
| | Psmd4 |
| | Ipo9 |
| | Rasal1 |
| | Iars2 |
| | Bsdc1 |
| | Ndufaf5 |
| | Enoph1 |
| | Ranbp1 |
| | Cntn2 |
| | Dpysl2 |
| | Letm1 |
| | Mocs3 |
| | Echs1 |
| | Tpp2 |
| | Slc27a4 |
| | Ppp6r3 |
| | Fnbp1l |
| | Coro1c |
| | Pkp4 |
| | Gstp1 |
| | Ggps1 |
| | Cdc42bpb |
| | Septin7 |
| | Sccpdh |
| | Pef1 |
| | Cyb5a |
| | Frrs1l |
| | Lrrc8a |
| | Gars1 |
| | Get3 |
| | Ppme1 |
| | Kcna1 |
| | Bag6 |
| | Tubgcp2 |
| | F8a1 |
| | Rab8a |

| | |
|---|---|
| | Shisa9 |
| | Them6 |
| | Pin1 |
| | Dock9 |
| | Mcts1 |
| | Hspa1b |
| | Brcc3 |
| | Dnajb6 |
| | Nt5c3a |
| | Tubb2b |
| | Hk1 |
| | Krt76 |
| | Lrrtm3 |
| | Kazn |
| | Psmd9 |
| | Scamp3 |
| | Numbl |
| | Gspt1 |
| | Gnai2 |
| | Nf1 |
| | Uba1y |
| | Adgrl1 |
| | Wwox |
| | Ruvbl1 |
| | H4c1; H4c11; H4c12; H4c14; H4c2; H4c3; H4c4; H4c6; H4c8; H4c9; H4f16; Hist1h4m |
| | Usp14 |
| | Prmt5 |
| | Rab21 |
| | Cdh13 |
| | Ipcef1 |
| | Lactb |
| | Qdpr |
| | Cars1 |
| | Vcl |
| | Tufm |
| | Scrn3 |
| | Enah |
| | Rida |
| | Homer1 |
| | Tmem43 |
| | Prkag1 |
| | Rpl11 |
| | Adgrl3 |

| | |
|---|---|
| | Ywhaq |
| | Atl2 |
| | Sphk2 |
| | Pcdhga4 |
| | Pafah1b1 |
| | Bag5 |
| | Coq9 |
| | Ap3s1 |
| | Rpl12 |
| | Syt12 |
| | H2ac15 |
| | Sco2 |
| | Usp5 |
| | Ncald |
| | Slc25a40 |
| | Lamp1 |
| | Uhrf1bp1l |
| | Aldh2 |
| | Cbr1 |
| | Ywhah |
| | Nckipsd |
| | Actr1b |
| | Psmc1 |
| | Dnaja1 |
| | Ganab |
| | Slc29a1 |
| | Cotl1 |
| | Hist1h2bp |
| | Abcb7 |
| | Ctsb |
| | Fdps |
| | Tln2 |
| | Plppr3 |
| | Rp2 |
| | Rheb |
| | Ak3 |
| | Ahcyl2 |
| | Uchl4 |
| | Tph2 |
| | Ckmt2 |
| | Mpp1 |
| | Ap1b1 |
| | Fgf14 |

## Supplementary Table 5: Proteomics Comparison With KEGG Neurotransmission Pathways

| Synaptic Vesicle Cycling | |
|---|---|
| Pre-synapse | |
| KEGG Name | Associated Genes |
| Transporters | Slc32a1 Slc18a1/2/3/ Slc17a6/7/8 |
| Transporters | Slc1a1/2/3/6/7 Slc6a1/2/3/4/5/7/9/11/12/13 |
| SYT | Syt1 |
| VAMP | Vamp2 |
| Rab3A | Rab3a |
| RIM | Rims |
| Munc18 | Stxbp1 |
| Munc13 | Unc13a/b/c |
| Syntaxin | Stx1a/b/2/3 |
| SNAP25 | Snap25 |
| Complexin | Cplx1/2/3/4 |
| VGCC | Cacna1a/b |
| NSF | Nsf |
| alpha-SNAP | Napa |
| Dyna | Dmn1/2/3 |
| AP2 | Ap2a1/2/b1/m1/s1 |
| Clathrin | Clta/b/c/cl1 |
| vATPase | Atp6v1a/b1/b2/c2/c1/d/e2/e1/f/g1/g3/g2 Atp6v0e1/2 |
| SYN | Syn1/2/3 |
| Glutamatergic Synapse | |
| Pre-synapse | |
| KEGG Name | Gene Name |
| mGluR2/3 | Grm2/3 |
| mGluR4/8 | Grm4/8 |
| GLNT | Slc38a1/2 |
| GLS | Gls/2 |
| GRK | Grk2/3 |
| AC | Adcy1/2/3/4/5/6/7/8/9 |
| Gi/o | Gnai1/2/3 Gnao1 Gnb1/2/3/4 Gng2/3/4/5/7/8/10/11/12/13 Gngt1/2 |
| PKA | Prkaca/b/g |
| VGLUT | Slc17a6/7/8 |
| mGluR7 | Grm7 |
| GIRK | Kcnj3 |
| KA | Grik1/2/3/4/5 |
| VGCC | Cacna1a |
| EAAT | Slc1a2/7 |
| Post-synapse | |

| KEGG Name | Gene Name |
|---|---|
| KA | Grik1/2/3/4/5 |
| AMPAR | Gria1/2/3/4 |
| EAAT | Slc1a1/6 |
| VGCC | Cacna1c/d |
| NMDAR | Grin1/2a/2b/2c/2d/3a/3b |
| PLA2 | Pla2g4a/b/c/d/e/f Jmjd7-Pla2g4b |
| PLD | Pld1/2 |
| ERK1/2 | Mapk1/3 |
| PKC | Prkca/b/g |
| PKA | Prkaca/b/g |
| IP3R | Itpr1/2/3 |
| PLC | Plcb1/2/3/4 |
| Homer | Homer1/2/3 |
| PP2B | Ppp3ca/cb/cc/r1/r2 |
| Gi/Go | Gnai1/2/3 Gnao1 |
| AC | Adcy1/2/3/4/5/6/7/8/9 |
| Gs | Gnas |
| Gq | Gnaq |
| SHANK | Shank1/2/3 |
| GKAP | Dlgap1 |
| PSD95 | Dlg4 |
| TRPC1 | Trpc1 |
| mGluR1/5 | Grm1/5 |
| mGluR2/3 | Grm2/3 |
| mGluR4/6 | Grm4/6 |
| mGluR7/8 | Grm7/8 |
| GABAergic Synapse | |
| Pre-synapse | |
| KEGG Name | Gene Name |
| GLS | Gls/2 |
| SystemA | Slc38a1/2 |
| GAD | Gad1/2 |
| VIAAT | Slc32a1 |
| GABA-T | Abat |
| AC | Adcy1/2/3/4/5/6/7/8/9 |
| Gi/o | Gnai1/2/3 Gnao1 Gnb1/2/3/4 Gng2/3/4/5/7/8/10/11/12/13 Gngt1/2 |
| PKA | Prkaca/b/g |
| VGCC | Cacna1a/b |
| GAT | Slc6a1/11/12/13 |
| GABAB | Gabbr1/2 |
| Post-synapse | |
| KEGG Name | Gene Name |

| | |
|---|---|
| GABAA | Gabra1/2/3/4/5/6 Gabrb1/2/3 Gabrd/e Gabrg1/2/3 Gabrp/q |
| PKA | Prkaca/b/g |
| Src | Src |
| PKC | Prkca/b/g |
| HAP | Hap1 |
| GRIF | Trak2 |
| PRIP | Plcl1 |
| Gephyrin | Gphn |
| GABARAP | Gabarap/pl1/pl2 |
| NSF | Nsf |
| KCC2 | Slc12a5 |
| GABAC | Gabrr1/2/3 |
| VGCC | Cacna1a/b/c/d/f |
| GABAB | Gabbr1/2 |
| GIRK2 | Kcnj6 |
| Gi/o | Gnai1/2/3 Gnao1 Gnb1/2/3/4 Gng2/3/4/5/7/8/10/11/12/13 Gngt1/2 |
| AC | Adcy1/2/3/4/5/6/7/8/9 |
| Cholinergic Synapse | |
| Pre-synapse | |
| KEGG Name | Gene Name |
| M2 | Chrm2 |
| M4 | Chrm4 |
| Gi/o | Gnai1/2/3 Gnao1 |
| VGCC | Cacna1a/b |
| vAChT | Slc18a3 |
| AChE | Ache |
| ChAT | Chat |
| CHT | Slc5a7 |
| Post-synapse | |
| KEGG Name | Gene Name |
| M5 | Chrm5 |
| M3 | Chrm3 |
| M1 | Chrm1 |
| Gq/11 | Gnaq/Gna11 |
| PLC | Plcb1/2/3/4 |
| IP3R | Itpr1/2/3 |
| PKC | Prkca/b/g |
| Kv7/Kir2 | Kcnj2/4/12/14/18 Kcnq1/2/3/4/5 |
| Kir3 | Kcnj3/6 |
| PI3K(y) | Pik3r5/6 Pi3kcg |
| Ras | H/K/N/ras |
| Mek | Map2k1 |
| Erk | Mapk1/3 |

| | |
|---|---|
| c-fos | Fos |
| Gi/o | Gnai1/2/3 Gnao1 Gnb1/2/3/4 Gng2/3/4/5/7/8/10/11/12/13 Gngt1/2 |
| M2 | Chrm2 |
| M4 | Chrm4 |
| AC | Adcy1/2/3/4/5/6/7/8/9 |
| PKA | Prkaca/b/g |
| CREB | Atf4/Creb1/3/5 Creb3l1/2/3/4 |
| nAChR | Chrna3/4/6/7 Chrnb2/4 |
| CaMK | Camk2a/2b/2d/2g/4 |
| Fyn | Fyn |
| JAK2 | Jak2 |
| PI3K | Pi3kca/cb/r1/r2/r3 |
| PKB/Akt | Akt1/2/3 |
| Bcl-2 | Bcl2 |
| VGCC | Cacna1c/d/f/s |

Dopaminergic Synapse

Pre-synapse

| KEGG Name | Gene Name |
|---|---|
| D2 | Drd2 |
| TH | Th |
| DDC | Ddc |
| VMAT | Slc18a1/2 |
| VGCC | Cacna1a/b |
| DAT | Slc6a3 |
| MAO | Maoa/b |
| COMT | Comtd1 Comt Lrtomt |

Post-synapse

| KEGG Name | Gene Name |
|---|---|
| D1 | Drd1 |
| D5 | Drd5 |
| VSCC | Scn1a |
| IP3R | Itpr1/2/3 |
| Calcyon | Caly |
| Gq | Gnaq |
| PLC | Plcb1/2/3/4 |
| Gs/olf | Gnas/l |
| AC5 | Adcy5 |
| PKA | Prkaca/b/g |
| CaM | Calm2/3 |
| PKC | Prkca/b/g |
| CaMKII | Camk2a/2b/2d/2g |
| PP2B | Ppp3ca/cb |
| CREB | Creb1/3/5 Atf2/4/6b Creb3l1/2/3/4 |

| | |
|---|---|
| c-fos | Fos |
| MAPK | Mapk8/9/10/11/12/13/14 |
| DARPP32 | Ppp1r1b |
| PP-1 | Ppp1ca/cb/cc |
| NMDAr | Grin1/2a/2b |
| AMPAr | Gria1/2/3/4 |
| Kinesin | Kif5a/b/c |
| Clock | Clock |
| BMAL1 | Arntl |
| D2 | Drd2 |
| PP2A | Ppp2ca/b Ppp2r1a/c Ppp2r2a/b/c/d Ppp2r3a/b/c Ppp2r5a/b/c/d/e |
| Gi/o | Gnai1/2/3 Gnao1 Gnb1/2/3/4 Gng2/3/4/5/7/8/10/11/12/13 Gngt1/2 |
| Akt | Akt1/2/3 |
| GSK-3 | Gsk3a/3b |
| bArrII | Arrb2 |
| Cav1.2/1.3 | Cacna1c/d |
| GIRK | KcnJ3/5/6/9 |
| Cav2.1/2.2 | Cacna1a/b |

**Supplementary Data: Uncropped images from capillary immunoblot experiments**

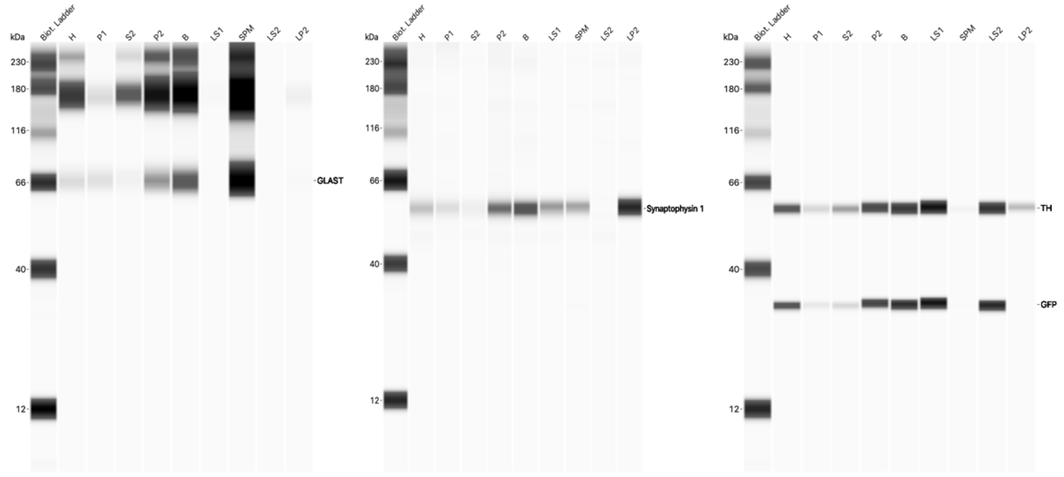


Pictures from Figure 1c

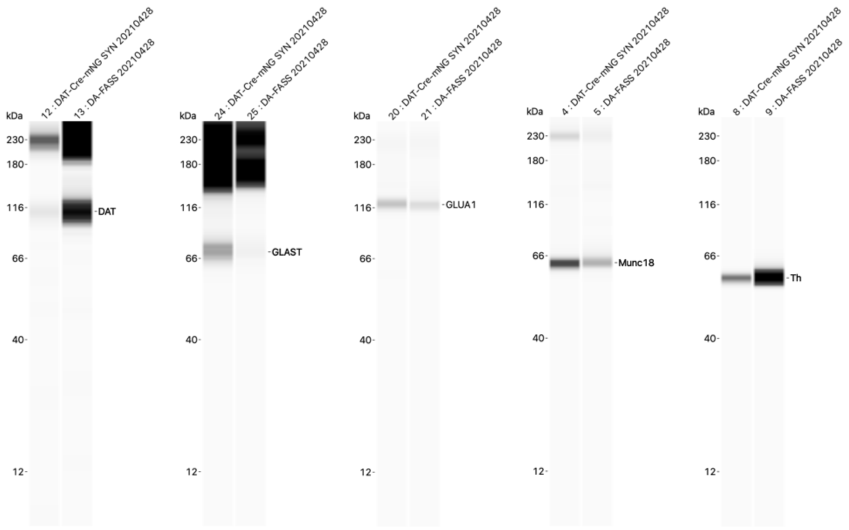


Pictures from Figure 1g

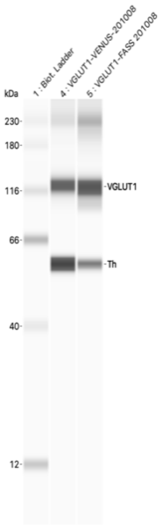


Picture from Figure 7c
