# Supplementary material for: A synaptomic analysis reveals dopamine hub synapses in the mouse striatum
Source: Nat Commun. 2022 Jun 3;13:3102. doi: 10.1038/s41467-022-30776-9 (PMC9166739; doi:10.1038/s41467-022-30776-9)
Supplement: Supplementary file 3 — Reporting Summary [file 41467_2022_30776_MOESM3_ESM.pdf]

nature portfolio

Corresponding author(s): Etienne HERZOG

Last updated by author(s): Apr 6, 2022

Reporting Summary

Nature Portfolio wishes to improve the reproducibility of the work that we publish. This form provides structure for consistency and transparency in reporting. For further information on Nature Portfolio policies, see our Editorial Policies and the Editorial Policy Checklist.

Statistics

For all statistical analyses, confirm that the following items are present in the figure legend, table legend, main text, or Methods section.

n/a	Confirmed	
☐	☒	The exact sample size (n) for each experimental group/condition, given as a discrete number and unit of measurement
☐	☒	A statement on whether measurements were taken from distinct samples or whether the same sample was measured repeatedly
☐	☒	The statistical test(s) used AND whether they are one- or two-sided<br>*Only common tests should be described solely by name; describe more complex techniques in the Methods section.*
☐	☒	A description of all covariates tested
☐	☒	A description of any assumptions or corrections, such as tests of normality and adjustment for multiple comparisons
☐	☒	A full description of the statistical parameters including central tendency (e.g. means) or other basic estimates (e.g. regression coefficient) AND variation (e.g. standard deviation) or associated estimates of uncertainty (e.g. confidence intervals)
☐	☒	For null hypothesis testing, the test statistic (e.g. F, t, r) with confidence intervals, effect sizes, degrees of freedom and P value noted<br>*Give P values as exact values whenever suitable.*
☒	☐	For Bayesian analysis, information on the choice of priors and Markov chain Monte Carlo settings
☒	☐	For hierarchical and complex designs, identification of the appropriate level for tests and full reporting of outcomes
☐	☒	Estimates of effect sizes (e.g. Cohen's d, Pearson's r), indicating how they were calculated

Our web collection on statistics for biologists contains articles on many of the points above.

Software and code

Policy information about availability of computer code

Data collection	Sorts data collection: BD FACSDiva v9.0.1 Software. Immuno cyto fluorescence acquisition: Metamorph 7.10.4.407. STED: LAS X 3.5.7.23225. WES data collection: Compas for SW 4.0.0. Proteomics data collection: Xcalibur 4.3
Data analysis	Sorts data analysis: FlowJo 10. Immunofluorescence: FlowJo 10, ImageJ 2.3.0 and Macro https://github.com/fabricecordelieres/IJ-Toolset_SynaptosomesMacro Version 10 and https://github.com/flevet/RandomizerColocalization. WES data collection: Compass for SW 4.0.0. Proteomics analysis: Proteome Discoverer 2.5, Python 3.7 and BioInfoKit 0a48410 https://github.com/reneshbedre/bioinfokit . Statistical analysis: Graphpad Prism 9.3.1 .

For manuscripts utilizing custom algorithms or software that are central to the research but not yet described in published literature, software must be made available to editors and reviewers. We strongly encourage code deposition in a community repository (e.g. GitHub). See the Nature Portfolio guidelines for submitting code & software for further information.

Data

Policy information about availability of data

All manuscripts must include a data availability statement. This statement should provide the following information, where applicable:

- Accession codes, unique identifiers, or web links for publicly available datasets
- A description of any restrictions on data availability
- For clinical datasets or third party data, please ensure that the statement adheres to our policy

All data supporting the findings of this study are provided within the paper and its supplementary information. DA-FASS Proteomic dataset is publicly available at the ProteomeXchange Consortium http://proteomecentral.proteomexchange.org/cgi/GetDataset via the PRIDE partner repository with the dataset identifier PXD027534. The mouse brain proteome is available at http://www.mousebrainproteome.com/. SYNGO database is available at https://syngoportal.org/index.html.

DropViz database is available at http://dropviz.org/. KEGG database is available at https://www.genome.jp/kegg/pathway.html. Source data are provided with this paper as Source data file. The WES, FACS and immunofluorescence data generated in this study have been deposited in the Zenodo database (DOI: https://doi.org/10.5281/zenodo.6482952). Because of their size (more than 320 Gb) the raw microscopy images underlying the results will be made available upon request to the corresponding authors. Requests will be answered within a week.

Field-specific reporting

Please select the one below that is the best fit for your research. If you are not sure, read the appropriate sections before making your selection.

☒ Life sciences ☐ Behavioural & social sciences ☐ Ecological, evolutionary & environmental sciences

For a reference copy of the document with all sections, see nature.com/documents/nr-reporting-summary-flat.pdf

Life sciences study design

All studies must disclose on these points even when the disclosure is negative.

Sample size	No estimate of the needed statistical power was established prior to experiments. Experiments were performed at least on 2 independent sorts, most of the times 3 independent sorts. With a pool of at least three animals per sample, thousands of particles analyzed per field of view, 3 independent replications seemed good enough to compensate for possible experimental bias.
Data exclusions	Synaptosomes detected during image analysis were excluded when overlapping biological material prevented from an accurate quantification or when the focus was not good enough for accurate quantification.
Replication	Experiments were performed at least on 2 independant sorts, most of the times 3 independant sorts. When possible experimental results were confirmed using a different method (WES immunoblots, different sort criteria like VGLUT1venus for instance). Each replication attempt has been successful in reproducing the data.
Randomization	As our control condition is coming from the same sample pool "sorted" vs "unsorted" no subject randomization was performed in our study. Monte-carlo simulation randomization was applied during the immunofluorescence analysis of distances between proteins of interest.
Blinding	As we did not use any drug treatment in this study and because most of our analysis is automated we did not perform blind experiments. For immunofluorescence and immunoblot (simple western, WES) , data collection was also automated avoiding a potential operator bias.

Reporting for specific materials, systems and methods

We require information from authors about some types of materials, experimental systems and methods used in many studies. Here, indicate whether each material, system or method listed is relevant to your study. If you are not sure if a list item applies to your research, read the appropriate section before selecting a response.

Materials & experimental systems

n/a	Involved in the study	
☐	☒	Antibodies
☒	☐	Eukaryotic cell lines
☒	☐	Palaeontology and archaeology
☐	☒	Animals and other organisms
☒	☐	Human research participants
☒	☐	Clinical data
☒	☐	Dual use research of concern

Methods

n/a	Involved in the study	
☒	☐	ChIP-seq
☐	☒	Flow cytometry
☒	☐	MRI-based neuroimaging

Antibodies

Antibodies used	SynCAM 2 monoclonal antibody raised in rat against an epitope in the extracellular domain (Clone 3E1, provided by Thomas Biederer, unpublished). Anti-D1 receptor, goat polyclonal antibody (Frontier Institute Cat# D1R-Go-Af1000, RRID: AB_2571594). Guinea pig polyclonal antibody to: VGLUT2 (Millipore Cat# AB2251, RRID:AB_1587626); VGLUT1 (Millipore Cat# AB5905, RRID:AB_2301751); MGL (Frontier Institute Cat# MGL-GP, RRID:AB_2716807); Homer 1 (Synaptic Systems Cat# 160 004, RRID:AB_10549720). Mouse monoclonal antibody to GFP (Roche Cat# 11814460001, RRID:AB_390913, Clone 7.1 and 13.1); Munc-18 (BD Biosciences Cat# 610336, RRID:AB_397726, Clone 31/Munc-18); Synaptophysin 1 (Synaptic Systems Cat# 101 011C3, RRID:AB_887822, Clone 7.2); Tyrosine Hydroxylase (Millipore Cat# MAB318, RRID:AB_2201528, Clone LNC1), PSD-95 (Abcam Cat# ab2723, RRID:AB_303248, Clone 6G6-1C9). Rabbit polyclonal antibodies to: GluA1 (Millipore Cat# AB1504, RRID:AB_2113602) ; Tyrosine Hydroxylase (Synaptic Systems Cat# 213 102, RRID:AB_2619896); D2 dopamine receptor (Millipore Cat# ABN462, RRID:AB_2094980); Synapsin 1/2 (Synaptic Systems Cat# 106 002, RRID:AB_887804); VGLUT2 (Cat# VGLUT2, RRID:AB_2315563)47; Sybnaptopodin (Synaptic Systems Cat# 163 002, RRID:AB_887825); VAChT (Synaptic Systems Cat# 139 103, RRID:AB_887864); VIAAT/VGATs (Synaptic Systems Cat# 131 002, RRID:AB_887871); DAT (Millipore Cat# AB2231, RRID: AB 1586991); EAAT1/GLAST (Cat# Ab#314, RRID:AB_2314561 a kind gift by Niels Christian Danbolt, University of Oslo); GFP (Abcam Cat# ab290, RRID:AB_303395); Cpne7 (OriGene Cat# TA334534); Mint

1 (Synaptic Systems Cat# 144 103, RRID:AB_10635158); Cadps2 (Synaptic Systems Cat# 262 103, RRID:AB_2619980); Syntaxin 4 (Synaptic Systems Cat# 110 042, RRID:AB_887853);. Anti-Tyrosine hydroxylase, chicken antibody (Millipore Cat# AB9702, RRID:AB_570923); Goat polyclonal Anti Mouse Alexa 488 (Thermo Fisher, Cat# A-11001, RRID: AB_2534069 , Lot 2379467) ; Goat polyclonal Anti Rabbit Alexa 568 (Thermo Fisher, Cat# A-11011, RRID:AB_143157, Lot 1778025) ; Goat polyclonal Anti-Chicken Alexa 647 (Thermo Fisher, Cat# A-21449, RRID:AB_2535866, Lot 2186435) ; Goat polyclonal Anti-Chicken Alexa 594 (Thermo Fisher, Cat# A-11042, RRID:AB_2534099, Lot 2181000) ; Goat polyclonal Anti Rabbit Atto-647 (Sigma-Aldrich, Cat# 40839, RRID:AB_1137669, Lot BCCF3161) ; Goat polyclonal Anti-Chicken Alexa 488 (Thermo Fisher, Cat# A-11039, RRID:AB_2534096, Lot 2304258) ; Goat polyclonal Anti-Guinea Pig Alexa 647 (Thermo Fisher, Cat# A-21450, RRID:AB_2735091, Lot 2231672) ; Goat polyclonal Anti-Mouse Alexa 647 (Thermo Fisher, Cat# A-21235, RRID:AB_2535804, Lot 2306581) ; Goat polyclonal Anti-Mouse Alexa 568 (Thermo Fisher, Cat# A-11004, RRID:AB_2534072, Lot 1419715) ; Goat polyclonal Anti Rabbit Alexa-647 (Thermo Fisher, Cat# A-21244, RRID:AB_2535812, Lot 1910774) ;Goat polyclonal Anti Rabbit Alexa 488 (Thermo Fisher, Cat# A-11008, RRID:AB_143165, Lot 1705869).

Validation

Rat monoclonal anti-SynCAM 2, No lot number. Validated by our collaborator with the following statement "The monoclonal antibody against SynCAM 2 was raised in rat. Animals were injected with two peptides corresponding to epitopes in the extracellular domain and hybridoma clone 3E1 was selected based on a screen for immunocytochemical detection of heterologously expressed SynCAM 2. Specificity in immunoblotting and immunohistochemical staining applications was confirmed with brain samples from SynCAM 2 conditional KO mice in which the gene was deleted using Emx1-Cre, targeting the majority of neurons in cortex and hippocampus." Validation data sheet is provided.
Goat polyclonal anti-Dopamine D1 receptor Cat# D1R-Go-Af1000, RRID: AB_2571594, No lot number info not possible to retrieve from collaborator. Validated by the manufacturer with pre-absorption peptide https://nittobo-nmd.co.jp/pdf/reagents/D1R.pdf. Tested in mouse.
Guinea pig polyclonal anti-VGLUT2 Cat# AB2251, RRID:AB_1587626, Lot 2879548. Validated by the manufacturer with the following statement “This Guinea Pig polyclonal antibody detects Vesicular glutamate transporter 2. It targets an epitope within 18 amino acids from the C-terminal region” https://www.merckmillipore.com/FR/fr/product/Anti-VGluT2-Antibody,MM_NF-AB2251-I. Tested in mouse.
Guinea pig polyclonal anti-VGLUT1 Cat# AB5905, RRID:AB_2301751, Lot 3616495. Validated by the manufacturer with the following statement “Recognizes Vesicular Glutamate Transporter 1 (VGLUT1, BNPI {Brain specific Na+ dependent inorganic phosphate cotransporter}). The antiserum has been tested on tissue sections from the rat central nervous system (CNS) using immunofluorescence histochemistry. The antiserum mainly labels nerve fibers and terminals. VGLUT1 is an excellent marker for glutamatergic nerve terminals. The staining pattern for the staining obtained with the VGLUT1 antiserum corresponds to the pattern described using other antisera to VGLUT1 (Bellocchio et al., 1998; Fremeau et al., 2001; Fujiyama et al., 2001; Sakata-Haga et al., 2001; Tong et al., 2001; Kaneko et al. 2002; Varoqui et al., 2002). Preabsorption of the VGLUT1 antiserum with immunogen peptide eliminates all immunostaining”. Validated in mice https://dx.doi.org/10.1016%2Fj.cell.2018.02.004.
Guinea pig polyclonal anti-MGL Mgll Cat# MGL-GP, RRID:AB_2716807, Lot Af200. Validated by the manufacturer with the following statement “The lack of immunofluorescent signals with use of the MGL antibody was confirmed in the brain of MGL-knockout mice” https://nittobo-nmd.co.jp/pdf/reagents/MGL.pdf.
Guinea pig polyclonal anti-Homer-1c Cat# 160 004, RRID:AB_10549720, Lot 2-16. Validated by the manufacturer with the following statement “Specific for Homer 1. According to Soloviev et al. (2000), aa 1 - 180 are present in isoforms a, b, c and d”. Tested in mouse.
Mouse monoclonal anti-GFP Cat# 11814460001, RRID:AB_390913, Lot 27575600. Validated by the manufacturer with the following statement “Anti-GFP is tested for functionality and purity relative to a reference standard to confirm the quality of each new reagent preparation”. Species independent.
Mouse monoclonal anti-Munc-18 Cat# 610336, RRID:AB_397726, No lot number info, not possible to retrieve from collaborator. Validated by the manufacturer with the following statement “western blot knockout validation” https://www.labome.com/product/BD-Biosciences/610336.html. Tested in mouse.
Mouse monoclonal anti-Synaptophysin 1 Cat# 101 011C3, RRID:AB_887822, Lot 1-61. Validated by the manufacturer with the following statement ”Specific for synaptophysin 1, no cross-reactivity to other synaptophysins. K.O”. Tested in mouse.
Mouse monoclonal anti-Tyrosine hydroxylase Cat# MAB318, RRID:AB_2201528, Lot 3506431. Validated by the manufacturer with the following statement ”Recognizes an epitope on the outside of the regulatory N-terminus. Recognizes a protein of approximately 59-61 kDa by Western blot. Does not react with the following on Western Blots: dopamine-beta-hydroxylase, phenylalanine hydroxylase, trytophan hydroxylase, dehydropteridine reductase, sepiapterin reductase or phenethanolamine-N-methyl transferase (PNMT)”. Tested in mouse.
Mouse monoclonal anti-PSD-95 Cat# ab2723, RRID:AB_303248, Lot GR3589-1. Validation status unknown, seller recommendations provided in 2012: Immunofluorescence; Immunoprecipitation; Western Blot; Immunocytochemistry/Immunofluorescence, Immunofluorescence, Immunohistochemistry-Fr, Western Blot.
Rabbit polyclonal anti-Tyrosine hydroxylase Cat# 213 102, RRID:AB_2619896, Lot 213102/2. Validated by the manufacturer with the following statement ”Specific for tyrosine hydroxylase without cross-reactivity to tryptophane hydroxylase”. Tested in mouse.
Rabbit polyclonal anti-Dopamine D2 receptor Cat# AB5084P, RRID:AB_2094980, No lot number info, not possible to retrieve from collaborator. Validated by the manufacturer with the following statement ”Specific for Dopamine Receptor D2 short and long form. Dopamine D2 receptor is known to exist in the short (D2S) and the long (D2L) forms that are encoded by splice variants of a single gene and differ only by the presence of an additional 29 amino acid in the intracellular, cytoplasmic loop 3 (1). D2L receptor is a 446 aa, G-protein coupled and transmembrane receptor protein (2). The immunogen peptide shows no significant homology with other dopamine receptors (D1 and D3-D5)”. Tested in mouse.
Rabbit polyclonal anti-Synapsin 1/2 Cat# 106 002, RRID:AB_887804, Lot 106002/16. Validated by the manufacturer with the following statement ” Recognizes Synaptophysin1 and 2 (Synaptoporin) with strong preference for Synaptophysin1 in Western blot”. Tested in mouse.
Rabbit polyclonal anti-VGLUT2 Cat# VGLUT2, RRID:AB_2315563, Lot N/A. Validated in mouse in the following study https://doi.org/10.1002/cne.23268.
Rabbit polyclonal anti-Synaptopodin Cat# 163 002, RRID:AB_887825, Lot 163002/7. Validated by the manufacturer with the following statement ”Specific for Synaptopodin. Fragment used for immunization is present in all 3 isoforms. K.O”. Tested in mouse.
Rabbit polyclonal anti-VAChT Cat# 139 103, RRID:AB_887864, Lot 5-67. Validated by the manufacturer with the following statement ”Specific for VAChT K.O. PubMed: 24027290”. Tested in mouse.
Rabbit polyclonal anti-VIAAT/VGATs Cat# 131 002, RRID:AB_887871, Lot 31002/240. Validated by the manufacturer with the following statement ”Specific for VGAT. K.O”. Tested in mouse.
Rabbit polyclonal anti-DAT Cat# AB2231, RRID: AB 1586991, Lot 3417479. Validated by the manufacturer with the following

statement "Cat. AB2231 recognizes the N-terminus of the reduced and non reduced forms of DAT". Tested in mouse.
Rabbit polyclonal anti-EAAT1/GLAST Cat# Ab#314, RRID:AB_2314561, Lot 1998-07-29 Tested in mouse. Production date: 1998-07-29; Host animal ID number: 8D0161; Peptide name: A522-541. Used in multiple studies that are accessible here https://pubmed.ncbi.nlm.nih.gov/?term=19328838%2C21800304%2C21853059%2C22859703%2C24099732%2C25834045%2C25914127%2C26875663%2C29303644%2C29350434%2C29530756%2C30053506%2C9972824%5Buid%5D.
Rabbit polyclonal anti-GluA1 Cat# AB1504, RRID:AB_2113602, Lot 2207172. Validated by the manufacturer with the following statement "This antibody recognizes Glutamate receptor 1 at the cytoplasmic domain". Tested in mouse.
Rabbit polyclonal anti-GFP Cat# ab290, RRID:AB_303395, Lot GR3251545-1. Statement of validation "External validation for lot# GR158277-1 is available under ENCODE ID: ENCAB976SGG. This antibody is reactive against all variants of Aequorea victoria GFP such as S65T-GFP, RS-GFP, YFP and EGFP. Species independent. Info: Independent validation by the NYU Lagone was performed for: IHC"
Rabbit polyclonal anti-Cpne7 Cat# TA334534, RRID: N/A, Lot QC28458/40659. Validated on KO in the following paper https://doi.org/10.1016/j.neures.2020.04.002. Tested in mouse
Rabbit polyclonal anti-Mint 1 Apba1 Cat# 144 103, RRID:AB_10635158, Lot 4. Validated by the manufacturer with the following statement "Specific for Mint 1". Tested in mouse.
Rabbit polyclonal anti-Cadps2 Cat# 262 103, RRID:AB_2619980, Lot 2. Validated by the manufacturer with the following statement "Specific for CAPS 2, no cross-reactvity to CAPS 1 K.O". Tested in mouse.
Rabbit polyclonal anti-Syntaxin 4 Cat# 110 042, RRID:AB_887853, Lot 21. Validated by the manufacturer with the following statement "Specific for syntaxin 4". Tested in mouse.
Rabbit polyclonal anti-Bassoon Cat# 141 003, RRID:AB_887697, Lot 1-25. Validated by the manufacturer with the following statement "Specific for bassoon". Tested in mouse
Rabbit polyclonal anti-RIM1 Cat# 140 013, RRID:AB_2238250, Lot 2-13. Validated by the manufacturer with the following statement "Specific for RIM 1, no cross reactivity to RIM 2. K.D. PubMed: 29230050". Tested in mouse.
Chicken polyclonal anti-Tyrosine hydroxylase Cat# AB9702, RRID:AB_570923, Lot 3616562. Validated by the manufacturer with the following statement "Recognizes Tyrosine Hydroxylase". Tested in mouse
Chicken polyclonal anti-GFP Cat# ab13970, RRID:AB_300798, Lot GR3361051-2. Validated by the manufacturer with the following statement"Our GFP antibody does cross-react with the many fluorescent proteins that are derived from the jellyfish Aequorea victoria. These are all proteins that differ from the original GFP by just a few point mutations (EGFP, YFP, mVenus, CFP, BFP etc.)". Species independent.

Animals and other organisms

Policy information about studies involving animals; ARRIVE guidelines recommended for reporting animal research

Laboratory animals
11 to 15 weeks male and female transgenic mouse expressing cre recombinase under the control of the dopamine transporter (DAT) were used (DAT-Cre strain), or VGLUT1venus strain. Mice were maintained in C57BL/6N background and housed in 12/12 LD with ad libitum feeding, 50-70% humidity and 18 to 22° C ambient temperature. Autofluorescence controls for FACS were performed using male or female Wild-Type littermate.

Wild animals
The study did not involve Wild animals

Field-collected samples
The study did not involve field-collected samples

Ethics oversight
The experimental design and all procedures were in accordance with the European guide for the care and use of laboratory animals and approved by the ethics committee of Bordeaux University (CE50) and the French Ministry of Research under the APAFIS n° 8944 and #21132.

Note that full information on the approval of the study protocol must also be provided in the manuscript.

Flow Cytometry

Plots

Confirm that:

☒ The axis labels state the marker and fluorochrome used (e.g. CD4-FITC).

☒ The axis scales are clearly visible. Include numbers along axes only for bottom left plot of group (a 'group' is an analysis of identical markers).

☒ All plots are contour plots with outliers or pseudocolor plots.

☒ A numerical value for number of cells or percentage (with statistics) is provided.

Methodology

Sample preparation
The preparation of sucrose synaptosomes was adapted from a previously published protocol 25. Briefly, animals were euthanized by cervical dislocation, decapitated and the head was immersed in liquid nitrogen for a few seconds. The striatum of WT and bright fluorescent parts of the striatum of DAT-cre+ mice were subsequently dissected under an epi-fluorescence stereomicroscope (Leica Microsystems, Germany, Figure 1 Panel 2). Non-fluorescent control striata were dissected following anatomical borders. Samples were then homogenized in 1ml of ice-cold Isosmolar buffer (0.32M sucrose, 4mM HEPES pH7.4, protease inhibitor cocktail Set 3 EDTA-free (EMD Millipore Corp.)) using a 2ml-glass-Teflon homogenizer with 12 strokes at 900 rpm. The homogenizer was rinsed with 250µL of isosmolar buffer and 3 manual strokes and then, the pestle was rinsed with additional 250µl of isosmolar buffer. The final 1.5ml of homogenate (H) was centrifuged at 1000xg for 5min at 4°C in a benchtop microcentrifuge. The supernatant (S1) was separated from the pellet (P1) and centrifuged at 12,600xg for 8min at 4°C. The supernatant (S2) was aliquoted and the synaptosomes-enriched pellet (P2) was resuspended in 350 µL of isosmolar buffer and layered on a two-step ficoll density gradient (5mL of 13% Ficoll, 0.32M sucrose, 4mM HEPES and 5mL of 7,5%

Ficoll, 0.32M sucrose, 4mM HEPES). The gradient was centrifuged at 16,000 rpm for 1 hour and 10 min at 4°C (Thermo Sorvall WX Ultra 90 with a TH 641 rotor). The synaptosome fraction (Syn) was recovered at the 7.5 and 13% ficoll interface using a 0.5ml syringe. After collection, sucrose/ficoll synaptosomes were stored on ice and sequentially diluted in ice-cold PBS with protease inhibitor as described above, and the lipophilic dye FM4-64 dye was added at 1μg/ml to the solution to red label all membrane particles.

Instrument

The FACSAria-II from BD Biosciences was used to collect and analyze the flow Cytometry data.

Software

The data was collected with FACSDiva v9.0.1 and analysed using Flowjo 10.

Cell population abundance

Quality of the sort was systematically monitored by sample reanalysis and reported in the main document or supplemental figures.

Gating strategy

Detection of events was calibrated on a FM4-64 fluorescence threshold. A "Singlets" gate was empiricaly established (through trial and error repetitions) on FSC/SSC plots to gate all singlet events in the sample. Within "singlets", EGFP positives and negatives gates were established using a non fluorescent control synaptosome sample. EGFP+ events were sorted.
The gating strategy is described in details in supplemental figures.

☒ Tick this box to confirm that a figure exemplifying the gating strategy is provided in the Supplementary Information.
